# Oral Presentations

**DOI:** 10.1155/1996/78528

**Published:** 1996


### ORAL PRESENTATIONS

### Topic: LIVER

### F001

### UNRESECTABLE HEPATIC METASTASES FROM COLORECTAL CANCER: RESULTS OF A COMBINED APPROACH BY CHEMOTHERAPY AND SUBSEQUENT RESECTION

R. Adam, H. Bismuth, Ch. Farabos, F. Lévi, F. Waechter
Hepatobiliary Surgery and Liver Transplant Center, Paul Brousse Hospital, Villejuif, France

Resection is the sole curative treatment of hepatic metastases from colorectal cancer. However, it may be achieved in only 10% of patients (pts) since most pts have at the first irresectable lesions associated with a poor prognosis. Over the past 6 years, we have managed these pts with a new protocol of chemotherapy with the aim to perform subsequent curative liver resection. From April 1988 to March 1994, 53 out of 337 pts (16%) with liver metastases initially considered as non resectable were subsequently submitted to hepatic resection with a curative intent. All pts have been treated by intravenous chronomodulated chemotherapy combining 5 Fluorouracil, Folinic acid and Oxaliplatinum, a non nephrotoxic platinum complex. To optimize dose intensities and tolerance, drug delivery was sinusoidally modulated along the 24 hour-scale with peak flow rates at 04.00 hours for 5-FU and Fol and at 16.00 hours for Oxa, using an ambulatory programmable-in-time pump. Initial non resectability was assessed by the same surgical team and was related either to technical impediment due to large (n=8), multinodular (n=24) and central ill-located tumours (n=8) or to the presence of extrahepatic disease (n=13 - Peritoneum (6), Epiploon (3), Lungs (4)). Pts received 3 to 29 courses of chemotherapy (mean =10) for 2 to 29 months (mean= 8 months) before surgery.
**Results**: An objective reduction in tumour size was observed following chemotherapy in all pts subsequently submitted to liver resection. A significant reduction of tumor markers was also demonstrated. A major hepatectomy ($\geq$ 3 segments) was performed in 37 pts and a minor resection in 16. There was no operative mortality within 2 months. Post operative complications included 2 infected collections that needed non operative drainage, 1 transient biliary fistula and 1 reoperation for bleeding. Chronomodulated chemotherapy was routinely continued post operatively in all pts for 6 courses at less. Associated procedures included repeat hepatectomy (15), pulmonary resection (11), hepatic cryotherapy (8), splenectomy (1) nephrectomy (1), resection of the diaphragm (2), repeat resection of colon cancer recurrence (2). Twenty eight pts are presently alive (of whom 16 without disease) with a mean follow of 2.5 years (range 1.3 - 6.4). Median survival is 3.2 years with a patient survival rate of 61% at 3 years.
**Conclusion**: Resection may be achieved in some unresectable pts with the help of an efficient chemotherapy. The benefit in survival seems comparable to that obtained with liver resection for initially resectable liver metastases. This therapeutic strategy involves a multimodality approach including repeat hepatectomy and extrahepatic surgery.

### F002

### IMPACT OF PREVIOUS VARICEAL BLEEDING ON SUBSEQUENT LIVER TRANSPLANTATION IN CIRRHOTIC PATIENTS.

R. Adam, J. Raccuia, H. Bismuth.
Hepatobiliary Surgery and Liver Transplant Center, Paul Brousse Hospital, Villejuif, France

In order to establish a treatment strategy based on the perspective of liver transplantation, we reviewed 1000 consecutive liver transplants over a 10 year period with special emphasis on prior history of portal hypertension with variceal hemorrhage.
The impact of bleeding and subsequent therapy was analyzed to integrate the proper timing of liver transplant in the multimodality therapy of portal hypertension. Transplantation for fulminant hepatitis (n=148) and re-transplantation (n=122) were excluded. Of 730 primary transplanted patients with chronic liver diseasc, 544 (74%) had no prior history of variceal hemorrhage. There were 186 (26%) patients with variceal bleed prior to transplantation of which 130 (70%) required interventional therapy to palliate the bleeding. In the two groups of patients with and without bleed prior to transplantation there were no significant differences in regards to age, sex, etiology of the liver disease or donor liver morphology. Sclerotherapy was performed in 93 (50%), surgical portal diversion in 27 (15%) and TIPS in 10 (5%) patients. Moderate to severe liver dysfunction (grade B and C of a modified Child classification) accounted for 91 percent of the patients with bleeding complications. The overall survival for all patients was 76 percent at five years. Previous history of variceal bleeding alone or treatment by initial sclerotherapy demonstrated no significant difference with either graft or patient survival. The patients who had TIPS to control hemorrhage had lower, but insignificant, graft and patient survival. The group of patients with variceal hemorrhage who had prior surgical shunt did, however, demonstrate a significant increased survival at five years when compared to the non-shunted group (96% versus 73%, $p<0.007$). In conclusion, the impact of variceal bleeding does not seem to be critical to subsequent liver transplantation. In contrast to sclerotherapy and TIPS, portocaval shunt demonstrated an improved outcome following liver transplantation. The perspective of liver transplantation should not be a contraindication to perform portocaval shunting in properly selected patients.

### F003

### ROLE OF NITRIC OXID IN ACUTE LIVER INJURY AND THE ASSOCIATED BACTERIAL TRANSLOCATION

[1]D. Adawi, [1]F.B. Kasravi, [2]G. Molin, [1]B. Jeppsson

[1]Department of Surgery, [2]Department of Food Technology, Lund University, Lund, Sweden

The effect of nitric oxid in acute liver injury at different time intervals was evaluated in an acute liver injury model induced by D-galactosamine (1.1 gm/kg body wt.) intraperitoneally. Rats were divided into 4 groups: normal control, acute liver injury, acute liver injury + $N^G$-nitroL-arginine methyl ester (L-NAME) and acute liver injury + L-NAME + L-arginine. After 6 hours of the liver injury, the Alkaline Phosphatase (ALP), Bilirubin (BIL), Aspartate Aminotransferase (ASAT) and Alanine Aminotransferase (ALAT) increase in the acute liver injury + L-NAME group compared to the acute liver injury control group with significant difference in ALP (P-<0.01), BIL (P<0.05) and ASAT (P<0.05). The acute liver injury + L-NAME + L-arginine group show reduced levels of ALP, BIL, ASAT and ALAT compared to acute liver injury + L-NAME group, with a significant difference in ALP (P<0.05). After 12 hours the inhibition of nitric oxid increase the level of liver enzymes and translocated bacteria but without significant difference. After 24 hours in the acute liver injury + L-NAME group there is a significant increase in BIL (P<0.05) compared to acute liver injury group. The acute liver injury + L-NAME + L-arginine show significant reduction in the level of ALP (P<0.05) compared to acute liver injury + L-NAME group. The number of the translocated bacteria to arterial blood, portal blood, liver and mesenteric lymph nodes at all time intervals increased in acute liver injury + L-NAME groups compared to acute liver injury group with a significant difference in the arterial blood after 24 hours (P<0.05) and a decreased number in acute liver injury + L-NAME + L-arginine groups compared to acute liver injury + L-NAME groups with a significant difference in the arterial blood after 24 hours (P<0.05). These show that inhibition of nitric oxid increase the number of the translocated bacteria and potentiate the liver injury.

### F004

### PREOPERATIVE ALBENDAZOLE TREATMENT FOR LIVER HYDATID DISEASE DOES NOT AFFECT THE VIABILITY OF THE CYST

**AKTAN A.Ö., YALIN R.**
Marmara Univ. School of Medicine,Dept.of General Surgery, İstanbul, TURKEY

The treatment of hydatid cysts of the liver is still primarily surgical. The surgical treatment of this disease, however, is far from ideal. Intraoperative spillage and postoperative recurrence around 10 % are yet unresolved problems. Although in experimental models the efficacy of albendazole has been demonstrated, clinical data are still lacking. In addition to the gross appearance of the cyst, the intracystic pressure (ICP) has also been found to be a reliable guide for the assessment of viability. High intracystic pressures are found in viable cysts.

In this study a three week course of preoperative albendazole (10 mg/kg) was given to patients with liver hydatid cysts and the intraoperative viability of the cyst assessed.

The study consisted of two groups and the first group had 15 patients (5male, 10 female)with a median age of 31 (21-75). All cysts were located in the liver. In two patients the cysts were grossly degenerated and the ICP was 0. In two others the cysts were partly degenerated and the ICPs were 4 and 5.5 cm. In the remaining patients the mean ICP was 27 cm H2O (range 8-42 cm H2O). Direct microscopy with eosin exclusion test revealed viable scolices in four patients in whom the test was performed.

The second group consisted of 40 patients with liver hydatid cysts without any preoperative treatment. In this control group, there were 9 non-viable (mean ICP 0cm H2O) and 31 viable cysts (mean ICP 35cm H2O). The differences among the groups were not significant ($p< 0.05$).

It is concluded that a three week course of preoperative albendazole treatment does not effect the viability of the hydatid cyst.

### F005

### IN-VITRO ASSESSMENT OF UPTAKE AND CYTOTOXICITY OF LIPIODOL AND OTHER FATTY ACIDS IN PRIMARY AND METASTATIC LIVER CANCERS

**R. A. M. Al-Mufti**, **J. LEWIN**, **M. C. WINSLET**, **K. E. F. HOBBS**
**University Department of Surgery, Royal Free Hospital School of Medicine, Pond Street, London NW3 2QG, ENGLAND**

Lipiodol, an iodinated poppy seed oil, has been shown to be taken up and retained selectively by primary and some metastatic liver cancers, following injection into the hepatic artery. The mechanism of uptake, retention and cytotoxicty of Lipiodol, constituent and non-constituent fatty acids by primary and colorectal hepatic metastases in tissue cultures were assessed using growth curves, trypan blue & LDH assays, $^{3}$H-leucine uptake and electrpn microscopy. Cultures of Hep-G2 (human hepatoma), LoVo (human hepatic colorectal cancer), SW620 (human metastatic colorectal cancer) cell lines were studied. Control non-malignant cell lines were used in the form of human hepatocytes, HUVEC (human umbilical vein endothelial cells) and U937 (histiocytic lymphoma) cell lines. The cytotoxic effects of Lipiodol were compared with that of its constituent fatty acids (Linoleic, Oleic, Palmitic and Stearic acids). Other fatty acids used were iodinated Linoleic, Docosahexanoic and Eicosapentanoic acids. The cultures were exposed to different concentrations (1%, 2% and 4% v/v) for a variable duration (3, 6, 12, 24, 48 & 72 hours). All the cell lines (malignant and non-malignant) have shown intra-cytoplasmic incorporation of membrane-bound lipid vesicles when exposed to Lipiodol or other fatty acids. There was a linear increase in the uptake in relation to prolonged period of exposure to the fatty acids. The non-malignant cells managed to void their contents of Lipiodol or other fatty acids as shown by image analysis, but the malignant cell lines did not. This may explain why liver cancers selectively retain Lipiodol in-vivo.

Lipiodol had no effect on the cell growth and viability, LDH release or protein synthesis. Similar effects were seen with Oleic and Palmitic acids. However, Linoleic and Stearic acids were very toxic to all the malignant cell lines but not to the non-malignant controls. Iodinated Linoleic, Docosahexanoic and Eicosapentanoic acids were cytotoxic to all the cell lines (malignant and non-malignant). This study revealed that there was uptake and retention of Lipiodol and all other fatty acids. Some fatty acids were only toxic to cancer cells while others were toxic to all types of cells. The use of Linoleic acid or Stearic acid in targeting liver cancers should be investigated in animal models. This may prove to have great therapeutic benefits in targeting therapy for liver cancers.

### F006

### RADICAL SURGERY FOR LIVER HYDATID DISEASE

S.Alfieri, GB Doglietto, C.Carriero, F.Pacelli, G.Costamagna, M. Liberatori, F.Crucitti
Department of Surgery, Catholic University, Rome, Italy.

BACKGROUND. Surgical treatment of hydatid liver disease remains controversial. METHODS The outcome of 90 consecutive patients who underwent surgical treatment in the period 1983-1995 for hepatic hydatid disease was reviewed. It has been also analysed the therapeutic approach in the management of biliary fistula. Total "closed" cystopericystectomy was performed as the first choice procedure. Hepatic resection was performed in cases where single large or multiple cysts had destroyed a segment or an entire lobe of the liver. The residual cavity was left opened and drained with an external tube. Endoscopic retrograde cholangiography (ERC) has been adopted to detect and treat biliary tract involvement.
RESULTS A total of 122 cysts in 90 patients were surgically treated. Total cystopericystectomy was adopted in 62 patients (70%); in 4 additional patients with a deep intraparenchymal cysts adherent to large vessels (inferior vena cava - n. 3) or to hilar structures (n.1), a small portion of poorly vascularized pericyst was left in place (subtotal pericystectomy). Total "closed" cystopericystectomy was performed in 58 patients; 8 cases were treated with open cystopericystectomy.The remaining 23 patients (25.8%) underwent hepatic resection. We observed 15 cases of cystobiliary communication: 8 were discovered by ERC in symptomatic patients and treated preoperatively by endoscopic sphincterotomy and 7 were discovered and treated during operation. The mean (s.d.) operating time was 214.6±76 (range 70-450) minutes: 209.2±61 (range 70-350) minutes for cystopericystectomy (n.66) and 227±103 (range120-450) minutes for liver resection (n.23-p=0.42). Thirty-six patients (40%) required intraoperative blood transfusion: mean 1520±1125cc (min250-max3000) and 1141±669cc during liver resection (n.12) and cystopericystectomy (n.24) respectively (p=0.2). The overall incidence of postoperative complications was 19%: 19% and 17% after cystopericystectomy and liver resection respectively (p =0.32). In case of an external post-operative biliary fistula, cure was achieved through endoscopic sphincterotomy and introduction of a temporary nose-biliary stent.Post-operative stay was 14±10 (range7-65) days with no differences between cystopericystectomy and liver resection:14±11 (range7-65) and 14±8 (range7-35) days respectively (p=0.9). Overall postoperative mortality was 1%. Among the 72 patients available for follow-up, only one (1%) had a local recurrence of the disease. Sixteen patients (18%) were lost at follow-up.
CONCLUSIONS Results suggest the safety and efficacy of radical procedures in surgical management of liver hydatid disease. Total cystopericystectomy is the treatment of choice. Liver resection is justified in selected cases. ERC is a valuable tool for diagnosis and treatment of biliary complications.

### F007

### OMENTALLY ADMINISTERED PROSTAGLANDIN $E_1$ EFFECTIVELY INCREASES PORTAL VENOUS BLOOD FLOW IN PARTIALLY HEPATECTOMIZED RATS

T. Aono, K. Tsukada, T. Sakaguchi, M. Ohtake, D. Ishiduka, N. Fujita, K. Hatakeyama
Departments of Surgery and Physiology, Niigata University School of Medicine, Niigata, Japan

Portal venous blood flow (PVF) and systemic arterial blood pressure (SAP) were recorded after prostaglandin $E_1$ (PGE) administration to the greater omentum and femoral vein in 66 percent hepatectomized rats. Twenty-four male rats were used. The PVF increased when PGE was given to the omentum and femoral vein at 7.5 μg/kg/min for 2 min. The magnitude of PVF response after both administrations was dose dependent, but the duration of PVF response in the omental application was longer than in the femoral administration. The femoral injection reduced SAP synchronistically with an increase in PVF, while omental application caused no change in SAP. These findings suggest that the omentum is a better site for PGE administration in the hepatectomized condition, and that omental PGE delivery is efficacious in increasing PVF without systemic circulatory change.

### F008

### DOES FORMATION OF THE CAVERNOUS PORTAL VEIN AFTER COMPLETE THROMBOSIS IMPROVES PORTAL LIVER INFLOW?

V. Artiko, K. Kostić, M. Stojković, B. Davidović, V. Obradović;

Institute of Nucl. Med. CCS, School of Med., Beograd-YU

The aim of the study is the estimation of the liver perfusion in patients with complete portal venous thrombosis (CT, n=7) and in those, where, consequently, cavernous portal vein was developed (CPV, n=7), as well as comparison to the physiological values (C, n=11). Dynamic liver and spleen scintigraphy was performed with bolus injection of (740 MBq) 99m-Tc-pertehnetate (60f/60sec).

The physiological arterial phase of the liver vascularisation was X+/-SD=8+/-1sec, while in CT (n=5) and CPV it was prolonged (p < 0.01) and lasted 12+/-3sec and 10+/-2sec respectively, without the differences between the last two groups (p > 0.05). Portal phase in C was 12+/-3 sec, in CT (n=5) wasn't registered (p <0.01), while in CPV it was prolonged (21+/-11 sec) in comparison to C (p < 0.05) and CT (p < 0.01). Relative liver portal perfusion (HPI, Sarper's method), in C was 0.67+/-0.06, while in CT (n=5) was not registered at all (p < 0.01). By formation of the CPV, portal inflow was improved (p < 0.05) (0.32+/-0.23) comparing to CT, but it remained lower than physiological (p < 0.01). However, in 2/7 patients with CT, with hepatopetal inflow through peribiliary varices (Doppler-US), arterial phase of the liver vascularisation lasted 9 sec, portal one was prolonged (34, 37 sec) and HPI was 0.37 and 0.35. Lienal radionuclide angiogram in CT, and in 5/7 CPV, showed horisontal venous phase, pointing out impaired blood outflow to the portal vein.

According to the results obtained, by formation of the collateral circulation (CPV, peribiliary varices), after complete portal thrombosis, arterial liver phase shortens, portal phase is being prolonged and portal inflow increased. However, all of presented values; do not reach physiological ones. Thus, formation of the CPV improves portal liver blood flow, while splenic outflow remains impaired.

F009

### PERCUTANEOUS ASPIRATION OF THE HEPATIC HYDATID CYSTS

E.Aygün, A.Kartal, Ö.Karahan, K.Ödev, S.Yol, C.Er

Department of Surgery, University of Selçuk, Konya, Turkey

Forty hepatic hydatid cysts of 30 patients were treated by percutaneous aspiration method between May 1992 and November 1995. Ultrasonography (US), computerized tomography (CT), Latex agglutination and Elisa tests were used either for diagnosis or post drainage follow-up. Seventeen patients were female, and 13 were male, the ages ranged between 7 and 68 years (mean, 42 years). Twentyseven cases had only one cyst and 3 cases had more than one. Half volume of the cysts were aspirated under the guidance of US or CT and 0.5 % silver nitrate solution was injected into the cyst as well as aspirated fluid. Five minutes later, cyst cavity was aspirated totally and the catheter was withdrawn. Three patients had allergic reactions (mild cyanosis in two and bronchospasm in one patient) due to silver nitrate injection, not necessarily to quit the procedure. The patients were administered Albendazole (10 mg/kg/day) orally, for 13 days, 3 days before the application and 10 days following the aspiration. Totally 5 secondary aspiration was necessary in three patients in the early post drainage period because of insufficient evacuation of the cysts. The mean follow-up period was 27 months. Recurrence was seen only in one patient with abdominal cyst which was also treated percutaneously.

We concluded that, percutaneous aspiration of the hepatic hydatid cysts was an effective and an alternative method to the surgery in convenient cases.

F010

### THE SPLIT LIVER IN LIVER TANSPLANTATION: A RECENT EXPERIENCE

D. Azoulay, I.Astarcioglu, M. Johann, R. Adam, D. Castaing, H. Bismuth
Hepato-Biliary Surgery and Liver Transplant Center, Paul Brousse Hospital, Villejuif, France

The good results of liver transplantation (LT) have allowed its larger use. However this increased use of LT has uncovered a relative organ shortage. The split liver is one of the means to increase the number of available grafts. We report here our recent experience with this technique. From January to December 1995, a systematic proposal to perform a split was made to the French organ sharing network as often as possible. Ninety LT were performed using 61 whole grafts, 27 split liver grafts, 2 reduced size grafts. Twenty livers were splitted at our center generating 40 split liver grafts: 23 transplanted at our center in 23 patients and 17 shipped to other centers. We received 4 split liver grafts from 4 livers splitted in other centers. Our 27 patients were transplanted for cirrhosis in 19 cases, amyloid polyneuropathy in 6 cases and fulminant hepatitis in 2 cases. Operative mortality (< day 60 post-LT) occured in one case of fulminant hepatitis and long term mortality (> day 60 post LT) occured in 1 case of cirrhosis. One patient was retransplanted at day 6 for primary non function due to a too small graft (the lowest liver to recipient weight ratio = 0.87).Patient and graft actuarial survival are respectively 91.4 ± 5.8% and 87.5 ± 6.8%. Twelve technical complications occured in 10 patients: 3 arterial complications (2 thrombosis and 1 dissection) of which 2 were successfully treated by urgent desobstruction; biliary fistula : 4 cases, biliary stenosis: 2 cases; hemoperitoneum: 2 cases, segment 4 necrosis: 1 case. Eight of these complications needed surgery to be controlled.
Conclusion : 111 LT were performed with 87 livers realising an economy of 24/111 grafts (22%). During the same period, 16 proposals of split were refused in France. The graft economy would have been of 28%. When used for elective transplantation ,the split LT gives good results comparable to those of whole LT. Our results promote the use of split LT every time it is possible.

F011

### VALUE OF TRANSJUGULAR INTRAHEPATIC PORTOSYSTEMIC SHUNT IN CIRRHOTIC PATIENTS AWAITING LIVER TRANSPLANTATION.

D. Azoulay, J. Raccuia, D. Castaing, H. Bismuth
Hepato-Biliary Surgery and Liver Transplant Center, Paul Brousse Hospital, Villejuif, France

From November 1991 to January 1995, 34 transjugular intrahepatic portosystemic shunt (TIPS) were attempted in 34 cirrhotic patients (mean 46.4 ± 2.4; 22 - 66) candidates for liver transplantation (LT). Patients were classified Child class A in 5 cases, B in 11 cases, C in 18 cases. Indication for TIPS was sclerotherapy failure in 23 cases and intractable ascites in 11 cases.Two patients were excluded because of technical failures which were treated by OLT in one case and open calibrated porta-caval shunt in one case. The follow-up with LT as end point was 1 to 34 months (7.6±1.6 M).
Results: Early thrombosis (< 3 months) occurred in 8 cases: 6 were desobstructed via the internal jugular vein and 2 were desobstructed surgically together with calibrated porta-caval shunt. Late thrombosis occurred in 1 case with portal vein thrombosis and was treated by mesenterico-caval shunt followed by LT 6 months later.
Recurrence of hemorrhage occurred in 2/22 patients who underwent TIPS for sclerotherapy failure (one rupture of varices, one duodenal ulcus).Ascites disappeared in 7/10 patients who underwent TIPS for intractable ascites and was controlled together with diuretics in 2 patients. Ascites remained unchanged in 1 patient.
21 patients were transplanted following TIPS with a mean delay 6.4 ± 1.6 (range: 1- 26) months. During the same period, 7 patients with cirrhosis and surgical open porta-caval shunt were transplanted. Comparison of patients with TIPS to patients with surgical open shunt showed a shorter duration of operation for patients with TIPS (332 ± 351 vs 467 ± 480 min, P>0.05), less blood transfusion (3.5 ± 2.1 vs 7.3 ± 2.6 L, P<0.05). Graft and patient survival at 3 months were comparable.
We conclude that TIPS controles the complications of portal hypertension in patients awaiting transplantation. TIPS diminishes blood requirement during liver transplantation procedure.

F012

### PORTAL VEIN EMBOLISATION IN THE STRATEGY OF MAJOR LIVER RESECTION

D. Azoulay, J. Raccuia, D. Castaing, H. Bismuth.
Hepatobiliary Surgery and Liver Transplant Center, Paul Brousse Hospital, Villejuif, France

Portal vein embolization (PVE) is useful with liver tumors when extended hepatic resection is technically feasible, but, the potential for postoperative liver failure prohibits primary surgical treatment. Achieving a "functional hepatectomy" percutaneously, while inducing controlateral hypertrophy, in anticipation of major hepatic resection is the goal of this modality. This technique was applied in 20 patients who were already enrolled in different protocols with neoadjuvant chemotherapy including arterial chemoembolization for primary tumor, and chronomodulated and/or hepatic artery infusion chemotherapy for hepatic metastatic cancer. Although most patients had either primary or metastatic hepatic tumor, one patient had cholangiocarcinoma and another had a neuroendocrine tumor. Liver cirrhosis was associated with tumor in 5 (23%) patients. Final decision to perform hepatic resection was based upon the degree of liver hypertrophy of the future remaining liver by clinical, biologic, volumetric computed tomographic (CT) scan and tumor response to chemotherapy. There were no deaths and one complication. Exploration was performed after PVE in 18 (90%) patients, while 2 (10%) are still awaiting decision to operate. At exploration 5/18 (28%) had disseminated disease and were considered incurable, while the remaining 13 (72%) had hepatic resection with curative intent. Specimens at the time of resection revealed tumor necrosis in 8 (62%) patients (3 with 100% necrosis) and in 5 (38%) patients the resection was less extensive than anticipated. Tumor margins were negative in all patients resected (68%), while the remainder were either inoperable (23%) or are still awaiting surgery (9%). There was a significant increase in serum bilirubin after PVE (p<0.02) and a decrease in lactate dehydrogenase (p=0.05) prior to surgery. There was no significant change in other biochemical liver function tests or with indocyanine gree ($ICG_{R15}$). When primary resection of liver malignancy is not feasible for various reasons, in both cirrhotic and non cirrhotic livers, neoadjuvant PVE which was performed in conjunction with chemotherapy, permitted resection with intent to cure, when otherwise was prohibitive.

### F013

**LIVER TRANSPLANTATION IN CIRRHOTIC PATIENTS WITH RESECTED HEPATOCELLULAR CARCINOMA**

Balsells J, Charco R, Murio E, Lázaro JL, Bilbao I, Hidalgo E, Fdez-Cuadrado T, Margarit C.

Liver Transplant Unit. Hospital Vall d'Hebron. Barcelona. Spain

The main causes of death following liver resection (LR) for hepatocellular carcinoma (HCC) in cirrhotic patients are tumour recurrence and liver failure. The aim of this study was to analyze patientc who underwent liver transplantation (LTX) after LR of their HCC between Jul-87 and Feb-95.
LR was performed in 57 cirrhotic patients with HCC. Recurrence of HCC was detected in 28 of 50 patients who survived surgery (56%). Seven patients underwent LTX, the indications being tumour recurrence in 4 and liver failure in 3. Those patients transplanted for tumour recurrence, only one patient is alive 9 months later with no evidence of tumour recurrence; one died from carcinomatosis 5 months post-LTX and the other two died from cryptococcal meningitis and from upper digestive bleeding with dyseminated aspergillosis respectively and with severe recurrence of viral hepatitis C in both. Liver failure was the indication for LTX in one patient during the early postoperative LR period and is still alive 38 months post-LTX. Other two patients underwent LTX 47 and 5 months after LR, but both died from septicaemia in early and late postoperative periods, respectively. The actuarial survival rates at 1 and 4 years for patients who underwent LR and afterwards a LTX were 86% and 43% respectively. (Actuarial survival of patients with LR alone were 60% and 32%, respectively).
Conclusion: LTX may offer a second opportunity in selected cirrhotic patients after LR for HCC. LR plus LTX has an actuarial survival at 4 years of 43%.

### F014

**PARTIAL PORTA-CAVAL SHUNT AND LIVER FUNCTION**

G.Batignani, M. Zuckermann, R.Valanzano, F.Tonelli.

Dipartimento di Fisiopatologia Clinica-Unità di Chirurgia

Università degli Studi di Firenze

From April 1993 to October 1995 19 patients ( mean age 63.5 years; range 54-75) underwent a partial portocaval shunt with a ringed Polytetrafluoroethylene (P.T.F.E.) prosthesis ( 8 or 10 mm.). The mean follow up was of 10.3 months ( range 6-30 ).
17 of them were cirrhotics ( 6 were Child A, 5 Child B and 6 Child C) while 2 pts. had a chronic active hepatitis. Viral status was B positive in 5 pts., C pos. in 18 pts. while 2 pts. were alcoholics. 17 out of 19 pts. had documented previous variceal hemorrhages and 6 pts. had concomitant portal hypertensive gastropathy (P.H.G.). In 2 pts.the indication was a refractory ascites. We used an 8 mm H graft in ten patients and a 10 mm. in 9 patients. 6 patients were operated in emergency and in this situation we used a 10 mm. prosthesis in order to obtain a better decompression of the portal system.
Postoperative complications were: 1 neuropathy problably related to crioglobulinemia, 1 bleeding fron erosive gastritis, 2 bleeding duodenal ulcers and 1 duodenal perforation. In 1 pt. (5.3%) an HCC was showed a year after shunt procedure.
4 pts. died after surgery so the overall operative mortality has been 21% : 2 of these pts. underwent shunt procedure in an emergency setting and they were 3 Child C (50%) and 1 Child B (20%). Cumulative shunt patency in survived pts. was 100% and we have not observed neither episodes of rebleeding from oesophageal varices nor a graft thrombosis during follow up. 4 out 15 (26.6%) survived pts. had an episode of acute encephalopathy while only a patient (6.6%) developped a chronic encephalopathy. Our results show that partial portacaval shunt with a small diameter P.T.F.E. prosthesis (8 or 10 mm.) is an effective procedure for the treatment of variceal rebleeding with low rate of chronic encephalopathy. We conclude that this technique, which does not compromise liver transplantation, should be used in Child A pts. when sclerotherapy has failed, in presence of P.H.G., when the pt. does not comply with sclerotherapy or when he or she lives in a non-urban area. Pts. with poor liver function showed high morbidity and mortality so they are probably better served with T.I.P.S. procedure. A clinical trial comparing costs, complications and results of variceal injection-ligation and partial portacaval shunt in Child A pts. might be indicated.

### F015

**RISK FACTORS FOR MORTALITY AFTER HEPATIC RESECTION**

W.O.Bechstein, N.Kling, O.Guckelberger, S.Jonas, P.Neuhaus
Dept. of Surgery, Virchow Clinic, Humboldt University, Berlin, Germany

In order to identify risk factors for mortality after elective hepatic resection a retrospective univariate analysis of risk factors was performed. Between 1988 and 1995 315 hepatic resections were performed in 161 males and 154 females (median age 58 yrs., range 14-90 yrs.). 187 Resections were performed for metastases, 64 for primary liver tumors, and 64 for benign lesions such as hemangioma or FNH. Resection methods included 121 anatomical lobectomies, 56 extended lobectomies and 138 so-called minor resections with removal of solitary of multiple segments or atypical subsegmentectomies.The 30-day mortality in the whole series was 3.8% (12/315). The following risk factors were significantly different between patients who died within 30 d and those that survived >30 days: bilirubin (Bilir), AST, alkaline phosphatase (AP), albumin (Alb), pseudo-cholinesterase (pCHE), prothrombin acc. to Quick, and duration of surgery (OP-time).Results are shown in table 1, all values expressed as median, statistical analysis was carried out with Mann-Whitney U-test.

| Survival | Bilir. mg/dl | AST u/L | AP u/L | Alb g/dl | pCHE u/L | Quick % | OP time (min) |
|---|---|---|---|---|---|---|---|
| ≤30 d | 1.3 | 24 | 298 | 3.6 | 1.8 | 91 | 240 |
| >30 d | .6 | 13 | 141 | 4.2 | 4.8 | 100 | 200 |
| p-value | <.05 | <.001 | <.001 | <.001 | <.01 | <.05 | <0.05 |

Age (62,5 vs. 58 yrs), median number of lesions (1 in each group), median tumor size (68 vs. 50 mm), duration of hilar occlusion (25 vs.26 min), and median number of units red packed cells (1 vs. 0) did not differ significantly between both groups. There were significantly more early deaths in patients with cirrhosis (5/42 ) compared to those without cirrhosis (7/273) and in those with primary liver tumors (6/64) compared to those with metastases (6/187) or those with benign lesions (0/64) (p <.05, Chi-square). Patients sex, extend of the resection, and lobar involvement had no influence on early outcome. The significantly different preoperative biochemistry values in patients who died within 30 days following elective hepatic resection underline the importance of preoperative patient selection for avoiding early mortality.

### F016

**AUXILIARY LIVER TRANSPLANTATION FOR FULMINANT LIVER FAILURE : LIMITS OF AN ATTRACTIVE CONCEPT.**

J. Belghiti, F. Zinzindohoue, F. Durand*, R. Noun, A. Sauvanet, J. Bernuau*.
Departments of Digestive Surgery & *Hepatology, Hospital Beaujon, University Paris VII, Clichy, France.

Auxiliary liver transplantation (ALT) theoretically bridges the period of acute liver failure until the native liver (NL) recovers and immunosuppression can be discontinued. However, this attractive concept is burdened by technical problems and by the selection of candidates. We report our experience of ALT with special references to early and long term graft function in a prospective study including all patients who underwent emergency liver transplantation for acute liver failure from April 1993 to October 1995.
Patients: Thirty patients aged from 16 to 62 years with acute liver failure were candidates for emergency liver transplantation according to Clichy criteria. Causal disease was drug toxicity (n=10) including paracetamol in 3; hepatitis B (n=6); hepatitis A (n=2) and other (n=12). We decided to perform OLT in 18 because of age>60 years (n=3), pre-existing chronic liver disease (n=4), haemodynamic instability (n=4), poor liver graft (n=2) and poor neurological status with immediate risk of cerebral herniation (n=5). Seven patients died postoperatively including 5 after ALT; in the latter group mortality was due to vascular thrombosis (n=3) graft compression (n=1) and sepsis (n=1). With a follow up ranging from 3 to 31 months among the 7 surviving patients, graft was removed in 2 respectively after 1 and 7 months, immunosuppression was stoped in 2 respectively after 9 and 27 months. Liver biopsy demonstrated the presence of mild fibrosis in 3 respectively after 6 and 9 months.
Conclusion: After auxiliary liver transplantation for fulminant hepatitis, there is no predictive value of the extent nor the delay of sufficient regeneration of the native liver. The higher operative risk associated with ALT suggests that this procedure should: (a) not be indicated earlier than standard OLT; (b) be restricted to patients < 50 years without haemodynamic instability and (c) be performed using good quality ABO compatible graft.

### F017

**PREDICTIVE FACTORS OF ACUTE RENAL FAILURE (ARF) IN LIVER TRANSPLANT (LT).**

Bilbao I, Hidalgo E, Charco R, Balsells J, Lázaro JL, Murio E, F.Cuadrado T, MARGARIT C.
Liver Transplantation Unit. Hospital Universitario Vall d'Hebrón. Paseo Vall d'Hebrón s/n 08035 Barcelona. SPAIN.

PURPOSE : To Analyse the risk factors for ARF requiring renal depuration in early postoperative LT.
MATERIAL : Between Oct-88 and Dec-94, 172 liver transplants were performed in 158 patients with end-stage liver disease. The median age was 51 years (r:16-66). Child's classification prior to LT was: A(10%); B(37%); and C(53%). Ninety percent were UNOS I and II status. Renal insufficiency prior to LT attained to 18% of patients.
Donor and recipient factors studied in relationship to ARF were: preoperative parameters (age, sex, indication to LT, Child-Pugh score, UNOS status, laboratory data, renal function prior to LT), intraoperative parameters (lenġth of surgery, type and length of anhepatic phase, preservation time, periods of hypotension, blood product requirements, diuretic doses, balance, etc), and postoperative parameters (prymary liver disfunction, blood products transfused etc).
RESULTS : More than 50% (88 patients) developed ARF while in ICU: 27% mild (SCr 1,5-3); 7% moderate (SCr >3); and 17% severe requiring renal depuration (30 patients). Renal depuration was required: immediate postoperative < 5 days in 19 ; between 5-14 days in 5 ; and late postoperative > 15 days in 6 . Type of depuration employed was : hemodyalisis in 9 patients; ultrafiltration in 19; and continous arterio-venous ultrafiltration in 2. Overall postoperative mortality in depuration group was 50% in contrast to 13,4% in the rest of patients.
Univariate analysis of donor and recipient factors showed that: urgent retransplantation, advanced Child-Pugh score, renal function prior to LT, preservation time, blood product requirements, and primary liver disfunction, were the variables observed statistically significant in relationship to ARF. However, multivariate analysis revealed that among 38 variables investigated in our serie, only two had independent prognostic value : preoperative SCr > 1.5 mg/dl and graft dysfunction grades III and IV.
CONCLUSION : Early ARF is a common and severe complication in LT, with high morbi-mortality, that can be predicted particularly in relation to some well-known factors, mentioned aboved.

### F018

**RISK FACTORS FOR SEVERE ISCHEMIC LESION (IL) AFTER LIVER TRANSPLANT (LT).**

I.Bilbao, E.Hidalgo, JL.Lázaro, R.Charco, J.Balsells, E.Murio, A.Edo , C.MARGARIT.
Unidad de Trasplante Hepático. Hospital Vall d'Hebrón. Barcelona, Spain.

To meet the demand for grafts to transplant patients with end-stage liver diseases or acute liver failure, it is necessary to accept the so called non-optimal donors, which in adittion to other negative factors can lead to important graft dysfunction or even primary non-function. The aim of this study is to analyse the risk factors that could influence in the appearence of IL, originated either in the donor (prepreservation injury), or during hypothermic storage (preservation injury), or during reperfusion/reimplantation period.
MATERIAL AND METHODS : From October 1988 to December 1994 172 OLT were performed in 158 patients. The mean age was 51 years. Sex distribution was 65% males and 35% females.
Ischemic hepàtic lesion was classified regarding liver function test during the first 4 days : Mild (GPT < 1000, and prothrombin time (PT) > 60%); Moderate (GPT 1000-5000 and PT 30%-60%; and Severe (GPT > 5000 and PT < 30%). All the events ocurring during the immediate postoperative period during their stay in ICU were stored in a database.
RESULTS : Out of 172 transplants : 109 (63%) had a mild IL ; 45 (26%) had a moderate IL ; and 18 (11%) had severe IL. In the severe IL , 4 presented PNF and were retransplanted or died.
Univariate and multivariate analysis revealed that: advanced Child-C hepatopathy and older recipient age, ICU donor stay longer than 5 days, and positive crossmatch were associated with appearence of severe graft dysfunction. Postoperative mortality and 1-year graft survival for severe IL were 50% and 33% respectively, with differences statistically significant compared to the rest of transplants.
Patients with severe ischemic lesion showed higher morbidity in terms of greater need of dialysis, rate of severe infections, need of respirator, and ICU stay.
CONCLUSION : Incidence of severe ischemic injury was 10.4% with 2.3% of PNF. Risk factors for severe ischemic lesion were recipient age, Child-C, and donor ICU stay longer than 5 days. As increased morbidity and decreased graft survival were found in this group of patients, association of several risk factors should be thoroughly outweight in order to perform a LT.

### F019

ENDOSCOPIC SCLEROTHERAPY (ES) OF GASTRIC VARICES (GV)
OUR EXPERIENCE.

Bisello M.,Gerunda GE, Zangrandi F.; Neri D.; Merenda R.; Meduri F.; Barbazza F.;Da Giau G.;Bruttocao A., Ciardo L.; Scopece A., Girardi R., Renon L., Maffei Faccioli A.
First Department of General Surgery University Padua.

GV bleeding presents high risck of mortality. Emergency surgery has high mortality. ES og GV using Bucrylate (B) material, may be a valid alternative particularly in emergency. The aim of this paper is to presents our experience in Polidocanol Vs Bucrylate ES of GV bleeding.
MATERIALS: 52 Pts ( mean age 48+/-16 - range 43-68 ) were trataed in our Istitution. 50% had alcoholic cirrhosis, Acc.to Child pug risk 6% were A,61% B, 33% C. Acc.to NIEC Criteria 61% were of type I, 35% of Type II,4% of type III (type I+II = 17% ). 88% and 12% were respectively emergency and electively trated. 16pts were treated with Polydocanol + Saline ES ( respectively 30+/-6 range 38-465 and 40+/-7 ml range 36-48 ) . 36 Pts were treated with B -ES (with or without LIPIODOL 1:1 ) with a mean of 2.9+/-0.9 ml. RESULTS:

| | Pol.±fSal. | Bucrylate | Overall | $\chi 2$ |
|---|---|---|---|---|
| PATIENTS | 16 | 36(30 in urg.) | 52(46in urg.) | p<0.01 |
| STOP HEMORR. (-24h) | 9 (56%) | 30 (100%) | 39 (85%) | p<0.01 |
| REC. HEMOR. (<7gg) | 12 (75%) | 3 (10%) | 15 (33%) | p<0.01 |
| REC. HEMOR. (<30gg) | 6 (38%) | 2 (7%) | 8 (17%) | p<0.01 |
| EMERGENCY SURG. | 4 (25%) | 0 | 4(9%) | p<0.01 |
| EARLY MORT. (<30gg) | 7 (44%) | 2 (7%) | 9 (20%) | p<0.01 |
| ELECTIVE SURGERY | 1 (6%) | 7 (24%) | 8 (18%) | p<0.02 |

Conclusion: B-ES represents a real efficacious method to resolve the mergency of GV bleeding. The complessity of the etechnic and the risks for instruments and operators, needs a very experinced team.

### F020

LIVER TRANSPLANTATION FOR HEPATOCELLULAR CARCINOMA ON CIRRHOSIS : PROGNOSTIC IMPACT OF AN ADAPTED PATIENT SELECTION

H Bismuth, R Adam .
Hepato-Biliary Surgery and Liver Transplant Research Center, Paul Brousse Hospital, Villejuif, France.

Hepatocellular carcinoma (HCC) is an established but still debated indication of liver transplantation (LT). The high risk of recurrence and five year- survival rates significantly lower (0-50% in different series) than those of benign diseases have questionned the place of LT for HCC in the current period of organ shortage. We report in this study the consequences of a new selection of patients adapted from prognostic indicators established in the first phase of a same series. From November 1985 to March 1994, 109 patients with cirrhosis were transplanted for HCC. Of these 109 patients, only 95 patients with HCC diagnosed before LT were included in the study. The presence of extrahepatic deposits on pretransplant staging or peroperative exploration was considered as a contraindication to LT. In the first period of our experience (November 85-December 91), the selection criteria only included the absence of any extrahepatic tumour (60 patients). After assessment of prognostic factors in this first period (mainly tumor size > 30 mm, number of nodules > 3 and presence of a portal thrombosis), we proceeded to a more restrictive selection of those patients at very high risk of recurrence. Results in terms of patient selection and 3 year-survival were as follows :

| | 1st period (85-91) n=60 | 2sd period (92-94) n=35 | p |
|---|---|---|---|
| No nodules >3 | 24 (40%) | 6 (17%) | 0.03 |
| Size> 30 mm | 29 (48%) | 12 (34%) | 0.01 |
| Portal thrombosis | 6 (10%) | 1 (3%) | NS |
| No and Size<30 mm<3nod | 21 (35%) | 22 (63%) | 0.04 |
| >30 mm >3 nod. | 14 (23%) | 5 (14%) | |
| Recurrence | 20 (33%) | 4 (11%) | 0.01 |
| 3 yr-Surv.(Overall- Disease free) | 55%-49% | 76.5%-70% | NS-0.07 |

Conclusion: An adapted selection of pàtients with HCC for LT allows a significant decrease of the recurrence rate and a trend to improved survival. This warrants the indication of LT for HCC even in the current period of organ shortage.

F021

AUXILIARY PARTIAL ORTHOTOPIC LIVER TRANSPLANTATION (APOLT) IN FULMINANT HEPATITIS (FH).

H. Bismuth, D. Azoulay, D. Samuel, Ph. Ichai, F. Saliba, R. Adam, D.Castaing
Hepatobiliary Surgery and Liver Transplant Center, Paul Brousse Hospital. Villejuif, France

APOLT has been proposed in the treatment of FH to provide temporary hepatic support until the native liver regeneration. We report herein our experience with APOLT for FH in 5 patients during the past 12 months. During the same period X patients were treated at our center for FH needing liver transplantation. Conventional orthotopic liver transplantation (OLT) was chosen in X most severe cases to shorten as much as possible the waiting time for a liver graft. APOLT was chosen as a potential bridge in 2 cases because the graft was ABO incompatible, in 1 case because of a too small graft from a living related donor, and in 3 cases because the potential for native liver regeneration was judged reasonnable in stable patients. The main caracteristics of the 5 patients are sumarized in table 1.

**In conclusion,** Our experience with APOLT confirms the technical feasibility of this approach in FH. ABO incompatible graft may be a good indication of APOLT in FH. If, due to rejection or biliary complications, the graft should be removed, there is a chance of regeneration of the native liver allowing to avoid retransplantation.

| patient n/sex/age | cause of FH | liver graft* | native liver* | survival after APOLT | Comments |
|---|---|---|---|---|---|
| 1/M/19 | HBV | 2-4 | 4-8 | A,19M | native liver regeneration, liver graft removed at 10M |
| 2/M13 | unknown | 5-8 | 1-4 | A,13M | native liver atrophy, chronic rejection both removed and reOLT at 4M |
| 3/F/58 | drug induced | 2-4 | 4-8 | D,3M, sepsis | Autopsy: no native liver regeneration and normal graft |
| 4/M/15 | Reye | 2-3 LRLT | 4-8 | D, 10 brain death | autopsy d11, native liver regeneration, mild graft rejection |
| 5/F/40 | HAV | 5-8 | 1-4 | A, 1M | biopsy d15, partial native liver regeneration, acute graft rejection |

* according to Couinaud : A = alive, D, dead, d= day, m= month, M=male, f=female, OLT= conventiaonal orthotopic liver transplantation

F022

SECONDARY ALTERATIONS TO ISCHEMIA / REPERFUSION INJURIES AFTER PARTIAL HEPATIC ISCHEMIA IN WISTAR RATS USING WEB2086

I.F.S.F. Boin, O. Castro e Silva Jr., L.S. Leonardi

Unit of Liver Transplantation / UNICAMP
Hepatic Studies Laboratory / FMURP - SP

The objective of this work was to verify and analyse, in Wistar rats, the alterations caused by 90-minutes partial hepatic ischemia, followed by immediate, 15 and 60 minutes reperfusion, using a PAF-antagonist (WEB2086). A total of 48 male Wistar rats were used, with mean weight of 310g, previously submitted to a 6-hour fast with water ad libitum. Twelve rats were separated for hemodynamic and electrolytes analysis of: MAP, CVP, pH, $pCO_2$, $pO_2$, BE, $HCO_3$, Na, K, $Ca^{++}$, GLY, Hb, htc. Thirty-six rats were separated for analysis of mitochondrial function, RCR, liver function tests (AST, ALT, LDH, $LDH_5$) and GLY. In the first part of the study the twelve rats were divided in two sub-groups of 6 rats each. The first sub-group was submitted to partial hepatic ischemia of the median and left lobes; the second sub-group received a specific PAF-antagonist (WEB2086) 5 minutes before the ischemia. In the second part of the study the rats were also divided in two sub-groups, one sub-group submitted to partial hepatic ischemia, the other receiving WEB 2086 5 minutes before (20mg / kg). The rats were submitted to partial hepatic ischemia for 90 minutes and studied in reperfusion times of zero, 15 and 60 minutes (R0, R15, R60).The results of the several groups were analysed with ANOVA and Friedman test. We observed statistically significant difference in the time R0 of the following : MAP, CVP, Na, Hb, htc, ADPO and OPR. In the time R15: E III, MAP, $HCO_3$, BE. In the time R60: CVP, BE, $HCO_3$,GLY, E IV and LDH. Although, the use of WEB 2086 (PAF-antagonist) had inhibited the hypotension proprierties of PAF that occurred during the reperfusion, we did not observed improvement in that liver function and the mitochondrial function was maintained.

F023

EFFICACY OF TRANSARTERIAL CHEMOEMBOLIZATION (TACE) ASSOCIATED TO PERCUTANEOUS ETHANOL INJECTION (PEI) IN THE TREATMENT OF SMALL HEPATOCELLULAR CARCINOMA (HCC).

L. Bolondi, S. Sofia, N. Venturoli, A. Casali, S. Siringo, T. Livraghi, E. Caturelli, G.L. Rapaccini, C. Bartolozzi, L. Gandolfi and the multicentric study group of the Italian Association for the Study of Liver Disease (AISF)

Non controlled studies have reported better results for the association of TACE with PEI than for PEI alone in the treatment unifocal HCC.

Patients and methods. We report here the preliminary results of a prospective multicentric controlled study comparing these two treatment strategies in 150 cases of small (<5 cm) unifocal HCC in liver cirrhosis in Child Pugh class A (n=89) and B (n=61) in whom liver resection was not feasible (age >65 years; Child B class; esophageal varices at risk; location of HCC in central segments). Patients were enrolled in 12 different italian centers since February 1992 to May 1994 and 84 of them were randomly assigned to PEI and 66 to TACE + PEI. The two groups were matched for sex, age, Child-Pugh class, size of HCC and AFP level at entry. Diagnosis of HCC was established by echo-guided liver biopsy and unifocality at US examination was confirmed in all cases by contrast-enhanced CT. In the group TACE + PEI, TACE was performed prior to PEI. All patients were followed at four months intervals by US and AFP. PEI was repeated in case of recurrence. The mean follow up was 18 months in the group TACE + PEI and 19 months in the group PEI. The treatment outcome was evaluated as follows: 1) successful when the lesion remained stable or decreased over time; 2) local progression; 3) diffuse progression. Survival curves in the two groups were then assessed by the Kaplan-Meier method and compared by the Mantel-Cox method.

Results. A decrease of the AFP levels was observed in both groups of treatment at 12 months interval, but any significant difference was not found between the two groups. A non significant ($p<0.1>0.05$) trend towards a better outcome (80% stable disease vs 59%) at 16 months was found in the group TACE + PEI. The analysis of survival curves showed a non significant trend for an improved survival in the group TACE + PEI in comparison with PEI alone (estimated survival at 24 months = 65% vs 45%). The severity of liver cirrhosis was not worsened over time in the group TACE + PEI in comparison with the group PEI.

Conclusions. A follow up longer than 2 years is needed to demonstrate a possible better efficacy of TACE + PEI in comparison to PEI alone in the treatment of small unifocal HCC.

F024

**PARTIAL VS. TOTAL PORTACAVAL SHUNT: A PROSPECTIVE RANDOMIZED TRIAL.**

Capussotti L., V. Vergara, R. Polastri, H. Bouzari, M.M. Marucci.

Department of Surgery, Ospedale Mauriziano, Largo Turati 62 - Torino, Italy.

From January 1990 to December 1994, forty-six patients were considered for inclusion in a prospective randomized trial. 23 patients underwent to 10 mm portacaval H-graft (Group 1) without collateral ligation, 23 underwent to side-to-side portacaval shunt (Group 2) in order to evaluate: operative and long-term mortality and survival, hepatic score and hepatic encephalopathy (HE). Inclusion criteria consisted of: age< 70, documented hepatic cirrhosis, Child-Pugh's class A or B (only patients with score = 7), maintained hepatic portal flow, no ascites, bleeding from oesophageal and/or gastric varices requiring two or more blood Units. Group 1: 17/6 M/F, mean age 58 years (range: 27-67). 12 patients had alcoholic cirrhosis. Mean±SD of haemorrhagic episodes was 2.6±1.3. According to Child-Pugh's classification: 11 patients were class A, 12 class B. Group 2: 15/8 M/F, mean age 60 years (range:34-68). 13 patients had alcoholic cirrhosis. Mean of haemorragic episodes was 2.3±0.7. Ten patients were Child-Pugh A and 13 were B. Long term survival was calculated by Kaplan-Meier method. Differences between the groups with respect to preoperative parameters were examined usign the $\chi2$ test and Student's T-test where appropriate. No operative mortality (at 60 days) occurred in both groups. One patient of each group had an early postoperative variceal haemorrage episode. No patient of group 1 and 2 patients of group 2 had one haemorrage episodes at 12 and 17 months after operation (p=NS). Long-term HE was significantly lower ($p < .05$) in group 1 (13%) vs group 2 (39%). No patient of group 1 and 2 patients of group 2 had severe chronic HE. Long-term evaluation of hepatic score was 0.59±0.11 in group 1 and 0.67±0.11 in group 2 ($p < .02$). Actuarial survival rate at 1,3 and 5 years was 100%, 77% and 67% in group 1 and 86%, 63% and 52% in group 2 respectively (p = .04 Gehan's Wilcoxon Test). In conclusion: partial shunt, mantaining a good hepatic flow, significantly improves long-term survival and reduces postoperative encephalopathy and postoperative hepatic deterioration vs. total shunt.

### F025

**Advantages and disadvantages of inferior vena cava preservation in ortothopic liver transplantation**

D. Casanova, M G Fleitas, E Martino, L Herrera, F Hernanz, JM Rabanal, G Solares
Department of Surgery, University Hospital Valdecilla, University of Cantabria, Santander, Spain

Inferior vana cava preservation (IVCP) has been proposed an alternative procedure in ortothopic liver transplantation (OLT). This techniques provides a continuous caval flow to the heart, minimizing the hemodynamic alterations and increasing the renal perfusion. **Aim:** The objective is prospective study of ortothopic liver transplantation using IVCP. **Patients and methods:** Between November 1990 and November 1995, 130 OLT were performed in 118 adult patients (12 were retransplants). Donors and recipients were matched for size (weight and height) and ABO blood groups. During the surgery, in all cases, the liver was removed preserving the inferior cava vena. Cross clamping the IVC, was placed laterally preserving blood flow throughout the anhepatic phase. **Results:** We have performed the hepatectomy with IVCP during the consecutives 130 OLT. Postoperative vascular complications (thrombosis of suprahepatic veins) related to the technique occurred in three patients (2, 3%), and need a retransplantation to solve the complications. The advantages of this technique include the absence of retrocaval disection, preservation of caval flow during the anhepatic phase, and allow to avoid the anastomosis of inferior cava vein. Intraoperative hemadynamic data suggests the stability during all the procedure:

| | Before lateral clamping | During lateral clamping | After revascularitation |
|---|---|---|---|
| Mean arterial pressure | 88±14 | 89±13 | 82±14 |
| Inferior vana cava pressure | 19±4 | ·20±6 | 19±6 |
| Renal perfusion pressure | 68±15 | 71±17 | 67±15 |
| Cardiac Index | 4, 7±1, 5 | 4, 4±1, 4 | 6, 4±2,4 |
| Systematic vascular resistance | 525±348 | 512±280 | 319±211 |

**Conclusions:** OLT with IVCP, appears to be feasible in all liver transplants without important surgical complications, and permit a hemodynamic stability during the anhepatic phase.

### F026

IMPROVED DONOR LIVERS FOR TRANSPLANTATION: ASSESSMENT BY MAGNETIC RESONANCE SPECTROSCOPY.

K. Changani, B.Fuller, #D. Bryant, #J. Bell, #S. Taylor-Robinson, #M. Ala-Korpela, D. Moore, B. Davidson.
Dept of Surgery, Royal Free Hospital & School of Medicine; #NMR Unit, Hammersmith Hospital, London, UK.

Regeneration of hepatic ATP is one of the single most important pre-requisites for liver function following transplantation This study has used clinical liver harvesting and storage techniques in the pig to evaluate the benefits of a prostacyclin derivative and adenosine following a period of hypothermic reperfusion (HtR). The non-invasive technique of $^{31}P$ magnetic resonance spectroscopy (MRS) was employed to monitor ATP regeneration in real time without the need of biopsy. Land Race Large White pigs (n=5, 30kg) were anaethetised and intubated. Midline and lateral abdominal incisions were made to expose the 4 anatomical lobes of the liver. Following hepatic artery occlusion, the hepatic portal vein was cannulated and the liver perfused with 1 litre of ice-cold citrate plus 1 litre of ice-cold modified University of Wisconsin solution (UW). Two other groups of pigs (n=5 per group) had either adenosine (1.34g/l) or a prostacyclin derivative [ZK 36374] at $10^{-8}$M added to both solutions. The liver was positioned in the bore of a 1.5 Tesla Picker Prototype MRS machine and phosphorus spectra collected at 26 MHz. The liver was reperfused with the respective oxygenated buffer solution and the regeneration of ATP monitored following the acquisition of 2 min time resolved spectra. Concurrently, changes in inorganic phosphate, phosphomonoesters and phosphodiesters could also be resolved in real time. Spectra were analysed using a dedicated program designed specifically from the biochemical composition profiles of resonant peaks. Initial rates of ATP regeneration in the UW group was $9.7 \times 10^{-3}s^{-1}$ whereas groups with added adenosine (Ad) or the prostacylin derivative (PD) had rates of $9.5 \times 10^{-3}s^{-1}$ and $14.2 \times 10^{-3}s^{-1}$, respectively. The maximal attained amounts of ATP with respect to total phosphate in these same groups were 4.49 ± 0.52% (UW), 5.82 ± 0.27% (UW + Ad) and 6.79 ± 0.40% (UW + PD). These changes represent an increase from the UW solution of 30% with added adenosine ($p<0.05$) and 51% with added prostacyclin ($p<0.02$). This study suggests that improvements can be made in ATP regeneration using prostacyclin derivatives and adenosine to buffer the adenylate pool.

### F027

**MAJOR LIVER RESECTIONS WITHOUT PREOPERATIVE BILIARY DRAINAGE IN PATIENTS WITH OBSTRUCTIVE JAUNDICE**

D. Cherqui, V. Rodriguez, R. Alon, N. Rotman, M. Julien, P.L. Fagniez.
Department of Surgery, Hôpital Henri Mondor Créteil France

It has been suggested that obstructive jaundice increases the risk of liver resections because of reduced regeneration capacities, increased operative bleeding and general effects associated with jaundice. Therefore, preoperative percutaneous biliary drainage is often recommended. However, the risks of sepsis and tumor seeding along drain tracts led us to avoid preoperative biliary drainage in these patients.
From 1989 to 1995, 85 major liver resections (≥ 3 segments) were performed. 13 patients (15%) had obstructive jaundice and underwent resection without preoperative biliary drainage. Mean serum bilirubin was 215±108 µmol/L (60-439). They included 4 hilar cholangiocarcinomas, 4 gallbladder carcinomas, 3 intrahepatic cholangiocarcinomas extended to the hilus and 2 tumors with thrombus extension in the biliary tract. These cases were compared with 72 resections in patients without jaundice.

| mean values | jaundice + (13) | jaundice - (72) | p |
|---|---|---|---|
| extended resection | 13 (100%) | 11 (15%) | < 0.01 |
| vascular exclusion | 7 (54%) | 16 (22%) | < 0.05 |
| clamping time | 39 min | 38 min | |
| transfusions | 5 units | 2.6 units | 0.08 |
| operative time | 5.9 h | 3.6 h | < 0.01 |
| AST peak/minimal PT | 543 / 54% | 311 / 53% | NS |
| biliary fistula | 38 % | 6 % | < 0,01 |
| mortality | 2 (15%) | 1 (2%) | NS |
| hospital stay | 28 days | 15 days | < 0,01 |

The two deaths were due sepsis and myocardal infarct and occurred in one patient with the highest serum bilirubin concentration and hypoalbuminemia and in one patient with renal failure.
These results suggest that most patients with obstructive jaundice can undergo major liver resection without preoperative biliary drainage. A long and complex procedure is necessary and the incidence biliary fistula is high. Increased operative bleeding was observed but tolerance to ischemia and regeneration were not affected by jaundice in this series. Preoperative drainage may be indicated in selected cases (bilirubin > 300, prolonged jaundice, hypoalbuminemia, renal failure).

### F028

EFFECT OF INOTROPES AND STEATOSIS ON TISSUE PERFUSION OF HUMAN LIVER GRAFTS.

V. Chidambaram, A.M. Seifalian,' A. Giudiceandrea, N.S.Sreekumar, M. Farouk, K. Rolles, B.R. Davidson.
University Department of Surgery, Royal Free Hospital & School of Medicine, London U.K.

We studied the hepatic surface perfusion during organ retrieval in humans donors using Laser Doppler flowmeter (LDF) to assess the microcirculatory alteration caused by inotropes used for cardiovascular support of donors and fatty infilteration (steatosis) of the liver. **Method**: Using a Multimoor LDF, we measured the surface perfusion as flux units in 17 liver donors. Nine donors were on inotropic support (dobutamine, high dose dopamine, epinephrine or norepinephrine for a minimum period of 2 hours) and 5 livers were macroscopically fatty. The surface perfusion was recorded continuously from 2 sites on each lobe at the beginning of organ retrieval (Pre) and after mobilisation - isolation of its vessels (Post). The data, discussed as mean ± SD was analysed using unpaired students t test. **Results**: Mobilisation of the liver caused no alteration to surface perfusion ($p>0.05$). The donors receiving inotropes (n=9) had lower surface perfusion 150 ± 49 flux units than those (n=8) not receiving it 302 ± 92 flux units ($p=0.002$). Macroscopically steatotic livers (n=5) had a diminished perfusion 138 ± 103 flux units compared with normal (n=12) - 256 ± 51 flux units ($p=0.007$). **Conclusion**: Inotropes and steatosis diminish hepatic tissuegraft perfusionLDF is an useful tool for assessment of microcirculation during organ retrieval.

F029

### PROGNOSTIC SIGNIFICANCE OF p53 PROTEIN IN HEPATOCELLULAR CARCINOMA PATIENTS

Dong-Wook Choi, M.D., Gui-Tark Hong, M.D., Jong-Inn Lee, M.D., Nan-Mo Moon, M.D., Sang-Min Lee, M.D., Seung-Sook Lee, M.D.*
Department of Surgery and Pathology*; Korea Cancer Center Hospital, Seoul, Korea

Hepatocellular carcinoma(HCC) is the one of most frequent cancers in Korea, and the mortality rate from HCC is highest in this country all over the world, probably due to high hepatitis B virus infection rate and extremely poor prognosis. Established prognostic factors in HCC are presence of venous invasion, multiplicity, curative resection, but we do nok know all about prognosis in curatively resected cases. So, we studied p53 protein expression in Korea HCC patients, which is independent prognostic factor in breast cancer and lung cancer. We performed immunohistochemistry using Pab 1801, which is monoclonal antibody for p53 protein in 39 HCC patients, who underwent curative liver resection in Korea Cancer Center Hospital. Positive expression rate of p53 protein was 2.6%. p53 protein expression seemed to be higher in HCC patients with poor prognostic factors such as venous invasion, encapsulation, and multiplicity, but they did not reach the stastistical significance. And slightly better survival rates were shown in p53 negative protein group, but no significant difference was detected(p=0.593). In the other hand, presence of venous invasion, multiplicity and no capsule formation were related with bad prognosis statistically(p<0.05) In conclusion, we could not detect the prognostic significance of p53 protein expression in curatively resected HCC patients, but we think that more extensive study will be needed in more patients using diverse antibodies to mutant p53 protein.

F030

### SYSTEMIC CHEMOTHERAPY IN METASTATIC COLORECTAL CANCER

Ciferri E., Municinò O., Gazzaniga G.M.
1st Surgery Dept. - S. Martino Hosp. - Genoa - Italy

On the basis of recent demonstrations in vitro of two possible mechanisms of action and of induced resistance depending on the dosage and schedule of FUra administration, we submitted, in collaboration with the 1st Dept. of Medical Oncology IST of Genoa, 14 patients with metastatic colorectal cancer liver disease, considered unresectable, without other sign of recurrence, to chemotherapy, from August 1993 to Oct. 1995. According to this rationale we began a phase II trial of schedule-oriented biochemical modulation of FUra bolus by MTX and ß Interferon, and FUra continous infusion by Leucovorin. In particular, the treatment schedule provided a hybrid regimen of two biweekly administrations of 600 mg/sqm of FUra bolus, which had to be preceded the day before by 200 mg/sqm of methotrexate, and had to be followed, the same day and the next day, by two administrations of 3 x $10^6$ ß-Interferon i.m.; after an interval of two weeks, the cycle arried on with three weeks of continuous infusion of 200 mg/sqm per day of FUra, which was preceded every first day of the week by an administration of 20mg/sqm of Leucovorin bolus. The entire cycle was repeated after a week of rest, having first evaluated the lesions through US/CT scans and plotted the percent change of total measured tumour mass and dosed tumor markers. All the 14 patients, with no prechemotherapy, had the primary colorectal tumor mass resected for necessity and their livers were considered unresectable for the characteristics of the hepatic metastases: their number, dimensions, contiguity/continuity with important vascular structures didn't allow a radical operation. 13 patients (average age 63) have completed at least one cycle of the treatment and have been reevaluated, while one patient has just been included in the study; we have obtained two complete responses, after six months of chemotherapy, and, at the moment, also nine partial responses (75% of all); two patients died after 8 months because of the advancement of their illness. We have had one death due to toxicity after the first administration of FUra, MTX and Interferon in the first cycle, and two cases of III grade toxicity (diarrhoea and mucositis). The two patients that had a complete response to the chemotherapy were operated. two hepatic metastasis, in each one, that had reached dimensions of 2 cm in diameter, were resected, after a previous US controll.

F031

### LIVER TRANSPLANTATION MEDIATED TRANSFER OF IMMUNITY: ACCELERATED REJECTION OF A SKIN GRAFT AFTER LIVER TRANSPLANTATION FROM A SENSITIZED DONOR

Dahmen U, Tanigawa T, Rogiers X, *Lindkaer-Jensen S, Broelsch CE
Dept. of General Surgery, University Hospital Hamburg, Hamburg, Germany, *Dept. of Surgery L, University Hospital Aarhus, Aarhus, Denmark

Microchimerism after organ transplantation has been demonstrated repeatedly in clinical and experimental transplantation. Little is known about the functional relevance of the transferred immune cells. This study analyses the immunological activity of the chimeric donor cells in the recipient.

Male Lewis rats were either transplanted with a liver from a previously ACI-skin-sensitized BN-donor or a nontreated BN-rat and tested 2 weeks after liver transplantation with an ACI-test-skin graft for the transfer of this sensitization. Recipient sensitization before liver transplantation was included as a control.

Transplantation of a liver graft from previously skin sensitized BN-liver donors to Lew-recipients led to accelerated rejection of ACI-test skin grafts (median of 10 days (n=11) compared to median of 13 days in the control group (n=6); p-value (Mann-Whitney U test) = 0.004729); indicating a transfer of donor sensitization to the recipient. Similar results were achieved after recipient sensitization (median rejection time of test skin graft 9.5 days, (n=2)). BN-skin grafts used as marker for the tolerogenicity of the BN-liver graft were prolonged to the same extent, including permanent acceptance, in all three groups. Preliminary experiments using ACI-hearts as test grafts are pointing in the same direction.

This is the first systematic approach to demonstrate donor specific immune functions in a liver graft recipient, most likely explained by transfer of donor derived lymphocytes. In addition the recipient specific development of liver graft induced tolerance was not impaired; leading to observation of both, donor and recipient specific immune properties in the liver graft recipient, thus pointing towards merging of donor and recipients immune system.

F032

### ANTI-FERRITIN MONOCLONAL ANTIBODIES FOR DIAGNOSIS OF HEPATOCELLULAR CARCINOMA IN CIRRHOTIC PATIENTS

P. De Nardi, *P. Magnani, *C. Songini, G. Ferrari, V. Di Carlo
Chirurgia Generale e *Medicina Nucleare - IRCCS H S. Raffaele - Università degli Studi di Milano

Antibodies directed against hepatocellular carcinoma (HCC) antigens have been used in animal models as well as in humans. However lack of specific antibodies and the high background level when radiolabelled antibodies are administered, have hampered a wide use of immunoscintigraphy. Recently a new method, employing biotinilated monoclonal antibodies (MoAb), not directly radiolabelled, and avidin has been used, for diagnosis of CEA producing tumours, with good results. Aim of this work is to evaluate the sensitivity of anti-Ferritin MoAb immunoscintigraphy with the avidin biotin system, in identifying HCC in cirrhotic patients.

Between January and December 1995, 9 patients with proved HCC were studied. After performing abdominal ultrasound, NMR(6 pts), Lipiodol Tc (6 pts) and biopsy, the patients were injected with 1 mg anti-Ferritin MoAb; 24-36 hours later 10 mg of avidin were administered and after further 24 hours In-111 labelled biotin was injected. Two hours after the radioactivity administration planar and tomographic imaging were obtained. Eight patients subsequently underwent surgery during which 10 lesions were diagnosed. The lesions ranged in size between 1.5 and 13 cm. Seven patients underwent surgical resection. Ultrasound, NMR and TC correctly diagnosed HCC in 8/9,6/8 and 6/6 cases respectively. Immunoscintigraphy identified 6/9 HCC; no false positive radioactivity localization was observed. The lower resolutive power of immunoscintigraphy was 1.5 cm.

Our results suggest that anti-Ferritin immunoscintigraphy is effective in localizing HCC nodes in cirrhotic patients.

### F033

### LIVER TRANSPLANTATION IN ANTI-HCV POSITIVE PATIENTS: DYNAMICS OF HCV-VIREMIA AND CLINICAL EVOLUTION

E.De Raffele, R.Bellusci, A.Manzin°, R.Miniero*, B.Santoni, E.Lucci, M.Clementi#, L.Solforosi°, F.Fruet^, G.Sprovieri*, A.Mazziotti, A.Cavallari. Clinica Chirurgica II, Università di Bologna; °Istituto di Microbiologia, Università di Ancona; *Laboratorio Centralizzato, Policlinico S. Orsola, Bologna; # Dipartimento di Scienze Biomediche, Università di Trieste; ^Servizio Trasfusionale, Policlinico S. Orsola, Bologna. ITALY.

**INTRODUCTION.** The hepatitis C virus (HCV) is one of the major etiological agents of end-stage liver diseases requiring liver transplantation (OLT). However, recent studies demonstrate the high rate of reinfection after transplantation in HCV carriers. The purpose of the study was to evaluate: 1) the evolution of post-transplant viraemia in patients anti-HCV positive pre-OLT; 2) the clinical behavior of transplanted patients reinfected with the HCV; 3) the relation between HCV viraemia and ALT levels. **PATIENTS AND METHODS.** Sixty-one patients transplanted between 1987 and 1994, who were anti-HCV positive after OLT, with a post-OLT follow-up >3 mos. entered the study. Twelve (19,7%) became anti-HCV positive after OLT and 49 (80,3%) were anti-HCV positive pre-OLT. Their follow-up is 50,4±30,7 mos. Fifteen (24,6%) were also HBsAg positive pre-OLT. Antibodies against HCV were assayed using an ELISA assay and a RIBA assay of 2nd and/or 3rd generation. For HCV-RNA detection, a PCR was employed. **RESULTS.** Ten patients were HBsAg positive after OLT. Among 51 HBsAg negative patients, persistent or intermittent elevations of ALT levels were >2xN in 37 and <2xN in 7; five patients always showed normal ALT levels (two other patients were excluded). Seriated post-OLT determiantions of the HCV-RNA were available in 51 patients out of 61 (6±4 determinations per patient): 5 (9,8%) were HCV-RNA negative (3 were HBsAg positive after OLT and 2 had a follow-up <5 mos.), while 46 (90,2%) resulted HCV-RNA positive at least once. In this group of patients, a total of 307 post-OLT determinations were available: 103 (33,7%) resulted HCV-RNA negative. In 16 patients, one or more determinations of the HCV-RNA resulted negative after demonstration of viremia. No correlation was evident between viraemia and ALT levels: the HCV genome was usually found also in patients with persistingly normal ALT values, while HCV-RNA fluctuations were observed in patients with persistent ALT alterations. **CONCLUSIONS.** In our experience, patients carrying the hepatitis C virus were at high risk of reinfection after OLT. The HCV-RNA reappearead early in the serum in most cases, although without any apparent correlation with ALT levels. Signs of hepatitis of the transplanted liver, although usually mild and subjectively asymptomatic, were frequent. In these patients, repeated PCR assays may be useful for confirmation of HCV active replication, especially in case of abnormal liver function tests and histology, when a differential diagnosis with rejection is required.

### F034

### TOWARDS GENE THERAPY FOR LIVER MALIGNANCIES: ISOLATED LIVER PERFUSION FOR LIVER-SPECIFIC DELIVERY OF SUICIDE GENES WITH RECOMBINANT ADENOVIRAL VECTORS

*W.K. de Roos, F.J. Fallaux, A.W.K.S. Marinelli, M.M van der Eb, I.H.M. Borel Rinkes, C.J.H. van der Velden, O.T. Terpstra, R.C. Hoeben. Dept. of Surgery and Medical Biochemistry, University Hospital Leiden, State University of Leiden. The Netherlands.*

**Introduction.** Hepatic suicide gene therapy may provide a new approach to treat irresectable hepatic malignancies, either primary or metastatic. This strategy involves the infection of tumor and liver cells with replication-defective adenoviral vectors carrying suicide genes. Subsequently, systemically administered cytotoxic substrates selectively destruct replicating tumorcells, infected with suicide genes, whereas infected liver parenchymal cells are unaffected because of their minimal proliferative activity. Systems have been developed to deliver genes *in-vivo* with replication-defective adenoviral vectors. A major concern in this approach is that suicide genes could theoretically enter non-target organs with a high mitotic activity and, in this way, cause their destruction. The present study has focused on the development of strategies to achieve a targeted and more efficient adenovirus-mediated gene delivery to the liver.
**Methods.** For this purpose, we analysed the efficiency and liver-specificity of gene transfer with recombinant adenoviruses administered *via* isolated liver-perfusion (ILP). ILP involves the complete vascular isolation of the liver. The *E-coli* LacZ gene and the firefly luciferase gene, carried in adenoviral vectors (Ad.RSV.ß-gal and Ad.CMV-Luc, respectively), were used as reporter genes. In Wistar rats, the recombinant adenoviral vectors were administered to the liver *via* either intraportal infusion (IPI) or ILP. *Ex-vivo* perfusion experiments with the Ad.RSV.ß-gal were, initially, performed to optimize conditions for hepatic gene transfer.
**Results.** Ex-vivo perfusion of rat livers with $2x10^9$ plaque forming units (pfu) for 10 minutes was sufficient to infect at least 40% of the liver cells. Similar efficiencies were obtained in liverlobes of Rhesus monkeys, illustrating the feasibility of this approach in primates. *In-vivo* gene transfer *via* IPI or ILP was performed with $2x10^9$ PFU of Ad.CMV-Luc. A significantly more efficient gene transfer (p=0,028) was found in the ILP group when compared to the IPI group (mean $5,2x10^6$ vs $1,1x10^6$ lights units/mg protein, respectively). Also, gene transfer *via* ILP proved more reproducible. Although detectable in both groups, extrahepatic luciferase activity was considerably reduced in the ILP group.
**Conclusions.** Our data demonstrate that perfusion of vascularly-isolated liver with adenoviral vectors can be used as a modality for an efficient, reproducible and highly specific delivery of suicide genes to the liver.

### F035

### SIMULTANEOUS LIVER AND COLORECTAL CARCINOMA SURGERY

E. de Santibañes, A. Aldet, F. Bonadeo, M. Benati, G. Ojea Quintana, M. Ciardullo, J. Pekolj, J. Matera, P. Argibay, and C. Vaccaro.
HPB Surgery Section, General Surgery Service, Hospital Italiano, Buenos Aires, ARGENTINA

Between May 1982 and September 1995, 311 liver resections were performed as a results of colorectal metastasis. In 55 patients (17,6%) liver surgery was performed simultaneous with colorectal resection. The purpose of this report is the retrospective study of morbi-mortality risk in simultaneous liver and colorectal surgery as well as long-term and free of disease survival. The average age of the group was 61,3 years (38-82); 36 were male and 19 female. 11 of the initial tumors were found in the right colon (20%), 22 in the left colon (40%) and 22 in the rectum (40%). 1 was classified as Duke's A (1.8%), 15 Duke's B (27,2%), 26 Duke's C1 (47.2), 13 Duke's C2 (23,6).Hepatic resections consisted of: right lobectomy 4 cases (7,1%); left lobectomy 2 (3,6%), central lobectomy 1 (1,8%); left lateral segments 7 (12,7%); 20 segmentectomy (36,3); multiple segments 10 (18,1%); right trisegmentectomy 1 (1,8%); wedge resections 10 (18,1%). Colonic procedures consisted of: right colectomy 13 (23.6%); left colectomy 4 (7,2%); total colectomy 3 (5,4 %) subtotal colectomy 1 (1,8%); Anterior rectal resection procedures 19 (34,5%); low anterior resection 10 (18,1%); low anterior resection and hysterectomy 2 (3,6%); Miles procedures 3 (5,4%). Average surgical time was 4,3hs (2-6hs). Average ventilatory support time was 12.8hs (3-48); average intensive care stay was 49.4 (12-96hs). Mean hospital stay was 9 days (6-24). Postoperative complications were: intrabdominal abscesses 7 cases (12,7%); hepatic failure 3 (5,4%); sepsis 3 (5,4%); atelectasis 2 (3,6%); bile leakage 1 (1.85); pleural effusion 1 (1.8%) and anastomotic leakage 1 (1.8%); 34 patients received post-operative systemic chemotherapy; operative mortality rate was 0%. The follow up was possible in 80% of patients, with an average time of 25,6 (3-108). Free of disease survival was 53,5% at 1 year; 17% at 3 yr. and 7% at 5 yr. Actuarial survival rate was 87% at 1 yr.; 37 % at 3 yr. and 30% at 5 yr. Conclusion: Simultaneous surgery can be performed with similar mortality and morbility rate than sucesive colorectal and hepatic surgeries.

### F036

### LIVER TRANSPLANTATION FROM LIVING RELATED DONORS: IMPACT ON MORTALITY RATE IN WAITING LIST PATIENTS.

E. de Santibañes, M. Ciardullo, F. Mattera, J. Grondona, J. Pekolj, D. D'Agostino, J. Sívori.
HPB Surgery Section, General Surgery Service, Hospital Italiano, Buenos Aires, ARGENTINA

Scarcity of cadaveric donors, especially pediatric donors, has prompted the implementation of the living related donors liver transplantation program around the world.Despite the use of the reduced-size liver technique, mortality rate of patients ón waiting list was 39.2%, in 1992 and above 50% in recipients with weight less than 10 kg. Since then, the living related donors technique has been an option for patients with low weight and end-stage liver disease. 24 donors whose mean age was 30 years (13 fathers, 9 mothers, 1 uncle, 1 grandfather) were studied. Preoperative evaluation included liver volume via CT scan, hepatic vasculature and a complete medical and psychiatric evaluation. 54% of potential living related donors were refused due to liver conditions 6, psychological reasons 4, asthma 1, hepatitis c 1 and pregnancy 1. Mean age of the 11 donors was 29.3 years (22-37) and weight was 65.5 Kg (47-76). Postoperative complications included 1 case of duodenal ulcer and 1 case of wound infection. Hospital mean stay was 5.6 days. All donors are currently well and alive. Recipients included 10 children with biliary atresia and 1 child with ATT deficiency. The group's mean age was 2.2 years (0.9-10) and weight was 0.7Kg (7.4-26). Recipients urgency status was elective in 10 cases and of high urgency in 1 case (primary non-function of cadaveric liver). The technique employed was similar to the described by Broelsch and others. Postoperative complications in pediatric transplants were: Biliary (1 biliary leakage and 2 strictures) 3 cases (27%); infectious (2 peritonitis and 1 intrabdominal abscess) 3 cases (27%); portal thrombosis, 1 case (9%); pulmonary haematoma, 1 case (9%) and atelectasia 1 case (9%). The patient in high urgency status died of a stroke while in the other group 1 patient received a retransplant due to portal thrombosis and 1 patient died due to multiorganic failure.Currently, mortality rate of children on waiting list has decreased by 16.4%. Conclusion: The living related donor technique has allowed a decrease of mortality rate of children in waiting list.

### F037

### CELL PROLIFERATION-RELATED MARKERS IN PROGNOSIS OF RESECTED HEPATIC METASTASES.

R. Doci, A. Costa, P. Bignami, L. Mochen, L. Gennari.
Istituto Nazionale Tumori, Milano, Italy

Neoplastic tissue of 104 patients who had undergone curative hepatic resection for metastatic colorectal cancer was processed for determination of 3H-dT Labeling Index (LI); p53 and bcl-2 expression; and DNA ploidy. Clinical and pathological features were recorded: stage of primary, percentage of hepatic replacement (PHR), and site and number of metastases. The prognostic impact of different variables was evaluated by uni- and multivariate analysis.
At the first analysis the disease-free survival at 4 years was significantly related to LI, PHR and Dukes' stage of primary. At multivariate analysis only LI of metastases and Dukes' stage of primary retained their statistical significance. The disease-free survival of two groups of patients, one combining favorable (LI < 11%; Dukes A+B) and the other unfavorable factors (Dukes A+B, LI > 11%; Dukes C), was significantly different (30 vs 5%; p=0.002). LI and Dukes' stage can help in selecting patients with different prognoses.

### F038

### DIURESIS VERSUS THERAPEUTIC ABDOMINAL PARACENTESIS : EFFECTS ON ASCITIC FLUID INTER-LEUKIN-1, OPSONIC ACTIVITY AND COMPLEMENT

El-Said Hassan, Myriam Abou Seif Helmy
Hepatobiliary Unit, Faculty of Medicine, Alexandria University, Alexandria, Egypt.

In this randomized controlled study, the effects of diuretics on ascitic fluid interleukin-1 (IL-1β), opsonic activity (OA) and complement C3 concentrations were compared with those of therapeutic paracentesis in patients with mixed cirrhosis (i.e. mixed schistosomal hepatic fibrosis and cirrhosis) and tense ascites. Twenty four patients were randomly allocated to two groups: group A included twelve patients treated with spironolactone (200-400 mg/day) and group B included twelve patients treated with twice weekly 3-4 liters paracentesis for two weeks. Ascitic fluid samples from all patients were analyzed for total protein and albumin concentrations, C3 and IL-1β levels and OA at the beginning and 2 weeks after treatment. IL-1β, an immuno-regulatory cytokine that stimulates a variety of cells that function as effector of immune response towards antigens, was significantly decreased following, paracentesis while it remained almost stable among the diuretic treated patients. The ascitic fluid OA and C3 concentrations increased significantly in diuretic treated patients ($P<0.05$) while patients treated with paracentesis had significantly decreased C3 concentration, and their ascitic fluid OA remained stable. It can be concluded that diuretics, may have the potential advantage over therapeutic paracentesis of providing better protection from spontaneous bacterial peritonitis.

### F039

### SYSTEMIC, PORTAL AND RENAL HEMODYNAMICS IN PATIENTS WITH SCHISTOSOMAL HEPATIC FIBROSIS AND ASCITES: ROLE OF GLUCAGON

El-Said Hassan, Myriam Abou Seif Helmy,
Sanaa Ashour Mohamed
Hepatobiliary Unit, Faculty of Medicine, Alexandria University, Alexandria, Egypt.

In the present study, systemic, portal and renal hemodynamics were assessed using Doppler flowmetry in 24 patients with schistosomal hepatic fibrosis (SHF); 12 of them had ascites due to portal hypertension and 12 were non ascitic and in 12 healthy subjects. Also, renal function tests, fractional sodium excretion (FENa) and plasma level of glucagon, an endogenous vasodilator were determined in all patients and healthy subjects. Hemodynamic assessment of systemic and portal circulations showed a significant decrease in systemic vascular resistance and significant increases in cardiac index, and portal vein blood flow volume and congestion index in patients with SHF regardless of the presence of ascites as compared with healthy subjects. These circulatory changes were associated with significant increases in plasma glucagon levels in the ascitic and non-ascitic patients but without' significant correlations between these parameters. Moreover, these hemodynamic alterations become more marked with development of ascites. Renal hemodynamics showed a significant decrease in hilar renal blood flow and significant increases in renovascular resistance indices in ascitic and non-ascitic patients as compared with healthy subjects. It can be concluded that peripheral arterial vasodilatation particularly in the splanchnic area is the trigerring signal for sodium retention and ascites formation in patients with SHF. Hyperglucagonemia might contribute to the occurrence of these hemodynamic abnormalities. Considering these hemodynamic changes, the use of vasoactive drugs in the management of ascites should be carefully studied in the future.

### F040

### PREOPERATIVE SELECTIVE PORTAL VEIN EMBOLIZATIONS (PPVE) ARE AN EFFECTIVE MEANS OF EXTENDING THE INDICATIONS OF MAJOR HEPATECTOMY IN NORMAL AND INJURED LIVER

D. Elias, T. Debaère, A. Roche, S. Bonvalot, Ph. Lasser
Department of Surgical Oncology, Institut Gustave-Roussy, Comprehensive Cancer Center, Villejuif, France

The objective was to determine retrospectively the efficiency of selective pre-operative portal vein embolization (PPVE) in inducing hypertrophy of the future remaining liver before major hepatectomy resecting a substantial portion of functional liver parenchyma. Twenty eight patients with initially unresectable liver tumors had PPVE between sept 87 and sept'95. Twenty-three of them were curatively hepatectomized after PPVE. They represent 6% of the 371 patients hepatectomized for malignant lesions in the same period. PPVE was done under sonographic guidance, using a simple 5-F catheter, by the transparenchymatous approach of a branch of the portal vein, controlateral to the embolized liver. Different types of PPVE were performed, according to the site of the tumors: right liver, right liver and segment IV, right liver and left lobe, left liver and bisegment V-VIII, and central liver (segment IV-V-VIII). In 23 cases, isobutyl-2-cyanoacrylate glue was injected for free flow distal and proximal embolization, to block the portal tree definitively. 3-D volumetric assessments of the liver were done before PPVE and one month later, before hepatectomy.
**Results** : Induced hypertrophy of the non-embolized liver was successful in 25 cases (89%). The mean percent increase in volume was 70% (SD:50) for the 28 patients. The mean ratio between the remaining liver and the whole functional liver was 21.5% before PPVE, and 33.9% after PPVE. Three patients were not hepatectomized because liver hypertrophy could not be induced and two patients for cancer-related reasons. Two patients died during the postoperative course with no symptoms of liver failure during the three first weeks following hepatectmy.
**Conclusion** : PPVE was an efficient means of inducing hyperthrophy of the future remaining liver in 89% of the cases and permitted a 12.4% mean enhancement in the ratio between this remaining liver and the whole liver. These very good results were due to the distal and proximal free flow embolization technique with a non absorbable material, and an interval of one month between PPVE and hepatectomy. Indications in normal liver parenchyma are for patients with a very small left lobe or necessitating a right hepatectomy with wedge resections in the left liver. Indications for damaged

### F041

**EVALUATION OF THE LIVER FUNCTION WITH THE LIDOCAINE (MEGX) TEST**

G Ercolani, A Mazziotti, GL Grazi, E Jovine, R Callivà, A Gallucci, E Scalzi, G Varotti, A Gardini, A Principe, A Cavallari
2° Dept Surgery, University of Bologna, S.Orsola Hospital, Italy

The lidocaine (MEGX) test, which has been recently proposed as a sensible index of the hepatic function in organ donors, has been employed in the evaluation of patients carrying chronic liver diseases or hepatic tumors. **MATERIAL AND METHODS** The study was carried out in 200 patients admitted from 2/93 to 2/95. There were 120 males and 80 females; their mean age was 52.6 ± 13.4 (ranging from 21 to 80 years). Admission diagnosis were hepatocellular carcinoma in 90 cases, hepatic metastases in 34, decompensated cirrhosis in 41 and different focal liver lesions in 35. Overall, cirrhosis was present in 112 (56%): according to the Child-Pugh classification there were 74 A's, 34 B's and 4 C's. The MEGX test was performed together with the laboratory tests studying the liver function. Values of the MEGX test (normal level > 50 μg/ml) were statistically compared with results of conventional laboratory tests to assess its efficacy in revealing liver failure. **RESULTS** There were no major complications with the MEGX test: 38 (19%) patients experienced minor side effects as dizziness and headache. Linear regression analysis carried out with the MEGX values and the index of hepatic function revealed a significant correlation with total bilirubin, prothrombin activity, ammoniemia, albuminemia, phosphatase alkaline, AST and ALT (P<.05 for each index), but multiple regression revealed a significant correlation only with cholinesterase, ammonemia and total bilirubin. 125 (62.5%) patients had a reduced MEGX values: of these 107 (95.5%) were cirrhotic and 18 (20.5%) were not cirrhotic (P<.05). The sensibility, specificity and diagnostic accuracy of the MEGX test in revealing cirrhosis were 95.5%, 79.5% and 88.5% respectively. **CONCLUSIONS** The MEGX tests appears to be a reliable index in the study of the hepatic function. It is easy to be performed and it promptly gives useful informations on the state of the residual liver function. The test is very sensible for revealing the presence of cirrhosis.

### F042

LIVER TRANSPLANTATION FOR BUDD CHIARI SYNDROME

M. Farouk, S.W. Ahmet, T.R. Kurzawinski, B.R. Davidson, K. Rolles.
University Dept. of Surgery, Royal Free Hospital, London, UK.

Portocaval shunt surgery for end-stage Budd Chiari Syndrome (BCS) is often unsucessful. We report the long term results in 8 BCS patients who were treated by hepatic transplantation over a period of 5 years (age range: 27- 58 years). The indication for transplantation in 6 patients was chronic end stage decompensated liver disease and 2 patients was fulminant liver failure secondary to acute BCS. All patients had hepatic outflow obstruction confirmed by venography which also revealed significant caval obstruction in three patients (retrohepatic gradient>12mm Hg). Standard orthotopic transplantation was performed with veno-venous bypass being used in 5 patients (mean ascitic volume = 6 litres). Recipient hepatectomy was noted to be difficult in all patients with chronic disease because of severe peri-caval fibrosis. Coagulation profiles were continually monitored and the underlying hypercoagulable state treated appropriately by full early postoperative anticoagulation. Using this method, no arterial or portal thrombotic episodes have been encountered. Both patients from the fulminant group died. The first patient with acute BCS died of cardiac arrythmia after unclamping of the graft at the time of surgery. The second patient with acute BCS presented very acutely and initially underwent portocaval shunt surgery but because of gross ascitic fluid production and secondary dilutional coagulopathy required urgent transplantation. Subsequent to this she developed multiorgan failure secondary to sepsis and died 1 month after transplantation. All patients in whom OLT was performed for chronic disease (6) are alive and well with a mean follow up of 52 months (range: 16 - 130 months). In conclusion, we have found that OLT is associated with a good long-term outcome in patients with BCS who present with established hepatic fibrosis. Our limited experience of OLT for acute BCS has not been favourable.

### F043

BACTERIAL TRANSLOCATION DURING PORTAL CLAMPING FOR LIVER RESECTION. A CLINICAL STUDY.

M. Ferri, A. Gavelli, S. Gabriel, Ph. Franconeri, C. Huguet
Department of Surgery, Anesthesiology and Microbiology,
Princess Grace Hospital, Principality of Monaco

**The migration of viable enteric bacteria across the intestinal barrier (Bacterial Translocation) has been experimentally demonstrated after liver resection. A prospective study was carried out to assess the incidence and the clinical significance of bacterial translocation in 14 patients undergoing elective liver resection (10 major hepatectomy) under normothermic ischemia (mean duration : 40 ± 13 minutes). A systemic and portal blood sample and a mesenteric lymph node (MLN) were simultaneously obtained before and after resection, and cultured for a qualitative analysis. All specimens taken before resection were sterile. After resection, blood cultures were sterile in all cases, and the systemic blood remained sterile up to 24 hours; the MLNs were culture positive in 6 of 14 patients (43%). Patients with positive MLN showed no postoperative septic complications. The only septic complication occurred in a patient with a negative MLN. In conclusion, bacterial translocation in the MLNs significantly occurs during portal triad clamping and liver resection, athough it does not predispose to postoperative infectious complications.**

### F044

**A GALACTOSE BASED CONTRAST AGENT TO IMPROVE PORTO-HEPATIC DOPPLERSONOGRAPHY**

**G. Fischer, G. Paumgartner, R. Rak* and M. Sackmann**
Dept. of Medicine II, University of Munich, Klinikum Grosshadern,
81377 Munich, * Schering AG 13342 Berlin; Germany

**Background.** Microbubbles of air packed in galactose (SH U 508 A, Schering) constitutes a doppler-ultrasound contrast agent [DUSCA], which doubles doppler signal intensity from the vessel of interest. DUSCA has the size of erythrocytes and circulates through both the pulmonary and systemic circulation with a half-life-time of about 5 minutès. **Aim** of this study was to investigate effects and tolerability of DUSCA in patients with portal hypertension and insufficient doppler signal intensity, because portal and hepatic veins can not be visualised sufficient enough for conventional dopplersonography [DS] in up to 15% of such patients (J Ultrasound Med; 1990: 9:705).
**Material and methods.** A Hitachi-Picker ultrasound unit with a 3,5 MHz convex array was used for DS. 146 patients with liver diseases of various origin were investigated prospectively (male n=93, female n=53; age 14-83 years, mean 50±13 years). 210 examinations were performed, in 39 patients repeatedly (mean 1.9±1.0 examinations). The patients were referred to DS to determine the presence of portal hypertension (n=49), to exclude portal (n=64), splenic (n=7), or hepatic (n=15) vein thrombosis or to evaluate TIPS function (n=75).
Patients were included in the study when no diagnosis could be made by means of DS. Due to the characteristic effects of DUSCA the study could not be blinded to the investigator. In 194 examinations (92.3%) a diagnosis could be made without DUSCA. The remaining 16 patients (7.7%) received DUSCA to assess portal (n=10) or hepatic (n=4) vein patency or to evaluate TIPS function, (n=2). 8 ml of DUSCA (400 mg/ml) were quickly injected i.v.. Angiography, CT, or MRT, respectively was performed as a control in every patient after receiving DUSCA.
**Results.** Portal and hepatic vein could be unequivocally assessed in 12 patients (portal vein thrombosis n=3, hepatic vein thrombosis n=1, in the remaining 8 patients the vessels were patent), whereas in 2 patients portal vein thrombosis could only be suggested, but not confirmed. TIPS function could be assessed correctly in the 2 patients after receiving DUSCA.
No serious side effects were encountered. 11 patients did not feel anything after injection of DUSCA, 5 patients had short (duration less then half an hour), self limiting episodes of discomfort (bad taste (n=2), temperature sensations at the injection site, dizziness, and palpitations, n=1 each) possibly related to DUSCA.
**Summary and Conclusion.** The galactose based DUSCA SH U 508 A is well tolerated and appears to be of great value in the assessment of portohepatic vessels not amenable to conventional dopplersonography.

### F045

IMPACT OF TIPS ON THE LIVER TRANSPLANT (LT) OPERATION

M. Fraiman, S. Pan, A. Hoffman, C. Cosenza, L. Sher, L. Makowka, R. Lopez
Liver Transplant Service, St. Vincent Medical Center, California, USA

TIPS is being used with increased frequency to treat the complications of portal hypertension in transplant candidates. However, its impact on the transplant operation is unknown. **Methods:** We reviewed our experience with LT in recipients with and without TIPS. The incidence of portal vein thrombosis (PVT), operative blood use, operative time, the use of venovenous bypass (VVB), and survival were compared using t-test and Fisher's exact test. **Results:** Seventy-eight adults had LT for chronic disease from 1/1/94 to 11/30/95 at Cedars-Sinai & St. Vincent Medical Centers. Twelve patients (15.4%) had TIPS placed prior to LT: 23.3% of recipients in 1995 & 10.4% in 1994. Five patients had TIPS in place for > 1yr. There was no difference in the age, disease indication, or operative time between the two groups.

| | operative PRBC's (units/pt) | partial or complete PVT % (n) | use of VVB % (n) | 30 days survival (%) | 1 year survival (%) |
|---|---|---|---|---|---|
| TIPS (n=12) | 5.1 ± 4.7 | 41.7 (5) | 25.0 (3) | 91.7 | 75.0 |
| no-TIPS (n=66) | 8.2 ± 8.9 | 6.1 (4) | 60.6 (40) | 97.0 | 93.9 |
| p-value | 0.248 | 0.003* | 0.029* | 0.400 | 0.069 |

*p<0.05

Five patients (41.7%) with TIPS had partial or complete PVT at the time of LT (4 with TIPS in place for > 1 yr.). Four of these needed thrombectomy prior to porto-portal anastomosis and 1 required an interposition vein graft to the superior mesenteric vein. In 2 patients (one with PVT), the TIPS extended to the confluence of the superior mesenteric and splenic veins necessitating extraction prior to portal anastomosis

**Conclusions:** TIPS effectively controls portal hypertension prior to LT and may decrease the need for VVB. However, it predisposes patients to PVT, particularly if placed > 1 yr prior to LT, and may impact the transplant operation. In the transplant candidate, TIPS should be performed by an experienced radiologist and considered a temporary bridge to liver replacement. The transplant surgeon should be prepared for portal venous reconstruction in patients with TIPS.

### F046

**HIGHLY-SENSITIVE IDENTIFICATION OF α-FETOPROTEIN mRNA IN CIRCULATING PERIPHERAL BLOOD OF HEPATOCELLULAR CARCINOMA PATIENTS**

Naomi Funaki, Junji Tanaka, Shin-ichi Seto, Takayuki Kasamatsu, Toshimi Kaido and Masayuki Imamura.

First Department of Surgery, Kyoto University, Kyoto, Japan

In order to capture hepatocellular carcinoma (HCC) cells in circulating peripheral blood, we made analysis to see if α-fetoprotein (AFP) mRNA exists in the peripheral blood obtained from patients with HCC and also, as a control, from hepatitis-viral-marker-positive patients without HCC and a healthy volunteer. As the number of HCC cells in 1cc of peripheral whole blood and the quantity of AFP mRNA are expected to be very small, the analysis was performed by the reverse transcription followed by an original three-step polymerase chain reaction. By this highly-sensitive method, 5 of 7 HCC patients were positive for AFP mRNA. These 5 positive patients consisted of 3 with clinically apparent recurrence, one preoperative patient with tumor thrombus in the portal vein and one recurrence-free patient who developed clinically detectable recurrence 3 months after this analysis. On the other hand, one follow-up patient without any clinical recurrence and negative for AFP mRNA showed no recurrence even after 3 months. One preoperative patient, whose serum AFP protein level was as high as 67213 ng/ml was negative for AFP mRNA. Neither 4 patients with positive viral markers nor a healthy volunteer was positive. The results suggest that detection of AFP mRNA from HCC patients' peripheral blood by our highly-sensitive RT-PCR may be a practical and powerful tool to diagnose the preoperative spreading of HCC and to monitor its recurrence.

### F047

AN IMMUNOLOGICAL COMPONENT TO SUICIDE GENE THERAPY

S. Gagandeep, I. Pope, B. Green, S. Christmas, [2]D. Klatzmann, A.R.Kinsella and G.J. Poston.
Department of Surgery, University of Liverpool, Liverpool, U.K.
[2]L' Hopital de la Pitie Salpetriere, Paris, France.

The integration and over expression of the herpes simplex virus type 1 thymidine kinase (HSV1-TK) gene in localised tumours, results in tumour regression following the administration of the specific nucleoside analogue ganciclovir (GCV). Although only 10-20 per cent of the tumour cells take up the HSV1-TK the neighbouring cells die as a consequence of what has been termed the "bystander effect". Subcutaneous tumours were created following the injection of 1 x $10^6$ cells of the mouse colon adenocarcinoma cell line MC26. All control mice were co-injected with 1 x $10^6$ cells of the NB16 packaging cell line expressing the nls-lacZ gene and all test mice were co-injected with 1 x $10^6$ cells of the PLJ-TK packaging cell line expressing the HSV1-TK gene. The mice were divided into four groups: nude Balb/C mice into a control and a test group (Groups 1 and 2 respectively) and normal Balb/C mice into a control and test group (Groups 3 and 4 respectively). Seven days were allowed for retroviral gene transduction and tumour growth prior to treatment with GCV twice daily for five days. At the end of this time the animals were sacrificed and the tumour volume in each group was assessed. A significant tumour regression was observed in the test groups versus the control groups. The mean tumour volume was 42.1$mm^3$ in the control groups (Groups 1 and 3) compared with 3.3$mm^3$ in the test group 4 (p<0.01). The test group for nude mice did not respond with the same efficacy only reaching a reduced tumour volume of 20.5$mm^3$ (p<0.05) These data demonstrate a near complete regression of established subcutaneous tumour in normal Balb/C mice following the successful transduction of the HSV1-TK suicide gene followed by treatment with GCV. The same was not true for the Balb/C nude mice suggesting a strong cell mediated immune component to the 'bystander effect'. It is therefore possible that suicide gene therapy may trigger a more general anti-neoplastic action by facilitating a specific anti-tumour immune response. Further experiments to determine if there is a generalised systemic anti-tumour immune response are in progress. We have also developed an animal model for the treatment of multiple hepatic metastases in the rat, with the packaging cell line delivered by hepatic artery cannulation. In this way it may be possible to treat patients with otherwise inoperable hepatic metastases by suicide gene therapy.

### F048

AGEING ENHANCES ANOXIA/REOXYGENATION INJURY IN ISOLATED RAT HEPATOCYTES.

A. Gasbarrini, S. De Notariis, M. Simoncini, P. Caraceni, G.R. Angelini, G. Sica, A. Colantoni, F. Trevisani, A. Mazziotti, A. Cavallari, P. Pola, M. Bernardi.
Angiology and Internal Medicine, Catholic Universty of Roma; Medical Pathology, University of Bologna, Italy.

The reoxygenation phase that follows a period of prolonged anoxia is characterized by the massive formation of oxygen free radicals (OFR), highly reactive substances responsible of damage to cell membranes. Ageing is associated with a reduction of antioxidant status. Aim of this study was to determine the effects of ageing on the sensitivity of liver cells to a period of anoxia/reoxygenation. OFR formation and cell injury were evaluated in hepatocytes isolated from rats of different ages.

METHODS: Sprague Dawley male fed rats of 2 or 8 months of age were utilized. After isolation, liver cells were cast in agarose gel threads and perfused with oxygenated Krebs Henseleit bicarbonate buffer (KHB). A 2 h period of anoxia was obtained shifting the gas phase of the perfusate to 95%N2-5%CO2. Successively, the cells were reperfused for 1 h with oxygenated KHB. OFR were evaluated by enhanced chemiluminescence: anion superoxide (O2-) by adding 1 mM lucigenin to the solution, hydrogen peroxide (H2O2) by adding 10 mM luminol. Cell damage was assessed by measuring LDH release in the effluent.

RESULTS: in basal conditions, no differences were observed in the levels of lucigenin or luminol-enhanced chemiluminescence between the two groups (13±1 vs 14±4 nA and 23±3 vs 24±5 nA, respectively). During anoxia, lucigenin or luminol-enhanced chemiluminescence, expression of O2- and H2O2 formation, respectively, decrease to background values, while LDH release was significantly greater in cells isolated by older animals (750% vs 490% after 2 h of anoxia; p<0.05). During reoxigenation, OFR formation increased in both groups; such a rise, however, was markedly greater in cells obtained from older rats: O2- reached a peak after 15 min (140±10 vs 100±11 nA; p<0.05), while H2O2 increased progressively during the hour of reoxygenation (121±9 vs 83±12 nA; p<0.05). LDH release rise markedly during reoxygenation in both groups; newly, however, higher values were observed in cell obtained from older rats (1300% vs 750%; p<0.05). The peak of lucigenin-enhanced chemiluminescence, expression of O2- production, was observed 10-15 min before the peak release of LDH.

CONCLUSIONS: our results show that: 1) liver cells produce high level of anion superoxide and hydrogen peroxide during the reoxygenation phase that follows a period of prolonged anoxia; 2) the age of the rats influences the sensitivity of liver cells to anoxia/reoxygenation injury.

### F049

**SPLIT LIVER TRANSPLANTATION - IS THE RIGHT GRAFT RECIPIENT AT RISK ?**

K.A: Gawad, X. Rogiers, M. Malagó and C.E. Broelsch
Department of Surgery, University of Hamburg, Germany

**Introduction:** The increasing shortage of donor organs has led to an increased application of split liver transplantation. Three different techniques for the partition of the liver were applied: The classical technique and the new techniques of ex-situ and in-situ (in the heart beating cadaveric organ donor) splitting that have been introduced by our group.
**Patients and methods:** Between 1.1.94 and 31.12.95 thirtyone split liver transplantations have been performed at our department. During the same time 115 whole organ transplantations were performed. 15 patients with a mean age of 33 (5-64) years, a mean weight of 62.5 (28-89) kg and a mean donor/recipient ratio of 1.1 (0.7-1.9) received right liver grafts. 5 (31 %) transplantations were performed high urgent.
**Results:** *Classical technique:* patient survival 3/5, graft survival 2/5. Median cold ischemic time 850 (550-1005) minutes. Median postoperative GOT-peak 617 (120-1100) U/l. *Ex-situ technique:* patient and graft survival 3/5. Median cold ischemic time 715 (510-960). Median postoperative GOT-peak 534 (168-4389). *In-situ technique:* patient and graft survival 6/6. Median cold ischemic time 690 (285-875). Median GOT-peak 196 (122-1232) U/l.
**Conclusion:** Partition of the liver in the heart beating cadaveric organ donor results in a right graft with superior initial function and a better survival compared to the "ex-situ" techniques. Should these results be confirmed with increasing numbers a systematic approach to liver splitting with the in-situ technique should be aimed at.

### F050

**DISTAL SPLENO-RENAL SHUNT (DSRS):LONG TERM PORTAL PERFUSION (PP) MAINTENANCE.**

Gerunda G.E.; Zangrandi F.;Neri D.; Bisello M.; Merenda R.; Barbazza F.; Meduri F.; Da Giau G., Ciardo L.; Bruttocao A.; Maffei Faccioli A.
First Department of General Surgery - University Padua.

Maintenance of high portal perfusion is the most important feature of DSRS. When spleno-pancreatic disconnection (SPD) is added survival and quality of living are increased. 91 Patients that previously bleeded from oesophageal varices underwent DSRS : in the first 26 a traditional, limited, porto-splenic disconnection was performed ; in the other 65 a spleno-pancreatic, spleno-colic and gastro-colic disconnection (SPD) was added. Peri-operative mortality was 14%, related to Child's risk (Child A-B 4%). No mortality due to rebleeding from esophageal or gastric varices was reported; loss of long-term PP was observed in 23%. Persistence of disease and incomplete SPD were the two prognostic factors related to survival (38% vs 11% -p<0.001 and 43% vs 14% -p<0.05 respectively).

DSRS - PERFUSIONE

SOPRAVVIVENZA

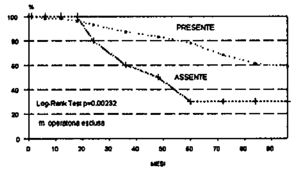

DSRS:TYPE OF DSRS AND ELECTIVE EVS
SURVIVAL

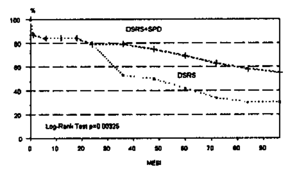

Porto-systemic encephalopathy (PSE) was found in 24% (evident in 4%) and was influenced by worsening of PP (57% vs 23% -p<0.01), by persintence of disease (48% vs 17% -p<0.02) and incomplete SPD (48% vs 14% -p<0.05).

### F051

**LOSS OF HETEROZYGOSITY ON CHROMOSOME 16q IN SMALL (<4 cm) HEPATOCELLULAR CARCINOMA.**

L.Gramantieri, B.Stecca*, S.Gaiani, A.Mazziotti**, P.Chieco*, A.Casali, L. Masi, A.Cavallari**, L.Bolondi. Dipartimento di Clinica Medica e Gastroenterologia; *Istituto Oncologico F. Addarii, Ospedale S.Orsola, Bologna; **Clinica Chirurgica II Università di Bologna, Italy.

Introduction: The genesis of human cancers, including HCC, is thought to be due to multiple genetic alterations. Loss of heterozygosity (LOH) has been frequently found at multiple loci in HCC: 1p, 4q, 5q, 8p, 8q, 10q, 11p, 13q, 16q, 17p, 22q. Loss of some putative tumor suppressor genes within these chromosomal regions might be involved in transformation of hepatocytes to malignant tumor cells.
Aim of this study was to analyze small HCC without signs of local or systemic spread, to define a minimal region of loss on chromosome 16q, where other previous reports made on unselected cases of HCC found large areas of LOH.
Materials and Methods: twenty-eight cases of small (less than 4 cm in diameter) HCC were selected. Extracted DNA was screened for LOH by means of PCR-SSCP. Blood lymphocytes, liver cirrhosis and HCC were available for 6 cases, being available in the remaining cases either blood lymphocytes or liver cirrosis.
Results: analysis of 10 polymorphic microsatellite markers on chromosome 16q identified 3 out of 28 cases of small HCC exhibiting LOH of 16q in at least one locus. One tumor had monosomy of 16q, displaying allelic loss for all informative markers tested, and 2 out of 19 informative cases revealed a minimal area of loss at 16q24 (D16S413 locus). None of the cases of liver cirrhosis presented LOH in the loci analyzed.
Conclusions: our data confirm in some small HCC, the LOH on chromosome 16q that was frequently found in advanced HCC. The deletion of this area was associated to large, metastasized HCC in a previous report, where only 6 early lesions were examined. In our study the minimal area of loss was restricted at 16q24 (D16S413 locus).

### F052

**COMBINED, ARTERIAL AND PORTAL, OILY CHEMOEMBOLIZATION (OCE) OF LIVER NEOPLASMS**

A.M.Granov, D.A.Granov, P.G.Tarazov

Dept Surgery & Div Angio/Interventional, St.Petersburg Research Institute of Roentgenology and Radiation Therapy, St.Petersburg, Russia

The purpose of this study was to assess the effecacy of combined, arterial and portal, OCE *vs* arterial OCE alone in patients with unresectable liver malignancies.
Between 1986 and 1994, 109 patients with advanced hepatocellular carcinoma ( HCC, 27 cases ) and liver metastases ( Mts, 82 cases ) underwent 179 courses of OCE. We used a new liposoluble cytostatic Dioxadet (15-20 mg) or Doxorubicin (30-100 mg) in iodized oil for arterial procedure. In 45 patients, the same solution was injected into the portal vein 15-20 days later.
The 1-2-3yr survival for arterial OCE was 20%, 10%, 0% for HCC, 51%, 6%, 0% for colorectal and 43%, 22%, 14% for other liver Mts. The same rates for combined treatment were 83%, 42%, 25% for HCC, 81%, 23%, 9% for colorectal and 91%, 36%, 9% for other Mts.
These data show that portal OCE may be useful addition to arterial OCE in patients with unresectable liver malignancies.

### F053

**HBV INFECTION DOES NOT AFFECT SURVIVAL AFTER LIVER TRANSPLANTATION.**

GL Grazi, A Mazziotti, C Sama, E Jovine, FG Stefanini, A Gallucci, V Pilotti, G Varotti, A Cavallari
2° Dept. Surgery, University of Bologna, S.Orsola Hospital, Italy

Liver transplantation (LT) has became the elective treatment for end-stage liver diseases: questions still arise on its efficacy in HBsAg positive cirrhotics. ***MATERIAL AND METHODS*** Of the 123 cirrhotics grafted over the past 9 years, 39 (31.7%) were HBsAg positive. Four (10.3%) were HBeAg positive, 1 (2.6%) was HBV-DNA positive and 17 (43.6%) were anti-Delta positive. An HBV specific intramuscular prophylaxis (to keep anti-HBs titer over 100 mU/ml) was carried out in the latter 28 cases. ***RESULTS*** Three HBsAg patients (7.7%) died within 30 days for HBV unrelated diseases (2 M.O.F. and 1 primary graft non function). Survival of the entire group of 123 cirrhotics was 75.7% and 69.9% after 1 and 3 years respectively. Twelve (33.3%) patients returned HBsAg positive after a mean time of 10.3 ± 15.5 months (range 43 days-55.8 months): recurrence appeared in 3 (75%) HBeAg positive patients and in 5 (29.4%) out of the 17 anti-Delta positive. The 1 HBV-DNA positive died 80 days after LT because of stroke. The recurrence rate was 72.7% in the group of patients without prophylaxis and 16% in the group with prophylaxis (p=0.0008). Survival was 77.8% and 73.3% for HBsAg negatives and 71.7% and 63.1% for HBsAg positives after 1 and 3 years respectively (p=NS). The hazard of HBsAg recurrence in treated patients was reduced to 24.9% as compared to untreated patients. There were 3 deaths due to HBV relapse, 2 in the untreated group and 1 in patients treated with immuno globulins. Of the 8 surviving patients with HBsAg recurrence, 3 (37.5%) did not present signs of the disease, while in 5 (62.5%) cases recurrence developed into a mild chronic hepatitis. ***CONCLUSIONS*** Recurrent disease can occur after LT for HBV related cirrhosis with evidence of viral replication. Prophylaxis protocols seem effective in the prevention of HBsAg reappearance after LT and they represent a further tool in the postoperative treatment of HBsAg positive cirrhotics. LT can be successfully performed in these patients only with the adoption of such protocols.

### F054

**PORTAL VEIN THROMBOSIS AFTER SPLENECTOMY AND DEVASCULARIZATION FOR BLEEDING ESOPHAGEAL VARICES: A POTENTIALLY LETHAL SYNDROME**

I. Hamdi, A. Khamag, J. Faitori
Department of Surgery, Arab Medical University, Benghazi, Libya

Although portal vein thrombosis is a well-recognised phenomenon in patients with liver cirrhosis, its incidence after splenectomy and esophago-gastric devascularization is underappreciated.

We present nine cases of thrombosis of the portal vein after splenectomy and devascularization (Hassab operation) performed on 46 patient (19.6%) for bleeding esophageal varices. There were 7 men and 2 women. The mean age was 42 years (range, 32-64 years). Six patients were classified as Child grade C and three patients were Child grade B. Seven patients survived the event and two patients died, representing mortality rate of 22.2%. We currently employ the flow Doppler ultrasonography for both diagnosis and follow-up. Resolution of the thrombus was observed in one patient and cavernous transformation of the portal vein occurred in 6 patients. Mesenteric venous infarction of the small bowel was not encountered in any patient in this series.

Portal vein thrombosis should be suspected in patients who develop fever, abdominal pain, and with or without intestinal complains after Hassab operation, even if the operation is remote. Prompt treatment is lifesaving.

### F055

A LOW CENTRAL VENOUS PRESSURE REDUCES BLOOD LOSS DURING LIVER SURGERY

S.S. Hanna, J. Proctor, T.P. Hanna
Division of General Surgery, Sunnybrook Health Science Centre, University of Toronto, Toronto, Canada

A retrospective study was carried out using our liver resection data base to determine the relationship between Central Venous Pressure (CVP) and blood loss during liver resection. The data base contained 110 patients who underwent liver resection between 1980 and 1995 and who had their CVP recorded. Since January 1, 1991 an attempt was made to keep the CVP as low as possible especially during the liver transection phase. Prior to January 1, 1991 no attempt was made to maintain a low CVP. Patients were divided into 2 groups: major liver resections (n=66) and minor liver resections (n=44). A liver resection of 1-2 Couinaud segments was considered a minor liver resection and a resection of 3 or more segments was considered a major liver resection. Mean CVP, Estimated Blood Loss (EBL), and Number of Units of Blood transfused (NUB) were compared in major and minor liver resections before (n=49) and after (n=71) January 1, 1991. There were 31 major and 18 minor liver resections before January 1, 1991 and 35 major and 26 minor liver resections after that date. Analysis of variance confirmed that after the introduction of our low CVP policy there was a decrease in both EBL and NUB; and as expected the reduction was greater in patients undergoing major liver surgery (P=0.01). A low CVP is associated with less blood loss during liver resection. It is recommended that liver surgeons communicate this information to the anaesthetists involved in the care of their patients in order to achieve the goal of keeping the CVP at or below 5 cm $H^2O$ to minimize blood loss during liver surgery.

### F056

**SEGMENTAL VOLUME IN LIVING RELATED TRANSPLANTATION (LRT): CLINICAL CORRELATION AND DONOR: RECIPIENT RATIO**

TG Heffron, AN Langnas, IJ Fox, AJ Matamoros, JC Anderson, DR Mack, TM McCashland, A Dhawan, S Kaufman, RK Zetterman, TJ Pillen, D Sudan, J Jerius, JA Vanderhoof, BW Shaw Jr.

**Reduced sized cadaveric and LRT were developed in response to pediatric donor shortage. The pediatric donor pool may be enhanced by using the largest donor/recipient ratio possible. However, large disparity between donor and recipient graft size may lead to significant problems resulting in graft and/or recipient loss. Preoperative estimation of segmental volume is necessary to avoid donor/recipient size disparity.Accuracy of preoperative segmental volume measurement and effect of donor recipient size disparity were retrospectively reviewed in 26 LRT's and one cadaveric donor. Material and Methods: Over 3 years LRT recipients(median age.8 years and wt 8kg) accounted for 29% (26/91) of pediatric liver transplants. Left lateral segmental (LLS) volumes were measured preoperatively via CT volumetry in living related donors and compared with surgical weight and displaced volume. A 6 kg recipient in acute fulminant hepatic failure emergently utilized a cadaveric LLS from a 130 donor requiring further resection of the left lateral segment. Results: Donor/recipient weight ratio ranged from 2.9 - 21.5 (average 11.1). Linear Regression Correlation Coefficient: Surgical measured volume/surgical measured weight (N=12) r = .9: Preoperative estimated volume/surgical measured wt(.N=26) r = .6: Donor weight/surgical measured weight (n=26) r = .2. Preoperative volume (n=26) average 229 ml (157-363ml), Surgical segmental volume (n=12) average 292 ml (200-300ml), Surgical measured weight (n=26) average 298.5 g avg (190-440 g). Patient and graft 2 year actuarial survival in the LRT group was 87% and 80% respectively. No grafts were lost secondary to donor recipient size disparity. Conclusion: Preoperative segmental volume estimation via linear CT scanning is an accurate method to determine segmental liver volume before donor hepatectomy. Left lateral segmental volume could not be accurately predicted from donor weight. Segments from donors greater than 20 times the recipient weight may be successfully utilized for transplantation. In emergent conditions, LLS grafts may be further reduced.**

F057

DISTAL SPLENORENAL SHUNT IN 1990'S: PATIENT SELECTION AND OUTCOME.

J. M. Henderson, C. Agnor.
The Cleveland Clinic Foundation, Cleveland, Ohio 44195, USA

This study prospectively evaluated 42 patients having distal splenorenal shunt (DSRS) from 10/92 to 10/95. Selection for DSRS was based on: variceal bleeding refractory to endoscopic and pharmacologic therapy; adequate liver function (Childs A-31, B-10, C-1); etiologies were alcoholic-14, cryptogenic-9, PBC-6, post hepatic-6, portal vein thrombosis-3, others-4. Five patients were shunted emergently, and 37 had elective DSRS, with standard DSRS in 37 patients and modified selective shunts in 5. There was no hospital mortality. The median hospital stay was 9 days, with median hospital charges of $23,000. There was one shunt thrombosis in a patient with a myeloproliferative disorder who required splenectomy/devascularization. Early rebleeding occurred in 3 patients (7%): all had patent shunts, and bleeding resolved with nonoperative management. Late rebleeding occurred in one patient (2%) in the presence of a patent shunt. Mild/moderate encephalopathy was found in 4 patients (10%) at median follow-up of 18 months. There have been 2 late mortalities, one from liver failure and one with a progressive myeloproliferative disorder. Two patients have required liver transplant to date, one with sclerosing cholangitis, and one with a cryptogenic cirrhosis 16 months after emergent DSRS. We conclude that DSRS plays a role in managing patients with variceal bleeding in the 1990's: good risk patients can have variceal bleeding controlled by DSRS with low morbidity.

F058

**PORTAL VEIN THROMBOSIS IN ADULT CANDIDATES TO LIVER TRANSPLANTATION.**

*Hidalgo,E.,Bilbao,I.,Lázaro,JL.,Murio,JE., Balsells,J.,Charco,R.,Gifre,E.,Ruiz,C., Margarit,C. Liver Transplant Unit. Hospital. Gral. Universitari Vall d'Hebron. Barcelona. SPAIN.*

The Portal Vein Thrombosis (PVT) is a common complication observed in patients with end-stage liver diseases,and it has been considered as relative contraindication. We have studied a total of 195 LTx in 177 patients, made from December 1988 to August 1995, with 18 retransplantations. The median age of the group was 51 years old,19 male and 5 female. The main end-stage liver diseases which required LTx were:Postnecrotic cirrhosis (111/195), Malignant hepatic tumors (36/195), Cholestatic diseases (13/195) and Acute hepatic failure (8/195). The preoperative diagnosis was done by Doppler ultrasonography and detected 4.6% thrombosis (9/195). The real incidence of PVT found at time of surgery was 12.3% (24/195). Nine out of 24 PVT were complete (total occlusion of portal trunk) and 15 were partial. A higher incidence of PVT was observed in patients affected by CHV (15/86 vs. 9/109, $X^2$=3.75, p=0.05). Upper GI bleeding and sclerotherapy sessions were more frequent in patients with PVT (p<0.05). During surgical procedure, resection and end-to-end anastomosis was performed in 15 cases, thrombectomy and anastomosis in 7 cases, anastomosis at confluence of mesenteric superior vein and splenic vein was performed in one case, and anatomic venous iliac graft in another. When we compared operative mortality and postoperative mortality there were no significant differences. Only one patient who had had PVT before LTx presented thrombosis after LTx, while 2 patients without previous PVT developed further thrombosis (1/24 vs 2/171 ,NS). There was a higher incidence of hepatic artery thrombosis developed after LTx in those patients with previous PVT:3/24vs 43/171, $X^2$=6.27, p= 0,013 and OR= 5.9. In summary we believe that PVT can not be considered as a contraindication to LTx.

F059

TREATMENT OF IRRESECTABLE HEPATOCELLULAR CARCINOMA WITH REPEATED TRANSIENT DEARTERIALIZATION. (A RANDOMIZED STUDY)

[1]J.F. Huang, [1]W-S. Wang, [1]L-J. Liang, [1]M-D. Lu, [2]B. Jeppsson, [2]B. Persson, [2]S. Bengmark

[1]Department of Surgery, First Affiliated Hospital Sun Yat-Sen University of Medical sciences, Guangzhou, China, [2]Department of Surgery, Lund University, Lund, Sweden

40 patients with the irresectable hepatocellular carcinoma (HCC) admitted to the department of HPB surgery in the first affiliated hospital of SUMS were randomized into two groups: 20 (each) from February 1994 to April 1995. Patient's inclusion criteria are non-resectable primary hepatocellular carcinoma. Preoperative diagnosis was confirmed by ultrasonography or computed tomography with biopsy.

40 patients were treated with hepatic artery ligation (HAL) and repeated transient dearterialization (RTD) respectively. Postoperative response to treatment, liver function change (ALT), AFP, imaging examination of the tumour, patient's survival were evaluated in two groups.

It has been shown that RTD is superior to HAL in terms of the objective response to therapy, reduction of tumour size, patient's symptom relief, liver function and AFP changes and patient's survival. (In RTD group, operative mortality was 0, morbidity was 10%, the effective rate was 70%, the mean survival time was 8. 2 months, 6 month survival was 79. 7%; in HAL group, operative mortality was 35%, morbidity was 35%, the effective rate was only 5%, mean survival time was 5. 1 months, 6 month survival was 35.8 %).

It seemed to us that RTD would be a promising palliative option for HCC and it has its own significance of the armamentarium for the management of HCC.

F060

**PERCUTANEOUS NEEDLE ASPIRATION AND DRAINAGE OF SOLITARY HYDATID CYSTS OF THE LIVER.**

**R.Ikramov, V.Vishnevsky, A.Gavrilin.**
**A.V.Vishnevsky Institute of Surgery,Moscow,Russia.**

**We present a method of percutaneous treatment of hydatid cysts of the liver which reliably prevents dissemination of scoleces of the parasite within the abdominal cavity and development of anaphilactic shock. The method involves percutaneous puncture of the cyst with CHIBA needle, aspiration of cyst content, injection of antiparasitic solution /30% NaCl/ for 10 - 15 min. with subsequent puncture of the cyst with troacar- catheter and aspiration of cyst content with chitinous membraine/.The cysts where drained for 2 weeks with irrigation of cyst with 30% NaCl solution. Futher removal of remnants of the chitinous membrane was performed under the gidence of fistuloscopy and fistulography.Eight patients were treated by this method with no mortality and morbidity. Follow-up of 7 pts. for 3-10 years showed no recurrences.**

### F061

TITLE: Effect of Total Hepatic Vascular Exclusion During Liver Resection on Hepatic Ultrastructure

Authors: A.M. Isla, M.E. Moussa, C.E. Sarraf, H. Sawada, N.A. Habib

ABSTRACT:

Total vascular exclusion (TVE) during liver resection is achieved by clamping the hepatic artery, portal vein, infrahepatic and suprahepatic vena cava. It aids the surgeon in resecting lesions close to major blood vessels and diminish blood transfusion. A major drawback of TVE is liver ischaemia.

Eleven non cirrhotic patients that underwent major resectional liver surgery under TVE were studied. The duration of TVE (warm liver ischaemia) ranged between 16 and 48 minutes. Biopsies were taken at three stages: pre-ischaemia, before the application of vascular clamps; ischaemic, just at the end of TVE; and post-ischaemia, 12 to 120 minutes following reperfusion. Light-electron microscopy study was performed to study the hepatic architecture, hepatocyte morphology, bile canaliculi and sinusoids. All specimens were coded and assessed blindly for tissue (light microscope level) and cellular (electron microscope level) changes. Prior to TVE hepatocytes and hepatic architecture were as normal as could be expected. During ischaemia the hepatic architecture was distorted due to widespread collapse of sinusoidal spaces. Hepatocytes showed focal chromatin condensation at the nuclear margins, distented nuclear envelope and swelling of both mitochondria and endoplastic reticulum. After reperfusion these changes reversed.

It can be concluded that TVE over a period of 48 minutes has no irreversable deleterious effects on the ultrastructure of the non-cirrhotic liver.

### F062

HIGH PREOPERATIVE SERUM ALANINE TRANSAMINASE LEVEL INCREASES THE RISK OF LIVER RESECTION FOR HEPATOCELLULAR CARCINOMA (HCC) IN CHILDS' GRADE A CIRRHOTIC PATIENTS

P. Jagot, O. Farges, R. Noun, J. Belghiti.
Department of Digestive Surgery, Hospital Beaujon, University Paris VII, Clichy, France

Liver resection in patients with liver cirrhosis (even in the absence of overt liver insufficiency) is associated with a greater risk than in patients without underlying liver disease. Because the incidence of HCV-cirrhosis related HCC is anticipated to increase rapidly in the near future we have assessed, by multivariate analysis, parameters associated with in-hospital mortality and morbidity in a consecutive series (1984-1994) of 108 Childs' grade A cirrhotic patients undergoing liver resection of an HCC (1 or less liver segment, 2 segments or 3 or more segments in 42, 23 and 43 patients respectively).
Parameters entered for analysis included age, aetiology of cirrhosis, preoperative serum bilirubin, AST, ALT, GGT, albumin, creatinine levels as well as prothrombin time, presence or absence of pathological features of superimposed active hepatitis, extent of resection, type and duration of vascular clamping and amount of intraoperative blood loss.
Overall incidence of in-hospital death and major postoperative complications were 8.3% and 48.1% respectively. By univariate analysis, preoperative serum ALT levels (p=0.001) and intraoperative transfusions (p=0.01) were the only parameters significantly associated with in-hospital death. However, only serum ALT concentrations was an independent risk factor. In-hospital mortality in patients whose preoperative serum ALT was below 2N (n = 77), comprised between 2 and 4N (n = 23) and greater than 4N (n = 8) was 3.9 %, 13.0 % and 37.5 % respectively. Increased ALT levels (> 2N) was also associated with an increased incidence of postoperative ascites (58 vs. 32 %, p=0.01), kidney failure (16 vs. 0 %, p=0.0003) and UGI bleeding (6.4 vs 0 %, p = 0.02).
Conclusion: In contrast to previous studies, we have shown that preoperative serum ALT level is an independant and reliable predictor of in-hospital mortality and morbidity following liver resection in Child A cirrhotic patients. Our results suggest that cirrhotic patients with ALT > 2N should undergo only a subsegmentectomy or, if a larger resection is considered, be considered as liver transplant candidates. Alternatively, anti-viral therapy may prove usefull in patients with an HCV-related cirrhosis.

### F063

**PIGGY BACK VS CONVENTIONAL TECHNIQUE IN LIVER TRASPLANTATION: PRELIMINARY REPORT OF A RANDOMIZED TRIAL.**

E Jovine, A Mazziotti, GL Grazi, M Morganti, B Nardo, M Masetti, F Pierangeli, B Begliomini, P Gaito-Mazzetti, R Paladini G Martinelli, A Cavallari
2° Department of Surgery, University of Bologna.

The Piggy-back technique of liver trasplantation preservs the full length of the recipient inferior vena cava and avoids the utilization of veno-venous by-pass. We are performing a randomized trial to asses if there are any significative advantages of the piggy back versus the conventional technique in adult trasplant recipients. **MATERIALS AND METHODS:** From January 1995 and December 1995, 54 liver transplantations in adults were performed in our Institution, 30 of whom were included and randomized in our study. All the patients considered were cirrhotics and patients with previous abdominal surgery, acute hepatic failure, renal insufficiency or retransplantations were excluded. In the Piggy back technique a temporary porto-caval shunt has never been used. All conventional operations have been performed utilizing a Griffith veno-venous by-pass. Hemodynamic parameters (CO, CVP, MAP, AMP, pH, $pO_2$, $pCO_2$, BE) registred at different time of operation, blood loss, cold and warm ischemic time, surgical time, intraoperative and postoperative complications, graft function, ICU and hospital stay, were evaluated in both techniques. **RESULTS:** Two patients randomized as Piggy back were switched to conventional technique for a presence of a caudate lobe embracing posteriorly the retrohepatic vena cava and for a caval lesion respectively. No patients developed vascular complications or postoperative hemorrhage. There were no intraoperative deaths. The following table shows main evaluated parameters:

| ‡ p < 0.05 | Blood loss (l) | Operating time (min.) | Warm ischemia (min.) | PGNF (#) | Renal Failure (#) |
|---|---|---|---|---|---|
| Piggy Back (13) | 1.9±1.2 | 476.8±82.8 | 48.5±14.5 ‡ | 3 (20%) | 0 ‡ |
| Conventional (15) | 2.7±2.9 | 500.8±95.0 | 60.6±12.6 | 3 (23%) | 4 (30.8%) |

There were no significant differences in the intraoperative hamodynamic parameters. We observed a trend toward the reduction of ICU (7.3±7.1 vs 4.3±1.3) and total hospital stay (27.5±17.6 vs 17.0±3.0) with the use of the piggy back technique. **CONCLUSIONS:** These preliminary results don't show any statistical differences except for the renal insufficiency and the warm ischemic time. The Piggy back technique is feasible in the majority of the cases and can be used safely in patients who undergo liver transplantation saving costs (veno-venous by-pass, intraoperative blood requirement, hospital stay). Further evaluations are necessary to establish the real supremacy of this technique.

### F064

**SIGNIFICANCE OF MEMBRANE FLUIDITY OF HEPATOCELLULAR CARCINOMA IN POSTOPERATIVE RECURRENCE**

T.Kasamatsu[1], J.Tanaka[1,2], N.Funaki[1], T.Kaidou[1], S.Seto[1], M.Imamura[1]

[1]First Department of Surgery, Kyoto University,

[2]Department of Surgery, Shiga Medical Center for Adult Diseases, Kyoto, Japan

Membrane fluidity of tumor cells has been suggested to influence tumor invasion or metastases. The present study was conducted to clarify whether or not membrane fluidity (MF-value) of hepatocellular carcinoma (HCC) is related to postoperative recurrence. Membranes of resected HCC tumors and non-tumorous liver tissues from the same patients were prepared by gradient ultracentrifugation and fluidity was determined by spectrofluorophotometer equipped with polarizer using 1,6-diphenyl-1,3,5-hexatriene as a probe dye. MF-values of both tumor and non-tumorous tissues (0.232 and 0.231, n=55, individually) were significantly higher than that of normal liver controls (0.190, n=12, p<0.001). However, no significant difference was observed between tumor and non-tumor, as a whole. Although MF-values of HCC's did not concern tumor size, number, capsular invasion or intrahepatic metastasis, they significantly related to tumor invasion into the portal vein (p<0.05) and serum α-fetoprotein value (p<0.05). Recurrence group (0.212, n=23) had a significantly lower MF-value than that of recurrence-free group (0.242, n=32, p<0.01). From the comparison of MF-value between tumor and non-tumorous liver tissue in individual case, HCC patients were classified into three groups.

- Group I : MF-value of tumor is higher than that of non-tumor and the difference is more than 0.01. (n=28)
- Group II : MF-value of tumor is lower than that of non-tumor and the difference is more than 0.01. (n=19)
- Group III : Difference in MF-value between tumor and non-tumor is less than 0.01. (n=9)

Three-year disease-free survival rates were 54% in group I and 8% in group II and p=0.021 by log-rank analysis.
In conclusion, membrane fluidity intimately concerns postoperative recurrence of HCC after hepatic resection.

### F065

### *FACTORS AFFECTING RECURRENCE IN HYDATID DISEASE*

S. Kılıçturgay, C İrgil, Y.Özen, C.Aşcı, H.Bilgel

Department of Surgery, Uludağ University School of Medicine, Bursa/Türkiye

A follow-up programme was carried out for 240 patients that were operated in our center due to hydatid disease, but only 131 patients could be contacted and clinically evaluated. A complete physical examination, routine chest x-ray, abdominal ultrasound (US), abdominopelvic CT and indirect hemaglutination for hydatid disease (IHA) were performed on all patients. In this study, the data of 45 patients who were either found to be recurrent after this investigation or were operated in our center initially with the diagnosis of secondary hydatidozis, were analized. The effects of misdiagnosis, mistreatment and inappropriate follow up methods on the reccurence rate have been evaluated.

Of the 45 cases, 6 had recurred twice, 2 had recurred three times. Altogether there were 55 recurrences. 30% dextrose solution was used as a scolosidal agent at the first operation in 51.6% of the case. While in 71.1% of the 45 cases multipl hydatid cysts (HC) were found, 48.8% of these had recurred in the liver as unilo-culer cysts. 24.4% of the first recurrences were free intraabdominal cysts (FIAC). 4 of these were FIAC only and 7 of them were accompaind by liver hydatitozis. Only 3 of these 11 FIAC were diagnosed as FIAC in the first operation. Totally, 13 of the 55 recurrences (23.6%) had an extrahepatic intraabdominal localization. When 131 HD cases were considered, the reccurence rate in solitary cysts was 18.2% and the recurrence rate in multipl cysts was 31.6%. In FIAC this recurrence rate was even higher, being 56.25 %.

9 cases (16.4) were diagnosed in the early 2-6 months period following the first operation. Those were probably not real recurrences and should be accepted as preoperative or intraoperative insuffient evaluation. It was found that US was 20% less efficient than CT in determining the recurrences. In four cases who were not included in these 45, CT and/or US suggested recurrence but diagnostic aspiration excluded this possibility. In this study IHA was also used as a parameter to define recurrence. When titrations which were higher or equal to previous titrations were considered as an index of recurrence, IHA had a 92.3% sensitivity and 46.8% specifity.

In this series, total cystectomy was performed in a very limited number of cases and there was not a cese in which pericystectomy was performed. Therefore, the effects of the application of radical procedure on recurrence could not be evaluated.

In conclusion, careful preoperative and intraoperative evaluation and meticulous surgery will decrease recurrences in hydatid diseases.

### F066

### COMPLIANCE of EXTENDED LIVER RESECTIONS

Kupcsulik P.K.,Pinkola K.Antony P.,Harsanyi L,Flautner L.

1st Dept of Surgery,Semmelweis Univ.of Medicine,Budapest

Between 1989 and 1995 443 patients underwent surgery for liver tumor. 244 interventions were performed for liver malignancy. Preoperative chemoembolisation was applied in 47 cases of liver carcinoma. No direct intraoperative advantage of arterial embolisation was detected compared to the nonembolised patients. In contrast to this 2 year survival of chemoembolised patients is superior to the controls ,however. Intraoperative portal clamping was used in 26 cases. Noninvasive circulatory monitoring by impedance cardiography showed transient, but significant diminution of CO and decrease of PVR. 3 minutes reperfusion after 30 minutes liver ischaemia resulted significant elevation of CO, but complete restitution occured only in the early postoperative phase. Intraoperative blood loss interferes significantly with CO recovery. In 1/26 pts with portal clamping splenic rupture occured. No long lasting splanchnic stasis or any other form of circulatory consequences after portal clamping was observed. Despite to the risk of transient portal devascularization, its combination with ultrasonic dissector significantly diminishes blood requirements during extended liver resections.186 surgical interventions resulted in removal of tumorous mass. Lethality of this group was 3.1. percent respectively.

The use of ultrasonic dissector, its combination with partial vascular liver exclusion diminishes intraoperative blood loss, and promotes extended resection of the liver.

### F067

### RANDOMIZED TRIAL OF END TO END VS SIDE TO SIDE BILE DUCT ANASTOMOSIS AFTER ORTHOTOPIC LIVER TRANSPLANTATION

TR Kurzawinski, L Selves, M Farouk, J Dooley, A Burroughs, KR Rolles, B R Davidson.

Liver Transplantation Unit, Royal Free Hospital and School of Medicine, London UK.

The best method of biliary reconstruction is controversial and much debated technical aspect of orthotopic liver transplantation (OLT). The aim of this study was to compare end to end (E-E) and side (S-S) duct to duct biliary anastomosis after OLT.

Patients undergoing OLT were randomized to receive E-E (n=50) or S-S (n=50) duct to duct biliary anastomosis without T tube. Patients age, sex, graft preservation time and indication for transplantation were similar in both groups. Within 30 days of OLT all patients were scheduled to have endoscopic retrograde cholangiography (ERC). Cholangiographic findings were classified as normal, leak or stricture. Biliary complications were defined as leak or stricture which required endoscopic or surgical treatment. Data were analyzed according to intention to treat.

60 patients received E-E and 40 S-S anastomosis. 10 patients randomized to have S-S had E-E anastomosis done due to shortness of recipient or donor duct. Number of biliary abnormalities on ERC were similar in both groups (E-E 32% vs S-S 30%, NS), leaks (18% vs 16%) and biliary strictures (14% vs 14%). There was no difference in number of biliary complications (E-E 22% vs S-S 22%). Patients and anastomosis survival was similar (median months E-E 11 vs S-S 13.5,NS, E-E 7.5 vs S-S 8.5,NS).

This is the first randomized study of E-E versus S-S biliary reconstruction following OLT and it showed no short term benefit of either technique. We would like to conclude that either of anastomoses could be used according to surgeon preference.

### F068

### THE ROLE OF WHOLE-BODY POSITRON EMISSION TOMOGRAPHY WITH [$18^F$] FLUORODEOXYGLUCOSE IN IDENTIFYING OPERABLE COLORECTAL LIVER METASTASES

D.T.M. Lai, M. Fulham, M.S. Stephen, K.M. Chu, D.M. Sheldon, D.W. Storey, T. Sachinwalla, M.J. Solomon, J.F. Thompson.

**Departments of Upper Gastrointestinal Surgery & PET, Royal Prince Alfred Hospital, Sydney.**

The accuracy of whole-body positron emission tomography (PET) with [$18^F$] fluorodeoxyglucose was compared with conventional radiology in the selection of patients with operable colorectal liver metastases.

Between May 1993 and October 1994, 34 patients with suspected colorectal liver metastases were evaluated. There were 18 males and 16 females with an average age of 62 years, 5 months. Staging by conventional radiology consisted of abdominal CT (n=34), chest Xrays (n=15), and chest CT scans (n=19) to evaluate extrahepatic disease in all patients, and magnetic resonance imaging (n=24) and CTantiography (n=3) of the liver to determine anatomical resectability in 27 patients. PET scan was performed in all patients within 8 weeks of conventional radiology.

PET identified unsuspected extrahepatic malignancy in 11 patients (32%) that was missed by conventional radiology, and these included paraaortic nodal metastases (n=6), pulmonary metastases (n=3) and loco-regional recurrence (n=2). PET consequently affected the clinical management in 10 patients (29%). However, PET did not provide any additional information compared to conventional radiology in the assessment of hepatic metastases per se.

In conclusion, PET improves the preoperative evaluation and selection of patients with isolated colorectal liver metastases for hepatic resection.

### F069

**LONG-TERM RESULTS OF LIVER RESECTION OF HEPATOCELLULAR CARCINOMA IN CIRRHOSIS**

P. Lamoureux, H. Bouzari, C. Vons, L. Capussotti, G. Borgonovo, C. Smadja, D. Franco.
Services de Chirurgie, Hôpital Antoine Béclère, Clamart, Université Paris XI, France, et Ospedale Mauriziano, Turin, Italie.

Liver resection still remains the best treatment for the majority of hepatocellular carcinomas (HCC) in cirrhotic patients. The purpose of this work was to analyze the results of resection in a series of 200 consecutive patients with HCC and cirrhosis operated on from 1992 to 1994. Mean age was 61 years (range : 62 to 78 years). Cirrhosis was of alcoholic origin in 40% of patients. HBV and HCV markers were positive in respectively 23% and 59% of patients. Liver function was good in 79% of patients and most of them (74%) had a single HCC nodule. Nineteen p.cent had major (right or left) and 81% had limited liver resection. Operative mortality was 7.5%. Cumulative 5-year survival was 30% and cumulative 5-year recurrence rate was 69%. The survival rate was significantly higher in Pugh's A (34%) than Pugh's B-C patients (12%n $p<0.001$) and in patients with preoperative serum αFP concentration less than 500 ng/ml (36%) than in those with higher levels of aFP (10%, $p<0.01$). The survival rate was significantly higher in patients without pathological predictive markers of recurrence. Significant pathological markers of recurrence were the absence of free margin of non tumorous parenchyma ($p<0.01$), a tumor above 5 cm ($p<0.02$), the absence of a capsule around the tumor ($p<0.01$) and the presence of satellite nodules and distal portal invasion ($p<0.001$). Postoperative adjuvant arterial chemotherapy slightly decreased the recurrence rate but did not increase survival. This study confirms that liver resection is a good treatment of HCC in cirrhotic patients with a good liver function and a small unique nodule with no biological nor pathological signs of invasiveness.

### F070

**PERCUTANEOUS HEPATIC INTRA-ARTERIAL YTTRIUM-90 MICROSPHERES FOR INOPERABLE HEPATOCELLULAR CARCINOMA**

W.Y. Lau, T.W.T. Leung, S. Ho, M. Chan, P. Johnson, A.K.C. Li
The Joint Liver Cancer Study Group, Prince of Wales Hospital, Shatin, New Territories, Hong Kong.

Our previous studies have shown that radioactive yttrium-90 microspheres, when given in the hepatic artery, concentrated preferentially in liver tumours, giving an average tumour : normal internal radiation dose ratio of 4:1. The need for intraoperative dosimetry by laparotomy has been replaced by the use of simulation technetium-99m macroaggregated albumin scan which have been shown to accurately predict the distribution of the therapeutic yttrium-90 microspheres.

Over a period of 3 years, 89 patients with inoperable hepatocellular carcinoma were treated with hepatic intra-arterial yttrium-90 microspheres given through an angiographic catheter placed percutaneously using the Seldinger technique. The dosage given was calculated using a partition model taking into account of extrahepatic shunting, the tumour : normal ratio and the liver tumour : non tumour volumes measured on computed tomography. There were 76 males and 13 females. The median age was 54 years (range 24 - 85). Sixty-eight patients had primary inoperable tumours, 20 had post-operative recurrences, and 1 had recurrence after lipiodol-$I^{131}$ therapy. The tumour dose given was > 12,000 rads.

All patients had a drop in alpha-fetoprotein of over 80% of the pre-treatment level. Patients with pre-treatment normal alpha-fetoprotein were monitored with ferritin levels which showed a similar drop after therapy. The median survival was 8 months (median survival for systemic chemotherapy in previous studies was 10 weeks). The patient with the longest survival was 25.9 months after treatment. He is still alive.

This study suggests that radioactive yttrium-90 microspheres may be of use in patients with inoperable hepatocellular carcinoma. A randomised study to further evaluate this treatment is indicated.

### F071

**HEPATOCELLULAR CARCINOMA PRESENTING AS OBSTRUCTIVE JAUNDICE**

W.Y. Lau, K.L. Leung, T.W.T. Leung, M. Chan, S.C.H. Yu, A.K.C. Li
The Joint Liver Cancer Study Group, Prince of Wales Hospital, Shatin, New Territories, Hong Kong.

Obstructive jaundice as the main presenting feature of hepatocellular carcinoma is uncommon.

In a period of 11¼ years, 48 of 2,037 patients with hepatocellular carcinoma seen in our hospital presented with obstructive jaundice (2.4%). There were 39 men and 9 women. The mean age was 54 years (s.d. - 12.9). Obstructive jaundice was diagnosed by blood tests, ultrasound and cholangiography (ERCP or PTC). Hepatocellular carcinoma was diagnosed by either histological proof or in patients with space occupying lesions in the liver with serum alpha fetoprotein of over 500 ng/ml.

Ultrasound showed dilated intrahepatic biliary system in all 48 patients. The pathologies causing obstructive jaundice included tumour casts (n - 10), free tumour fragments in the extrahepatic biliary systems (n - 7), diffuse tumour involvement of intrahepatic ducts (n - 28), extrahepatic bile duct obstruction by enlarged porta hepatis lymph nodes (n - 3) and haemobilia (n - 8). Some patients had more than one pathology.

Patients with potentially operable liver tumours were further evaluated with computed tomography and hepatic angiography. Preoperative investigations revealed 37 patients to have inoperable tumours and they were treated with endoscopic stent (n - 24), percutaneous stent (n - 6), stent put in by combined endoscopic / percutaneous approach (n - 2) or supportive treatment only (n - 5). Eleven patients underwent laparotomy and "curative resection" was possible in 9, while the other 2 had surgical intubation only. With proper management the survival of patients with hepatocellular carcinoma with obstructive jaundice was similar to those without jaundice. Four patients who had "curative" liver resection were still alive, 3 had no evidence of disease at 11.6, 13.7 and 99.2 months after operation and one had recurrence and is still alive 20.5 months after surgery.

Patients with hepatocellular carcinoma with obstructive jaundice should be treated actively. Good palliation, and occasional cure, are possible.

### F072

**LIVER RESECTIONS : A SINGLE INSTITUTIONAL EXPERIENCE OF 612 PATIENTS**

B. Launois, M.A.C. Machado, C. Stasik, G. Spiliopoulos, B. Chareton, JP. Campion
Department of Surgery, University of Rennes - France.

Between 1972 and 1995, 612 patients underwent laparotomy in view of liver resection. There were 400 males and 212 females with a mean age of 56.4 ± 14.3 years. Indication for surgery included hepatocellular carcinoma (n=207), other primary liver tumors (n=47), liver metastases (n=221), benign liver tumors (n=77), liver trauma (n=21), and others (n=39). Variables were compared using $\chi^2$ test or Fisher's exact test. Survival was calculated according to the Kaplan-Meier method and survival curves were compared using the Logrank test.
RESULTS : 205 patients underwent major liver resections comprising at least 3 segments. 128 patients underwent right hepatectomy, 63 patients underwent left hepatectomy and 14 patients had 3 or 4 non-contiguous segments removed. 199 patients had 1 or 2 segments resected and 68 had non-anatomical resections. 131 exploratory laparotomies were performed for unresectable tumors. Overall resectability rate was 78.6 %. Operative mortality and morbidity was 6% and 21% respectively. 5-year survival following resection of colorectal metastases, hepatocellular carcinoma developed on cirrhotic and non-cirrhotic livers was 24.6%, 21.7% and 28.6% respectively (p= NS) When results of our early experience were compared to those obtained after 1990 there was a significant decrease in operative mortality and morbidity. Five year survival was also improved. ($p < 0.05$).

### F073

### IS LIVER TRANSPLANTATION (OLT) A RATIONAL APPROACH FOR NEUROENDOCRINE METASTASES (NEM) ? REPORT OF A FRENCH MULTICENTRIC STUDY OF 31 CASES.

Y.P. Le Treut*, J.R. Delpero*, D. Houssin, D. Cherqui, P. Segol, G. Mantion, L. Hannoun, G. Benhamou, B. Launois, O. Boillot, J. Domergue, H. Bismuth, (*) Hôpital la Conception, 147 Bd Baille, 13005 Marseille and ACHBT.

Indications of OLT for NEM are still a matter of controversy, as far as only limited data are available. This retrospective study involves 31 cases of OLT collected among 11 centers from 1989 to 1994. • There were 17 males and 14 females, age 45 ± 9 years (M ± SD). Primary tumor site was pancreatic in 17 cases, ileal (7), bronchial (3), gastric (2) colonic, rectal or lymphatic (1 each). Tumor varieties were carcinoid (15 cases), gastrinoma (7), glucagonoma (1), nonfunctioning (8). Hormonal-related symptoms were present in 17 patients. Seventeen patients (54 %) underwent excision of the primary tumor and 23 (74 %) received chemotherapy before OLT. There was a 30 ± 32 months interval (median : 19, range : 2-120) between the diagnosis of NEM and OLT ; it was 31 ± 34 months for carcinoid and 29 ± 30 months for others NEM. Fourteen patients had not undergone resection of primary tumor at the time of OLT : simultaneous removal of the primary tumor and NEM was performed in 11, one by ileal resection, 3 by Whipple resection and 7 by upper abdominal exenteration (with synchronous pancreatic transplant in 3 cases). In the 3 remaining cases, the site of the primary tumor was discovered only after OLT. • Six patients (19 %) died post-operatively : 4 out of 7 after "cluster resection" and all 3 after composite liver and pancreatic graft. Major surgical complications occured in half of the patients. Among the 25 survivors, 4 died from late complications of the procedure, without evidence of recurrent disease (4, 5, 8 and 10 months after OLT), and 8 patients died of recurence (2 to 41 months after OLT). Thirteen patients are still alive. Overall actuarial survival rates were 58 % at one year and 36 % at 5 years (5 patients). Theses rates were 80 %, 80 % and 69 % at 1, 3 and 5 years respectively for the 15 patients with carcinoid tumors, versus 38 %, 15 % and 0 % respectively for the 16 patients with others NEM. • Theses results suggest that OLT can achieve benefit in patients with carcinoid metastases, but not in patients with other type of NEM.

### F074

Authors: LIEDKE, M; ROGIERS, X; BRUNKEN, C; MALAGO; FRILLING, A; BROELSCH, CE.

### The efficacy of hepatic resection for more than 4 liver metastases from colorectal carcinoma?

During the last decade, surgeons have taken a more aggressive attitude in the treatment of primary or secondary liver tumors, particulary those from colorectal primaries. Several authors have demonstrated, that resection of a single metastastic lesion is benificial for the patient.

From jan. 1986 til dec. 1993, we underwent by 142 patients liverresections for hepatic metastases of colorectal cancer. The patients devided in three groups

(group 1: one metastase n= 61, group 2: 2-4 metastases n=48, group3: more than 4 metastases n=23), which were analysed retrospectivly.

The overall hospital mortality was 3,52%. The results are illustrated in table 1.

Tab.1:

| Group | n | R0/R1/R2 | hosp. mort. | 1 year survival | 5 year survival | 1 year survival R0 | 5 year survival R0 | 5 year survival R1 |
|---|---|---|---|---|---|---|---|---|
| 1 | 61 | 46/5/10 | 1.6% | 86% | 21.2% | 73% | 11.1% | 8.1% |
| 2 | 48 | 28/3/17 | 2.0% | 72.9% | 8.3% | 47.9% | 8.3% | 4.1% |
| 3 | 23 | 3/1/19 | 13.04% | 30.4% | 0% | 17.3% | 0% | 13% |
| | 142 | | 3.52% | | | | | |

Our results represent, that the most benifit have patients with R0 resection. The number of metastases in the liver is a deceiding factor for the prognoses and the survival benifit patients will have from the resection. The group 3 had the highest hospital mortality and only Ro resected patients will have a benifit from the liverresection.

### F075

### DEARTERIALIZATION THERAPY OF LIVER CANCER-INSTITUTIONAL EXPERIENCE

G. Lindell, B. Ohlsson, B. Jeppsson, B. Persson, S. Bengmark, K-G. Tranberg

Department of Surgery, Lund University, Lund, Sweden

Normal hepatic parenchyma is supplied by portal and arterial blood. In contrast, overt liver tumours have a predominant arterial supply, which is the basis for treating hepatic tumour with arterial ischemia. This report reviews our total experience for hepatic non-endocrine tumours during 1971-1995. The material consists of 80 patients (aged 59 (37-77) years) with primary liver cancer (n=15), colorectal liver metastases (n=61) or other hepatic malignancies (n=4) and includes all consecutive developments: complete (permanent) dearterialization (n=12), intermittent (16 h) dearterialization (n=26) and repeat transient dearterialization (1-2 h once or twice daily using an implantable occluder) (n=42). The liver tumours were bilateral in 90 % of the patients and occupied 25-75 % of the liver in 2/3 of the patients. Extrahepatic tumour was present in 29 % of the patients. 51 patients received chemotherapy, mainly 5-fluorouracil and leucovorine. Patients with primary liver cancer had a median survival (after start of treatment) of 9 months and a 5-year survival rate of 7 %; corresponding figures for patients with colorectal liver cancer were 11 months and 2 %. There were no obvious differences in survival between permanent (n=8), intermittent (n=24) and repeat transient (n=29) dearterialization for colorectal liver cancer (median survivals were 10, 11 and 13 months, respectively). Liver tumour volume and a history of weight loss varied negatively with survival in patients with colorectal liver cancer. Three patients (4 %) died in the postoperative phase, and 5 patients (6 %) suffered from hepatic or intraabdominal abscess formation. It is concluded that hepatic dearterialization is of doubtful benefit but may be considered in carefully selected cases with tumour growth confined to the liver.

### F076

### BILIARY COMPLICATIONS AFTER HEPATIC RESECTION

C.M. Lo, S.T. Fan, E.C.S. Lai, C.L. Liu, J. Wong

Department of Surgery, The University of Hong Kong, Queen Mary Hospital, Hong Kong.

A retrospective study was conducted to determine the incidence, risk factors and consequence of biliary complications after hepatic resection and to evaluate the management. Between January 1989 and October 1995, 347 hepatic resections were performed at the Department of Surgery, The University of Hong Kong at Queen Mary Hospital. There were 251 male and 96 female patients with a mean age of 53.2 years (range 2-86). Major hepatic resection (defined as resection of 3 or more segments according to Couinaud's description) was performed in 229 (66%) cases and the liver was not cirrhotic in 169 (49%) patients. Twenty-eight (8.1%) patients developed biliary complications. The biliary complication rate was 6.5% from 1993 to 1995 and 9.5% from 1989 to 1992 (p=0.31). The hospital mortality rates for the 2 periods were 4.8% and 12.3% respectively (p=0.01). The clinical presentation included biliocutaneous fistula (n=18), peritonitis (n=3), intraabdominal abscess (n=6) or intraoperative bile duct injury (n=1). The site of leakage was from the raw surface (n=2), right/left hepatic duct stump (n=6), common hepatic duct (n=3), bilioenteric anastomosis (n=4), T-tube insertion site (n=1) or unidentified (n=12). Logistic regression analysis revealed that the risk of biliary complication was increased by older age, higher preoperative white cell count, longer operation time and a higher serum total bilirubin 3 days after operation. Ten of 28 (35.7%) patients with biliary complications died in hospital, most often due to intraabdominal sepsis. Initial treatment consisted of surgery (n=9; 6 died), percutaneous drainage (n=9; 3 died), endoscopic papillotomy ± stent (n=4; 1 died) and conservative (n=6; nil died). The development of biliary complication was associated with a higher incidence of liver failure and prolonged hospital stay. We conclude that biliary complication is a frequent cause of morbidity and mortality after hepatic resection and may be avoided by shortening of operation time. A high bilirubin level 3 days after hepatic resection should arouse suspicion and facilitate early detection. Reoperation carries a high mortality and non-operative management is effective in selected patients.

### F077

### "PIGGY-BACK" IMPLANTATION WITH TEMPORARY PORTACAVAL SHUNT- A SUPERIOR TECHNIQUE FOR LIVER TRANSPLANTATION

J.A. Lowell, S. Shenoy, S. Dolan, T.K. Howard. Section of Transplantation, Washington University School of Medicine, St. Louis, MO, USA

Liver transplantation (OLTx) is most commonly performed with vena caval replacement.Veno-venous bypass (VVB) during the anhepatic phase is used routinely, selectively, or not at all. Without VVB, significant volume loading is required; there maybe hemodynamic instability, and bowel edema maybe problematic. By preserving the vena cava, and anastomosing the donor vena cava to the recipient's hepatic veins ("piggy-back"), the complications associated with vena caval clamping and replacement are obviated. Portal vein (PV) decompression is accomplished by creating a temporary portacaval shunt (PCS) during the native hepatectomy, which is then ligated and taken down during the graft implantation. **Results:** We have used this technique in 58 consecutive adult OLTx recipients. The etiology for liver disease included: Hepatitis C (n=14), ETOH (n=11), Cryptogenic (n=6), PBC (n=7), PSC (n=6), and other (n=14). 2 patients had previously been transplanted. 10 patients had previous TIPSS procedures. 2 patients required PV extension grafts because the TIPSS was extrahepatic. 1 patient had a large spontaneous porta-systemic shunt and did not require a PCS. All patients (including 1 patient transplanted for fulminant hepatic failure) demonstrated stable hemodynamics during hepatectomy and implantation. Median operative PRBC replacement was 2.0 units (range 0-19). 18 patients (31%) received no PRBC transfusions, and 34 patients (58%) received 2 units or less. No complications attributable to this technique were noted. 55/58 patients are currently alive and well. VVB costs of approximately $4,000 per patients were saved using this technique. **Conclusions:** This technique of OLTx is safe, and offers all of the benefits of VVB without incurring its risks and costs. Early creation of the temporary PCS may be especially useful in the difficult hepatectomy.

### F078

### LIVER TRANSPLANTATION AFTER JEJUNO-ILEAL BYPASS

J.A. Lowell*, S. Shenoy*, S. Dolan*, M. Peters#, T.K. Howard*
Departments of Surgery* and Medicine#, Washington University School of Medicine, St. Louis, MO, USA

Jejuno-ileal (JI) bypass was developed as a therapy for morbid obesity in the late 1960's, but has since been abandoned because of a high rate of complications, including cirrhosis (incidence 7-10%). The need for liver transplantation (OLTx) after JI bypass has been infrequent, with only 4 previous patients reported in the literature. However, as the time to the development of symptomatic end-stage liver disease (ESLD) after JI bypass maybe quite long (25 years or more), the incidence of patients who will require OLTx may now only be increasing. We retrospectively reviewed our experience with JI bypass and OLTx in 280 adult patients since 1985. **Results:** 3 patients (2 females) have undergone OLTx for decompensated ESLD due to steatohepatitis after JI bypass, all within the last 48 months. One patient had concomitant alcoholic liver disease. The mean age of this group was 51 years (range 46-60). The mean duration from time to JI bypass to OLTx was 24 years (range 23-35). Two of three patients had other complications related to the JI bypass (renal and biliary stones). One patient had the JI bypass taken-down prior to OLTx, which precipitated acute liver and renal failure, necessitating urgent OLTx. One patient had the JI bypass taken-down at the time of OLTx. The third patient continues to have the JI bypass and is followed closely with monthly liver function studies and yearly liver biopsies, and to date (2 years post-OLTx) has not demonstrated any complications attributable to the JI bypass. The patient who underwent concomitant OLTx and JI bypass take-down, has developed recurrent obesity, while the other two patients have not. **Conclusions:** The incidence of patients who require OLTx after JI bypass maybe on the increase. Takedown of the JI bypass may precipitate acute liver failure in the cirrhotic patient, and lead to the need for urgent OLTx. JI bypass reversal should be accomplished either at the time of OLTx, or if signs of liver dysfunction occur after OLTx. OLTx recipients maybe at risk for recurrent obesity after takedown of the JI bypass.

### F079

### CHANGES OF LIVER PERFUSION IN PRESENCE OF COLORECTAL CANCER METASTASES

*L. Lupo, GL DiGiulio*, A. Panebianco, G. Angelelli*, V. Memeo*

Ist. di Clinica Chirugica and *Radiologia, University of Bari, Italy

Changes in liver blood flow were observed in presence of overt metastases by invasive and non invasive methods.
Doppler Ultrasound is a non invasive method to quantify liver blood flow by measuring the mean velocity ( Vm ) and the cross-section area ( A ) of the hepatic artery and the portal vein.
To assess the relationship between blood liver perfusion and the presence and extent of liver metastases, the Hepatic Perfusion Index (HPI), defined as the Arterial/Total blood flow ratio, was evaluated by Colour Doppler US in 20 patients with US detected hepatic colorectal cancer metastasis (MET), in 33 patients with confined colorectal cancer (CA) and in 24 control subjects (CON) matched for age and weight.
Results HPI resulted significantly higher in MET than in CA and CON: 0.25 ( SE 0.04 ) vs 0.17 ( 0.02 ) vs 0.10 ( 0.025 ), $p<0.0001$ ( Wilcoxon test ) by increase of arterial flow ( + 32 %, $p<0.001$) and reduction of portal flow ( -18 %, $p<0.05$ ). By uni- and multi-variate analysis HPI resulted correlated to the number of lesions: >3 metastases vs < 3: 0.30 vs 0.21, $p< 0.05$ and $R^2= 0.29$, $p<0.001$.
In conclusion, HPI correlated with the presence and number of liver metastases in patients with colo-rectal cancer, as effect of increased arterial blood supply and reduced portal flow.
HPI could be proposed as a test for liver metastasis screening.

### F080

### PREDICTIVE FACTORS OF SURVIVAL FOLLOWING SURGERY FOR COLORECTAL LIVER METASTASES

M.A.C. Machado, C. Stasik, G. Spiliopoulos, B. Chareton, JP. Campion, B. Launois
Department of Surgery, University of Rennes - France.

Nearly 40% of patients with colorectal malignancies will develop liver metastases in the course of their illness. The aim of this retrospective study was to determine predictive factors of long-term survival in patients treated surgically.

Between 1976 and 1995, 166 patients underwent surgery for colorectal liver metastases. There were 98 males and 68 females with a mean age of 59.8 years (range: 31-80). 123 metastases were metachronous and 43 were synchronous of the primary tumor. Prognostic variables were analyzed using the $\chi^2$ test and Fisher's exact test. Survival was calculated according to the Kaplan-Meier method and survival curves were compared using the Logrank test.

100 patients underwent major liver resections comprising at least 2 liver segments: right hepatectomy (n=48), left hepatectomy (n=17), resection of 2,3 or 4 non-contiguous segments (n=35). Minor resections and non-anatomical resections were performed in 22 patients. The resectability rate was 86.5%. Operative mortality and morbidity was 6.2% and 28% respectively. Mean hospital stay for survivors was 16 days. 1,3 and 5 year survival rate was 75%, 32.3% and 24.6% respectively. Predictive factors of long term survival included size of metastases, number of metastases, staging of the primary tumor and resective margins. CONCLUSION: Exploratory laparotomy is indicated in most patients with colorectal liver metastases. Resectability is best assessed using intraoperative ultrasound. Resection should only be undertaken when complete eradication of the disease is possible.

### F081

**PROGNOSTIC VARIABLES INFLUENCING LONG TERM RESULTS OF RESECTIVE SURGERY FOR HEPATOCELLULAR CARCINOMA**

M.A.C. Machado, C. Stasik, G. Spiliopoulos, B. Chareton, JP. Campion, B. Launois
Department of Surgery, University of Rennes - France.

Worldwide hepatocellular carcinoma is among the most common malignancies. The aim of the present study was to identify prognostic variables having an influence on long-term survival.

Between 1974 and 1995, 207 patients underwent surgery for hepatocellular carcinoma. There were 185 males and 22 females with a mean age of 60.1 ± 11.7 years. The liver was cirrhotic in 149 patients and non-cirrhotic in 58 patients. Prognostic variables were analyzed using the $\chi^2$ test and Fisher's exact test. Survival was calculated according to the Kaplan-Meier method and survival curves were compared using the Logrank test. 37 patients (18%) underwent major liver resection comprising 3 or more segments. There were 25 right hepatectomies, 12 left hepatectomies, 25 left lobectomies, 6 right lateral segmentectomies, 57 of 1 or 2 segments and 11 non anatomical resections. 71 patients had exploratory laparotomy only. The resectability rate was 65.7% Operative mortality for patients resected was 8.4%, 9.5% for those with cirrhosis versus 4.8% for patients with non-cirrhotic livers (p= NS.). Morbidity was 34.7%. 1, 3 and 5 year survival for cirrhotic patients was 70%, 43.1% and 21.7% respectively. 1, 3 and 5 year survival for non-cirrhotic patients was 81%, 50% and 28.6% respectively (p= NS). Prognostic variables included tumor size, number of tumor nodules, serum alfafetoprotein levels and resective margins.

CONCLUSIONS : Because of the high rates of early recurrence associated with liver transplantation and the palliative nature of intraarterial/systemic chemotherapy, resection remains the mainstay of therapy for resectable hepatocellular carcinoma.

### F082

**LIVER RESECTION : THE EASTERN EXPERIENCE**
**---- SPECIAL COMPARISON WITH ALCOHOL INJECTION ----**

M. Makuuchi
Second Department of Surgery, University of Tokyo, Tokyo, Japan

Since October 1979, we have laparotomized 1391 patients for hepatectomy. Hepatic resection was undergone 1274 (91.6%). among them, 829 had hepatocellular carcinoma and hepatectomy was carried out 777 (93.7%). Five hundred ninety five patients had the largest tumor no more than 5 cm in diameter. Hepatic resection was carried out in 572 (96.1%).

Resection area of the liver was decided depending on the indocyanine green retention rate at 15 min. After laparotomy, intraoperative ultrasonography was performed for evaluation of extent of HCC in the liver and indication of hepatic resection and operative procedure were finally decided. Vascular occlusion techniques such as selective vascular occlusion or Pringle maneuver were routinely applied. Division of the liver was proceeded under the guide of ultrasound.

Survival rate of all the series at five years was 40 %. Survival rate in patients received systematic subsegmentectomy was significantly better than that in limited resection. The most significant prognostic factor was year trends. After 1985, 5-year survival reached to 65 %. Repeated resections were one of the important factor to improve long term survival.

Since 1990, percutaneous ethanol injection therapy (PEIT) has been generally performed. However our recent group study clearly suggested that first choice of treatment in small HCC was surgery, not PEIT. Even in early HCC, hepatic resection showed much better survival than PEIT when pathological diagnosis was made by 6 pathologists specialized in liver tumors. Therefore, indication of PEIT in Japan may be limited in near future.

### F083

HOW IS POSSIBLE A CERTAIN PREOPERATIVE DIAGNOSIS IN THE MANAGEMENT OF BENIGN TUMORS OF THE LIVER?

G. Mangiante*, N. Nicoli*, F. Nifosi*, GC. Mansueto§, P.P. Aurola*, L. Bortolasi*, C. Iacono*, A. Acerbi*, G. Serio*
Departments of Surgery * and Radiology §, University of Verona, Verona, Italy

A certain preoperative diagnosis is mandatory in the management of benign tumors of the liver as hemangioma (HMG), focal nodular hyperplasia (FNH) and hepatocellular adenoma (HCA); periodic follow-up is the best therapeutic choice. In order to evaluate diagnostic and therapeutic work-up we review retrospectively the results of biochemical data and diagnostic imaging procedures as US, CT, abdominal angiography (ANG), magnetic resonance (MRI), tecnetium colloid scintigraphy (TCS), DISIDA scintigraphy (DISIDAS) of 90 pts with HMG, 50 pts with FNH and 12 pts with HCA observed from 1975 to 1995. Sensitivity and specificity of each test for benign lesions were determined through the ratio of the true and false positive rate and the true and false negative rate at radiologic findings of the total number of hepatic neoplasms, considering not only benign tumors but also malignant lesions as hepatocellular carcinoma, colangiocellular carcinoma and metastases. All the results were confirmed by hystologic diagnosis. In the pts with HMG, MRI and ANG reached the best sensitivity and specificity (respectively 96% and 100%), while specificity of US and CT were respectively 66% and 99.3%. In the pts with FNH US had a low sensitivity (66%) but a very high specificity (98%), while CT scan showed sensitivity 88% and specificity 99%; in this group of pts the best results were reached by TCS (sensitivity 94% and specificity 100%) and DISIDAS (sensitivity 91%, specificity 100%). In the pts with HCA the best results were achieved with ANG (sensitivity 75%, specificity 98%), while US showed the worst sensitivity (only 7%); in this case the most common differential diagnosis was with malignant form. The contribution of imaging techniques is able to obtain a quite sure diagnosis of HMG and FNH: only symptomatic cases have to be resected. The diagnostic doubt in HCA is still too high and these lesions have always to be resected.

### F084

**CLINICAL UTITILITY OF INTEGRATION OF IMAGING METHODS IN THE DIAGNOSIS OF FOCAL LIVER LESIONS**

G. Maresca, A. De Franco, R. Manfredi, A.R Cotroneo, G.B. Doglietto*, P. Marano, F. Crucitti*
Department of Radiology and Surgery*, Università Cattolica del Sacro Cuore, Policlinico "A. Gemelli", Rome, Italy.

PURPOSE: to evaluate if the imaging methods integration, echo-color-Doppler (ECD), computed tomography (CT) and magnetic resonance imaging (MRI) enhances the results of the single method in detecting and characterizing focal liver lesions

MATERIALS AND METHODS: 35 patients with focal liver lesions underwent ECD, CT and MRI. Semeiologic criteria were determined for each method, and a first diagnostic evaluation is expressed. Subsequently all three diagnostic methods are evaluated together, and a final, integrated evaluation is expressed regarding the number, the site and the type of the lesions.

RESULTS:

| SENSITIVITY | NUMBER OF LESIONS (total 124) | |
|---|---|---|
| ECD | 78 (62%) | |
| TC | 92 (74%) | |
| RM | 90 (72%) | |
| SPECIFICITY | BENIGN LESIONS | MALIGNANT LESIONS |
| ECD | 65% | 56% |
| TC | 71% | 75% |
| RM | 75% | 68% |
| INTEGRATION | SENSITIVITY | SPECIFICITY |
| ECD + CT | 82% | 87% |
| ECD + MRI | 80% | 89% |
| ECD + CT + MRI | 95% | 96% |

CONCLUSION: The imaging integration is very helpful in the preoperative staging in oncologic patients (number, and site of the lesions), and enables a better tissue characterization for a variety of signs.

F085

**MANAGEMENT OF BLEEDING FROM GASTRIC VARICES**

S.K. Mathur, S. Shah, L. Kakkar, M. Sanzgiri.

G.I. Surgical Services, Seth G.S. Medical College and K.E.M. Hospital, Bombay, India.

**The present study was done to assess the efficacy of various therapeutic modalities in the management of gastric variceal bleeding. During the last 10 years, 60 patients presented with bleeding from gastric varices (GV). The cause of portal hypertension was cirrhosis in 23, extrahepatic portal venous obstruction (EHPVO) in 22, non cirrhotic portal fibrosis in 13 and isolated splenic vein thrombosis in 2 patients. Site of bleeding was lesser curve GV (LCGV)in 16 and fundal GV (FGV) in 44 patients (isolated FGV-13 and FGV + esophageal varices in 31). Endoscopic sclerotherapy (EST) was initially successful in controlling bleeding from LCGV in all 16 patients, 15 continued on chronic EST and one required elective surgery. Of 44 patients with bleed from FGV, initial control could be achieved with balloon tamponade in 12 of 25 (48%) patients, with EST in 2 of 15 patients (13%) and with injection of glue in 3 of 4 patients (75%). Five of these 17 responders were continued on chronic EST, 2 patients were lost to follow up and 10 patients were subjected to elective modified Sugiura's surgery. Emergency surgery was performed in 27 patients (modified Sugiura's operation-25 and porto-caval shunt -2) after other therapies had failed or in those who had rebled after initial control. Results: Operative mortality was nil for the elective group, 36% for emergency devascularisation and 50% for shunt surgery.Mortality was 63% for cirrhotics (all Childs score C), 25% for EHPVO and 25% for NCPF. There were 2 late deaths due to liver cell failure in the cirrhotic patients. Twenty five patients are alive and free from rebleed. In conclusion, while LCGV can be successfully managed with EST, Fundal varices respond poorly to conservative therapy. Surgery in the form of gastroesophageal devascularisation with stapling is highly successful in controlling active bleeding as well as for long term prevention of rebleed from fundal varices and should be the therapy of choice for these patients.**

F086

USEFULNESS OF ANGIO CT FOR DIAGNOSIS OF HEPATOCELLULAR CARCINOMA

S.Mikami, H.Kinoshita, K.Hirohashi, S.Kubo, H.Tanaka, T.Tsukamoto, T.Shuto, C.Sakata,
Second Department of Surgery, Osaka City University Medical School, Osaka, Japan

In the last seven years, 67 new nodules of hepatocelluer carcinoma (HCC) were detected in non-tumor specimens which had been resected from in 43 of 213 patient of HCC at our institution. Despite improvement of several imaging modalities, many hepatic nodules not detected preoperatively are thought to exist in resected specimens. This study was performed to evaluate the detectability of hepatic nodules on computed tomography during angiography (angio CT) compared other modalities including digital subtraction angiography (DSA), computed tomography with other methods (conventional CT), magnetic resonance imaging (MRI) and ultrasonography (US). Forty-seven patients with HCC underwent hepatic resection or liver biopsy after angio CT. In these 47 patients, seventy-three nodules were detected in resected specimens or in needle biopsy specimens. These nodules included 54 nodules of HCC, 3 of early hepatocellular carcinoma (eHCC), 5 of adenomatous hyperplasia (AH), 5 of hemangioma, and 6 of other benign lesions. The rate of detection of all hepatic nodules was 91.8% (67 nodules out of 73; 67/73) with angio CT, 58.9% (43/73) with DSA, 74% (54/73) with conventional CT, 72.5% (37/51) with MRI and 80.8% (59/73) with US. The rate of detection of eHCC and AH was 50% (4/8) with angio CT, 12.5% (1/8) with DSA and conventional CT, and 25% (2/8) with MRI and US. In conclusion, in this study angio CT could detect hepatic nodules more frequently than other imaging modalities. However, thirteen nodules in 9 cases detected preoperatively with angio CT were not detected with intraoperative US. This suggests that it is important to follow these nodules carefully.

F087

ARE HILLAR HYDATID CYSTS A TREATH TO INTRAHEPATIC BILE DUCT INTEGRITY

Milicevic MN, Jekic IM, Bulajic PD, Zuvela M, Radovic Z, Djukic V, Kovačević N.
The First Surgical Clinic, Clinical Center of Serbia, Belgrade, Yugoslavia

**INTRODUCTION** : Intrahepatic bile duct ( IHBD ) - hydatid cyst ( HC ) communication is a common complication of liver hydatidosis. Aim of the study was to analyze effects of site, size and number of cysts on the incidence of IHBD-HC communication.
**MATERIAL AND METHODS** : There were 132 ( 22,3% ) patients with IHBD - HC communication out of a total of 593 operated for hydatid disease. Male / female ratio was approx. 1:1 ( 51,5 : 48,5% ), age ranging from 18 to 79, mean age 40,4 yrs. Only 22 pts. ( 16,66 % ) out of 132 presented with symptoms and signs of bile duct obstruction. Intraoperative diagnostics consisted of IO cholangiography and IO US when necessary. Out of 84 pts. with hillar localization of the cyst 28 pts.(33.3%) had IHBD-HC communication while only 104 pts.(20,4%) of 509 pts. with non-hillar HC communication had communications. Solitary cysts were bigger in the group with hillar localization, in average 13,9 cm compared to 10,3 cm. Management of IHBD - HC communication depends on the site of the communication, which can be accurately verified by IO cholangiography. It was possible to suture the communication in 72 pts. ( 70,5%). Communication with major ducts demands more sophisticated procedures.
**DISCUSSION** Features of the cyst depend of it's size, site, viability and infection status. It has been demonstrated that the probability of IHBD-HC communication increases with hillar location, size and multiplicity of cysts. The communication is not always apparent during operation and should be carefully searched for especially in bile stained cysts. IO cholangiography and IO US are useful tools in determining operative strategy.
**CONCLUSION** Hillar hydatid cysts frequently communicate with bile ducts. Due to the proximity of major bile ducts visualization of the communication site is useful in planing surgical strategy.

F088

RECURRENCE PATTERN OF HEPATOCELLULAR CARCINOMA AFTER CURATIVE RESECTION

K. Nagahori, M. Matsuda, J. Okuda, M.Yamamoto,Y. Matsumoto

First Department of Surgery, Yamanashi Medical University, Yamanashi, Japan

One cause of high incidence of intrahepatic recurrence after curative operation for hepatocellular carcinoma (HCC) might be the multicentric occurrence (MC) of HCC different from the primary tumor. In this study we tried to clarify the characteristics of MC determined by histopathological and molecular biological analysis. <Patients and methods> We analyzed the recurrence pattern of 106 patients who underwent curative primary resection between October, 1983 and December, 1994 in our hospital. While the cumulative survival rates 2 and 5 years after the operation were 82 and 64%, respectively, the corresponding cumulative disease-free survival rates after the operation were 56 and 31 %, respectively. <Results> Recurrence was seen in 45 patients. Clinicopathologic variables in the recurrence group were compared with those in the disease-free group. The sex of the patients, HBs Ag, HCV-Ab, tumor staging, tumor size, extent of hepatic resection did not affect the incidence of recurrence, but recurrence occurred less frequently in the patients with noncirrhotic liver (p=0.004) and in the patients with multiple HCC (p=0.0008). Among 41 patients with intrahepatic recurrence, one to three nodules in the same lobe as the primary tumor were seen in 9, those in a different lobe in 12, multiple nodules in 18 and marginal recurrences in 2. In 29 patients whose recurrent HCC were analyzed for clonality, 14 showed MC of HCC and 10 metastatic HCC. Repeat resection was performed in 10 patients with the form of one to three nodular recurrence and in 8 the recurrences were MC. The survival rate after the re-operation was the same as that after the first resection. <Conclusion> It seems important to detect MC, especially in cirrhotic liver, in the early stage after the first operation and perform radical treatments in order to prolong the survival.

### F089

### ROUXLOOPRECONSTRUCTION(RLR) ASPRIMARYTREATMENTFOR BILIARY COMPLICATIONS FOLLOWING ORTHOTOPIC LIVER TRANSPLANTATION(OLT)

A Nandy, N Doctor, L Horgan, A Burroughs, B Davidson, K Rolles
Hepatobiliary and Liver Transplantation Unit, Royal Free Hospital School of Medicine, London, UK

RLR is one method of treating biliary complications after OLT. This study looks at the morbidity and outcome of 24 consecutive RLR's performed between 23rd of July 93 and 26th of October 95 as a primary procedure for biliary complications following OLT. There were 17 males and 7 females with mean of 42.9 years. All had duct to duct anastomoses at OLT. Nine patients presented with a biliary leak, 13 with a stricture, 1 with a biliary cast and 1 with biliary sludge. All were associated with sepsis. 5 patients had associated hepatic artery thrombosis. Patients who had biliary leak had RLR at a mean peroid of 2.7 weeks after OLT. Patients presenting with strictures had a mean peroid of 13.5months between RLR and OLT. Preoperatively 11 patients had nasobiliary and 2 had percutaneous transhepatic drainage for a mean period of 5 days. The post-operative hospital stay ranged from 5 days to 3 weeks (mean 10.5 days). Early complications were 3 pneumonias, 1 renal impairment, 1 haematemesis and 1 colitis all which resolved with treatment. Of these, only the 3 pneumonias were directly related to the procedure. The mean period of follow up was 9.2 months. There were no early or late anastomotic complications. 1 patient developed a hilar stricture and another a left hepatic duct stricture which required RLR revision. There were 4 unrelated deaths. RLR is a safe and effective procedure when performed as a primary procedure for biliary complications following OLT.

### F090

### REDUCTION OF RAT HEPATIC ISCHEMIA/REPERFUSION INJURY BY INTERMITTENT ANOXIA

B. Nardo*, A. Gasbarrini^, M. Simoncini^, S. Cuccomarino*, S. De Notariis^, A. Recordare*, P. Caraceni^, F. Trevisani^, A. Mazziotti*, M. Bernardi^, A. Cavallari*
Clinica Chirurgica II* and Patologia Medica^, University of Bologna, Italy

**INTRODUCTION.** The most used technique to control intraoperatory bleeding during hepatoresection is the "Pringle manouvre" (temporary clamping of the hepatic pedicle). In spite of advantages, reperfusion injury represents an important problem in case of prolonged clamping. Intermittent clamping has been recently proposed as a method to reduce organ injury. In this study, we compared survival, hepatic adenosine triphosphate (ATP) level and lipid peroxidation (LP) in rats exposed to intermittent *vs* continuous ischemia of the liver. **MATERIALS AND METHODS.** Liver ischemia was induced in male Wistar rats (weight range 200-250 g), by clam-ping the appropriate branches of the portal vein and hepatic artery of the left lateral and medial lobes of the liver with noncrushing microvascular clamps. The times of ischemia were 60, 90 and 120 min intermittently (periods of 30 min of ischemia followed by 10 min of reperfusion) or continuously. Partial ischemia was performed to avoid splanchnic congestion. At the end of the ischemia, the right lateral and caudate lobes of the liver were removed. Survival of the animals was assessed at 5 days. ATP and LP levels were evaluated at the end of the ischemic time and 1 hour after reperfusion by bioluminescence analysis and malondialdehyde (MDA) tissue concentration. **RESULTS.** Intermittent clamping markedly improved the animal survival at all the ischemic time considered when compared to the continuous group (60 min: 100% *vs* 25%; 90 min: 75% *vs* 0%; 120 min: 50% *vs* 0%). ATP and MDA concentrations were significantly reduced after 2 hours of intermittent or continuous ischemia; however, the decrease was markedly greater in liver exposed to continuous ischemia (0.66±0.05 *vs* 0.91±0.07 pmol/mg; p<0.01). After reperfusion, ATP and MDA levels increased in both groups; however after 1 hour of reperfusion, MDA concentration was significantly lower in liver exposed to intermittent ischemia (2.1±0.6 *vs* 4.6±0.7; p<0.05). **CONCLUSIONS.** Intermittent oxigen deprivation of the liver is followed by an higher rat survival, if compared to continuous ischemia. Moreover, it reduces liver energy depletion caused by anoxia and decreases tissue lipid peroxidation that occurs during reoxygenation. These results suggest that intermittent rather than continuous "Pringle manouvre" may reduces liver injury during resections.

### F091

### ORTHOTOPIC LIVER TRANSPLANTATION (OLT) WITH PRESERVATION OF THE INFERIOR VENA CAVA (IVC) AND A TEMPORARY PORTO-CAVAL SHUNT (PCS).

D. Neri, G.E. Gerunda, R. Merenda, F. Barbazza, A. Bruttocao, G. Da Giau, A. Maffei Faccioli
Istituto di Chirurgia Generale I, Padua University

OLT is a codified surgical procedure since many years. The use of the veno-venous by-pass (VV) during epatectomy and the resection of the IVC with the recipient liver are steps of the usual technique. Recently some AA. described the preservation of the recipient IVC and the implant of the graft performing an end-to-side anastomosis between the donor IVC and a cuff obtained or by the confluence of the recipient suprahepatic veins (Piggy-back technique, PBT) or by a longitudinal enlargement of the recipient left suprahepaic vein (Belghiti technique, BT). Moreover the recipient IVC preservation make possible to perform an end-to-side PCS during the anhepatic fase to avoid the use of the VV. From July 1991 to December 1995 we perform 77 OLT in 67 patients. The standard technique was used in 7 cases, all with the VV. The PBT and the BT were used both in 35 cases; VV was restrict to only 6 cases of PBT; PCS was used in 16 and 24 cases of PBT and BT, while in 13 and 11 cases respectively we were able to perform nor a VV nor a PCS, because portal vein clamping was well tollerated. All the new techniques have pros and cons and the surgeons working in this field have to know all the possibility, even if the standard technique is the right choice when the dissection of the IVC appear too difficult. Moreover performing a PBT it is possible to cross-clamp the IVC when the surgeon have to prepare a cuff using both the recipient suprahepatic veins; the BT, with a longitudinal clamping of the IVC, avoid this problem. In any case the use of the techniques of IVC preservation have these advantages: 1) avoid of the VV, with the associed risks, cost and time consume, 2) easier control of the bare area, 3) easier mobilization of the liver performing hemostasis, 4) eventually, an easier retransplantation.

### F092

### THE EFFECTS OF THROMBOXANE A2 SYNTHETASE INHIBITOR (OKY-046) ON HEPATIC ISCHEMIA-REPERFUSION INJURY IN RATS WITH OBSTRUCTIVE JAUNDICE

S. Noshima, N. Morita, Y. Kobayashi, D. Hayashi, K. Okamura, T. Inokuchi, M. Orita, H. Shinagawa, T.Takahashi, K. Esato
First Deprtment of Surgery, Yamaguchi Univ, Ube., Japan

The effects of OKY-046, a thromboxane $A_2$ synthetase inhibitor, on complete hepatic ischemia with obstructive jaundice were studied in male Wistar rats in vivo. This objective was to investigate the influence of OKY-046 on the hepatic microcirculation as measured by tissue partial oxygen pressure ($tPO_2$) and on release of interleukin-8 (IL-8) in hepatic tissue after reperfusion. Fourteen days after bile duct clamping rats were subjected to 30 minutes of warm complete liver ischemia and then reperfusion 30 minutes. Rats were divided into three groups; the first without treatment (group C), the second administered OKY-046 from 15 minutes before hepatic ischemia to the end of the experiment (group OKY), and the third Gadolinium chloride (group Gd) was injected intravenously to evaluate the contribution of the Kupffer cell to IL-8 production. Group OKY maintained significantly higher levels of $tPO_2$ at 5, 10, 15 min after declamping compared with group C ($p<0.05$). The level of IL-8 in liver tissue in group OKY tended to be lower compared with group C. Group Gd demonstrated the lowest level of IL-8 among the three groups ($p<0.05$). Results demonstrated that OKY-046 improved the hepatic microcirculation during the reperfusion period, influenced the Kupffer cells, and depressed the concentration of IL-8 in liver tissue.

### F093

**ASSESSMENT OF STENOSIS IN TRANSJUGULAR INTRAHEPATIC PORTOSYSTEMIC SHUNTS: A PROSPECTIVE DOUBLE-BLINDED STUDY COMPARING ULTRASOUND WITH DIRECT PRESSURE MEASUREMENT**

**C. Owens, D. Warner, C.Bartolone, R.Eisenstein, J.Hiblen, T.Wiley, T.Layden**
Department of Radiology and Internal Medicine, University of Illinois Medical Center, Chicago, Illinois, USA

A Prospective, double-blinded study was undertaken to assess the sensitivity of ultrasound in determining patency, stenosis or occlusion of transjugular intrahepatic portosystemic shunts on routine follow-up examination. Thirty-six evaluations were preformed on twenty-seven patients over a four month period. The ultrasonographer and the interventional radiologist were blinded to each others findings. The data was collected and analyzed by the gastroenterologist.

Of the thirty-six examinations preformed, ultrasound was inaccurate in assessing the status of the shunt 42% of the time.

Our study contraindicates the current openion that ultrasound is highly sensitive in detecting early stenosis of intrahepatic portosystemic shunts. With the high restenosis rate seen with transjugular intrahepatic portosystemic shunts and the high morbidity associated with recurrent variceal bleed, accurate assessment of shunt patency and early detection and correction of shunt stenosis is paramount in the management of patients with portal hypertension and intrahepatic shunts.

### F094

THE USE OF PROSTAGLANDIN E1 IN LIVER TRANSPLANT (LT) RECIPIENTS WITH SEVERE GRAFT DYSFUNCTION (SGDF)

S. Pan, R. Lopez, A. Hoffman, L. Sher, M. Fraiman, L. Makowka
Liver Transplant Service, St. Vincent Medical Center, California, USA

Severe hepatic allograft dysfunction is associated with graft loss and may predispose to infectious complications. Prostaglandin E1 (PGE1) has cytoprotective and vasodilatory properties which improve endothelial cell integrity and increase hepatic blood flow. We evaluated the use of PGE1 in recipients with SGDF following ABO compatible LT. **METHODS**: SGDF was defined as poor bile production & one or more of the following: 1) initial prothrombin time (PT) > 19sec., 2) initial AST > 2000u/l, 3) elevation of PT for 24hrs, 4) elevation of AST for 24hrs. Thirty-nine recipients had SGDF & 22 were treated with PGE1 at 0.009 ± 0.003mcg/kg/min for 68.9 ± 32.6 hrs (mean ± SD). The change in PT & AST at 24 & 48hrs, the use of FFP within 48hrs, ICU stay & graft loss within 3mos due to re-LT for nonfunction or death due to infection were compared using t-test & Fisher's exact test.

| **RESULTS:** (mean±SD) | **Control (n=17)** | **PGE1 (n=22)** | **p value** (* P<0.05) |
|---|---|---|---|
| Baseline PT | 19.14 ± 3.07 | 20.87 ± 4.06 | 0.152 |
| Δ PT at 24hrs | 1.05 ± 3.32 | - 1.18 ± 3.66 | 0.057 |
| Δ PT at 48hrs | - 1.48 ± 3.14 | - 4.39 ± 3.58 | 0.013* |
| Baseline AST | 2404 ± 1037 | 3437 ± 1754 | 0.038* |
| Δ AST at 24hrs | 311 ± 1614 | - 1151 ± 2282 | 0.031* |
| Δ AST at 48hrs | - 962 ± 1529 | - 2622 ± 1791 | 0.004* |
| Graft loss in 3mos | 6 (35.3%) | 1 (4.5%) | 0.030* |

Four patients had re-LT (3 in control, 1 in PGE1). Three control patients died due to infection & SGDF. The use of blood products & ICU stay were not statistically different. The cost of PGE1 therapy was $972 ± 645/pt. PGE1 was well tolerated.

**CONCLUSIONS**: PGE1 improves allograft function as measured by PT & AST and reduces allograft loss due to re-LT & death from infection. However, it does not diminish the need for blood products or shorten ICU stay. The cost of this therapy should be weighed against the cost of re-LT, the morbidity & mortality of serious infection, and limited donor availability. Our data support the need for a controlled trial.

### F095

PROPHYLACTIC SCLEROTHERAPY (PES) IN HIGH-RISK CIRRHOTICS SELECTED BY ENDOSCOPIC AND HEMODYNAMIC CRITERIA - A SECOND PROSPECTIVE CONTROLLED RANDOMIZED TRIAL

K-J. Paquet, J-F. Kalk, C-P Klein
Dept. of Surgery and Medicine, HEINZ-KALK-Hospital, D-97688 Bad Kissingen, Germany

Although the first variceal bleeding in patients with liver cirrhosis has an extremely high mortality rate, prophylaxis is a matter of controversy. Thirteen controlled trials of sclerotherapy (ST) for the prevention of the first variceal hemorrhage in cirrhotics have given conflicted results. Main reasons were different patients' populations and different selection criteria. Therefore, we designed a new study in which 89 from 396 investigated patients after endoscopic and hemodynamic selection were randomised either to ST (44 patients) or control (45 patients). Admission criteria were no history of variceal bleeding, the presence of high risk varices, i.e. varices degree III and IV with minivarices on their top and a portal pressure over 16mmHg. ST sessions were performed at time 0, 7, 14, 21, 28 days, until the varices were reduced at least for two degrees in size and completely covered by fibrous tissue. Follow-up endoscopy was performed at four and thereafter at six month intervals. Control patients underwent repeated clinical investigation and endoscopy at six months intervals. Bleeding episodes were treated by emergency sclerotherapy (EES) in both groups, whenever possible. Mean follow-up was 33 months. Analysis of the results were performed by Students T- and Longrank test. Variceal bleeding occured in 12 ST-patients (27.3%) and 33 controls (73.3%) ($p < 0.01$). Overall-mortality was 31.8% in ST-pat. and 68.9% in controls ($p < 0.01$). PES was able to prolong survival in CHILD class A and B but not in C. It is concluded, that PES does reduce the incidence of first variceal bleeding in cirrhotics and is able to prolong survival in pat. with good liver function if only high risk patients are selected and PES is performed by endoscopic experts.

### F096

PROGNOSIS OF DISTAL SPLENORENAL SHUNT IN CIRRHOTICS WITH SCLEROTHERAPY FAILURES AFTER STRICT SELECTION BY FUNCTIONAL AND HEMODYNAMIC CRITERIA

K.-J.Paquet, A.Lazar, F.Janocha, R.A.O.Sonak, R. Kuhn
Dept of Surgery, Medicine, HEINZ KALK-Hospital (HKH), D-97688 Bad Kissingen, Germany

Endoscopic sclerotherapy (ES) and recently ligation (EL) has been established as therapy of first choice in cirrhotics with bleeding esophageal varices. However, early or later sclerotherapy failures (SF) occur in a frequency between 20 to 50%. In concurrence to ES and in spite of TIPS shunt procedures are still the best prophylaxis for recurrent variceal hemorrhage. Should this modalitiy not offered to SF with good liver reserve? - From Jan. 1, 1983 until Jan. 1, 1995 922 pat. with bleeding esophageal varices were admitted to HKH; 507 (55%) belonged to the CHILD-PUGH (CP) classification A and B. 162 (32%) were SF; 72 of them were selected for distal splenorenal shunt (DSRS) on the basis of the following criteria: liver volume between 1000 and 2500ml, portal perfusion index over 30%, exclusion of activity and progression of liver cirrhosis (LC) by biopsy. LC was mainly of alcoholic origin (51/75%). 48 were male and 24 female with a median age of 52.3 (16-71) years. 38 were CP A and 34 B. In ten cases DSRS was technically to risky; a narrow-lumen mesocaval interposition shunt was performed. Thus, 62 pat. (6.7%, 12.2%) received DSRS. - Hospital mortality was 4.8%. In two cases (3.2%) recurrence of variceal hemorrhage occured because of shunt thrombosis (1) and a shunt lumen of 7mm (1). Variceal hemorrhage could be stopped by emergency or elective ES. All pat. could be followed up to July 1, 1995; there was no case of shunt encephalopathy and 8 further deaths. Cumulative survival time according to KAPLAN-MEIER is 80% after five and 70% after ten years. Thus, elective DSRS in a highly selected patients population offers the current best decompressive method for recurrent variceal hemorrhage after SF.

### F097

**INDUCTION OF PLASMINOGEN ACTIVATOR INHIBITOR (PAI-1) mRNA BY SHORT-TIME WARM ISCHEMIA IN HUMAN LIVER.**

**R.Pellicci*, F.Dondero*, A.Antonucci*, M.Barabino*, M.Bertocchi*, C.Bottino*, G.Dardano*, F.Ermili*, R.Mondello*, N.Morelli*, A.Pozzo*, U.Valente*, L.Sampietro#, D.Storace#, D.Boeri#, M.Maiello#**
***Transplantation Center of Genoa #Department of Internal Medicine - University of Genoa, Italy.**

**Histologic finding of the primary failure suggest that at least the final pathway of the process leads to ischemic-type injury and many observations document altered coagulation and fibrinolysis during the postoperative period of OLT. PAI-1 is the main modulator of fibrinolysis, is a component of the acute phase response to injury and is synthesized by endothelial cells and hepatocytes. Aim of this work is to study the effect of ischemia-riperfusion injury on PAI-1 mRNA synthesis in human liver. In 10 subjects undergoing partial hepatectomy for localized lesions the unaffected portion of the liver was biopsed before the clamp of the hepatic vessels and at the end of the surgical procedure, after an average period of recovery from ischemia of 76±3 9 min. Total RNA was isolated by the guanidine isothiocyanate method. The amount of transcripts encoding for PAI-1 and γ-actin mRNAs was determined by the Norhtern technique, with $^{32}$P-labeled cDNA probes. After an average 30 min. of ischemia a four fold increase of PAI-1 mRNA (2p<0.01) is detectable in the hepatic tissue. The increase of PAI-1 mRNA is evident even after a very short time of ischemia (7 min.) and does not seem to be time dependent. The mRNA de novo synthesis seems also very rapidly induced, since its increase is detectable already after 20 min. of recovery from the vascular occlusion. It seems also relevant the great individual variability of PAI-1 synthesis both in basal (1 to 5 in an arbitrary scale) and in the post ischemia-riperfusion state (4 to 20). A decrease of fibrinolytic activity due to the ischemia-riperfusion induced PAI-1 overproduction may play a key role in the pathogenesis of both the primary failure and thrombotic complications.**

### F098

**COMPARISON OF DIFFERENT METHODS FOR THE TREATMENT OF NONPARASITIC LIVER CYSTS**

**A. Petri, É. Makula*, S. Karácsonyi**

**Department of Surgery, * Institute of Radiology, Albert Szent-Györgyi Medical University, Szeged, Hungary**

**Nonparasitic liver cysts (NC) belong to the most common benign focal diseases. Usually they are accidentally detected by different radiological imagine methods when examining patients with atypical complaints. Only a small part of them cause characteristic complaints to the patients.**
**Between 1982 and 1995 eightytwo patients underwent operations because of NC of the liver, 18 of them were operated on by laparoscopic way. There were 16 male (mean age: 51.9 years) and 66 female (mean age: 54,6 years) patients.**
**The most typical complaint was pain in the right hypochondrial region (n = 80).**
**The mean size of the cysts were 57.4 mm in diameter (20 - 140 mm). Seventyfour were solitary and eight were multiple.**
**In thirtyone cases we performed an enucleation, in sixty cases a fenestration and in one case a punction of the cysts. A liver resection had to be carried out in two cases. The gall-bladder was removed simultaneously at eleven patients. Except for the laparoscopic way the operations have been carried out in temporary occlusion of the hepatoduodenal ligament according to Pringle.**
**Postoperative morbidity rate was low in both groups. Fever could be observed in eight cases, leucocytosis in one case, jaundice in three cases, pleuritis and hydrothorax in one case, suppuration of the wound in one case and a sterile abdominal wall disruption also in one case. There was no mortality.**
**Another twentysix patients with NC have been treated with ultrasound guided puntion of the liver cysts. There were four male and twentytwo female patients (mean age: 58.27 years). The cysts were solitary in twentythree cases and multiple in three other cases. The number of the cysts were thirtytwo. The cysts have been sclerotized with aetoxysclerol in three cases and by means of absolut alcohole in four cases. In one case an ultrasound guided drainage of the liver cyst was carried out. One case had to be operated on besause of recurrence of the cyst. Mortality could not be observed.**
**In the laparotomised group the everage time of the hospitalisation was 14.3 days, and among the patients who were operated on by laparoscopic way 7.0 days. In the third group the patients did not require any hospitalisation.**

### F099

INTERMITTENT OR CONTINUOUS HEPATIC PEDICLE CLAMPING . A RANDOMIZED STUDY

F. Pierangeli, P. Jagot, O. Farges, A. Sauvanet, S. Jany, J. Belghiti.
Department of Digestive Surgery, Hospital Beaujon, University Paris VII, Clichy, France.

Pedicle clamping reduces blood loss during hepatic resection. In an effort to increase the tolerance of the liver to ischemia, intermittent pedicle clamping (IC) has been used, especially in cirhhotic patients by many authors. The aim of this study is to assess the results obtained by IC and continuous clamping (CG) by means of a controlled study.
Patients and methods : Sixty three consecutive patients who underwent hepatic resection without total vascular exclusion were included from April 1995 to December 1995. Patients were stratified according to the presence of an underlying cirrhosis. Patients randomly assigned to IC (15 min) included 20 non cirrhotic and 10 cirrhotic whereas 23 non cirrhotic and 10 cirrhotic were assigned to CG. Both groups were well matched with respect to operative risk factors as age, extent of resection and preoperative hepatic function. Clamping duration, total operative bleeding volume with special reference to bleeding volume during the hepatic clamping phase and postoperative liver tolerance including prothrombin time (PT), bilirubin serum level(B) and aminotransferases (ALT,AST) were compared and summarized as follow :
Results : 1) In the non cirrhotic group :

| | Clamping Duration(mn) | Total operative bleeding (ml) | Clamping operative bleeding (ml) | Mortality (n°) | Hepatic failure(n°) |
|---|---|---|---|---|---|
| I C | 40±13 | 1494± 1100 | 503±242 | 0 | 0 |
| CG | 39±13 | 1271± 1050 | 242±221 | 0 | 1 |
| | NS | NS | p<0,01 | NS | NS |

Postoperative AST serum level was higher in the CG group than in the IC group with a significant difference at day 2 (322 ± 180 and 148 ± 108 respectively)(p<0,05). Postoperative PT and Bilirubin were not different.
2) In the cirrhotic group :

| | Clamping Duration(mn) | Total operativo bleeding (ml) | Clamping operative bleeding (ml) | Mortality (n°) | Hepatic failure(n°) |
|---|---|---|---|---|---|
| IC | 47±12 | 1347±267 | 531±317 | 0 | 0 |
| CG | 32±9,5 | 1228±761 | 413±302 | 2 | 3 |
| | p<0,01 | NS | NS | NS | P<0,1 |

Postoperative Bilirubin was significantly higher in the CG group than in the IC group at day 2, day 3 and day5. PT, AST and ALT were not different in the two groups.
In conclusions although operative bleeding during the clamping phase is increased, intermittent pedicle clamping is better tolerated than continuous clamping and should be used especially in cirrhotic patients.

### F100

**HGF/SF ENHANCES TUMOUR-MATRIX INTERACTION BY STIMULATION OF THE PHOSPHORYLATION OF FOCAL ADHESION KINASE AND PAXILLIN**

MCA Puntis and WG Jiang. University Department of Surgery, University of Wales College of Medicine, Cardiff, UK

Cell-matrix interaction is an essential parameter in the process of liver metastasis formation. This interaction is via the binding of extracellular matrix and cellular integrin and activation of down-stream signals including the focal adhesion kinase (FAK) and paxillin. This study examined the role of hepatocyte growth factor/scatter factor (HGF/SF), a tumour invasion promoter, in tumour-matrix interaction.

Human colon cancer cell HT115 was used. The basement membrane (matrigel) was used to determine cell-matrix interaction. Cell numbers after adhesion were determined by Hoescht 33258 assay. Tyrosine phosphorylation of focal adhesion kinase and paxillin was detected by immunoprecipitation and Western blotting. The presence of HGF/SF in the assay system significantly increased cell-matrix binding and this was seen in a concentration dependent manner. Pretreatment of cell with HGF/SF and subsequently depletion of HGF/SF also increased the binding, suggesting an activated adhesion mechanism. HGF/SF increased the tyrosine phosphorylation of both focal adhesion kinase and paxillin shortly after treatment. This was seen together with the activation of HGF/SF receptor (cMET proto-oncogene).

We conclude that HGF/SF enhances tumour-matrix interaction by stimulation of the phosphorylation of focal adhesion kinase and paxillin.

### F101

### INTRAOPERATIVE ASSESSMENT OF THE GRAFT BLOOD FLOW PERFUSION IN LIVER TRANSPLANTATION BY LASER DOPPLER FLOWMETER: FIRST CLINICAL EXPERIENCE

A. Seifalian, S. Mallett*, K. Rolles, B. Davidson
University Department of Surgery and Liver Transplantation and *Department of Anaesthesia, Royal Free Hospital and School of Medicine, London, U.K.

Satisfactory graft re-perfusion is fundamental to successful liver transplantation. Laser Doppler flowmetry is a technique which can measure hepatic blood flow continuously without altering the flow in the microcirculation. Here we report its first application to clinical liver transplantation.

Hepatic tissue microcirculation was measured intraoperatively in 22 liver grafts. Data was examined with respect to (a) effects of portal vein (PV) inflow, and (b) effect of hepatic artery (HA) inflow, both up to 30 min after revascularisation. Laser Doppler flowmeter (LDF) readings from the surface of the left lobe were validated against total liver blood flow using an electromagnetic flowmeter (EMF) on PV after revascularisation of PV flow.

There was a significant correlation ($r = 0.96$; $p < 0.001$, n=8) between surface left lobe liver perfusion using the LDF and total liver blood flow measured by EMF. The LDF was reliable and robust under clinical conditions and provided reproducible measurements of perfusion with a coefficient of variation of approximately 4%. Re-perfusion of the transplanted liver with venous blood was accompanied by an immediate increase in liver blood flow perfusion. Over the subsequent 10-30 minutes there was no significant increase in flow and re-perfusion of the graft with arterial blood did not increase liver blood flow perfusion. Hepatic tissue blood flow showed a negative correlation with cold ischaemic time ($r = -0.48$, $p < 0.025$, n=22) on graft perfusion at the early phase of revascularisation of portal vein blood, but this correlation disappeared by the end of the operation ($r = -0.11$, $p = 0.611$).

We conclude that LDF provides a reliable non-invasive method of monitoring liver graft blood flow perfusion during transplantation.

### F102

### SINGLE CENTRE EXPERIENCE IN HEPATIC MALIGNANCY AND TRANSPLANTATION

SreekumarNS, El-WashM, DavidsonBR, RollesK, BurroughsAK.
University Dept of Surgery, Royal Free Hospital, London, UK.

Past decade has brought major changes in treating Hepatic Malignancies with OLT. This study looks at outcome of Transplantation for tumors.
283 patients underwent 316 OLT from 22-10-1988 to 22-10-1995. 42 had tumors(14.8%), classified into following three categories.
1. 7/42 had OLT (1988-1990) for large irresectable tumors without other liver diseases; HCC(4/7), CCA(1/7), Epitheloid Haemangioendothelioma (1/7), Recurrent Cystadenocarcinoma(1/7). 5/7 died of metastasis(4/12 to 2 yrs). 2/7 disease free at 5 years(HCC & Rec.cystadenoCa).
2. 21/42 patients had cirrhosis(HBV-7, HCV-6, ALD-1, PSC- 4, Glycogen Storage Disease-2, and Budd Chiari Syndrome-1), and malignancy-HCC(17/21), 4/21 cholangiocarcinoma (CCA). Pre-operative staging(I,II,"Transplantable") was upgraded in pathological staging (Stage-III-5, IVa-4, IVb-1).Adjuvant therapy (15/17, HCC only) preop were Lipiodol Epirubicin(9), TACE+Epi(1), Targeted alcohol(2),Targeted Alc+IV Epi(2). Post op Chemotherapy in 7 patients so far, IV Epirubicin(6), 5FU+Cisplatin(1). One CCA patient(NED 4yrs) is alive. Two died of reccurence; 8/12 and 2 yrs, confirming poor prognosis of OLT in CCA reported by others. 2/17 HCC have reccurence, 9/17 have died- metastasis(1), reccurent Hepatitis B with no tumor-4(5/12 to 17/12 post OLT), other causes (4/17). 7/17 are alive NED (4/12 to 5yrs).
3. 14/42 had incidental HCC diagnosed postoperatively(7/14) or suspected preoperatively(7/14) in OLT for ESLD. Lesions were <1.0cm(1),1to 3 cms(6), >3.0cms(5);unifocal(8), and multifocal(4). 5/14 died with NED, and 9/14 are alive (5/12 to 6.4 yrs) with recurrence in 1.
OLT has a role in the treatment of HCC. Careful preoperative staging is required,including exploratory laparotomy, backup recipient, and improved imaging techniques for tumors in cirrhotics. Study protocols are required to evaluate adjuvant therapy in HCC. Incidental tumors have better prognosis but pathological staging determines the outcome.

### F103

### POST-TRANSPLANTATION GROWTH IN PEDIATRIC LIVER RECIPIENTS

J.F. Renz, M. de Roos, P. Rosenthal, J.P. Roberts, N.L. Ascher, and J.C. Emond
Liver Transplant Program, University of California San Francisco, San Francisco, CA

Growth failure is a major complication in children with end-stage liver disease. While transplantation reverses liver failure, post-transplantation growth patterns have not been well characterized. We examined growth patterns in pediatric liver recipients to determine the incidence of growth restoration after transplantation and identify risk factors associated with poor growth. Between 1988 and January 1995, 38 recipients (male:22, female:16) with a mean follow-up of 22.4±14.5months were included according to the following criteria: age 15years or younger, greater than one year post-transplantation, liver only recipient, extensive post-transplant follow-up, and have not required retransplantation. Children were classified by age and indication for transplantation: cholestasis (50%), metabolic (28%), fulminant hepatic failure (12%), hepatitis (5%), and miscellaneous(5%). Immunosuppression was CSA/prednisone primary therapy with α-T-cell induction. Tacrolimus was reserved for treatment failures. Post-transplant growth was assessed at one year by comparison of individual recipient weight for height growth curves to age and sex-matched National Center for Health Statistic (NCHS) growth percentiles and classified as:below NCHS predicted, 0-15% above NCHS predicted, 16-40% above NCHS predicted, and greater than 40% above NCHS predicted.

| GROUP (years): | 0-1 (n=11) | 1-2 (n=9) | 2-7 (n=8) | 7-15(n=10) |
|---|---|---|---|---|
| mean age (mo) | 5.6±2.0 | 17.2±3. | 656.2±16.0 | 146.7±15.0 |
| <NCHS (%) | 10 | 22 | 12 | 30 |
| 0-15% abv NCHS (%) | 45 | 12 | 12 | 20 |
| 16-40% abv NCHS (%) | 18 | 33 | 50 | 50 |
| >40% abv NCHS (%) | 27 | 33 | 25 | 0 |

Rapid growth (>15% above NCHS predicted) was observed in 61% of recipients below seven years of age. In recipients with cholestatic disease (n=20), there was an inverse relationship between NCHS percentile at transplantation and one year post-transplant growth. Age was not related to one year growth below the age of seven; however, recipients above the age of seven demonstrated overall poor growth. No relationship was found between growth and incidence of rejection. These data demonstrate that despite pre-existing growth failure, most recipients grow well. "Catch-up" growth occurred in each recipient group with rapid growth in 61% of recipients below seven years of age. Below seven years, there was an inverse relationship between one year post-transplant growth and NCHS percentile at transplant. While the role of immunosuppression could not be clearly defined, our data are in close agreement with growth data obtained from pediatric renal recipients.

### F104

### THE SCID MOUSE AS A MODEL FOR TRANSPLANTATION STUDIES

J.F. Renz, M. de Roos, Z. Lin, A. Dalal, and N.L. Ascher
Liver Transplant Program, University of California San Francisco, San Francisco, CA

Mice expressing the Severe-Combined-Immunodeficiency trait (SCID) lack functional T- and B- lymphocytes and have been widely used for the study of B-cell development, cancer, and HIV research. The purpose of this investigation was to determine the immune response of reconstituted SCID mice and assess the validity of the SCID mouse model for transplantation studies. C3H SCID mice screened by fluorescence activated cell sorting (FACS) and radial-immunodiffusion assay (RID) were defined as $SCID^{+}$ if $CD3^{-}$, $CD22^{-}$ and serum [IgG] <5mg/L. $SCID^{+}$ mice pre-treated with 250R g-radiation were reconstituted (recon) with 3-$5x10^{7}$ donor bone marrow cells (syngeneic[syn]: male C3H, allogeneic[allo]: male BALB/c) by IV injection. Four weeks post-transplant, engraftment was determined by 1) FACS, 2) repopulation of blood (WBC), thymus, and spleen, 3) RID and 4) histologic evaluation. Greater than 90% of syn and allo reconstitutions expressed $CD3^{+}$,$CD4^{+}$, $CD8^{+}$, and $CD22^{+}$ cells of donor origin in peripheral blood and spleen. Cell subpopulations were not significantly different between recon SCID mice and C3H or BALB/c $SCID^{-}$ controls with engraftment stable for >4 months. WBC, total thymocyte, and total splenocyte counts were significantly elevated ($p< 0.05$: ANOVA, students' t) following recon to levels found in wild-type controls. Serum [IgG] for recon SCID mice was >150mg/L (n=10) vs 322±26mg/L for wild-type controls with histologic lymphocyte engraftment of spleen, duodenum, and thymus. Immune function against donor, recipient, and third party antigen was assayed *in vitro* by mixed lymphocyte response (MLR) and cell-mediated cytotoxicity. Allo-SCID splenocyte response against third party antigen was significantly elevated ($p<0.01$: ANOVA, students' t) compared to unrecon SCID with a stimulation index (SI) equal to donor and recipient wild-type controls. *In vivo* immune function was determined by full-thickness skin grafting and pancreatic islet cell transplantation. Allo-SCID mice demonstrated rejection of third party skin grafts between post-operative days 9-14 (controls: postoperative day 7-11). In islet transplantation experiments, allo-SCID mice accepted both donor and recipient background islets (euglycemic > 100days) while rejecting third party donor islets between post-transplant day 6 and 9. Chimerism within the allo-SCID was further assayed by FACS analysis of the liver nonparenchymal (NPC) cell fraction. The NPC fraction express MHC Class II and are central in antigen presentation. With engraftment, the allo-SCID NPC fraction is principally of donor background. The immunologic role of these cells is under study. The SCID mouse model demonstrates a stable chimera with B- and T-cell immune function comparable to wild-type mice. Thus, it serves as a useful model for the induction of chimerism, thymic education, and cellular and tissue transplantation studies.

### F105

**SMALL-FOR-SIZE GRAFT UTILIZATION IN LIVING RELATED LIVER TRANSPLANTATION**

J.F. Renz, N.L. Ascher, J.P. Roberts, R.C. Lim, and J.C. Emond
Liver Transplant Program, University of California San Francisco, San Francisco, CA

Living related liver transplantation (LRLT) is an effective modality for children with end-stage liver disease. Optimal results have been realized in small children using adult donors since the minimal graft volume necessary to support homeostasis is unknown. While formulae exist to predict donor liver volume based upon body surface area (BSA), ideal body weight, and volumetric computed tomography, to date, no study has analyzed small-for-size graft function as the lower threshold of graft size is approached. From July 1992 through November 1995, 20 LRLT have been performed on 18 children and two small adults. Recipients were divided into "small-for-size" ([SFS], male:3, female:3, median age:160mo) and "appropriate-for-size" ([AFS], male:9, female:5, median age:14mo) groups based upon the actual volume of the transplanted graft. "Small-for-size" is defined as actual graft volume less than 60% of predicted recipient liver mass as calculated by BSA (SFS mean=0.38±0.1%, AFS mean=1.20±0.2%). Retrospective analysis of intra-operative, clinical and laboratory data indicate SFS grafts exhibit a specific pattern of dysfunction as the lower threshold of functional volume is approached. Early function was significantly decreased in the SFS group with PT=18.2±2.2sec vs 14.8±1.6sec on post-operative day(POD) #3 ($p=0.034$) and remained significantly different through POD #12 ($p<0.05$). All SFS recipients developed cholestasis with significantly increased serum bilirubin by POD #7 and remained significantly cholestatic through POD #60 ($p<0.05$). Histologic review of protocol biopsies in the SFS group revealed a diffuse ischemic pattern characterized by "preservation injury" with cellular ballooning on POD #7 which progressed to cholestasis; however, early ischemic injury, as evaluated by serum aspartate-aminotransferase, was not significantly different between SFS (294±134) and AFS (183±55) at 24 hrs. Regression analysis indicated a significant correlation between graft volume and impaired function ($p<0.001$) with an exponential decrease in graft function below 50% of expected liver mass. An independent correlation was also identified between graft function and both donor and recipient blood loss as an indication of surgical injury. The recipient of the smallest graft (23% of expected liver volume) required retransplantation for primary nonfunction. This study represents an early attempt to define the lower limits of small liver graft use in LRLT which is imperative for the ultimate expansion of LRLT to include larger children and adults.

### F106

**INCIDENCE OF BACTEREMIA AFTER BAND LIGATION AND SCLEROTHERAPY OF ESOPHAGEAL VARICES: A COMPARATIVE STUDY IN SCHISTOSOMOTIC PATIENTS.**

MRS Rohr, ES Siqueira, RR Castro, E Della Libera Jr., AP Ferrari Jr.
Division of Gastroenterology, Universidade Federal de São Paulo, São Paulo, Brazil.

The incidence of transient bacteremia after Endoscopic Sclerotherapy (ES) of esophageal varices (EV) reaches 50% in some studies. This number is thought to be reduced with Endoscopic Band Ligation (EVL). We compared the incidence of transient bacteremia after both procedures in patients with hepatosplenic form of Schistosomiasis and EV. Five and 30 minutes after each treatment session, blood samples were inoculated in soy broth medium (BACTEC®). Aerobic and anaerobic cultures were performed. A total of 87 procedures were included: 50 in the ES group and 37 in the EVL. Bacteremia diagnosed by positive cultures occurred after 5 sessions: 2/50 (4%) of ES (one culture was positive in the 5 min. sample: Peptostreptococcus sp. and another in the 30 min. culture: and Streptococcus sp.). In EVL group, 3/37 (7,6%) procedures were accompanied with bacteremia (Staphylococcus aureus at 5 minutes, Staphylococcus aureus at 5 and 30 minutes, and Staphylococcus epidermidis at 5 minutes). No patient developed clinical evidence of infection. In conclusion, incidence of bacteremia after ES or EVL, in patients with hepatosplenic schistosomiasis was not statistically significant (p > 0,05) when procedures were performed in hepatosplenic schistosomiasis. Imunological differences between schistosomotic and cirrhotic patients could explain the low incidence of bacteremia in this study.

### F107

**HEPATIC HEMANGIOMA: US, CT and MRI FEATURES. 6-48 MONTHS IMAGING FOLLOW UP.**

A. ROLLE, C. BONINI, et al.
Sanatorio Parque. Fundación Dr. R.J.Villavicencio
Rosario. Argentina.

The hepatic hemangioma is the most frequent benign tumor of the liver. It is generally an ordinary and incidental finding in imaging routine examinations being mainly a problem for oncologic patients. They are more frequently found in women in the right lobe of the liver. Many authors have pointed out the differences among the imaging methods in order to establish the diagnoses of this lesion. The purpose of this paper is to introduce the US, CT, and MRI features of the hepatic hemangioma in a great number of patients (791) and to make a 6-48 months US follow up so as to determine possible changes in size and signal. Between December 1989 to December 1994 we examined 791 patients with hepatic hemangioma over a total of 30566 individuals. These 791 patients have undergone 1192 studies. All of them are under clinical, laboratory and imaging follow up. Hepatic hemangioma showed an hyperechoeic US feature en 93.2%, with no wall, and posterior enhancement (Bismuth criteria); in CT 100% of the lesion were hypodense without enhancement and hyperdense in 96.7% after contrast administration. The early angioscan technique presented peripheral enhancement being opacified all the lesion in late section (30') (Freeny's triad). The succes of the CT examinations depended on tumor size, intravenous contrast administration, patient cooperation, and angioscan technique. In MRI 92.5% of the tumors were hypointense in T1 and 91.3% hyperintense in T2. The hemangiomas do not present changes as regards size and US features during two years, for wich, **in an appropriate clinical context** US follow up is not essential.

### F108

**PROTECTIVE EFFECTS OF TRIIODOTHYRONINE ON ISCHEMIA REPERFUSION INJURY OF THE LIVER.**

A. Saglam, Ö. Şakrak, H. Paşaoğlu, F. Öztürk, A. Tutuş, Y. Arıtaş

Departments of Surgery, Biochemistry and Pathology, Erciyes University School of Medicine, Kayseri, Turkey.

We investigated the protective effect of triiodothyronine (T3) on a liver ischemia reperfusion model on rats. Portal and left lateral lobes of the liver clamped and 70% liver ischemia was made. Following the ischemia clamp was opened and reperfusion was established.

Study was carried out on T3 treatment and control groups. L triiodothyronine in a dose of 300 mcg/kg/day per os for 10 days was given to the treatment group and hyperthyroidism constituted. Each treatment and control groups has 7 subgroups: sham operation, ischemia only, 15 minutes, 2 hours, 24 hours, 48 hours and 7 days following ischemia reperfusion injury. Animals are sacrificed after obtaining blood and tissue samples, repeated reoperation is avoided.

Serum AST ALT LDH values and tissue lipid peroxidation were studied, and histopathologic examinations of the ischemic liver were done.

AST, ALT, LDH and lipid peroxidation of the ischemic tissue were significantly lower in the treatment group when compared controls. Histopathologic damage was also less severe in the treatment groups.

These findings suggests that L triiodothyronine pretreatment has protective effects on ischemia reperfusion injury of the liver.

F109

HOW TO IMPROVE NATIVE LIVER REGENERATION IN AUXILIARY LIVER TRANSPLANTATION ?

A. Sauvanet, S. Yang*, D. Bernuau§, P. Beyne*, MH. Denninger*, D. Lebrec*, S. Erlinger*, J. Belghiti. Department of Digestive Surgery and *Hepatology, Hôpital Beaujon, Clichy, France ; §Inserm U-327, Faculté Bichat, 75018 Paris, France.

In case of fulminant hepatitis, auxiliary liver transplantion (ALT) is proposed to supply for the native liver (NL) until it regenerates. Technical factors of the NL regeneration are not yet known. The aim of this experimental study was to assess the NL regeneration according to the graft (G) weight and the location of portal anastomosis in ALT.

Material and Methods: 24 syngenic ALT using an arterialized G were performed in a rat model after a 80% reduction of the NL and were allocated into 4 groups : A = 50% reduced-sized G anastomosed to the superior mesenteric vein (SMV) and NL vascularized by the pancreas, the spleen and the stomach (n=6), B = full-sized G on SMV (n=6), C = 50% reduced-sized G anastomosed to the portal vein (PV) and NL vascularized by the stomach (n=5), et D = full-sized G on PV (n=7). NL and G regeneration were assessed at day 2 or day 4 by in vivo incorporation of tritiated thymidine, then by weighting at day 30 after sacrifice. Weight variation of the NL ($\Delta W = (W_{30}-W_0)/W_0$) was calculated as well as the ratio NL/W = NL weight/rat weight and L/W = NL weight + G weight/rat weight at ALT and at sacrifice (respectively $NL/W_0$, $NL/W_{30}$, $L/W_0$, et $L/W_{30}$).

Results: At day 0, the ratio $L/W_0$ was 2.88±0.25% (group A), 4.58±0.28% (group B), 2.68±0.15% (group C), and 4.73±0.16% (group D) respectively (normal value = 3.88±0.24%). At day 2 and day 4, tritiated thymidine incorporation in NL was more important when G was anastomosed to PV but did not change according to the G size. At day 30, ΔW of the NL was more important in case of a full-sized G (groups B+D *vs* A+C) and G anastomosis to the PV (groups A+B *vs* C+D). The ΔP of NL was + 212±123% in group B and + 96±81% in group C (p=0.07, Mann-Whitney's U-test). The ratio $NL/W_{30}$ was 2.32±0.68% in group B and 1.21±0.63% in group C (p=0.02). The ratio $L/W_{30}$ was normalized in groups A (3.87±0.37%) and C (3.82±0.41%), and was still increased in groups B (4.65±0.60%) and D (4.56±0.63%).

Conclusions: In this model of ALT : 1) A better NL regeneration at day 30 was observed using a full-sized G anastomosed to the SMV; 2) This result is not fully determined by the regeneration occuring until day 4 ; 3) Excess of overall liver weight persists at day 30.

F110

HEPATIC HYDATIDOSIS. A MULTICENTRIC STUDY OF SURGICAL PROCEDURES IN 971 CASES.

M.A.Secchi;R.Pettinari;C.Ledesma;R.Bracco;O.Andriani;R. Cabral;R.Giménez;C.Mercapide;E.Tagliaferri;J.Fraracio y C.José.

H.Italiano(Rosario),H.Regional(C.Rivadavia),H.A.Zatti (Viedma),H.Interzonal(M.del Plata);H.Naval(B. Aires) Republica Argentina.

We retrospectively reviewed 971 surgical cases of hydatid cyst of the liver of patients treated over the period 1970-1995, in order to evaluated indications and results of the surgical procedures.METHODS:patients were divided into 3 groups:Group 1:n=238 Radical Treatment:total pericystectomy or segmentectomy in emergent cyst, when the risk is low. Group 2:n=524 Conservative Treatment:partial pericystectomy,marsupilization, omentoplasty or external drainage.In multiple hepatic cyst,very large or complicate cyst or when the operative risk is high. Group 3:n=209 Combined treatment, including biliary tract surgery.

| RESULTS:, | Morbility | Mortality | Recurrence |
|---|---|---|---|
| Group 1: (n=238) | 19% | 3.7% | 0.8% |
| Group 2: (n=524) | 29% | 1.3% | 1.5% |
| Group 3: (n=209) | 4.7% | 0.5% | 2.4% |
| Total serie(n=971) | 21.3% | 1.8% | 1.5% |

DISCUSION: Surgery remains today the only definite treatment. Radical procedures were the first choice (in selected cases) in the last ten years, because the progress in hepatic surgery attain to the objetives of shortening the duration of treatment and decreasing the postoperative recurrence rate. We concluded that in our experience the best clinical reaults were obtained with surgical treatment selected for each patient.

F111

SURGICAL TREATMENT OF HYDATIDE DISEASE OF LIVER AND POSTOPERATIVE ULTRASONOGRAPHIC EVALUATIONS

G. Shukriu, A. Siqeca

Department of surgery, Medical Centre, Prizren, Kosovë

There were 43 cases of liver hydatide disease treated surgically, which are evaluated, all performed during the period of June 1986 to Oktober 1995.
Off the cases mentioned, 31 (or 72.1%) were females while the rest 12 (or 27.9%) being male. Concerning the professional backgroung, all were housewifes respectively farmers. Localisacion of the hydatide cysts on the right lobe of liver was present on 30 (or 69.77%) of cases, on the left on 9 (or 20.93%) while in both lobes on 4 (or 9.3%) of all cases. Number of the cysts in the liver was in scale from 1 to 3, in average size of 11.7 cm. In 16 cases (or 37.21%) there were complications of the hydatide liver disease present: in 7 cases (or 16.28% ) there was a rupture of cyst in biliary tract with cystobilliary fistula, in 8 cases (or 18.61%) the secondary cysts in peritoneal cavity and in one case (or 2.32%) a recidive of hydatid cyst of liver. In 39 cases (or 90.7%) the partial pericystectomia was performed, removal of the cyst with omentoplication of the cavity remained while in 4 cases (or 9.3%) there was no omentoplication performed. In cases with rupture of the cyst in billiary tract the exploration of the billiary tract was performed, with the remova of the twin cyst and closing of cystobiliary fistula with omentum. The secondary cysts in the peritoneal cavity were completely removed. There were no intra or postoperative complications in any of the cases mentioned.
Postoperatily all the cases were examined by ultrasound after 3,6 and 12 months. There was no complication evident in controls performed by ultrasound examination in the cases, whatsoever.
We have specifically examined the remaining cavity in the liver after the removal of the cysts and came to a conslusion that in cases where omentoplicasion of the cavity within three months while on cases without the omentoplication it took over six months. As an outcome of our research we consider that the partial pericystectomia with omentoplication gives satisfactory results with a low postoperative morbidity. We sugest ultrasound controls in evaluation of the remaining cavity in the liver and appearance of eventual complications.

F112

BAND LIGATION OR SCLEROTHERAPY OF ESOPHAGEAL VARICES IN PATIENTS WITH HEPATOSPLENIC SCHISTOSOMIASIS ?

ES Siqueira, MRS Rohr, CQ Brant, M Morais, AP Ferrari Jr.
Division of Gastroenterology, Universidade Federal de São Paulo, São Paulo, Brazil

Hepatosplenic Schistosomiasis (HS) is the leading cause of Portal Hypertension (PH) in Brazil. Variceal bleeding is the major complication of the disease and carries high mortality. Although the studies comparing efficacy of Endoscopic Sclerotherapy (ES) and Variceal Ligation (EVL) in the treatment of esophageal varices in cirrhotic patients showed similar efficacy and lower incidence of complication with EVL, there are no studies using only patients with pre sinusoidal PH. We performed a prospective, double blind, randomized study to compare efficacy, complications and recurrence of esophageal varices treated by ES or EVL in patients with HS and Esophageal Varices (EV). Thirty six patients were included (18 in each group). They were treated with repeated endoscopy sessions until eradication of EV. Six months after eradication, endoscopy was performed to look for recurrence and local complications. Less sessions were necessary to eradicate VE in EVL group (2,9 vs. 3,9, p=0,04). Efficacy was similar in both groups (100% in EVL and 88,8% in ES). Treatment failure occurred in 2 (12,2%) patients in the ES group, who developed uncontrollable gastric variceal bleeding and were referred to surgery. There were not significant differences in the incidence of complications. However, 50% of the patients in the ES group needed additional sedation during or immediately after the procedures and it did not happen in any patient of the other group. There was only one case of recurrence in ES group. We concluded that both endoscopic methods of treatment of EV are efficient, with similar incidence of complications and recurrence in this study with schistosomotic patients. Eradication occurred faster and with less pain in EVL group.

### F113

### META ANALYSYS OF EFFICACY OF ENDOSCOPIC SCLEROTHERAPY (ES) ON THE FIRST VARICEAL BLEEDING (FVB) AND MORTALITY RATE IN CIRRHOTICS WITH HIGH RISK VARICES (HRV) IN LONG TERM (24 mo) RCTS

S. SIRINGO, R. COGLIANDRO, G. VITTURINI, C. CARBONE, B. MISITANO, L. BOLONDI, R. CORINALDESI.
ISTITUTO DI CLINICA MEDICA E GASTROENTEROLOGIA-BOLOGNA. ITALY

In unselected cirrhotics the 2 yr. FVB rate is 25% and 40% of the episodes occur within the first 6 mo (Hepatology 1994;20:66). Pts with HRV should have at least a 10% higher FVB rate respect the baseline risk. To minimise heterogeneity we grouped RCTs with similar: a)FVB rate in the placebo groups; b)length of f-u. RCTs whose placebo groups had a 24 mo FVB rate ≥35% were defined as RCTs of HRV. We included RCTs: a)with an average f-u of 24 mo; b)with a 2 yr. FVB rate ≥35% in the placebo group; c)whose results were published in life tables or data communicated by the Authors when requested. Eight of 9 RCTs met these criteria. A total number of 749 pts was included :347 treated and 372 controls (CTR). The FVB rate in the pooled control groups was 48.4% (range 34%-80%), 42% of the episodes occurred within 6 mo. The sample size of 749 pts was required to significantly decrease by ≥10% the risk of a FVB ($\alpha$=5%; 1-$\beta$=80%). The Messori's method for censored data was used (Comput. Progr.Meth.Biom.1993;40:261). The table show the results: among treated pts there was a trend toward a reduction of the FVB rate during the first 6 mo, then the FVB rate significantly decreased within and after the first six months.

| | CUM. BLEED. FREE RATE | | CUM .SURVIVAL RATE | |
|---|---|---|---|---|
| | <6 mo ES - CTR | >6 mo ES - CRT | >6 mo ES - CRT | >6 mo ES - CRT |
| K. Paquet et al. (1986) | 94 - 72 | 91 - 10 | 97 - 83 | 82 - 10 |
| T. Sauerbruch et al. (1988) | 79 - 82 | 51 - 57 | 86 - 77 | 59 - 47 |
| G. Plai et al. (1988) | 90 - 84 | 80 - 38 | 93 - 87 | 66 - 43 |
| R. Pötzl et al (1989) | 80 - 86 | 64 - 60 | 85 - 86 | 72 - 46 |
| E. Kobe et al. (1990) | 87 - 82 | 69 - 33 | 94 - 88 | 67 - 61 |
| D. Trigger et al. (1991) | 88 - 80 | 66 - 52 | 88 - 86 | 66 - 56 |
| R. De Franchis et al. (1991) | 69 - 77 | 58 - 52 | 76 - 85 | 42 - 47 |
| K. Paquet et al. (1994) | 81 - 67 | 75 - 31 | 93 - 85 | 75 - 30 |
| % reduction of risk | 5.2 | 17.1 | 5.8 | 16.6 |
| Long rank OR (95% C.I.) | 0.75 (0.52-1.10) | 0.45 (0.35-0.60) | 0.68 (0.45-1.04) | 0.47 (0.36-0.62) |
| Significance | 0.07 | <0.001 | 0.04 | <0.001 |

CONCLUSIONS. ES should be recommended as long term therapy to prevent the FVB in selected pts with HRV.

### F114

### SURGICAL VS NON-SURGICAL FOCAL LIVER LESIONS: PRE-OPERATIVE EVALUATION WITH SPIO-ENHANCED MR IMAGING

C. Stoupis, H-U Baer, P. Vock, M.W. Büchler.
Clinic for Visceral and Transplantation Surgery and Dept. of Radiology, University Hospital of Berne, Switzerland

Purpose: To assess SPIO-(superparamagnetic iron oxide, a reticuloendothelial system-RES-contrast agent) MR imaging (MRI) in detecting and characterizing liver lesions before surgery.

Methods: 50 patients with known or suspected liver lesions were examined with SPIO-enhanced MRI. Qualitative and quantitative analyses and correlation with surgical and pathological findings were performed.

Results: SPIO enhanced T2-weighted MRI images demonstrated marked decrease in signal intensity of liver (96%). This sequence provided improved liver lesion detection and diagnostic confidence compared to plain MR (81% and 86% resp.). Cases of FNH (n=16) were all depicted and characterized in SPIO-enhanced MRI and the central scar was better seen in post contrast images. Hemangiomas (n=12) revealed different signal behavior than FNH and all were correct identified in enhanced MRI. Adenomas (n=4) were found to have different RES activity. Hypervascular metastases (n=18), because of lack of RES cells were all depicted in SPIO-MRI.

Conclusion: SPIO-enhanced MRI has the potential to be the non-invasive method of choice in the preoperative evaluation of focal liver lesions due to its efficacy to detect and potentially characterize surgical from non-surgical liver lesions.

### F115

### HEPATIC ARTERIOVENOUS FISTULAS AND ANEURYSMS

P.G.Tarazov, A.A.Polykarpov
Division of Angio/Interventional, St.Petersburg Research Institute of Roentgenology & Radiation Therapy, St.Petersburg, Russia

A retrospective study was carried out on 39 patients with vascular diseases of the liver. Of them, 37 pts had intrahepatic arterioportal fistulas (APFs) mostly due to hepatocellular carcinoma (HCC, 21) or iatrogenic trauma (11); the 5 remaining cases included APF in cirrhotic (2), hemangiomatous (2), and normal liver (1). One patient with hepatic hemangioma had hepatic artery-to hepatic vein fistula (AVF) and the other one with cirrhosis had an aneurysm of the common hepatic artery (CHAA). Treatment included liver surgery (4 resectable HCCs), transcatheter embolization ( 20 = 7 HCC+6 large iatrogenic APFs+5 benign APFs+1AVF+1CHAA ), and observation ( 15 = 10 HCC+5 small iatrogenic APFs ).
Surgical treatment was uncomplicated in all 4 pts. Of them, two are alive in 2 and 3 yrs and two died of HCC 1.5 and 2.5 yrs later.
Arterial embolization successfully controlled portal hypertension in all 18 pts with APF and normalized systemic hypertension in a patient with AVF. Unfortunately, recanalization of CHAA developed after embolization. The survival of these 20 pts depended on the main liver disease and varied from 6 mo (HCC) to 10 yrs in benign cases.
All 10 pts with untreated HCC-related APFs died of massive variceal bleeding within 2 mo. All 5 small iatrogenic APFs showed spontaneous thrombosis during follow-up.
It is concluded that large long-standing APFs cause severe portal hypertension with consequent variceal bleeding, so arterial embolization is indicated in most patients. Hepatic AVS is also successfully treated by embolization, while surgery may be indicated in CHAA.

### F116

### RESECTION OF COLORECTAL LIVER METASTASES- A 25-YEAR EXPERIENCE

K.-G. Tranberg, B. Ohlsson, U. Stenram
Departments of Surgery and Pathology, Lund University, Lund, Sweden

This is a review of 107 consecutive patients with colorectal liver cancer resected with curative intent during 1970-1995. The hepatic tumours were single in 60 patients (56 %), unilateral in 81 (76 %) and associated with extrahepatic disease in 12 (11 %). Types of first resection were: right hepatectomy 48, extended right hepatectomy 9, left hepatectomy 10, extended left hepatectomy 1, left sectorectomy 8, mono- or polysegmentectomy 7, major atypical resection 6 and minor atypical resection 18. In addition, reresections were performed in 12 patients. Overall median survival was 27 months, and 5-year survival rate was 25 %. Median survival was 21 months in patients operated before 1985 and 53 months in patients operated later; corresponding 5-year survival rates were 18 and 46 %, respectively. Recurrence in the liver alone occurred in 18 patients (17 %), at hepatic and extrahepatic sites in 43 (40 %) and at extrahepatic sites alone in 19 (18 %). Prognosis was significantly improved by a low number of liver tumours, a free resection margin and no extrahepatic tumour (Cox's proportional hazards model), corroborating the findings in our 1985 review. Three patients died perioperatively (major bleeding 2, liver failure 1) (operative mortality 3 %), but no patient died from complications after 1985. Major postoperative complications (bleeding 3, liver insufficiency 1, intraabdominal abscess 6, respiratory insufficiency 1) were seen in another 8 patients (7 %). It is concluded that survival after curative resection for liver colorectal secondaries is favoured by a low number of hepatic tumours, no extrahepatic disease and a free resection margin. These selection criteria have been more strictly adhered to during the last ten years, which coincides with the observed improvement in survival.

### F117

### FOLLOW-UP AFTER CURATIVE SURGERY FOR COLO-RECTAL CARCINOMA: RANDOMIZED COMPARISON WITH NO FOLLOW-UP

K.-G. Tranberg, B. Ohlsson, U. Breland, H. Ekberg, H. Graffner
Departments of Surgery, Lund University, Lund, Sweden and Helsingborg Hospital, Helsingborg, Sweden

This study investigated the value of intense follow-up after curative surgery of cancer in the colon or rectum. 107 patients were randomized to no follow-up (control group, n=54) or intense follow-up (follow-up group, n=53) after surgery and early postoperative colonoscopy. Patients in the follow-up group were followed at frequent intervals with clinical examination, rigid proctosigmoidoscopy, colonoscopy, computed tomography of the pelvis (in patients operated with abdominoperineal resection), pulmonary x-ray, liver function tests, and determinations of carcinoembryonic antigen (CEA) and faecal hemoglobin. Follow-up ranged from 5.5 to 8.8 years after primary surgery. Tumour recurred in 18 patients (33 %) in the control group and in 17 patients (32 %) in the follow-up group. Reresection with curative intent was performed in three patients in the control group and in five patients (four of whom were asymptomatic) in the follow-up group. In the follow-up group two asymptomatic patients with elevated CEA levels were disease-free three and five and a half years after reresection and were the only patients apparently cured by reresection. No patient underwent surgery for metastatic disease in the liver or lungs. Symptomatic metachronous carcinoma was detected in one patient (control group) after three years. Five-year survival rate was 67 % in the control group and 75 % in the follow-up group (p> 0.05); the corresponding cancer-specific survival rates were 71 % and 78 %, respectively. It is concluded that intense follow-up after resection of colorectal cancer did not prolong survival in this study.

### F118

### PERCUTANEOUS FINE NEEDLE BIOPSY OF LIVER TUMOURS- IMPACT ON PATIENT MANAGEMENT AND OUTCOME

K.-G. Tranberg, B. Ohlsson, J. Nilsson, M. Åkerman, U. Stenram
Departments of Surgery and Pathology, Lund University, Lund, Sweden

Percutaneous fine needle aspiration biopsy followed by histopathological examination was carried out in 208 patients with liver tumours. Indications for aspiration biopsy were: suspect recurrence of previously resected primary (n=63), tumour of unclear origin or type (n=144) or ascites (n=1). The final diagnosis was primary liver cancer (n=103), colorectal liver cancer (n=47), non-colorectal liver metastases (n=49), benign tumour (n=5) and no tumour (n=4). Compared to the final histopathological diagnosis, cytological examination resulted in: correct diagnosis in 85 patients (41 %), correct classification as benign or malignant in another 72 patients (35 %), suspect malignancy in 10 patients (5 %) with malignant tumour, erroneous classification of a malignant lesion as benign or a benign lesion as malignant in 24 (12 %) and 4 (2 %) patients, respectively. Cytology was inconclusive in 13 patients (6 %). Considering the information available when aspiration biopsy was carried out, cytology provided new and correct information in 75 patients (36 %), confirmed a previous suspicion in 81 patients (39 %), gave no conclusive information i 28 patients (13 %) and incorrect information in 24 patients (12 %). Cytological diagnosis was valuable for planning further investigation and treatment in 58 patients (28 %) but was of no or doubtful importance in 139 patients (67 %). In 11 patients (5 %) it led to repeat aspiration biopies without definite diagnosis, delay of treatment or unnecessary investigations or operations. Implantation metastasis was recorded in 7 patients (3 %). It is concluded that fine needle biopsy is of limited value in the management of liver tumours and that the benefits and risks involved do not justify its use in candidates for curative resection.

### F119

### CHEMOEMBOLIZATION AND ALCOHOLIZATION FOR HEPATOCELLULAR CARCINOMA IN CIRRHOSIS BEFORE LIVER TRANSPLANTATION: ASSESSMENT OF EFFICACY ON SPECIMENS AND CLINICAL OUTCOME.

R. Troisi; U.J.Hesse; P. Pattyn; M. Praet*; L. Defreyne $ and B. de Hemptinne. Departments of: Surgery and Liver Transplantation, * Pathology, $ Radiology, University Hospital Ghent, Belgium.

Liver transplantation ( LT ) has been widely accepted in case of irresectable HCC because of the severity of the underlying cirrhosis, the location of the tumour within the liver hilus, or the extent of the liver involvement, but the benefit of pretransplantation tumor treatment with chemoembolization ( TAE ) and/or percutaneous alcohol injection ( PEI ) is still under investigation. We report our results after preoperative treatment with TAE and PEI, regarding the clinical outcome and the recurrence rate after LT for HCC. **Material and methods:** 17 patients underwent total hepatectomy and orthotopic liver transplantation for irresectable HCC on cirrhosis. There were 15 males and 2 females, whose ages ranged from 39 to 63 years (median age +/- sd = 54 +/- 5.7 ). All the patients were cirrhotics, with a prevalence of HCV infection in all but two, one affected by alcoholic cirrhosis and the other by cholestatic liver disease. The tumour nodules were solitary in 9 cases, multiple in 8, with a mean size of 28 +/- 15 mm, ranging from 10 to 70 mm. TAE and PEI were performed in all but two patients, and repeated every 2-3 months ( TAE until 5 time ) and every 2-3 weeks ( PEI until 6 time ). **Results:** the serum a-fetoprotein level was useful for evaluating the therapeutic effect, showing an important decrease in patients with a level higher than 100 ng/ml. Mild to severe local pain occurred immediately after PEI and high fever ( >38 °C ) developed after TAE in half of the patients without any complications. Extensive tumour necrosis ( > 90% ) was seen in 9 patients, but tumour could not be found in 3 despite a positive preoperative liver biopsy (in two) and a most suggestive preoperative radiographic imaging in the other one. We lost four patients: two because of tumour recurrence at 8 and 37 months after LT, one because a fulminant recurrence of HBV infection after retransplantation and the other one because of cerebral bleeding, respectively at 16 and 3 months after LT. The median follow-up is 20 +/- 15 months ( range 6-48 ), with a total recurrence rate of 11%. The overall actuarial survival and tumour free survival is 76% at four years.
**Conclusions:** in our opinion in selected patients treated with combined adjuvant therapeutic options now available such as TAE and PEI, long-term survival could improve even further if extensive tumor necrosis can be obtained. Efforts should be made to treat the patients before transplantation to reduce tumour volume and hopefully reduce micrometastasis.

### F120

### INTRAARTERIAL vs. SYSTEMATIC CHEMOTHERAPY FOR NON OPERABLE HEPATOCELLULAR CARCINOMA

E.Tzoracoleftherakis, D.Kyriakopoulou, S. Kakkos, A. Dimakopoulos, C. Mitsoulas
Department of Surgery, University of Patras, Patras, Greece

The efficacy of intraarterial infusion of adriamycin in inoperable hepatocellular carcinoma remains controversial. A series of 68 patients with inoperable hepatocellular carcinoma (mean age 63 years, range 49-75) were studied prospectively. The patients were randomised to receive adriamycin 50 mg /m$^2$, either as a bolus infusion via an implantable intrarterial catheter and port inserted in the hepatic artery (31 patients) or as systemic chemotherapy with a bolus injection (37 patients), every 21-28 days. The Karnofsky scale of the patients at diagnosis was at least 70 %. Complication resulted in the arterial infusion group were: infection of the port pouch 2, catheter migration 1, hepatic artery aneurysm, myelosupression 1. Five patients died without receiving chemotherapy. Of the remaining 26 patients, 17 (65.4 %) had a subjective improvement, while the 15 (57.7 %) presented an objective improvement. The Karnofsky scale showed improvement in 8 patients (30.8 %) while 10 (38.5 %) remained stable. The survival was 69.2 % (18 patients) in 6 months and 11.5 % (3 patients) in 12 months. The mean survival was 7.1 months (2-16 months). In the systemic chemotherapy group major complications included 3 cases of myelosuppresion. Three patients discontinued chemotherapy. Of the remaining 34 patients, 15 (44.1 %) had a subjective improvement while the 15 (44.1 %) presented an objective improvement. The Karnofsky scale was improved in 7 patients (20.6 %) while 11 (32.4 %) remained stable. The survival was 55.9 % (19 patients) in 6 months and 5.9 % (2 patients) in 12 patients. The mean survival was 6.5 months (1-13 months). There was a difference in the survival of the 2 groups. There was equal decreased survival of the cirrhotic patients in both groups. The quality of life remained at an acceptable level during the treatment and until the death.
In conclusion, a slight superiority of intraarterial chemotherapy against the systematic one is anticipated.

### F121

**LIVER RESECTION FOR HEPATOCELLULAR CARCINOMA ON CIRRHOSIS.**

A.Uzzau, F.Bresadola, M.Sistu, N.Cautero, M.G.Ventroni, G.Anania, S.Intini, P.Soro
Department of Surgery, University of Udine, Udine, Italy

A retrospective study was carried out on 53 liver resection for HCC performed over a six years period (June 1989-June 1995). Seventy-two consecutive patients were enrolled as possible candidate to resection 12 of which (16.6%) were escluded from a surgical therapy as a consequence of the following criteria: multifocality, portal thrombosis, and metastases. Out of 60 patients suitable for surgical treatment 53 underwent liver (resection index of 88.3%). Only one patient with haemoperitoneum underwent emergency exploration and resection. Istologically proven cirrhosis were present in 95.8% of the patients and Child-Pough's class was A in 63%, B in 28.2% and C in 8.8% respectively. In the last four years thirtyfive patients (66%) were prospectively evalueted for surgical risk by mean of the Okamoto index and all resulted under the critical value of 50% (mean 29.9%, SE 6.8). Intraoperative US exploration was always employed and all resections were done under transparenchymal approach by Kelly-fracture. Pringle clamping was used in 62.2% (with mean clamping time of 29.5 min., SE 2.85, range 8-74).
Histological studies showed HCC in 45 patients, cholangiocarcinoma in 6 and fibrolamellar variant in 2. In 62.2% cases HCC was single nodular whereas in 37.8% was multinodular. The mean diameter of the lesions was 6.4 cm (range 1-17, SE 0.4). In 23% of the cases the tumor was encapsuled and a free margin of resection was present in 73%.
In this series morbidity was 37.7% and two patients died within 30 days, with an overall mortality rate of 3.7%. Excluding deaths within one month, long term survival rates were investigated in relation to various prognostic factors using Kaplan-Meier method.
The overall survival at 1, 2, 3 and 5 years was 67%, 54%, 43% and 21%. Seventheen patients (35.4%) had recurrences during the first two years and were treated by TACE or percutaneous alcool injection. Statistical analysis of prognostic factors showed that a free resection margin and a single lesion was associated with a better survival (53% survival at 5 years for single lesion and 36.8% for free margin resection vs 22.5% for multiple lesions and 0% for patients without free margin resection)..

### F122

**WARM (20°C) FLUSH OF COLD STORED LIVERS PRIOR TO REPERFUSION ATTENUATES PRESERVATION INJURY.**

B.A. van Wagensveld, M.E. Reinders, T.M. van Gulik, W.M. Frederiks[1], R.J.A. Wanders[2] and H. Obertop. Depts of Surgery, [1]Cellbiol. and Histol. and [2]Biochem. Academical Medical Center, Amsterdam, the Netherlands.

In a previous study, we have shown a beneficial effect of intermittent warm (20°C) rinsing on microcirculatory injury in cold stored rat livers. Aim of this study was to evaluate the temperature effect (4°C vs 20°C) of a single flush preceding reperfusion in a rat liver preservation model. Preservation injury was assessed by measuring AST, LDH, hyaluronic acid (HA) and purine nucleoside phosphorylase (PNP). HA is metabolized by the sinusoidal endothelial cells (SEC) of the liver; uptake or release therefore reflects SEC function or death, resp. Release of PNP is associated with SEC death. *Methods.* Rat livers were washed out in situ via the portal vein with UW solution (4°C) and after hepatectomy, were stored at 4°C. Immediately after hepatectomy (t0, control livers) and after 8hr, 16hr and 24 hr cold ischemic time (CIT), resp, the livers were reperfused for 90 mins via the portal vein with oxygenated Krebs Henseleit buffer (37°C, without albumin) containing HA (34-55 µgr/L). At the end of CIT, prior to reperfusion, the livers were flushed with 10 ml UW solution, either at 4°C or 20°C. During reperfusion, bile production was monitored and HA (radiometric assay), PNP (fluorescence spectroscopy), LDH and AST were measured in the reperfusion medium. *Results.* Mean bile production was highest in control livers (1.88 ± 0.31 µl/gr/min). After 8hr CIT, significantly more bile was produced by livers preflushed with 20°C UW, as compared to those preflushed with 4°C UW (1.61 ± 0.13 µl/gr/min vs. 0.84 ± 0.15 µl/gr/min, p=0.006). After 16hr and 24hr CIT, more bile was produced in livers preflushed at 20°C, however not significantly different (1.03 vs. 0.99 µl/gr/min at 16h/20°C and 16h/4°C, resp., and 1.30 vs. 0.94 µl/gr/min at 24h/20°C and 24h/4°C, resp.). HA uptake after 90 min reperfusion in the contol group (t0) was 49%. After cold storage, progressive leakage of HA was seen in all groups, except for the 8h/20°C livers in which HA was taken up. PNP and AST release increased progressively in all groups with prolonged CIT, without showing differences between the 4°C and 20°C flush groups. LDH release increased with CIT in all groups, and was not significantly different except for the 8h/20°C and 8h/4°C groups (38.0 ± 9.2 U/l and 80.6 ± 16.8 U/l, resp.; p=0.004). *Conclusion.* Warm (20°C), single flush of cold stored livers prior to reperfusion attenuates preservation injury. This effect was most evident after 8h storage, compared to 16h and 24h storage, suggesting that the beneficial effect of warm, pretransplant flush solutions decreases with prolonged preservation times.

### F123

**TRANSPLANTATION FOR ALPHA 1 ANTITRYPSIN (A1AT) DEFICIENCY.**

G Vennarecci, T Ismail, BK Gunson, *D Kelly, AD Mayer, JAC Buckels, E Elias, P McMaster.
The Liver Unit, Queen Elizabeth Hospital and *The Children's Hospital, Birmingham, UK.

End-stage A1AT deficiency related-liver disease is the most common genetic cause for orthotopic liver transplantation (OLT) in children, although a rare indication in adults. The pathophysiology of the disease is poorly understood and the natural history, disease progression and outcome is unpredictable. OLT is the only therapeutic option for end stage liver disease. Of 1000 OLT performed between 1982-1994, 39 transplants (35 patients; 13 children, 22 adults) were for A1AT deficiency. Eleven children presented with features of neonatal hepatitis and intrahepatic cholestasis and two with hepatosplenomegaly. Jaundice failed to relapse in five and portal hypertension was present in all cases. Liver disease in adults presented with sudden onset and a progressive course leading to liver failure. Eight (2 children) patients had obstructive respiratory disease at time of OLT. All children were homozygous (PiZZ) with a median serum A1AT level of 0.45 g/l (range: 0.4-0.95 g/l). Two adult patients were PiZZ, 3 were PiMM with the remaining being heterozygous. The median serum A1AT level for adults was 1.10 g/l (range: 0.2-2.27 g/l). Fifteen OLT (13 patients, 10 male, 4 reduced grafts) and 24 OLT (22 patients, 19 male) were performed in children and adults respectively. Two children were retransplanted for chronic rejection and two adults for hepatic artery thrombosis and ischaemic graft failure. Current 1 year post transplant survival figures are 73% for adults and 87.5% for children. Replacement of the cirrhotic liver results in acquisition of donor phenotipe, a rise in serum levels of A1AT and apparent prevention of associated disease. All survivors have normal liver function, good quality of life with no liver disease recurrence or lung disease progression.

### F124

**LIVER TRANSPLANTATION FOR PRIMARY HEPATIC MALIGNANT DISEASE**

**Emilio Vicente M.D. Victor S. Turrión M.D.. Javier Nuño M.D. Fernando Pereira M.D. Nicolás P. Mora M.D. Julian Ardaiz M.D. Valentin Cuervas-Mons M.D. Antonio L. Sanromán M.D. Angel Candela M.D.**

**Liver Transplantation Unit. Hospital Ramón y Cajal. Clínica Puerta de Hierro. Madrid. Spain**

Liver transplantation is now widely accepted as a worthwhile treatment for selected patients with primary liver cancer. At Hospital Ramón y Cajal and Clínica Puerta de Hierro, we performed 340 liver transplantation between March 1986 and December 1995. 31 patients underwent transplantation for primary malignant disease. 23 patients had hepatocellular carcinoma, including 1 of the fibrolamellar type, 7 were diagnosed of cholangiocarcinoma and 1 of hepatoblastoma. Of the 31 patients, 6 were female and 25 were males. The ages of the patients ranged from 12 to 63 years. 4 of whom were less than 20 years old.

3 patients (9%) died in the first 3 months from complication of transplantation. Of the remaining patients who survived more than 3 months, 4 (14%) developed recurrent disease, diagnosed 7 months to 9 months after liver replacement. The only patient treated for fibrolamellar hepatoma is alive without tumor at more than 8 years after transplantation.

CARCINOMA HEPATOCELLULAR

20 of 22 patients with carcinoma hepatocellular had associated underlying liver disease. These patients were stratified according to the TNM classification: Stage I: 3 patients, Stage II: 10 patients, Stage III: 6 patients and Stage IVa: 3 patients. Actuarial survivals for all 22 patients with carcinoma hepatocellular at 1 year, 2 years and 3 years were 57%, 50% and 41%, respectively. Stage I and II (5 years actuarial survival: 56%) and incidental hepatoma (4 years actuarial survival: 77%) have a good prognosis after transplantation.

CHOLANGIOCARCINOMA

3 of the 7 patients who underwent liver transplantation for cholangiocarcinoma had nodal involvement and/or perineural and vascular invasion with infiltration of tissue around biliary tract. The 7 year actuarial survival was 66%. 2 patients survived tumor free for more than seven years. In 1 patient, recurrence occurred in the trasplanted liver, seven months after transplantation.

We conclude that liver replacement for malignant hepatic neoplasms should be considered for a selected group of patients with unresectable lessions and without evidence of extrahepatic disease.

### F125

### RESULTS OF 118 LIVER RESECTIONS FOR METASTASES OF COLORECTAL CARCINOMA

I.Vogel, D. Henne-Bruns, B.Kremer
Department of Surgery, University of Kiel, Kiel, Germany

Between 1985-1994 118 liver resections in 113 patients with liver metastases of colorectal carcinoma were performed by our group at the University hospitals of Hamburg and Kiel.
The data of 106 cases were analysed in a retrospective study regarding the following prognostic factors:
age, sex, localisation and stage of primary tumor, size, number and localisation of metastases and extent and radicality of the liver resection.
The results showed, that a significant influence regarding the long term survival rate could only be archieved in patients, in which a R0-resection with a histollogically proven tumor free margin of more than 1 cm was performed (p= 0,0087).
For patients in this group (n=46) a 5-year-survival rate of 40% was observed. In patients with tumor free resection margins less than 1 cm (n=38) the 5-year-survival rate was only 10% and comparible to patients with R1-resections (n=7). None of the patients with R2-resections (n=15) survived 5 years.
Other significant prognostic factors were the number of metastases and their size. Patients with more than 3 metastases or metastases > 10 cm had a significant shorter survival (p= 0,0467/ p= 0,0472) than patients with 1-3 metastases or metastases < 10 cm.

Our data support the importance of a sufficient safty margin in liver resections for colorectal metastases.

### F126

### TRANSPLANTATION OF AUTOLOGOUS CULTURED HEPATOCYTES IN PRIMATE: A MODEL FOR GENE THERAPY OF CONGENITAL HYPERCHOLESTEROLEMIA.

C. Vons, D. Mahieu, M. Andréoletti, F. Bargy, A. Weber, F. Capron, D. Franco.

Services de Chirurgie, Hôpital Antoine Béclère et Hôpital St Vincent de Paul, ICGM Hôpital Cochin, Paris, et Service d'Anatomopathologie, Hôpital Antoine Béclère, Clamart, France.

A possible treatment of autosomal hypercholesterolemia is the transplantation of autologous hepatocytes transfected with the gene coding for the LDL receptor. The purpose of this work was to assess the feasibility of preparing a large amount of hepatocytes from primate liver and to transplant them within the portal system. Nine Macacus Cynomolgus females weighing 3 to 6 kgs were used. The left posterior liver lobe was carefully removed under general anesthesia. This lobe represents about 30% of the total liver mass. During the same operation the catheter of an intravenous infusion chamber was inserted in the inferior mesenteric vein with its tip positioned at the confluence with the splenic vein. The chamber was inserted subcutaneously on the chest. Postoperative (5 days) portography through the catheter demonstrated the patency of the portal system in all cases. Hepatocytes were prepared from the resected liver lobe by perfusion of collagenase. Primate hepatocytes were cultured during 5 days in DMEM. They were then slowly infused into the portal system ($10^8$ cells in 20 ml of saline) through the catheter inserted into the inferior mesenteric vein. There were no postoperative death and infusion of cultured hepatocytes was well tolerated. Liver biopsies were taken from each remaining liver lobe under general anesthesia 7 and 14 days after hepatocytes transplantation. Histological examination of specimens showed hepatocytes in the sinusoids lumen of the liver. Our model represents a good model for transplantation of autologous hepatocytes in primates.

### F127

### PROLONGED INTERMITTENT ISCHEMIA FOR HEPATIC RESECTION OF THE CIRRHOTIC LIVERS - EFFECTS AND LIMITS

Cheng-Chung Wu, Chih-Jen Huang, Dah-Cherng Yeh, Tse-Jia Liu, and Fang-Ku P eng, Taichung Veterans General Hospital, Taichung, Taiwan

Clamping of hepatic pedicle (Pringle's maneuver) is a useful technique for reducing operative bleeding in hepatic resection. Although a normal liver can tolerate continuous warm ischemia for more than 60 min, the upper time limit for a cirrhotic liver is still undefined. On the other hand, intermittent clamping of liver pedicle is reported to be better than continuous clamping. To determine the effects and limits of prolonged intermittent liver ischemia on cirrhotic liver, we retrospectively reviewed our results of hepatic resection on cirrhotic patients using this technique. During operation, liver parenchymal dissection was performed under repeat clamping of liver pedicle for 15 min and declamping for 5 min. Based on the total ischemic time, these patients were divided into three groups: group A, < 40min (39 patients), group B 40-80 min (28 patients) and group C > 80min (16 patients). The non-tumorous liver parenchyma was preserved as much as possible. Longer total ischemic time was required in the resection of larger tumor(p=0.002), and was associated with longer operating time, greater amount of operative blood loss and blood transfusion (p<0.001). It also resulted in higher elevation of postoperative levels of serum transaminases and lactic dehydrogenase (p<0.001). Two patients in group A died of operation. The operative morbidity, mortality and late hepatic failure rate is not different among the three groups. The longest total ischemic time in this series is 204 min. However, the uppermost time limits for warm ischemia on cirrhotic liver requires a further investigation.

### F128

### THREE DACADES EVOLUTION IN SURGERY FOR PRIMARY LIVER CANCER

X.D. Zhou, Z.Y. Tang, Y.Q. Yu, Z.C. Ma
Liver cancer Institute, Shanghai Medical University, Shanghai 200032, China

During 1958-1994, 2018 patients with pathologically proven primary liver cancer(PLC) were explored and 1380 patients(68.4%) underwent liver resection. Comparison with PLC data of 1958-1970(n=125), 1971-1982(n=359), and 1983-1994(n=1534), encouraging changes in the prognostic pattern were observed, overall 5-year survival being 6.9%, 18.0%, and 46.3%, respectively, and 10-year survival being 6.0%, 11.8%, and 33.4%, respectively. The major factors that related to these encouraging results may be as follows; (1) A remarkable increasing proportion of small PLC (≤5cm), 3.2% (4/125) 18.4%(66/359), and 33.8%(519/1534), respectively. (2) A remarkable increasing proportion of curative resection, 40.6%(26/64), 69.5%(121/174), and 88.6% (1012/1142), respectively, (3) A remarkable increasing proportion of limited resection, 6.3%(4/64), 43.7% (76/174), and 74.8%(854/1142), respectively, which has resulted in decreasing operative mortality, 23.4% (15/64), 4.6%(8/174), and 1.5%(17/1142), respectively. (4) The increase number of re-operation for recurrent PLC(0, 27, and 114 patients) and cytoreduction and sequence resection for initially unresectable PLC(0, 5, and 67 patients) have added weight to improving survival further. These results indicate that early detection and curative resection are the principal factors influencing long-term survival; limited resection instead of lobectomy is the key to decrease operative mortality; re-operation for subclinical recurrence remains an important approach towards further survival prolongation after curative resection of PLC; cytoreduction and sequence resection may provide a hope for survival prolongation in some patients with unresectable PLC.

### CHANGES OF SPLANCHNIC ARTERIAL RESISTANCES SIX MONTHS AFTER LIVER TRANSPLANTATION.

G.Zironi, F.Piscaglia, *GL.Grazi, *E.Jovine, *M.Morganti, N.Venturoli, C.Serra, M.Valgimigli, G.Donati, *A.Mazziotti, *A.Cavallari, L.Bolondi. Istituto di Clinica Medica e Gastroenterologia, * Clinica Chirurgica II, Univ Bologna, ITALY

Introduction: liver cirrhosis is characterized by increased intestinal blood flow with reduced arterial resistances. On the contrary hepatic, splenic and renal artery resistances are reported to be increased. Aim: to assess the effect of orthotopic liver transplantation (OLT) on splanchnic hemodynamic changes induced by liver cirrhosis. Materials and methods: 20 patients submitted to OLT for end stage liver cirrhosis (m=14; with ascites=12; 14 HCV+ and/or HBV, 3 alcohol, 1 PSC, 1 PBC, 1 Byler's disease), when first in the waiting list, underwent an abdominal Doppler US exam to assess resistance indexes (RI) (an indirect measurement of resistance) in the superior mesenteric, intrahepatic, intrasplenic and renal interlobular arteries and portal blood flow velocity in the right portal branch. The same measurements were repeated at six months in 17 patients (none with ascites, two suffering from rejection, two retransplantated). The 3 other patients had died. Student t-test for paired data was used for statistical analysis. Results: at six months portal blood flow velocity increased from 14.4 to 28.6 cm/sec ($p<0.01$) and splenic artery RI decreased from 0.601 to 0.525 ($p<0.025$). RI changed from 0.678 to 0.644 in the intrahepatic artery, from 0.651 to 0.618 in the renal artery and from 0.810 to 0.841 in the superior mesenteric artery: these changes didn't reach, however statistical significance. Conclusions: OLT reverts the splenic artery RI increase. Persistance of a mild overflow in the splanchnic vascular bed after OLT is suggested by the marked increase of portal flow velocity and the incomplete reversal of superior mesenteric artery dilation (normal RI values in our experience=0.88). The contribution of the persistance of collateral pathways to this event should be investigated. Morevoer the high portal flow, probably stimulating the arterial buffer response with arterial costriction, could hide significant reduction of hepatic artery RI. Renal artery RI tends to decrease after OLT, probably not reaching statistical significance, only because in this series of patients the preOLT levels were already in the normal range (<0.70).

### ORAL PRESENTATIONS

### Topic: PANCREAS

### F130

### PANCREATITIS-INDUCED ACUTE LUNG INJURY AND THE COMPLEMENT SYSTEM

J. Acioli*, M. Isobe**, S.Kawasaki*

*First Department of Surgery and **First Department of Medicine
Shinshu University School of Medicine, Japan

Objective: We analyze, using a cerulein-induced acute pancreatitis model, the role of the activated complement system (ACS) in PMNs priming and lung sequestration, event related to the acute lung injury and ARDS occurring in this disease. Participation of the ACS at an early stage is evaluated using a specific receptor of the complement system activated components. Material and methods: Supramaximal doses of the secretagogue cerulein were infused for 4h to produce a mild edematous pancreatitis in Lewis rats. Soluble complement receptor, sCR1 (BRL55730-Smithklein Beecham Pharmaceuticals), was used to block the complement cascade. Rat lungs were resected at the end of the experiment for measurement of myeloperoxidase (MPO), a marker of PMN accumulation. Mac-1 expression on surface of leukocytes ("in vitro" and "in vivo' flowcytometric studies) was used to access activation status. Bronchoalveolar lavage (BAL) and wet:dry weight ratio indicated the degree of lung injury. Histological studies were performed in HE-stained specimens. Results: High MPO levels were found in rat lungs 4h after induction of pancreatitis, indicating a high concentration of PMNs at this time (OD460=1.54+/-0.13, n=8). This data was confirmed by histological examination. Treatment with sCR1 before pancreatitis induction significantly reduced the pulmonary concentrations of PMN (OD460=1.00+/-0.16, n=8). Also Mac-1 upregulation (activated pattern) was observed in circulating PMNs of rats with pancreatitis and in neutrophils incubated with serum of pancreatitis group rats as early as 2h after induction. This could be reversed by addition of sCR-1. During the time observed it could be seen no evidence of lung injury as shown by no change in wet:dry weight ratio and BAL fluid microscopy. Conclusion: 1)Complement system is responsible for neutrophils priming and consequent lung sequestration early in the course of pancreatitis in this model, and this could be efficiently reversed by the use of sCR-1. 2)No lung injury could be demonstrated in the period observed, suggesting that the function enhancement of the primed neutrophils occurs at a later stage probably under influence of another inflammatory mediator.

### F131

### PANCREATIC ENDOTHELIAL BARRIER ALTERATIONS IN EXPERIMENTAL ORGAN DYSFUNCTION

R. Andersson, X.M. Deng, Z.W.Sun, X.D. Wang
Department of Surgery, Lund University, Lund, Sweden

Alterations in pancreatic endothelial barrier integrity results in leakage of blood components, tissue edema, hemorrhage and necrosis. Over-activation of macrophages might be one of the responsible factors initiating pancreatic endothelial barrier compromise. In the first experiment, three kinds of macrophage stimulators (zymosan, concanavalin A (CCV) and thioglycolate medium (TM)), were chosen to evaluate the influence on pancreatic endothelial barrier integrity 24 hours after intraperitoneal challenge with 0.25 and 0.50 mg of the various substances per g bodyweight (BW). Zymosan induced a significant increase in pancreatic tissue water content, interstitial fluid volume and extravascular protein volume and a decrease in intravascular plasma volume as compared to controls. The administration of TM and CCV had none or minor effects on the endothelial barrier and induced an increase in reticuloendothelial system (RES) function, while zymosan resulted in a compromise of RES. In the second experiment, the dynamics of pancreatic endothelial alterations induced by various doses of zymosan by determining endothelial permeability 1 to 24 hours after challenge. Pancreatic endothelial injury was evident from one hour increasing by time. In the third experiment, possible mechanisms by which zymosan induced endothelial injury was investigated. Pretreatment with dimethyl sulphoxide (a scavenger), indomethacin (a cyclooxygenase inhibitor), and verapamil (a calcium channel blocker) partly prevented the increase in endothelial barrier permeability. Pretreatment with N-acetyl-L-cysteine, a scavenger of multiple oxygen free radicals completely protected from endothelial injury. Pretreatment with allopurinol (xanthine oxidase inhibitor), L-NNA and L-NAME (nitric oxide inhibitors), and pargyline (monoamine oxidase inhibitor) had no significant effects.
Thus, zymosan resulted in a compromised pancreatic endothelial barrier. Over-activation of macrophages might play an important role in the etiology of pancreatic dysfunction. Multiple factors including a variety of oxygen free radicals are probably involved.

### F132

### OPEN MANAGEMENT OF THE ABDOMEN FOR NECROTIZING PANCREATITIS

K. Bosscha, P.F. Hulstaert, A. Hennipman, H.G. Gooszen, Th.J.M.V. van Vroonhoven and Chr. v.d. Werken
Department of Surgery, University Hospital Utrecht, The Netherlands

Purpose of study. A prospective study was performed to evaluate 'open management of the abdomen' as the treatment for acute necrotizing pancreatitis, a disease with high morbidity and mortality to even 70 per cent. Systemic effects of infected necrosis are the main causes of death. Necrosectomy and drainage are the main elements of surgical treatment. 'Open management of the abdomen' with scheduled relaparotomies in the SICU was adopted as our standard treatment in case of extensive areas of infected necrosis.
Patients. From June 1988 through January 1995, 28 patients were treated along these lines. The mean age was 52 years, range 29-76 years. The cause was gallstone disease in 11 patients; 8 patients had alcohol-induced pancreatitis. In 9 patients the underlying cause could not be determined. The Ranson-score before the start of the 'open management of the abdomen' was 4.9 ± 0.3 (mean ± SEM), whereas the Imrie-score was 4.0 ± 0.2. The APACHE II-score was 18.4 ± 1.3 and the SAP-score was 14.4 ± 0.6. Preoperatively computed tomography confirmed clinical diagnosis in 20 patients; in 8 patients extensive areas of necrosis were detected at explorative laparotomy who needed exploration anyway.
Results. The mean number of relaparotomies performed in the SICU was 17, range 3 to 70. Colonic perforation led to subtotal colectomy with end-ileostomy in 5 patients. Nine abscesses were drained percutaneuosly. Seven fistulas developed in 7 patients: 6 closed spontaneously, one needed a diversion ileostomy. In 14 patients severe bleeding occurred without mortality. Eighteen patients developed MOF; in total 11 patients died (39 per cent). Probably, in two patients MOF was directly related to progressive necrosis of the bowel. Seven patients needed dialysis. The mean duration of stay in the SICU was 27 days, range 5 to 101 days; the mean duration of hospital stay was 69 days, range 5 to 171 days.
Conclusion. Despite necrosectomy and drainage by means of prolonged 'open management of the abdomen', mortality of acute necrotizing pancreatitis with extensive areas of necrosis remains high. The treatment strategy as described has considerable additional morbidity of the gastrointestinal tract.

### F133

### PROGNOSTIC INDICATORS FOR SURVIVAL AFTER RESECTION OF PANCREATIC ADENOCARCINOMA

M. Calgaro, R. Polastri, F. Bertolino, G. Mensa, L. Capussotti.

Department of Surgery, Ospedale Mauriziano, Largo Turati 62 - Torino, Italy.

We have rewiewed our experience with pancreatic resection for exocrine pancreatic cancer in order to evaluate short- and long-term survival. A number of variables were evaluated to identify factors predictive of long-term survival. From January 1982 to December 1994 we performed 92 pancreatic resections for adenocarcinoma of exocrine pancreas. Sixty-one were males and 31 females, mean age was 62±11 years (range:34-82). The operative procedures consisted of 66 pancreaticoduodenectomies (PD), 7 distal pancreatectomies and 19 total pancreatectomies. Forty-three of the PD included a distal gastrectomy and 23 were pylorus preserving; 11 patients had a pancreatogastrostomy. Twenty-two patients (23.9%) had associated vascular resections. Survival was analyzed by the method of Kaplan-Meier. Differences in survival among variables were compared with the log-rank test. Operative mortality rate (60 days) was 6.5% (6 patients). Major morbidity related to operative procedure was seen in 18.4% of patients(17 patients). Actuarial survival rate at 1, 5 and 10 years was 51.7%, 10.5% and 5.6% respectively. The factors significantly influencing a poor prognosis were: neoplastic invasion of preaortic lamina (p=0.006) and metastatic lymph node involvement (p=0.002). Vascular invasion, age >70 years and perioperative blood transfusions were not associated with a worse prognosis.
In conclusion: according to many data in literature, preaortic lamina invasion and lymph nodes metastases are the main prognostic factors influencing survival.

F134

**STAGING LAPAROSCOPY WITH LAPAROSCOPIC ULTRASONOGRAPHY: OPTIMIZING RESECTABILITY IN HEPATOBILIARY AND PANCREATIC (HBP) MALIGNANCY.**

**MP Callery, GM Doherty, JA Norton, NJ Soper, and SM Strasberg, Department of Surgery, Washington University School of Medicine, St. Louis, Missouri, U.S.A.**

**Open laparotomy has traditionally been required to stage hepatobiliary and pancreatic (HBP) cancers accurately. For unresectable patients, costs and morbidity have been high. Today, laparoscopy alone or combined with laparoscopic ultrasonography (LUS) is being examined for its value in defining the extent of malignancy. We have analyzed our routine implementation of this new staging technique in our HBP Center. METHODS: Staging laparoscopy (SL) with LUS was performed in 50 consecutive patients with HBP malignancies. All patients were considered to have resectable tumors as determined by traditional preoperative staging modalities. Primary tumors were located in the liver (n=7), biliary tract (n=11), or pancreas (n=32). Preoperative staging studies included computed tomography (96%) alone or combined with antegrade/ retrograde cholangiography (72%), arterioportography (22%) and magnetic resonance imaging (14%). An average of 2.7 preoperative studies per patient were required to initially determine resectability. SL-LUS was performed to detect occult hepatic, lymphatic or peritoneal metastases, and to detect local tumor invasion rendering the tumor unresectable. SL-LUS was performed under the same anesthetic as the subsequent laparotomy (70%) or as a separate staging procedure (30%). RESULTS: SL-LUS predicted a resectable tumor in 28 patients (56%). At laparotomy, 26 of 28 were actually resectable indicating a false-negative rate of 4%. SL-LUS indicated unresectability in 22 patients (44%). SL alone demonstrated previously unrecognized occult metastases in 11 patients (22%). For an additional 11 patients (22%) in whom SL alone was negative, LUS determined unresectability as a result of vascular invasion (n=5), lymph node metastases (n=5), or intraparenchymal hepatic tumor (n=1). All cases of unresectability due to vascular invasion were validated by laparotomy. 5 of 6 lymph node or hepatic metastases were proven histologically by LUS-guided needle biopsy rather than laparotomy. CONCLUSIONS: SL with LUS optimizes patient selection for curative resection of HBP malignancies. Unnecessary laparotomy can be safely avoided in unresectable patients reducing costs and morbidity.**

F135

IS THE PREOPERATIVE DIAGNOSIS OF A BENIGN AMPULLOMA SAFE?

O. Chapuis, A. Sauvanet, Ph.Ponsot, P.Hammel, P.Bernades, J. Belghiti.

Fédération d'Hépatogastroentérologie Médico-Chirurgicale, Hôpital Beaujon, Université Paris VII, Clichy, France.

Malignant ampullomas can be curatively treated by pancreaticoduodenectomy (PD). A local excision can be proposed in case of benign ampulloma. The aim of this study was to evaluate the value of side-viewing duodenoscopy (SVD) with biopsies, endoscopic sphincterotomy (ES), and endoscopic ultrasonography (EUS) for preoperative diagnosis of benign and malignant ampullomas.

Material et methods : From October 1989 to September 1995, 26 patients with ampulloma were explored preoperatively by SVD - including ES in 9 cases - and EUS. The papilla of Vater was always explored at SVD and forceps biopsies were performed in all patients except one with a typical malignant tumour. EUS evaluated the T stage of the TNM classification in all patients except six because of a previous ES (n=5) and difficulty to localize the papilla (n=1). The N stage of the TNM classification was evaluated by EUS in all cases. A curative resection was always performed : 2 local excisions of the ampulla (one adenoma with low-grade dysplasia and one xanthoma), and 24 PD for 20 carcinomas (including 9 (45%) N1) and 4 benign lesions.

Results : At SVD, papilla was both ulcerated and protruding into the duodenal lumen in 10 cases, prominent and smooth in 15 cases, and normal in one case. Histologic examination of the 25 biopsies revealed malignancy in 10 cases (always confirmed by pathological examination of the resected specimen) and a benign lesion in 15 cases (resected specimen : 6 benign lesions and 9 carcinomas); among the 9 biopsies performed after ES, two only revealed a carcinoma (resected specimen : 4 benign lesions and 5 carcinomas), with an accuracy rate of 64% globally and 66% after ES. At EUS, 13 lesions were presumed limited to ampulla (benign lesion or stages T in situ and T1) but 4 out of these 13 were classified T2 or more histologically; among the 7 tumours classified T2 or more by EUS, one was histologically limited to ampulla. The accuracy rate of EUS for the T stage was 75%. At EUS, lymph nodes were presumed benign (N0) in 21 cases (histology : N0 in 15 cases and N1 in 6 cases) and metastatic (N1) in 5 cases (histology : N0 in 2 cases and N1 in 3 cases), with an accuracy rate of 69%. All endoscopic explorations were compatible with a benign lesion in 11 patients ; histologically, 6 patients out of these 11 had a carcinoma including one with a T1N1 and two with a T2N0 tumour.

Conclusions : In patients with ampulloma tumour, SVD with biopsies even after ES, and EUS are not accurate enough to make sure preoperatively that the tumour is benign. Therefore a local resection cannot be safely indicated.

F136

PANCREATICODUODENECTOMY FOR PEERIAMPULLARY TUMOR - TAIWAN EXPERIENCE

MF Chen, YY Jan
Department of Surgery, Chang Gung Memorial Hospital

In a 11-year period between 1981 and 1991, 297 patients with periampullary tumor were refered to our surgical department and 131 pancreaticoduodenectomy were performed, including 79 for ampullary carcinoma, 22 for pancreatic head tumor, 18 for distal common bile duct carcinoma and 12 for duodenal tumor. Those patients who received pancreaticoduodenectomy consisted of 78 female and 53 male and their mean age was 55.8 years with a range from 21 to 82 years.

The overall mortality of the 131 pancreaticoduodenectomy was 7.63% and it was 4.76% in the last 5 years. The complication rate was as high as 38.9%. The anastomatic leakage (15.3%) was the leading cause of hospital mortality. Several factors were analyzed and revealed that old age, abdominal pain, duration of symptoms, serum bilirubin, albumin and serum Alk-P levels and operative blood loss all did not influence the survival rate. However, female patients, no lymph node metastasis, and well-differentiated tumor had better survival. Excluding the papillary cystoadenocarcinoma of pancreatic head, leiomyosarcoma of duodenum and benign disease, the actual 5-year survival of ampullary, pancreatic head, distal common bile duct and duodenal carcinoma were 47.4%, 20%, 30.8% and 33% respectively.

Female patients, patients with out lymph node metastasis and with well-differentiated carcinoma had better prognosis.

F137

**OVEREXPRESSION OF TISSUE FACTOR INCREASES PANCREATIC CANCER CELL INVASION**

V. Chinswangwatanakul, A.K. Kakkar, N.R. Lemoine*, S. Tebbutt, R.C.N. Williamson
Department of Surgery, Royal Postgraduate Medical School, London, U.K.
* Imperial Cancer Research Fund, London, U.K.

Tissue factor (TF), the transmembrane cellular receptor which is the physiological initiator of blood coagulation is not expressed in normal pancreatic tissue, but in the adenocarcinoma of the pancreas its expression increases with progressive dedifferentiation. We have studied the effect of TF expression on *in vitro* invasion by the human pancreatic carcinoma cell line MIA PaCa-2. The full length TF gene (1360 base pairs) was cloned into the plasmid DNA vector pcDNA3 in sense and antisense orientations. Diagnostic DNA digests confirmed the correct orientation of the cloned genes. These vectors were used to transfect the MIA PaCa-2 cell line *in vitro* using the lipofectin method. Expression of TF sense gene resulted in a five fold increase in cell surface procoagulant activity from 7.8 thromboplastin units per $10^4$ cells for wild type to 40 units (p=0.001). There was a small reduction in procoagulant activity for the TF/antisense clones to 7.6 units per $10^4$ cells. The total antigenic content of TF gene product (sense or antisense) markedly increased from 160 pg/mg for wild type to 7733 pg/mg for sense and 856 pg/mg for antisense. *In vitro* invasion was assessed in a standard matrigel assay by counting the number of cells per high powered field, after haematoxylin staining. The mean ± s.d. number of cells successfully invading through matrigel was 60.75±13.04 for the wild type and 210.2±57.62 for the TF/sense clone (p=0.02, 95% CI:-276.45 to -55.02) and 42.2±6.43 for the TF/antisense clone (p=0.09; 95% CI: -8.11 to 51.98). This study is the first to demonstrate that cellular expression of TF may influence the invasion of pancreatic tumour cells.

### F138

**PERCUTANEOUS CYSTOGASTRO- AND CYSTODUODENOSTOMY IN TREATMENT OF PANCREATIC PSEUDOCYSTS**

S.A. Dadvani, A.N. Lotov, V.J. Zavodnov, L.V. Chistov, G.Ch. Musaev, V.N. Avakian.
Surgical Clinic No 1, Moscow Medical Academy, MOSCOW, RUSSIA

Pseudocysts and abscesses of pancreas in abdominal surgery, particularly at the peak of the acute inflammation, are one of the most often and serious complications of pancreatitis.

From 1986 to 1995 years 128 patients with pancreatic pseudocysts were hospitalised in our clinic. Traditional surgical treatment was performed in 17 patients with calculous pancreatitis complicated by formation of pancreatic pseudocysts and in 3 patients with cystadenoma. The other patients were treated with ultrasound-guided percutaneous punction and drainage of pancreatic pseudocysts, including 13 cases of internal percutaneous cystogastro- and cystoduodenostomy. Simultaneously a complex set of conservative therapy including high doses of sandostatin (600-800 mg/day) was carried out for preventative purposes (3 days) or treatment of pancreatitis. Percutaneous fulfilling of cystogastroanastomosis was performed in 10 patients with pseudocysts of pancreas body and tail by means of the Hancke percutaneous pancreatic cyst drainage set /"COOK", Denmark/. Obtained material was sent for cytological, biochemical and bacteriologic investigations.

For the first time in our clinic we performed ultrasound- and endoscope-guided percutaneous formation of internal cystoduodenoanastomosis using catheter of our own modification in patients with pseudocysts of pancreatic head. Both patients had pancreonecrosis and acute destructive pancreatitis complicated by obstructive jaundice. On the 3-rd - 4-th day after cystoduodenostomy the level of direct bilirubin decreased to normal and on the 7-th - 8-th day the patients were discharged.

In one patient during the earliest stage of our work the attempt to form cystogastroanastomosis was failed, which demanded surgical treatment. Long-term results (14-46 months) didn't show disease recurrence. Therefore, we think that percutaneous cystogastro- and cystoduodenostomy is less traumatic and highly effective and should be the method of choice in treatment of such patients.

### F139

**ENDOSCOPIC DRAINAGE OF PANCREATIC PSEUDOCYSTS.**

E Della Libera Jr, ES Siqueira, CQ Brant, M Morais, AP Ferrari Jr, DL Carr-Locke.
Division of Gastroenterology, Universidade de São Paulo, São Paulo - Brazil

Pancreatic pseudocysts have been successfully treated by endoscopic drainage (cystogastrostomy or duodenostomy, and transpapillary drainage). We report our experience with endoscopic therapy of pancreatic pseudocysts. From July/94 to August/95, 16 patients with pancreatic pseudocysts were referred to ERCP because of persistent pain and/or jaundice. In 4/16 (25%) endoscopic therapy was not performed because we were not able to place a guide wire beyond a pancreatic stenosis and there was no indentation of gastric or duodenal wall. In the remaining 12 patients (9/3 male/female ratio), mean age 38.2 years (range 24 - 64 years), 15 pseudocysts were treated with cystogastrostomy (5), cystoduodenostomy (2) and transpapillary drainage (8). Etiology was: alcoholic chronic pancreatitis (8), blunt abdominal trauma (2), surgical trauma (2). Pseudocysts mean size was 7.83 cm (range 3,5 - 18 cm) and were located in the head (4), body (8) and tail (3). Complications were present in 7/16 patients: 2 early stent occlusion, 1 fever, 1 pneumoperitoneum, 1 bleeding, 1 proximal migration and 1 perforation. Except for the perforation that required surgery, all complications were minor and medically and/or endoscopically managed. There were no deaths. Mean follow up was 150 days (range 15 - 360 days) and mean stent period was 134 days (range 30 - 210 days). Clinical improvement was noted in 11/12 (91%): all but one pseudocyst resolved, 8 patients are asymptomatic and 3 are taking small doses of analgesics. In the only patient that persisted with pain, stent was removed and she was sent to surgery because of chronic pancreatitis.

### F140

**The expression of growth associated protein 43 and clinical symptoms correlated in patients with chronic pancreatitis.**

P. Di Sebastiano, T. Fink, E. Weihe, H. Friess, H.G. Beger, P. Innocenti and M.W. Buechler.
Surgical Unit, D'Annunzio University, Pierangeli Clinic Pescara (Italy); Institute of Visceral and Transpantation Surgery, University of Berne (CH); Department of General Surgery, University of Ulm (FRG)

**Background/aims.** Changes in innervation pattern and neuropeptide content have been demonstrated in chronic pancreatitis. Growth associated protein-43 (GAP-43) is expressed at high levels in the developing nervous system. Recent studies demonstrated re-expression of GAP-43 after experimental lesions of peripheral nerves. We used GAP-43 as a marker of neuronal plasticity in chronic pancreatitis and correlated histological findings with clinical data. **Methods.** The innervation of the normal pancreas from 14 organ donors was determined immunohistochemically with the panneuronal marker protein-gene product 9.5 (PGP 9.5) and the expression of GAP-43 was also characterized. The findings were compared with results obtained from 29 patients with chronic pancreatitis. The density of PGP 9.5 and GAP-43 was quantified by image analysis. In addition, patients responded to a symptom questionnaire for the evaluation of clinical findings. **Results.** In chronic pancreatitis a marked increase of PGP 9.5 and GAP-43 immunostaining was observed: digitized morphometry revealed that 70% of PGP-9.5-immunoreactive nerves were also GAP-43-immunoreactive whereas in the controls the relative area of GAP-43 was less than 3% ($p<0.01$). GAP-43-immunoreactivity significantly correlated with the intensity of pain ($p<0.01$) and duration of disease ($p<0.05$). **Conclusions.** GAP-43 re-expression at high levels both in nerves and intrinsic neurons indicates an axonal sprouting mechanism in chronic pancreatitis; the correlation between clinical symptoms and GAP-43-immunostaining suggest a link between plasticity of peripheral nervous system and pain generation in chronic pancreatitis.

### F141

**ROLE OF ADJUVANT RADIOTHERAPY IN TREATMENT OF RESECTABLE PANCREATIC HEAD CARCINOMA.**

**G. Doglietto, D. Frontera, G. Viola, G. Alfonsi, M. Buononato, A.G. Morganti *, N. Cellini *, F. Crucitti.**
**DEPARTMENT OF SURGERY; * DEPARTMENT OF RADIOTHERAPY - CATHOLIC UNIVERSITY SCHOOL OF MEDICINE, ROME, ITALY.**

From March 1990 we adopted a combined multimodal treatment of resectable pancreatic head carcinomas including subtotal (or total, when needed) pancreatectomy, intraoperative irradiation (IORT) and external beam radiation therapy (EBRT). Twenty-two patients were treated following the above mentioned protocol: 20 subtotal and 2 total pancreatectomies were performed. IORT was delivered to the tumor bed (including celiac axis, portal vein and origin of superior mesenteric vessels) with a dose of 10 Gy. Mean time spent to carry out IORT (patient's transfer to the bunker, irradiation and return to the operating room) was 54 minutes. Postoperative major complications were observed in 5 patients (23 %) and mortality was 9 % (2 cases). Postoperative EBRT of the tumoral and lymphatic bed (3 beams or box technique - 50 Gy) was performed in 16 patients. In 6 cases (27 %) postoperative EBRT was not started because of postoperative death (2 cases) or unsuitable clinical status. In the more recent period of our experience, 12 patients underwent preoperative "flash" radiotherapy of the liver and the pancreas (opposite beam technique - 5 Gy) to treat possible liver micrometastases and to reduce tumor spread during surgery.

Median survival was 11.8 months. Statistical analysis showed a significantly better survival in women (19.9 months vs. 9 months: $p<0.04$) and in patients undergoing preoperative "flash" EBRT (25.6 months vs. 9.5 months: $p<0.03$).

Our experience suggests that combination of EBRT and IORT with surgical resection of pancreatic cancer is safe, tolerable and offers a good local control. High frequency of liver metastases suggests the need to test new therapeutic combinations: in this respect, neoadjuvant radio-chemotherapy seems to be the most promising.

### F142

**ACUTE NECROTIZING PANCREATITIS (ANP): ANALYSIS OF 108 CONSECUTIVE CASES**

G.B. Doglietto, F. Pacelli, G. De Vivo, s. Alfieri, C. Carriero, M. Malerba, F. Crucitti
Department of Surgery, Catholic University of Rome, Rome, Italy

**AIM**. To review our experience in the management of ANP.

**MATERIALS AND METHODS**. Hospital charts of 108 patients with ANP consecutively admitted at our Unit between 1981 and 1995 were retrospectively reviewed. Demographic data, etiologic factors, diagnostic procedures, treatment modalities, mortality and evolution of the disease were noted. Apache II score was calculated in all patients. ANP was diagnosed on the bases of standard clinical criteria, ultrasonographic and computed tomographic scan. The presence of a secondary infection was detected by clinical findings (persistently febrile course associated with leukocytosis despite heavy antibiotic treatment) and confirmed in all cases by both intraoperative and bacteriological findings. Conservative treatment was based on fluids and electrolytes replacement, cardiac and emodynamic monitoring, wide spectrum antibiotics (in case of proven sepsis) and total parenteral nutrition; when necessary patients were tranferred in ICU for supportive treatment of renal and respiratory failure. The indications for surgical treatment were: the development of a secondary infection, acute abdomen, MOF syndrome and cardiocircolatory shock. The surgical procedure consisted of abdominal entrance by a bilateral subcostal incision, exposure of the pancreas by dividing the gastrocolic omentum, debridment and necrosectomy; in the presence of pancreatic abscess, the purulent collection was entirely drained externally. At the end of operation the abdomen was closed after the placement of large-bore sump drains and passive Penrose drains. In case of infection, since 1987, an open treatment with marsupialization of the lesser peritoneal sac was routinely adopted.

**RESULTS**. 55 patients were female and 53 male. The mean age was 55.5 (range 17-93). Gallstones were the most frequent etiologic factor (55 patients). Apache II mean score was 13.75. In 63 patients the necrosis was sterile: 13 underwent emergency operation for shock (n.6), acute abdomen (n.2) or MOF (n.5), in 50 a conservative treatment was adopted with completely resolution in 23 and development of pseudocyst in 27. In 45 patients (46.6% transferred from other hospitals) was documented a secondary infection: infected necrosis in 31 and pancreatic abscess in 14; all these patients were surgically managed. Overall mortality rate was 17.5 %: 8% (n.5) for sterile necrosis and 31.1% (n.14) for secondary infections (38.7% for infected necrosis and 14% for pancreatic abscess).

**DISCUSSION**. Controversy still surrounds the optimal management of ANP particularly regarding the indications, the timing and the methods of surgical intervention. For sterile necrosis in the absence of severe systemic complications a conservative treatment seems to be justified both for the natural history of the illness (high incidence of resolution) and the poor results of early surgery. In presence of a secondary infection surgical treatment is mandatory.

### F143

**LONG-TERM RESULTS IN DERIVATIVE vs RESECTIVE PROCEDURES FOR CHRONIC PANCREATITIS.**

M. Falconi, C. Bassi, N. Sartori, G. Talamini[1], L. De Santis, A. Bonora, R. Salvia, E. Caldiron, G. Cavallini[1] and P. Pederzoli.
Surgical and [1] Medical Departments. University of Verona, Italy.

Indications and evaluations of surgical results in ductal derivations compared to pancreatic resections are still matter of debate, owing to not yet clear natural history of chronic pancreatitis and changes in the course of the disease after surgery.

From 1971 to March 1995, out of 753 patients suffering from chronic pancreatitis 484 (56.7%) of them (421 males, 63 females; mean age 41.6 years, range 17-76 ys) were submitted to pancreatic surgery; severe not responding pain (73%) and pancreatic pseudocysts (10.6%) were the most frequent indications to surgery.

In 398 patients (82.2%) we performed derivative procedures (267 pancreaticojejunostomy, 68 cysto-pancreaticojejunostomy, 50 cysto-jejunostomy, 9 Du Val procedure, 4 double sphincteroplasties); 86 pts (17.8%) underwent pancreatic resections (28 Whipple procedure, 33 Puestow procedure, 20 distal pancreatectomy without anastomosis, 5 total pancreatectomy).

In two series were found no significant differences as regards sex, age, etiology, calcifications, time of appearance and severity of symptoms; main pancreatic duct was significantly more enlarged in derivated than resected patients ($p$<0.0001).

Length of operation, units of transfused blood, postoperative mortality (2 versus 5) and morbidity, hospital staying time were significantly lesser in derivated group ($p$<0.0001).

A similar number of patients in two series (77.2% vs. 71.9%) were pain-free after a 6.1 years median follow-up.

Given comparable results in reducing pain, we suggest that derivative surgery has however to be preferred owing to the less biological load; pancreatic resection instead has to be performed when pancreatic duct is not enlarged and in case of complications related to pseudocysts and suspect of pancreatic cancer.

### F144

**LIVER CHEMOEMBOLIZATION IN SYNCHRONOUS METASTASES FROM ENDOCRINE TUMORS OF THE PANCREAS.**

M. Falconi, C. Bassi, A. Bonora, G.C. Mansueto[1], G. Butturini, E. Caldiron, R. Salvia, N. Sartori, G. Talamini[2] and P. Pederzoli. Surgical and[1] Radiological Departments, [2] Gastroenterology Service. University of Verona, Italy.

The biological behaviour and slow evolution of endocrine pancreatic tumors, even in presence of liver metastases, have prompted to a most radical surgical approach and to research of any effective adjuvant treatment. Since 1991, hepatic arterial chemoembolization (TACE) has been proposed in the treatment of malignant endocrine pancreatic tumors, on the experience of primary hepatic malignancy.

From 1985 to June 1995, we observed 63 patients (25 males, 38 females; mean age 53.8 years, range 17-78 ys) suffering from histologically proven endocrine tumour of the pancreas, in 28 cases (44.5%) of functioning type, non functioning in 35 (55.5%). In 23 patients (36.5%) the tumours were malignant with presence at diagnosis (17 pts) or onset during follow-up (6 pts) of liver metastases.

Of 17 patients (74%) presenting synchronous metastases, one patient underwent radical resection with the removal of the only metastasis detected; 8 patients were not submitted to radical surgery because of either locally advanced disease or not available TACE.

Eight patients (3 males, 5 females; mean age 43.8 years, range 49-67) underwent one or more treatment of TACE prior and/ or after the palliative resection of pancreatic malignancy. After cannulation of proper hepatic artery, according to Seldinger technique, whole liver parenchyma was microembolized with a solution of Lipioidol FU (10 ml) and dacarbazine (Deticene) 500 mg; afferent artery was then embolized with fragments of Spongostan or contour microspheres. The response to the treatment was assessed by CT-scan within 30 days. TACE was repeated, if possible, within 90 days and in case of relapsing disease and the patients were followed up by CT-scans every 3 months.

Of the 8 patients undergoing TACE, 2 died respectively after 11 and 18 months from surgery, while 6 patients are still alive after a mean follow-up of 26.3 months (range 12-55), 2 with stable and 4 with progressive disease. The response to the first course of TACE was positive in 5 of these cases, with a 14 months mean time of stabilization. In one recently treated patient the response cannot yet be assessed. The median actuarial survival of these 8 patients is more than 55 months.

These results suggest that TACE, combined with surgical resection of pancreatic tumor, seems to be able of stabilizing the disease for a certain time and increasing patient survival. Further studies are necessary to settle the choice of better chemoterapeutic, dosage and time intervals between treatments.

### F145

**FACTORS CONTRIBUTING TO SUCCESSFUL TREATMENT OF INFECTED PANCREATIC NECROSIS**

G. Farkas, J. Márton, Y. Mándi*, E. Szederkényi

Department of Surgery and Institute of Microbiology*, A. Szent-Györgyi Medical University, Szeged, Hungary

Infected pancreatic necrosis and sepsis are the leading causes of mortality in necrotizing pancreatitis. Since 1986, 136 patients with infected pancreatic necrosis have been treated. The mean number of APACHE II score was 17.5 (range 11-31). In all cases, the infected necrosis was combined with retroperitoneal abscesses. 93 were situated in the right or left retrocolic area, 13 in the subphrenic region, and 30 in the retroduodenal or subhepatic area. The surgical treatment was performed on average 18.5 days (range 8-25 days) after the onset of acute pancreatitis. The operative management consisted of wide-ranging necrosectomy in the total affected area, combined with widespread lavage and suction drainage. In 62 of the 136 cases (45.5%), some other surgical intervention (distal pancreatic resection, splenectomy, cholecystectomy, sphincteroplasty or colon resection) was also performed. Continuous lavage and suction drainage was applied for an average of 39.5 days (range 21-90 days), with an average of 8.5 (range 5-15) litres of saline per day. The bacteriologic findings revealed mainly enteral bacteria, but Candida infection was detected frequently. The incidence of fungal infection was 21%. Twenty-three patients (17%) had to undergo reoperation. The cytokine production capacity (TNF, IL-1 and IL-6) was shown to correlate with the prognosis. The overall hospital mortality was 6.6% (9 patients died). In our experience, infected pancreatic necrosis responds well to aggressive surgical treatment, continuous, long-standing lavage and suction drainage, together with supportive therapy consisting of immunonutrition and blockade of cytokine production, combined with adequate antibiotic and antifungal medication.

### F146

### TGF-β1 AND IL-6: NEW ASPECTS IN PANCREAS REGENERATION ?

Gy. Farkas Jr., T. Takács, *Y. Mándi and J. Lonovics
First Department of Medicine and *Department of Microbiology,
Albert Szent-Györgyi Medical University, Szeged , Hungary

Some reports suggest that a low dose of cholecystokinin octapeptide (CCK-8) may induce regeneration of the pancreas. This regeneration can be detected in ever increasing degree up to the end of the first month; it then becomes stable. We wanted to investigate whether changes occur in the serum TGF-β1 and IL-6 levels during regeneration, and whether there is a connection between their levels and the rate of regeneration in rats.
Distal pancreas resection (75%) was performed. CCK-8 was administered subcutaneously in a 300 ng/kg dose 3 times per day to the investigated group, while the control animals received the same amount of saline. The rats were examined 3, 7, 14 and 28 days after the first injection. Serum TGF-β1 levels were determined by ELISA, IL-6 levels by bioassay, DNA content by Giles & Meyers method.
The weights of the residual pancreas were increased in both groups on day 3. At subsequent times the weights decreased in the controls but increased continuously in the CCK-8-treated group. There was significant difference on day 14, and on day 28 the pancreas weight almost doubled in the CCK-8 group, whereas it decreased to the normal level in the controls. The DNA content of the pancreas was continuously higher in the treated than in the control group. It reached the maximum level on day 28, with a significant different (1800 ± 350 vs. 780 ± 240 γ/pancreas). The protein content reached its highest level on day 28 in the CCK-8-treated group (32.435 ± 7.88 mg/pancreas). A significantly higher level of IL-6 was measured on day 7 vs. the control (250 ± 70 vs. 50 ± 30 pg/ml). It later decreased, but remained above the control level. Significantly different TGF-β levels were measured on days 7 and 14 (290 ± 40 vs. 155 ± 70, and 275 ± 10 vs. 180 ± 65 ng/ml, respectively). There was no difference between the TGF-β1 and IL-6 levels in the two groups by day 28. No significant changes in the amylase levels were observed; they remained at a normal level (4.8 ± 0.8 U/ml). This indicates that the increase in the pancreas weight was not caused by pancreatitis.
Our results reveal that regular low dose of CCK'8 injections resulted in pancreas regeneration following 75% distal resection. This was indicated by increases in the pancreas weight, and in the DNA and protein contents of the pancreas. Significantly elevated serum TGF-β1 and IL-6 levels were also detected up to day 14. These data suggests that TGF-β1 and IL-6 might play a stimulatory effect in the early stage of regeneration of the pancreas.

### F147

### TOTAL PANCREATECTOMY IN END-STAGE CHRONIC PANCREATITIS

W.R. Fleming, R.C.N. Williamson
Department of Surgery, Royal Postgraduate Medical School, Hammersmith Hospital, London, United Kingdom

Forty patients underwent total pancreato-duodenectomy for end-stage chronic pancreatitis. There were 34 men and 6 women, with a median age of 39 years (range 21-66 years). Alcoholism was the major aetiological agent (30 patients), and five patients had had previous acute idiopathic pancreatitis. The overwhelming indication for operation was severe abdominal pain, complicated by failing exocrine and endocrine function. Resection was performed in one (n=17) or two stages (n=23), following previous proximal (7) or distal (16) pancreatectomy; progression from partial to total pancreatectomy occurred over an interval of 8-96 months (median 15 months). Another 6 patients had undergone previous pseudocyst or duct drainage procedures. The pylorus was preserved in 28 patients and the spleen in 10. Median operation time was 6 hours (range 2.5-8.5 hours) and median blood loss 2000 ml (range 500-16000 ml). There were two hospital deaths and three patients required reoperation. Of 38 survivors, 30 obtained complete or substantial relief of pain. There were 15 late deaths at 2.5-120 months postoperatively, 13 in the alcohol group and 11 disease-related. Total pancreatectomy can relieve the intractable pain of chronic pancreatitis at the cost of possible premature death from continuing alcohol abuse.

### F148

### OPEN PANCREATIC DRAINAGE IN SEVERE NECROTIZING PANCREATITIS

G. Funariu, M. Suteu, I. Mihut, D. Grecea, O. Chira
1st Surgical Clinic, University of Medicine and Pharmacy, Cluj-Napoca, Romania

The aim of this study was to analyse comparatively results of controlled open pancreatic drainage in noninfected and infected pancreatic necrosis. Twenty four patients with necrotizing pancreatitis were treated surgically by open lesser-omental-sac drainage (celiostomy) in the last five years. They were classified into two groups according to date and indications of surgical drainage: a group of 12 patients with early celiostomy (in the first week after the onset of pancreatitis) for noninfected necrosis, and the second group of 12 patients with late celiostomy (2 to 12 weeks after pancreatitis) for infected necrosis and pancreatic abscess. Age, sex and etiology of pancreatitis were similar between surgical groups. The diagnosis has been based on clinical syndrome, laboratory tests including bacteriologic cultures, ultrasound and CT scan findings. Drainage procedure consisted in marsupialization of lesser-omental sac by suturing open gastrocolic ligament to anterior peritoneum. Abdominal wall was left largely open in this site and the drains were inserted via celiostomy. Only 6 patients with early operation underwent necrosectomy at date of laparotomy and repeated necrosectomy was possible in 4 patients. The necrosectomy was carried out during laparotomy in all patients of the second group and three to nine reexploration via celiostomy were needed for removal large residual necrosis and purulent material. In group with noninfected necrosis, eight patients died postoperatively (66.6%) because of multiple-organ-system failure. In the second group with pancreatic abscess, only one patient died early after surgery (8.3%) of septic shock. Two patients developed postoperatively distant submesocolic abscesses resolved by relaparotomy. Other postoperative complications were pancreaticocutaneous fistulas resolved spontaneously (2 patients) and external bleeding (1 patient) requiring surgical hemostasis. In conclusion, controlled open pancreatic drainage is more effective in patients with infected pancreatic necrosis and stable biologic condition. It facilitates intermittent debridements of residual necrosis and purulent foci. Early open drainage is not proved to be of benefit to unstable patients with severe pancreatitis and noninfected necrosis.

### F149

### ANALYSIS OF PURE PANCREATIC JUICE AND BILE FOR DIAGNOSIS OF MALIGNANCY — K-RAS POINT MUTATION, CEA AND CYTOLOGY

N.Futakawa, W.Kimura, N.Sata, S.Yamagata, Y.Takei, I.Han, K.Morikane, T.Inoue, H.Shinkai, T.Muto
First Department of Surgery, University of Tokyo, Tokyo, Japan

**[Aim]** To investigate the validity of K-ras codon 12 point mutation, values of CEA, CA19-9, and cytology of pure pancreatic juice and bile for a diagnosis of pancreaticobiliary carcinoma.
**[Materials and Methods]** Sixty four cases suspected of pancreaticobiliary diseases, were examined. Pure pancreatic juice could be obtained and measured in 33 cases--malignant :14 cases (42%), neoplasitc :19 cases (58%), and benign : 14 cases (42%). Bile could be measured in 33 cases--malignant : 20 cases (61%) and other 13 cases were benign non-neoplastic diseases. Pure pancreatic juice was taken from ERP catheter for 5 min without stimulation of secretin. Bile was also taken without stiulation. K-ras codon 12 point mutation, values of both CEA and CA19-9, and cytology were examined. K-ras mutation was detected by two-step PCR-RFLP method. Serous CEA , CA19-9 were also examined.
**[Results]** 1. CEA values of pancreatic juice in cases with carcinoma were significantly greater than those in benign group (P<0.01). When cut-off line of CEA values was set up to 50 ng/ml, predictive values were described bellow. 2. K-ras mutation of pure pancreatic juice was more frequent in carcinoma group (P<0.02). 3. When discrimination analysis was applied (Formula 1), accuracy was 100%. 4. CA19-9 in sera was most useful for diagnosis of bile duct carcinoma.

| | Pure Pancreatic Juice ras mutation | CEA≧50 (ng/ml) | cytology | Serum CA19-9≧100 (U/ml) | Discriminant Analysis |
|---|---|---|---|---|---|
| PPV | 67 (100) | 64 | 75 | 75 | 100 |
| NPV | 83 (78) | 75 | 69 | 70 | 100 |
| Accuracy | 79 (83) | 73 | 70 | 71 | 100 |

PPV : Positive Predictive Value NPV : Negative Predictive Value
( ) : discrimination between neoplastic and non-neoplastic diseases

<Formula 1>
0.145*(Age) + 0.0157*(P_CEA) + 2.51*(P_ras ) + 0.00801*(S_CA19-9) - 0.00000398*(P_CA19-9) - 10.83> 0

P : Pure Pancreatic Juice S : Serous ras : 1 (mutant), 0 (wild type)

**[Conclusion]** K-ras mutation and value of CEA of pure pancreatic juice were useful for discrimination between benign and malignant diseases of the pancreas. With multivariate analysis (formula 1), much better discrimination was possible.

F150

SANDOSTATIN IN THE MANAGEMENT OF ACUTE HEMORRHAGIC PANCREATITIS

E.Galperin, A.Chevokin, L.Platonova, N. Shono, G.Achaladze, K.Docuchaev.

Department of Liver surgery, Sechenov Moscow Medical Academy, Russia

Two groups (each of 14 identical patients with acute hemorrhagic pancreatitis) were investigated. The diagnosis of acute pancreatitis was made on the basis of clinical findings, data of laparoscopy, US, and laboratory studies. All patients had severe pancreatitis with three and more positive Ranson's prognostic signs. 10 patients had multiple organ failure. Both groups received the routine administration of antibiotics and dynamic laparoscopy with drainage of the abdominal cavity. The first group was treated for 6 days with 3x200 μg of Sandostatin s.c. The second group wasn't treated with Sandostatin and was arranged as the control group. The results was follows: in 12 patients among 14, treated with Sandostatin, brought to the break of the illness in the first phase. In 2 cases developed septic complications. These patients were operated on. In Sandostatin treated patients the normalisation of the inflammatory and toxic manifestation appears earlier then in the control group (5-7 days versus 11-12 days). Using of Sandostatin in the beginning of pancreatitis prevents the development of septic complications. These complications appeared in 2 patients out of 14, that received Sandostatin from 4-5th day of pancreatitis. In the control group 5 of 14 patients were operated on because of purulent complications. Application of Sandostatin led to the normalisation of serum proteases on 3-5 day. At the same time was noted an increase of antiferments. Sandostatin plays a useful role in the treatment of acute hemorrhagic pancreatitis.

F151

RAS PEPTIDE VACCINATION IN PATIENTS WITH ADVANCED PANCREATIC CANCER: RESULTS OF A PHASE I/II STUDY.

M. K. Gjertsen[1], A. Bakka[2], J. Breivik[1], I. Sæterdal[1], T. Gedde-Dahl III[1], K. T. Stokke[3] B. G. Solheim[4], T. S. Egge[5], O. Søreide[2], E. Thorsby[1] and G. Gaudernack[1].
[1]Institute of Transplantation Immunology, [2]Department of Surgery, [4]Red Cross and National Blood Centre and [5]Department of Radiology, The National Hospital and University of Oslo, Norway. [3]Pronova a.s., Gaustadalleen 21, Oslo, Norway.

In a pilot phase I/II study we have tested synthetic ras peptides used as a cancer vaccine in five patients with advanced pancreatic carcinoma. The treatment principle used was based on loading professional antigen-presenting cells (APCs) from peripheral blood with a synthetic ras peptide corresponding to the K-*RAS* mutation found in tumour tissue from the patient. Peptide loading was performed *ex vivo* and the next day APCs were re-injected into the patients after washing to remove unbound peptide. The patients were vaccinated in the first and second week and thereafter every 4-6 weeks. In 2 of the 5 patients treated, an immune response against the immunising ras peptide could be induced. None of the patients showed evidence of a T-cell response against any of the ras peptides before vaccination. The treatment was well tolerated, and could be repeated multiple times in the same patient. Side effects were not observed even if an immunological response against the ras peptide was evident. We conclude that ras peptide vaccination according to the present protocol is safe and can result in an immune response even in patients with advanced malignant disease. In a clinical setting where tumour is surgically removed, such T- cell responses may be of potential benefit.

F152

ACUTE PANCREATITIS IN CARDIAC AND RENAL ALLOGRAFT RECIPIENTS - A GRAVE COMPLICATION

E. J. Hagopian, J. Finegold, J. A. Chabot, P. D. Stevens, J. A. Fayter, Y. H. Hahn, A. I. Benvenisty, and M. A. Hardy.
Departments of Surgery, Medicine, and Radiology, Columbia University College of Physicians and Surgeons, New York, NY, USA.

The purpose of this report is to define the outcome of acute pancreatitis and to characterize and compare the disease and treatment patterns in cardiac and renal allograft recipients. Medical records of cardiac and renal allograft recipients from 1980 to 1994 whose ICD-9 codes included acute pancreatitis were reviewed. Pancreatitis was defined as abdominal pain associated with either hyperamylasemia (≥200) and/or classic radiographic findings. Severity of pancreatitis was classified as mild (hospitalization ranging 1-7 days), moderate (>7 days), or severe (ICU admission). During the study period, 1432 transplantations were performed including 726 cardiac and 706 renal allografts. Twenty-four patients developed acute pancreatitis following transplantation yielding an incidence of 1.7%. Of 706 renal allograft recipients, 11 (1.6%) developed acute pancreatitis whereas 13 of 726 cardiac recipients (1.8%) developed the disease. Severity of disease was more likely to be classified as moderate in both groups (69% cardiac, 67% renal); however, mild disease was more frequent in renal (18%) than in cardiac (7.7%) whereas severe disease was more frequent in cardiac (23%) than in renal (18%) allograft recipients. An unknown (including infectious) etiology was identified most commonly in both groups (69% cardiac, 73% renal). In 18% of renal and 23% of cardiac recipients, cholelithasis was determined to be the initiating factor for pancreatitis whereas ERCP was found to be causative in 7.7% and 8.3% of cardiac and renal patients respectively. Computed tomography images were available for review in 70%. A large proportion of allograft recipients were classified radiologically in the most severe scores by the Balthazar criteria. Three of 13 (23%) cardiac allograft recipients and 0 of 11 (0%) renal allograft recipients required operative pancreatic debribement. All patients requiring operative intervention had pancreatic candidiasis. Four of 24 patients (17%) died. The mortality in the cardiac group was 23% (3 of 13) as compared to 9% (1 of 11) in the renal group. Operative intervention resulted in 100% mortality (3 of 3). In conclusion, pancreatitis is relatively common among cardiac and renal allograft recipients and most frequently is of uncertain etiology. Cardiac allograft recipients present with more severe disease which more frequently requires operative intervention and results in a higher mortality.

F153

DUODENAL COMPLICATIONS AFTER WHOLE ORGAN PANCREAS (PA) TRANSPLANTATION (TX)

NS. Hakim, E. Benedetti, DER Sutherland, R. Gruessner
Department of Surgery University of Minnesota

There is a broad spectrum of duodenal complications after bladder-drained PA TX using the duodenal segment technique; little is known of the effect of duodenal complications on the long-term prognosis of patients and PA grafts. **MATERIALS AND METHODS:** We studied incidence and outcome of duodenal complications after 373 bladder-drained (stapled duodenoscystostomy in 95%) whole organ pancreatico-duodenal transplants (7/85 thru 1/95). Complications were defined as early if they occurred within the first postoperative month, and late otherwise. Mean follow-up was 5.5 months (range, 3 to 108 months). **RESULTS:** 1. There were 42 ***duodenal leaks*** (11.3%); 12 early with a mean of 11.5 days (range, 1-28 days) and 30 late with a mean of 6.9 months (range, 1-36 months). The site of the leak was at the duodeno-cystostomy site (true bladder anastomotic leaks) in 15 cases, at the stapled proximal duodenal stump in 8, and at the stapled distal duodenal stump in 4. In the other 15 cases it was impossible to identify the exact site of the leakage because the patients were treated conservatively and the studies (Cystogram/CT Scan) were unable to identify the site. In 23 (55%) patients the leakage was oversewn; but 6 (14%). 4 patients had a recurrent leak requiring enteric conversion 2 to 12 months after the first leak. 12 (28%) patients with small leaks were treated conservatively with an indwelling catheter for 1 to 2 months with resolution of the leak. 2. Gross ***hematuria*** (defined as severe enough to require cystoscopy) occurred in 26 patients (7%), 10 early with a mean of 14 days (range, 7-21 days) and 16 later with a mean of 11.5 months (range, 1.5-60 months); 2 (8%) patients had an enteric conversion and 2 (8%) had a graft pancreatectomy. 3. Two patients (0.5%) had recurrent ***bladder stones*** requiring repeated cystoscopies and removal of stones encrusted at the site of visible staples. There were no graft losses. 4. Nine patients (2.4%) with ***recurrent UTI*** required cystoscopy and removal of stitching and staple material as the focus of infection. There were no graft losses or conversions. 5. ***CMV duodenal ulceration*** was identified in 6 (1.6%) patients; 2 patients had enteric conversion. **CONCLUSIONS:** Duodenal complications are common, but are not associated with a high rate of PA graft loss (8%); mortality from duodenal complications in our series was 0%. Early surgical intervention, including enteric conversion is safe and can decrease morbidity and mortality in this patient population.

### F154

### PARTIAL DUODENOPANCREATECTOMY WITH RADICAL LYMPHADENECTOMY IN PATIENTS WITH PANCREATIC AND PERIAMPULLARY CARCINOMA

D.Henne-Bruns, I.Vogel, B. Kremer
Department of Surgery, University of Kiel, Kiel, Germany

Between 1988 and 1995 76 patients with pancreatic and periampullary carcinoma recieved a partial duodenopancreatectomy.
49 patients underwent partial duodenopancreatectomy with extended radical retroperitoneal lymphadenectomy (Group A: cancer of the head of the pancreas, n=27, periampullary cancer, n=22) and 27 patients partial duodenopancreatectomy with only regional lymphadenectomy (Group B: cancer of the head of the pancreas, n=16, periampullary cancer, n=11).
Perioperative mortality was 6,5 % (n=5) respectively (Group A: n=3, Group B: n=2). All three deaths in Group A where due to erosion and bleeding of the hepatic artery, in Group B due to pancreatitis and pneumonia. The last 56 operations where performed without perioperative mortality.
The cumulative 1-,3-, 5- year-survival rates for patients with pancreatic cancer were 62,1%, 34,5%, 34,5 % for Group A and 41,3%, 34,4%, 22,9% for Group B. In patients with periampullary carcinoma the 1-, 3-, 5-year-survival rates were 71,1%, 42,2%, 23,6% for Group A and 80,8%, 80,8%, 80,8% for Group B.
The analyses according to the tumor stage showed, that for patients with smaller pancreatic carcinomas (stage I and II) the 5-year-survival rate could be improved by the extended lymphadenectomy (Group A: 58,3%, Group B: 38,9%)
Patients with stage III/IV tumors showed no improvement in survival (3-year-survival rate Group A: 23,8%, Group B: 28,3%, 5-year-survival Group A and B: 0 %).
In patients with periampullary carcinomas partial pancreaticoduodenectomy with only regional lymphadenectomy seems to be superior to partial pancreaticoduodenectomy with extended radical retroperitoneal lymphadenectomy, but might be explained by selection of early stages of Group B and small numbers of cases.

### F155

### CARCINOMA OF AMPULLA OF VATER. RESULTS IN 60 PANCREATICO-DUODENECTOMIES

C. Iacono*, G. Zamboni°, A. Scarpa°, E. Facci*, L. Bortolasi*, G. Prati*, G. Bogina°, G. Serio*
Departments of Surgery* and Pathology°, University of Verona, Verona, Italy

Pancreatico-duodenectomy (PD) is the treatment of choice of Carcinoma of Ampulla of Vater (CAV) . The purpose of this paper is to report our results in 60 patients that underwent PD between 1970 and 1994 for CAV. Forty-three patients were male and 17 were female. Mean age was 56.8 years (range 35-73). Jaundice was present in 48 pts, 14 of these underwent percutaneous, surgical or endoscopic biliary drainage before PD. Operative risk was estimated with ASA score: 25 pts were ASA I, 30 ASA II and 5 ASA III. The histology of the tumors were: 57 adenocarcinomas and 3 small-cell neuroendocrine carcinomas. Five adenocarcinomas were Stage I according Talbot Classification, 20 were Stage II, 11 were Stage III and 21 were Stage IV. The degree of differentiation was: well differentiated (Grade I) in 7 pts, moderately differentiated (II) in 22 pts, poorly differentiated (III) in 24 pts. Four cases were colloid carcinoma and the degree of differentiation was not assessed. The 3 small cell neuroendocrine carcinoma presented with regional lymph nodes metastases.
Global operative mortality was 6.6% (4 out of 60 pts). This rate was 16.6% (2 out of 12 pts) in the decade 1970-1980 and it was 4% (2 out of 48 pts) in the following period 1981-1994. Morbidity rate was 48% (surgical complication: 35%, medical complication: 30%).
The three pts with small-cell neuroendocrine carcinoma died at 6, 7 and 17 months because of disseminated metastases. The actuarial survival rate at 5 and 10 years was calculated in 51 pts with adenocarcinoma and it was 48.8% and 16.9% respectively (Kaplan-Meier method). The 5 years survival rate calculated according to the Stage was: 50% for Stage I, 73.6% for Stage II, 27% for Stage III and O% for Stage IV. The 5 years survival rate of 35 pts without lymph nodes metastases (Stage I, II, III) and of 16 pts with lymph nodes metastases (Stage IV) was 57.5% and 0% respectively; the comparison of these 2 groups was statistically significant (p-value< 0.001). The 5 years survival rate calculated according to the grading was: 80% for Grade I, 54% for Grade II and 26% for Grade III.
The scoring system, based on the sum of Grade and Stage, allows to divide the pts in 2 groups: group 1 (score 2-4) and group 2 (score 5-7). The 5 and 10 years actuarial survival was 91.6% and 32.7% respectively for group 1 and 23.9% and 11.9% for group 2 (p-value < 0.0003). The scoring system represents a useful way to evaluate the prognosis of pts with CAV.

### F156

### COMPARISON OF PANCREATOGASTROSTOMY AND PANCREATOJEJUNOSTOMY AFTER PANCREATODUODENECTOMY PERFORMED BY A SINGLE SURGEON

S.W. Kim, E.G. Youk, Y.H. Park
Department of Surgery, Seoul National University College of Medicine, Seoul, Korea

The anastomosis between the remaining pancreas and the intestinal tract after pancreatoduodenectomy(PD) has been the site of complications responsible for considerable morbidity and mortality. Pancreatogastrostomy(PG) has been recommened by a few surgeons for some theoretical and practical advantages over pancreatojejunostomy(PJ). The purpose of this study is to determine whether PG can be a safe alternative to PJ. Eighty-six PDs performed by a single surgeon for peri-ampullary cancers during a 4.5-year period from Mar. 1991 to Sep. 1995 were analyzed to compare early results of PJ(n=38) and PG(n=48). PG was performed at the low body posterior wall of the stomach by two-layer interruped suture. PJ was done by the conventional end to side method with pancreatic stump anastomosed to the antimesenteric border of proximal jejunal end. The two groups were comparable for age, sex, diagnosis, stage and operation time. In PJ group, anastomotic leakage developed in 6 patients(15.8%) and 3 of them(7.9%) died of complications related to leakage. In PG group 1 patient (2.1%) developed leakage and expired due to bleeding and 1 patient died of respiratory failure without any abdominal complication. Pancreatic leakage rate was lower in PG group than in PJ(P<0.05). Other major complications and incidence in PJ/PG group were biliary fistula(3/1), bleeding(0/1), abdominal abscess(2/2), delayed gastric emptying(0/2), choledocho-jejunostomy stricture(0/1), afferent-loop obstruction(1/0) and hepatic dysfuction(1/0), respectively and all these were improved conservatively except one case of bleeding that was not related to leakage. Overall morbidity/hospital mortality were 34.2/7.9% and 18.8/4.2% in PJ and PG group, respectively. No evidence of endo/exocrine insufficiency have been found during the follow-up except two who developed diabetes, one in PJ and the other in PG group. Suspicious marginal ulcer was detected in one patient in PJ group. In PG group, anastomotic patency could be confirmed by measuring amylase activity of gastric aspirate during early postoperative period. In conclusion, PG showed more favorable early outcome than PJ. Besides, considering technical feasibility and easy postoperative access, PG is recommendable procedure for reconstruction after PD.

### F157

### PANCREATICOGASTROSTOMY VERSUS PANCREATICOJEJUNOSTOMY WITH REGARD TO GASTRIC ACIDITY.

I. Kovacs.,P.Arkosy.,Mrs.Mahunka.,P.Sapy.
2nd.Department of Surgery and Laboratory Informatics University Medical School of Debrecen .

The indications of surgery and the operation of chronic pancreatitis with duct abnormalities is a contentious subject. The main indication for operation is the intractable pain . There are three main types of operation: partial or total resection of the gland, duct drainage and denervation. There are two main drainage method in painful chronic pancreatitis: the longitudinal pancreaticogastrostomy -which has not spread widely- and the conventional pancreaticojejunostomy. The aim of our prospective study to measure whether the pacreatic juice released into the stomach had any effect on gastric acidity compared both form of surgery and evaluate the results of surgical treatment.
Between 1992 and Oct. 95 , 43 patients with chronic pancreatitis were selected to this study, investigated and operated in our Surgical Department. 22 patients underwent pancreaticogastrostomy, 7 cases had pancreatico-cysto-gastrostomy and in 14 patients pancreaticojejunostomy was applied when a two layer wide anastomosis was created between the pancreatic duct and the antrum or the jejunum loop to relieve the symptoms. A 24 hours gastric monitoring was taken on every patient before and 6 weeks after the operation. The patients have received netilmicin prophylaxis before surgery. Following a complete postoperative check up was found that both types of operations are effective for pain relief (74%), the median pain scores reduced from 12o (3o-22o) to 40 (10-190). 83 % of the patients had no digestive problems due to pancreatic enzyme substitution. The average postoperative weightgain was 3.8 kg. The number of diabetic patients increased from 9 to 12. There was not perioperative death. Three patients needed reoperations.
According to our statistical evaluation of 24 hours gastric pH monitoring test no alteration was detected in gastric pH level in both groups pre- and postoperatively. It might be the lack of activation of the pancreatic enzymes at the anastomotic site by the low gastric pH level and the decreased pancreatic exocrin function. On the basis of pH measuring and evaluated data we can consider the pancreaticogastrostomy -which is a simple and quick procedure- is also a good operation choice to releive intractable pain in selected patients with chronic pancreatitis associated with duct dilatation.

### F158

PREDICTIVE VALUE OF CT-SCAN FOR CLINICAL OUTCOME IN ACUTE PANCREATITIS.

Ph.M. Kruyt[1], A.R. van den Blezenbos[2], K. Bosscha[1], M.S. van Leeuwen[2], M.A.M. Feldberg[2], H.G. Gooszen[1].

Departments of Surgery[1] and Radiology[2], University Hopital Utrecht, Utrecht, The Netherlands.

**Purpose of the studie.** Acute pancreatitis cannot adequately be classified by clinical or radiological criteria, or even by a combination of both. Established clinical scoring systems have major drawbacks. Evaluation is necessary within 48 hours after initiation of pancreatitis. Referred patients cannot be classified for that reason. Therefore we conducted a study to asses the additional value of established CT scores in comparison with the clinical Simplified Acute Physiology (SAP) score in predicting outcome in patients with acute pancreatitis.
**Patients.** Forty five patients underwent dynamic contrast-enhanced CT, scored according to Balthazar, the CT Severety Index (CTSI) and Schröder. The SAP score was calculated within 24 hours of the CT-scan. The predictive values of these for mortality, intervention and hospital stay ≥ 20 days were compared.
**Results.**

| Positive predictive value | Mortality | Intervention | Hospital stay ≥ 20 days |
|---|---|---|---|
| Balthazar D or E | 41 % | 84 % | 66% |
| CTSI ≥ 6 | 41 % | 84 % | 66 % |
| Schröder ≥ 6 | 50 % | 85 % | 55% |
| SAPscore ≥ 8 | 48 % | 83 % | 65 % |

| Negative predictive value | Mortality | Intervention | Hospital stay ≥ 20 days |
|---|---|---|---|
| Balthazar A - C | 92 % | 69 % | 69% |
| CTSI ≤ 5 | 92 % | 44 % | 66 % |
| Schröder ≤ 5 | 84 % | 69 % | 44 % |
| SAPscore ≤ 7 | 42 % | 45 % | 55 % |

**Conclusions.** For identification of patients with severe acute pancreatitis, by prediction of clinical outcome, there is no clear additional benefit of CT scores compared to SAP score. However, to exclude the possibility of severe pancreatitis, any type of CT scoring system is more useful than the SAP score. The data show that early CT-scan in acute pancreatitis is of limited clinical significance. CT-scanning should be limited to patients,concidered to be operated upon, because of detoriated clinical condition.

### F159

THE MANAGEMENT OF PANCREATIC NECROSIS IN A SPECIALIST REFERRAL CENTRE

Paul C Leeder, Iain G Martin, Michael J McMahon (MJM)
Department of Surgery, The General Infirmary at Leeds, UK.

A retrospective study of 144 patients with acute pancreatitis, treated consecutively over a seven year period by one consultant (MJM). 70 were admitted from our local population and 74 were transferred from other hospitals. The mean age was 53 years (17-91) and 90 patients were male. The aetiology was thought to be ethanol in 44 patients, gallstones in 51, idiopathic in 33 and due to other causes in 16 patients. Dynamic CT imaging was performed in 99 patients and formed the basis of all subsequent management. Pancreatic necrosis was diagnosed in 54 patients on CT. In 24 of these patients (44%), infection was demonstrated within the necrosis either by fine needle aspiration or subsequently at operation. One patient was found to have infected pancreatic necrosis which was not diagnosed at CT. Of the 144 patients, 74 were managed conservatively, 33 had radiological intervention and 33 required operation, which consisted of debridement of necrosis in 27 patients. Surgery was carried out a mean of 27 days after presentation with a mean delay of 11 hours (range 1 to 31) after a decision to operate was made. Forty of the 144 patients required intensive care support and 79 needed intravenous nutrition. Overall 26 of the 144 (18%) patients died. The best predictor of mortality was the APACHE II score on admission (p<0.001). 14 (56%) patients with infected pancreatic necrosis died compared with only 6 (20%) of those with demonstrated sterile pancreatic necrosis. 70% of patients undergoing pancreatic debridement died, a mean of 68 days from onset of their attack. The mean length of stay of patients surviving their attack was 44 days (range 1 to 173). At an estimated cost of up to £250,000 per patient, this very sick group of patients pose a formidable challenge and place a great demand upon therapeutic and support services.

### F160

THE INFLUENCE OF ANTIOXIDANTS ON BACTERIAL TRANSLOCATION IN ACUTE PANCREATITIS IN THE RAT

P. Leveau, X. Deng, V. Soltesz, Z. Sun, I. Ihse, R. Andersson
Departments of Surgery and Medical Microbiology,
Lund University, Lund, Sweden

Severe acute pancreatitis is associated with infectious complications and multiple organ failure. The present study was carried out to determine the incidence of bacterial translocation in severe experimental pancreatitis in the rat and the influence of an antioxidant (N-acetyl-L-cystein).
Acute pancreatitis was induced by the intraductal infusion of taurodeoxycholate in the rat. At induction of acute pancreatitis or sham operation the animals received either sterile saline or N-acetyl-L-cystein intravenously. Sampling for bacterial cultures from portal and caval vein blood, mesentéric lymph nodes, spleen, pancreas, liver, lungs, peritoneal fluid, distal small intestine and colon was performed 12 h after induction of acute pancreatitis.
The bacterial overgrowth in the distal small intestine noted 12 h after induction of acute pancreatitis was prevented by treatment with N-acetyl-L-cystein. The frequent bacterial translocation to especially mesenteric lymph nodes, but also to peritoneal fluid, liver, pancreas, spleen and lungs, was completely prevented by pretreatment with N-acetyl-L-cystein.
Severe acute pancreatitits in the rat thus results in changes in the bacterial ecology in the intestine with bacterial overgrowth and bacterial translocation, both to mesenteric lymph nodes and also systemic dissemination to blood and various organs. Treatment with the antioxidant N-acetyl-L-cystein prevented bacterial translocation, maybe by affecting the previously described permeability changes in the gut barrier.

### F161

PROGNOSTIC SIGNIFICANCE OF THE KI-67 ANTIGEN AND P 53 GENE MUTATIONS IN PANCREATIC CARCINOMA.

S. Linder[1], C. Parrado[4], U. Falkmer[4], M. Blåsjö[2], P. Sundelin[2], A. von Rosen[3].

Departments of Surgery[1]and Pathology[2], Stockholm Söder Hospital; Departments of Surgery[3] and Pathology[4], Karolinska Hospital, Stockholm, Sweden.

Survival time in patients with adenocarcinoma of the pancreas is short, also when radical surgery is attempted. Various biological properties may be responsible for the rapid tumor progression. In the present study the expression of proliferation associated nuclear antigen Ki-67 and p53 gene mutations were studied as prognostic factors.
Metods. Fifty-three patients, 17 men 65 years (45-83) and 36 women 66 years (45-83) underwent attempted curative resection of pancreatic carcinoma (50 Whipple resections, 3 pancreatectomies). All original specimens were re-evaluated and paraffin embedded material was used for immunohistochemical analyses. Monoclonal antibodies were used with the ABC technique, MIB-1 (Immunotech) which detects Ki-67 antigen and DO-1 (Dako) reacting with both wilde type and mutant p53. MIB-1 positive nuclei were detected with interactive image analysis.
Results. The median percentage of Ki-67 positive nuclei was 29,7 (0,5-82,1). Staining for p53 was negative in 22 tumors, positive in 26, and in 5 stainings were unsuccessful. Median survival time was 12,5 months (2,5-125), 4 patients being alive. High proliferation rate (>45% MIB-1 positive nuclei, n=14) correlated with shorter survival time (p< 0,01) as did the presence of p53 mutations (p<0,05).
Conclusions. High proliferation rate indicated by the Ki-67 antigen and the presence of p53 mutations correlates with shorter survival time in pancreatic carcinoma.

### F162

### CT CLASSIFICATION OF PANCREATIC TRAUMA

F Loria, S Cantoni*, G Loria, P Frosina, A Blandino, G Ascenti, C De Renzis, E Scribano, C Frola*.

Ist. Radiologia Univ. Messina-*IV Div. Radiologia Osp. S. Martino, Genova

Our goal is to assess the usefulness of CT in the classification of pancreatic trauma and for determining a prompt and correct diagnosis.

6 patients with pancreatic trauma were examined with CT. Scans were obtained after administration of both oral and intravenous urographic contrast agents. In all cases we repeated sequences at 4 mm slice thickness in the pancreatic region. Pancreatic trauma were classified in contusion, parenchymal lacerations with or without duct distruption and complete rupture.

2/6 patients showed, at CT scanning, a pancreatic contusion with enlargement of the pancreas and peri pancreatic edema in the region of the body of pancreas around the superior mesenteric artery. 2/6 patients exhibited parenchymal lacerations with peripancreatic fluid collections, in one cases was associated distruption of the main pancreatic duct. In 2/6 patients CT scans demonstrated complete rupture of the pancreas as a clear separation(fracture line) across the long axis of the pancreas. The fracture line was present across the neck of the pancreas ventral to the lumbar spine, with retroperitoneal fluid collections. Thickening of the left anterior perirenal fascia was seen in 2/6 cases. 3/6 patients had associated a traumatic injury involving the liver, the spleen and the duodenum.

Meticulous attention to the scanning technique is important to avoid false positive or false negative diagnosis or delay in the diagnosis which commonly leads to complications as pseudocyst or abscess. When diagnosis and surgery are prompt, as in our series, patients have a benign postopertive course.

### F163

### COMBINED PANCREAS-KIDNEY TRANSPLANTATION (PKT) AFTER PREVIOUS TRANSPLANTATION

J.A. Lowell, R.J. Stratta, R.J. Taylor, R. Sindhi, D. Sudan, P. Castaldo, Department of Surgery, University of Nebraska Medical Center, Omaha, Nebraska, USA

The purpose of this study was to report our experience with PKT in patients who have undergone previous transplant. From 4/89 through 11/95, a total of 133 PKTs were performed at our center. Eleven patients underwent PKT after previous kidney transplant, including one patient who underwent simultaneous pancreas and double kidney transplant. The mean recipient age was 40 years with a mean duration of diabetes of 27 years. Six had 1 previous kidney transplant alone (KTA), 4 had 2 previous KTA, and 1 had a previous PKT. The mean interval between transplants was 6 years. Before retransplant, 4 patients had undergone previous transplant nephrectomy (TN; n=3) or pancreatectomy (TP; n=1). At the time of retransplant, 8 patients had concomitant procedures including bilateral TN (n=2), unilateral TN (n=4), native nephrectomy (n=2), and splenectomy (n=1). Venous extension grafts on the kidney and/or pancreas were needed in 3 patients. Mean HLA-ABDr match was 2.3 with a mean kidney cold ischemic time of 16.7 hours. Mean operative blood replacement was 2.0 units, and the mean operating time was 6.2 hours. All but one of the patients received quadruple immunosuppression. The mean length of initial hospital stay was 20 days with a mean of 1.5 readmissions. There were 4 acute rejection episodes (1 pancreas, 3 kidney), all successfully treated with steroids. Seven major infections occurred (3 CMV, 2 line sepsis, 2 peritonitis). Patient survival is 100% with a mean follow-up of 37 months (range 2-65). Kidney and pancreas allograft survival are 91% and 73%, respectively. Three grafts (2 pancreas, 1 kidney) were lost early from thrombosis, with two (1 kidney, 1 pancreas) successfully retransplanted. No patient required dialysis after transplant, and all patients are currently dialysis-independent with a mean serum creatinine of 1.9 mg/dl. Conclusion: PKT after previous transplant is a challenging but safe procedure that often requires concomitant procedures, the use of vascular extension grafts, and atypical placement of the allografts. However, the excellent results justifies an aggressive policy of retransplantation in the diabetic patient with a failed or failing allograft(s).

### F164

### CYSTIC NEOPLASMS OF THE PANCREAS : STUDY OF 72 CASES

M.A.C. Machado*, G. Spiliopoulos*, P. Volpe**, A.L. Montagnini**, C. Stasik*, T. Bacchella**, J.E.M. Cunha**, J.P. Campion*, M.C.C. Machado**, B. Launois*.
Department of Surgery and Transplantation Unit.
*-University of Rennes, France. **- University of São Paulo, Brazil.

Cystic neoplasms are an uncommon group among pancreatic tumors. These lesions are seen more frequently in recent surgical practice, probably because of advances in diagnostic and surgical techniques. The aim of this study is to describe the diagnostic features and therapeutical options in the management of cystic tumor of the pancreas. We report here our experience with 72 patients with pancreatic cystic tumors over a ten-year period. Sixty-two four patients were women and ten were men. The mean age of patients was 55.2 years (range, 21 to 81 years).

Mild abdominal pain was the main symptom in 70 % of patients. The lesion were incidental finding in 10% of patients. CT scan provided the diagnosis of cystic tumor in 94% of patients while ultrasonography provided the same diagnosis in 78% of patients. All patients underwent surgical treatment. The pathological diagnosis was: thirty patients with mucinous cystadenoma (41.7%), thirty-two patients with serous cystadenoma (44.4%), ten patients with mucinous cystadenocarcinoma (13.9%). There was no operative mortality. Seven of ten patients with cystadenocarcinoma ultimately died of the disease. One patient with extended resection is still alive 3 years after surgery without recurrence of the tumor. The survival rate was 20.5% at 3 years. All patients with cystadenomas (mucinous or serous type) that underwent complete resection are alive or died from other causes.

Only complete resection of the cystic tumors of the pancreas provides certain pathological diagnosis, the best chance of cure and may remove the risk of malign transformation of the cystadenomas, particularly of the mucinous type, with minimum operative risk.

### F165

### THE PATHOLOGICAL DIAGNOSIS OF PANCREATIC CYSTADENOMAS IS POSSIBLE BEFORE SURGERY ? CLINICAL AND RADIOLOGICAL STUDY OF 60 PATIENTS

M.A.C. Machado*, G. Spiliopoulos*, P. Volpe**, A.L. Montagnini**, C. Stasik*, T. Bacchella**, J.E.M. Cunha**, J.P. Campion*, M.C.C. Machado**, B. Launois*.
*-University of Rennes, France. **- University of São Paulo, Brazil.

Cystic neoplasms represent up to 15% of cystic lesions of the pancreas. The aim of this study is to analyze retrospectively 60 cases of cystic tumors of the pancreas operated on between 1990 and 1995. There were 53 women and 7 men with mean age of 55.9 years. All patients were operated on. 27 patients presented a mucinous cystadenoma (45%), 24 presented a serous cystadenoma (40%) and 9 patients presented a mucinous cystadenocarcinoma. There were 12 pancreaticoduodenectomies, 2 total pancreatectomies, 28 distal pancreatectomies, 8 enucleation and 8 biopsies. There was no operative mortality. A preoperative diagnosis of serous cystadenoma was made in 15 patients, confirmed in 12 of them (80%). A preoperative diagnosis of mucinous cystadenoma was made in 19 patients, confirmed in 12 of them (63.2%) while 2 presented a cystadenocarcinoma. Undetermined cystic lesion was the diagnosis proposed for 16 patients. The final diagnosis was serous cystadenoma (n=10), mucinous cystadenoma (n=2) and cystadenocarcinoma (n=4). Ten patients received preoperative diagnosis of pancreatic pseudocyst, but in eight of them the peroperative diagnosis was correct. Unfortunately two cases were mistaken by pseudocysts and a drainage into a viscous organ was performed. Both patients were reoperated on after diagnosis of cystic neoplasm. The final diagnosis was mucinous cystadenoma (n=6) and cystadenocarcinoma (n=4) (Table 1).

Table 1 - Presumed preoperative diagnosis compared to final pathological diagnosis.

| | Serous cystadenoma | Mucinous cystadenoma | Cystadenocarcinoma |
|---|---|---|---|
| Undetermined cystic lesion | 10 | 2 | 3 |
| Serous cystadenoma | 12 | 3 | 0 |
| Mucinous cystadenoma | 5 | 13 | 2 |
| Pseudocyst | 0 | 6 | 4 |

CONCLUSION : The pathological diagnosis of cystic neoplasm is still not possible before the surgery. Resection should be the treatment of choice.

### F166

GASTRIC MOTILITY UNDER DELAYED GASTRIC EMPTYING AFTER PYLORUS PRESERVING PANCREATICODUODENECTOMY IN DOGS

D. Maeda, S. Takahashi, M. Kitajima, Y. Ogata*, M. Fujisaki**
Department of surgery Keio univ. school of medicine, Tokyo,
* Tochigi cancer center, Utsunomiya, **Ashikaga red cross hospital, Ashikaga, Japan

INTRODUCTION We have often experienced delayed gastric emptying after pylorus preserving pancreaticoduodenectomy (PPPD), but the mechanism of the delayed gastric emptying is still unclear. Therefore we have investigated the cause of that by dogs. MATERIAL AND METHODS PPPD was performed on the nine mongrel dogs. Reconstruction was carried out by modified Cattel's procedure. The right gastric artery and the pyloric branch of the vagus nerve were preserved. Straingage force transducers (S.G.T) were placed on the stomach body, antrum and the jejunum. On the 7th postoperative day we gave five dogs barium and meal, and confirmed the patency of the pylorus ring by X-ray. On the 7th, 14th and 28th postoperative days we studied the contraction of the stomach and the jejunum for 24hrs and measured plasma motilin concentration in four dogs. Five normal dogs were implanted S.G.T. on the stomach body, antrum and the duodenum to obtain control measurements of gastrointestinal motility and measured plasma motilin concentration. RESULTS Pylorus ring patency : In all dogs given barium and meal we could see it passed through the pylorus ring and confirmed the patency of that. Motor activity of the stomach : On the 7th postoperative days only irregular and short contractions were observed. The interdigestive myoelectrical complex (IMC) of the stomach did not appear. On the 14th postoperative day bands of strong contractions like the IMC appeared. But the duration and the cycle were different from the IMC significantly. Plasma motilin concentration : Plasma motilin concentration when the bands of strong contractions appeared did not differ from those of other phases. CONCLUSION In the dogs undergone PPPD the pylorus was open and the disorder of the gastric motility was the cause of the delayed gastric emptying after PPPD. On the 14 postoperative day the bands of strong contractions like the IMC appeared. It was supposed that appearing those contractions showed the process of recovering from the delayed gastric emptying. It was speculated the low motilin concentration gave rise to the disorder of the gastric motility.

### F167

**PANCREATIC PSEUDOCYSTS ASSOCIATED WITH CHRONIC PANCREATITIS - early and late results of 1367 operations.**

**A. Magyar, L. Flautner, I. Pulay, T. Tihanyi, L. Harsányi**
First Department of Surgery, Semmelweis University Medical School, Budapest, Hungary

The authors performed 1367 operations on 1155 patients for pancreatic pseudocysts complicating chronic pancreatitis during 7 years between 1987 and 1993.
In 38% of the cases an acute exacerbation of the previously known chronic pancreatitis have been detected, whereas in the remaining 62% the pseudocysts constantly persisted without new onset of acute inflammatory changes. The surgical procedures of choice were drainage in 88%, resection in 12%. The majority (73%) of drainage procedures were internal, directed towards the gastrointestinal tract. Based on the favourable results the authors preferred anastomoses performed between the pseudocyst and stomach (posterior cystogastrostomy), as well as the pseudocyst and duodenum (blunt forced cystoduodenostomy). Additionally at the same time efforts have been made to resolve the morphological alterations of the duct of Wirsung caused by chronic pancreatitis. This has been achieved by pancreatic duct decompression procedures such as cysto-Wirsungo-gastrostomy, and sphincteroplasty of the papilla of Vater. (53% of the operations were such combined interventions.)
The early postoperative morbidity was 12.9%, mortality was 1.46%. The early mortality was significantly increased by complications requiring reoperations, such as insufficiency of the anastomosis, haemorrhage, and abscess formation. There were no statistically significant differences regarding complications and early death rate between the combined and non-combined procedures.
In the patient group (n=700) that have undergone operations in the first 5 years a long term follow up using standardised questionnaires was completed. 87% of the answers were satisfactory for evaluation with an average of 44 months follow up period. The late recovery results were found to be excellent in 23%, good in 36%, satisfactory in 30% and poor in 11%. The late mortality after 4 years follow up was found to be 15.5%.

### F168

INDICATIONS OF FIBRIN SEALING IN PANCREATIC SURGERY WITH SPECIAL REGARD TO OCCLUSION OF A NON ANASTOMOSED PANCREATIC STUMP WITH FIBRIN SEALANT

AP Marczell, Chirurgische Abteilung,
Hanusch-Krankenhaus, Heinrich Collin-Str. 30, A-1140 Vienna

Due to the specific properties of pancreatic tissue the complication rate is high. The bad results has prompted us to apply fibrin sealant increasingly in pancreatic surgery in the following indications: (1) wedge excision and enucleation; (2) tail resection and left pancreatectomy; (3) resection of head of pancreas, with or without pancreatojejunostomy; (4) drainage operation: and (5) traumatic lesions.

1. Fibrin sealing allows seamless sealing of the tissue defect.
2. Following tail resection or left pancreatectomy, the resection surface is trimmed in the form of a fish mouth: the gaping wound edges are approximated and sealed, and the "lips" are covered with an additional coat of the fibrin sealant.
3. Following Whipple's operation for chronic pancreatitis both anastomoses - hepatico- and pancreaticointestinal - are sealed with fibrin sealant. In cancer surgery the remaining pancreas is occluded with fibrin sealant via the pancreatic duct. The pancreatic duct is ligated and the resection surface sealed with an additional layer of fibrin sealant, eventually in combination with collagen fleece. In 92 patients there was no lethal case. As to local complications, there was one case of a clinically necrosing remaining pancreatitis and 9 cases of postoperative pancreas fistulae.
4. In interventions to secure an internal drainage the anastomosis can be sealed with a cuff of fibrin sealant.
5. Lesions of the tail, possibly perforating: combined head and corpus lesions with intact main duct; and management of all surfaces.

In 301 applications of fibrin sealant we have had only 20 failures. The high fibrinolytic activity of the pancreatic tissue requires a aprotinin concentration of the sealant of at least 10.000 KIU/ml to avoid premature dissolutation of the fibrin.

### F169

CYSTIC TUMOURS OF THE PANCREAS: OUR EXPERIENCE WITH 40 CASES

Marrano D., Casadei R., Greco V.M., Taffurelli M., Minni F.
1st Surgical Clinic, University of Bologna, Bologna, Italy

Cystic pancreatic tumours are uncommon. In the 1st Surgical Clinic of the University of Bologna, from 1975 to 1995, 40 cases have been treated: they represent 7.5% of all pancreatic neoplasms (532 cases) and 24.4% of all cystic pathologies seen at our Institute during this period. Different histopathological types of cystic tumours were observed of which 19 (47.5%) were malignant (19 mucinous cystadenocarcinomas), 16 (40.0%) benign (7 serous cystadenomas, 6 mucinous cystadenomas, 2 lymphangiomas, 1 cystic insulinoma) and 5 (12.5%) of uncertain malignancy (3 intraductal papillary neoplasms, 2 solid and cystic tumours). Of the 40 cases, 35 (87.5%) underwent surgical resection, of which 19 left pancreatectomy, 8 pancreatico-duodenectomy (4 according Child, 4 Traverso-Longmire), 4 exeresis of neoplasia, 3 subtotal pancreatectomy and, finally, 2 total pancreatectomy. Resection of benign and uncertain malignancy cystic tumours was always possible (resecability index = 100%); in mucinous cystadenocarcinomas, instead, pancreatectomy was performed in 14 out of 19 cases (resecability index = 73.6%). In these patients two-year and five year survival was respectively 46.2% and 36.9%. Of the 16 patients with benign cystic tumours, 1 died of postoperative complication and 15 were still alive and well at a mean follow-up time of 45 months (range 4-132 months). Three patients with intraductal papillary neoplasms and two with solid and cystic tumours of the pancreas were still alive and well at a mean follow-up time of 68 months (range 1-147 months). For all cases two-year and five-year actuarial survival was respectively 71.1% and 62.2%. These results indicate that cystic tumours of the pancreas have a high index of resecability (87.5%) and a relatively good prognosis with respect to solid pancreatic neoplasms. For these reasons careful differential diagnosis is essential and, in the case of positivity for cystic tumours, aggressive surgical treatment must be done.

### F170

**ENDOCRINE PANCREATIC TUMOURS: OUR EXPERIENCE WITH 59 CASES**

Marrano D., Greco V.M., Casadei R. Taffurelli M.
1st Surgical Clinic, University of Bologna, Bologna, Italy

Endocrine pancreatic tumours are rare. In the 1st Surgical Clinic of the University of Bologna from 1975 to 1995, 59 cases were observed: they represent 11.1% of all pancreatic neoplasms (532 cases). Two groups of endocrine tumours were recognized: functional and non functional. Functional endocrine tumours were distinguished in 21 insulinomas, 15 Zollinger-Ellison syndrome, 2 Vipomas, 1 glucagonoma, 1 somatostatinoma and 1 PPoma. Insulinoma were benign in 17 cases, malignant in 2 cases and, finally, in 2 cases on iperplasia of B cells was shown. All were resected (14 left pancreatectomy with splenectomy, 6 enucleation and 1 left pancreatectomy with hepatic resection of metastasis). Zollinger-Ellison syndrome were supported by a gastrinomas in 9 (64.2%) cases; in 5 (35.8%) gastrinomas were occult; in 1 case iperplasia of G antral cells was shown. All identified gastrinomas were resected. The other very rare functional endocrine tumours were all resected; in 1 case (somatostatinoma) with hepatic resection of some small metastasis and in another one (vipoma) with resection of retroperitoneal lymph nodes. Non functional endocrine tumours were resected in 15 out of 18 cases (index of resecability=83.3%) (8 left pancreatectomy, 3 enucleation, 2 pancreatoduodenectomy and 2 intermediate pancreatectomy). Of the 59 endocrine pancreatic tumours 32 (54.2%) were benign, 27 (45.8%) malignant. Resection of benign endocrine tumours was always possible while a pancreatectomy was performed in 24 out of 27 malignant tumours (index of resecability = 88.8%). In 3 cases pancreatic resection was associated to hepatic metastatic resection. In all cases postoperative course were uneventful with relief of the endocrine symptoms. Five-year survival were 100% for benign tumours, 60.2% for malignant. These data indicate that endocrine pancreatic tumours have an high index of resecability (95%). Moreover it is important to resect also hepatic metastasis when it is possible because of relief of endocrine symptoms and relatively good prognosis due to slow growing of these tumours.

### F171

**EFFECT OF EARLY JEJUNAL FEEDING ON THE SEPTIC COMPLICATIONS IN ACUTE PANCREATITIS**
**A prospective, randomized study**

Oláh A., Pardavi G., Varga Gy., Rácz I.,

Dept. of Surgery, Petz Aladár County Hospital, Győr, Hungary

**Purpose of study:** The necrotized tissue of the pancreas is colonized by bacteria mainly from the colon which may lead to abscess or infected necrosis. Our purpose was to prove, that early jejunal feeding – decreasing the paralytic condition and distension of colonic wall, starting the passage and preserving the normal colonic bacterial flora – can reduce the rate of septic complications.

**Patients and method:** The study included 38 patients with acute, non-biliary pancreatitis, randomizing into two groups. In group A (n=18) jejunal feeding was started in the first 24 hours (second jejunal loop; Survimed OPD, Fresenius). In group B (n=20) total parenteral nutrition was applied. Between the two groups neither in the male:female ratio (13:5 and 18:2), nor in the average age (48.2 and 44.7 years) nor in the etiology (13 and 16 alcoholic; 5 and 4 idiopathic) were significant difference found.

**Results:** Necrosis developed altogether in 14 patients (36.8 %). In group A two infected and three sterile necrosis were detected. In group B four infected, one sterile necrosis and four abscess were found. Septic complication due to bacterial contamination developed so in 2 cases in group A (11 %) and in 8 cases in group B (40 %). Statistical difference is significant (p=0.047; Fisher-test). In group A four patients, in group B eight patients underwent operating procedure. Mortality rate was 5.5 % in the group of jejunal feeding and 10 % in the control group.

**Conclusion:** Our results suggest that early jejunal feeding in the treatment of acute pancreatitis can reduce the rate of bacterial contamination of necrotized pancreatic tissue mainly in the later phase of disease, after the first week.

### F172

**DIAGNOSTIC AND SURGICAL MANAGEMENT OF INSULINOMAS.**

C. Pasquali, C. Sperti, ^ G. Liessi, S. Pedrazzoli.
Semeiotica Chirurgica - Univ. of Padua, ^ Radiology-Castelfranco V. Hosp., Italy.

From 1966 to 1995, 70 patients with organic hyperinsulinism (29 M, 41 F) were observed in our Department. Sixty five patients underwent surgery. Seven/65 patients had 2 operations (3 of them referred after surgery from other centers). Preoperative arteriography (AG) was performed in 61 cases; ultrasonography (U.S.) was performed in 26, computed tomography (C.T.) in 37, and magnetic resonance (M.R.) in 17 cases. Transhepatic portal sampling (P.V.S.) was performed in 28 patients. Four cases had an intra-arterial injection of calcium (I.A.C.I.). Six patients underwent 111-In octreotide scintigraphy. Intraoperative US was performed in 28 patients. Sensitivity of AG was 35.4%; we had 7 false positive tests (10.8%). The tumor was detectable with C.T. in 37.8% and in 46.2% with U.S. scan; false positive results occurred 3/37 (8.1%) and 1/26 (3.8%) cases respectively. M.R. was positive in 10/17 patients (58.8%). Octreotide scintiscan localized a single (1/6) 2 cm. tumor. P.V.S. gave positive results in 82.1% of cases. I.A.C.I. was positive in 2 cases. In the last 12 years (1984-95; 29 patients) AG, C.T. and U.S. showed a correct positivity rate of 41%, 45% and 52% respectively, however, only 18/29 patients (62.0%) underwent surgery with a correct pre-operative imaging information. Seventy-two laparotomies were performed in 65 patients: 5 explorations (2 for metastases), 2 pancreatico-duodenectomies, 23 tumor excisions, 36 left pancreatectomies (18 with spleen preservation ), 4 atypical pancreatic resections and 2 near-total pancreatectomies. Seven patients required reoperation. In 73.3% of the laparotomies a tumor was felt or seen. In 3 cases, additional undetected adenomas were resected by chance. Overall negative surgical exploration rate was 27.7% (20/72 cases) and in 14/20 the preoperative imaging was wrong or negative. Operative U.S. correctly localized the tumor in 22/28 (78.6%) patients detecting also 2 "occult" insulinomas, but had 2 false positive (7.1%). Only 51/65 patients (78.5%) had a typical single adenoma. Five (7.6%) had multiple adenomas and 5 had hyperplasia or nesidioblastosis. In 26.1% of patients the tumor size was < 1 cm. Two cases had liver metastases and in 2 patients the tumor was not found. Excluding the cases with metastases, the outcome of the patients was as follows: 60/63 (95.2%) were cured after surgery (1 of them recurred 3 years later); 2/63 (3.2%) were unchanged, and 2/63 developed diabetes after a near-total pancreatectomy (including the case who recurred). Morbidity was 25% and the hospital mortality was 5.5%. Only 1 patient died after surgery from 1977 (51cases) for causes unrelated to surgery.

### F173

**ULTRASONOGRAPHIC EVALUATION OF THE COMMON BILE DUCT IN BILIARY ACUTE PANCREATITIS PATIENTS. A COMPARISON WITH ENDOSCOPIC RETROGRADE CHOLANGIOPANCREATOGRAPHY.**

R. Pezzilli, N. D'Imperio, B. Barakat, P. Billi, D. Baroncini, A. Piemontese.
Emergency Department, St. Orsola Hospital and Department of Digestive Endoscopy, Bellaria Hospital, Bologna, Italy.

In patients with acute pancreatitis, evaluation of the morphology and contents of the biliary tract is essential for diagnosis and optimal management of the disease. The aim of this study was to compare the morphological findings pertinent to assessment of the common bile duct by ultrasonography (US) and endoscopic retrograde cholangiopancreatography (ERCP) in patients with biliary acute pancreatitis. Thirty-one patients were studied (10 males, 21 females, mean age 67 years, range 40-90); the diagnosis of acute pancreatitis was made on the basis of characteristic abdminal pain associated with an elevation of serum amylase and lipase. US was performed in all patients on admission to the hospital and all subsequently underwent urgent ERCP and endoscopic sphincterotomy within 24 hours. US was performed with an Ansaldo AU 450 apparatus, using a 3.5 MHz transducer; ERCP was performed using a Fuji ED7-XU2 duodenoscope. For the purpose of the study, the endoscopist was kept unaware of the morphological details reported by the sonographer. US showed choledocolithiasis in 2 patients and microlithiasis of the common bile duct in 12. ERCP showed choledocolithiasis in 4 patients and microlithiasis of the common bile duct was detected in 20. The mean of the common bile duct diameters determined by US was 7.6 mm (range4-12 mm), which was significantly smaller ($P<0.001$) than the value obtained with ERCP (mean value 10.2 mm, range 4-17.5 mm). For this parameter, there was good correlation between the values obtained with the two techniques ($r=0.74$, $P<0.001$). In 16 cases (52%) there was concordance between US and ERCP in the detection of lithiasis and/or microlithiasis of the common bile duct. The results show that in patients with biliary acute pancreatitis: 1. Attendable values of the common bile duct diameters can be obtained with either US or ERCP. The significantly larger values given by ERCP are attributable to the presence of contrast medium in the duct lumen. 2. US is the initial technique of choice: it is non-invasive and it is the preferred means for demonstrating cholelithiasis. 3. ERCP shows a higher sensitivity than US in detecting lithiasis or microlithiasis of the common bile duct.

### F174

### CT-SCAN ACCURACY IN THE DIFFERENTIAL DIAGNOSIS OF PANCREATIC CYSTIC LESIONS.

R. Polverosi, * C. Sperti, * C. Pasquali, E. Zanellato, # A. Caroli, * S. Pedrazzoli.
Radiology and # Medicine, Montebelluna Hospital -*Institute of Semeiotica Chirurgica, University of Padua, Italy.

Preoperative differentiation of pancreatic cysts is important for appropriate treatment. Cystic neoplasms of the pancreas are observed with increasing frequency, often as incidental finding. CT-scan is the most common radiologic investigation for preoperative detection of pancreatic lesions, but misdiagnosis of pancreatic cysts is relatively frequent. We reviewed our experience of CT-scan in 63 patients with 69 cystic lesions of the pancreatic area, observed from May 1988 to March 1995. We evaluated the accuracy of CT-scan to distinguish pancreatic or extrapancreatic lesions, to differentiate between benign and malignant cysts, and to identify different type of cysts. The final diagnosis included: 19 pseudocysts (28%), 6 serous cystadenomas, 20 mucinous neoplasms (29%; 5 adenomas, 12 carcinomas,3 IHMN), 9 ductal carcinomas, 3 endocrine tumors, 1 papillary cystic tumor, 1 lymphoma, 1leiomyo-sarcoma, 2 solitary true cysts, 7 extrapancreatic lesions (10%).
CT findings correctly identified 5/7 extrapancreatic lesions, and 52/69 (75%) as benign or malignant pancreatic lesions. Sensitivity of CT-scan in detecting benign pancreatic tumor was 79% and malignant was 73%. Among different type of cyst CT correctly identified 15/19 pseudocysts (79%), 14/20 mucinous neoplasms (70%), 3/6 serous cystadenomas, 7/9 ductal carcinomas (78%). None of the other rare types of tumor was correctly identified. In 8 % of cases the radiologic diagnosis was changed when the clinical history of the patient was known.
In 25% of patients with a cyst of the pancreatic area CT-scan in not able to differentiate the type and the malignant nature of the cyst. In our series 12% of cases were rare types of pancreatic lesion and in these patients there is no chance to recognize the nature and behaviour of the cyst.

Study supported by the Italian National Research Council (C.N.R.), contract nr. 94.01179.PF39.

### F175

### ELECTROGASTROGRAPHIC AND pH-MONITORING OF PATIENTS FOLLOWING CONVENTIONAL AND PYLORUS PRESERVING PANCREATODUODENECTOMY.

Popiela T., Matyja A., Thor P., Herman R.M., Plebankiewicz S., Lorens K., Kawiorski W.,
*Ist Dept. of General and GI Surgery Jagiellonian University, Cracow, Poland.*

Pylorus preserving pancreatoduodenectomy (PPP) has been proposed as an alternative procedure to conventional Whipple procedure (CWP).

The aim of this study was to evaluate the gastric motility and Ph using modern methods of studies.

Patients: Two groups of patients, each consisted of 20 persons were examined before and after surgery - group I - patients following PPP and group II - patients after CWP.

Methods: Transcutaneous electrogastrographic examination and 24-hour Ph monitoring was performed in each patients before and up to one year after surgery.The following EGG patterns were evaluated in fasting and postprandial state: slow waves time and space distribution, percentage of normal slow waves, percentage of gastric dysarrhytmias. During the 24hr pH monitoring the number of reflux of jejunal contents episodes and its duration were evaluated.

Results: In PPP the frequency of gastric dysarrhytmias is lower than following CWP. The dominant frequency of SW increased up to 10 ± 2 cpm which occupied 56% of the examination time. In CWP the significant decrease in SW frequency occurred, although in symptomatic patients the aboral propagation of jejunal pacemakers with frequency of 12 cpm were observed.

Conclusion: EGG evaluation and continuous 24-h monitoring appeared to be useful methods in diagnosing and monitoring of motility sequels following surgery. The frequency of motility disturbances following PPP was significantly lower than after CWP.

### F176

### THE FREY OPERATION FOR CHRONIC PANCREATITIS

J. Ramos, D. Bizos, M. Lebos, J. Myburgh
Dept. of Surgery, Univ. of Witwatersrand, Johannesburg, South Africa

The surgical strategy used in treating symptomatic chronic pancreatitis, namely drainage or resection, has generated much controversy. The Frey modification of the Puestow procedure combines the principle of wide drainage of the pancreatic duct with in situ resection of the head of the pancreas. The aim of this study was to prospectively assess the results of the Frey procedure in our patient population, this being the procedure of choice since early 1992.
Between March 1992 and November 1995, 24 patients underwent the Frey procedure for symptomatic chronic pancreatitis. There were 20 males and 4 females, mean age was 38.6 years (29-48). The aetiology was alcohol in 21, and idiopathic in 3. Pain alone was the presenting symptom in 17 (71%) patients, 6 had pain and jaundice, and one had jaundice alone. Six patients (25%) had previous pancreatic surgery, 3 having a distal pancreatectomy, and 3 a Puestow procedure. Exocrine insufficiency was present in 25% preoperatively, and 37% were diabetic. The Frey procedure was performed alone in 12 patients (50%), 11 were combined with biliary drainage (46%), and 1 with distal pancreatectomy. Significant morbidity requiring reoperation occurred in two patients, one an early adhesive obstruction, and in another major bleeding from the pancreatic bed occurred two weeks following surgery. Overall morbidity was 42% and mostly minor, respiratory problems being the commonest. One patient died of intra-abdominal sepsis following leakage of an entero-enterostomy, an avoidable complication (mortality 4%).
Of the 23 survivors, 4 have been lost to follow-up. In the remaining 19 patients, mean follow-up time is 13.2 months (range 1-41). Relief of pain has been good or excellent in 18 patients (95%), and poor in 1. No recurrence of jaundice has occurred in any of the patients. One of 12 patients seen at follow-up had developed new symptomatic exocrine insufficiency. No new cases of diabetes were detected following the surgery.
In conclusion, the early results of the Frey procedure have been promising. Serious morbidity is low and the one death in the series was avoidable. Although follow-up is short, very good relief of symptoms has been achieved. No significant deterioration in exocrine or endocrine function was detected following this operation.

### F177

### CYSTIC TUMOURS OF THE PANCREAS

D. Rodriguez Romano, R. Gomez Sanz, A. Lopez Labrador, M. Manzanera, C. Castellon, E. Moreno Gonzalez.

Introduction:

The cystic tumours of the pancreas are not very common. Must be done a differential diagnosis with pseudocysts and the treatment is always surgical.

Material and Methods:

We reported on a retrospective trial of 9 patients with CTP operated on during the last 10 years. Two were serosous cystoadenomas, 5 mucinous cystoadenomas and 2 cystoadenocarcinomas. Eight were females and [illegible] was male, with a median age of 47,2 years (28-72). The cystic character of the [illegible]T was preoperatively diagnosed (laparoscopy, ultrasonography CAT scanner) but not,even the fine needle aspiration can establish a certain diagnosis.

Results:

Depending on the tumour localization the surgical treatment is performed, carrying out 6 distal pancreatectomies (in 2 cases with splenic preservation) and 3 cephalic duodenopancreatectomies(CDP). During the postoperative period one patient died, a 72 years old male with CDP due to cystadenocarcinoma, of caute renal insufficiency. There were no complications in the postoperative period with the remaining patients. After a follow-up, varying from 4 months and 10 years (3 years), there was recurrence in one case, the patient subsequently died. One patient died due to unknown causes. The remaining (6 cases) still alive.

Conclusions:

1) The cytological and radiological criteria are not enough to achieve a preoperative diagnosis of the CPT.

2) These tumours must be treated surgically. It is curative in benign forms and offers good results in malignant cases regarding non-cystic types of adenocarcinomas.

### F178

### TRAUMATIC INJURIES TO THE PANCREAS

MD Shahrudin & SM Noori
Department of Surgery, Faculty of Medicine
University of Malaya, 59100 Kuala Lumpur, MALAYSIA.

Pancreatic injuries from blunt trauma are infrequent, and their diagnosis and management can be difficult. Over the last 5 years we treated 13 patients with major pancreatic injuries from blunt trauma. 12 were involved in MVA, none of whom were wearing seat-belts. 1 assault. Only 5 patients had physical findings suggesting intra-abdominal injury. Serum amylase were elevated in 7 of the 9 patients tested. CT demonstrated injury in 3 of 4 patients scanned. 1 diagnosed by u/s. 5 patients who had DPL suffered other concomitant injuries that produced haemoperitoneum. Injuries were equally distributed throughout the pancreas.All patients underwent celiotomy. 8 required no operative management other than drainage. ISS averaged 28.5 $\pm$ 2.6 & mean hospitalisation was 31 $\pm$ 9.8 days. 1 patient with delayed diagnosis of pancreatic injury developed pseudocyst, but all other complications and prolonged hospitalisations were due to injuries to the head, chest, other abdominal organs & extremities. All patients survived. The diagnosis of pancreatic injury requires a high index of suspicion, and diagnostic studies may demonstrate only subtle signs of injury. Most injuries can be managed by localised resection and/or drainage. In contrast with pancreatic injuries from penetrating trauma, blunt pancreatic injuries are not associated with major vascular injuries, and therefore have a lower mortality risk.

### F179

### SERUM TUMOR MARKERS AND CYST FLUID ANALYSIS ARE USEFUL FOR THE DIAGNOSIS OF PANCREATIC CYSTIC TUMORS.

C. Sperti, C. Pasquali, # P. Guolo, ^ R. Polverosi, § G. Liessi, S. Pedrazzoli
Semeiotica Chirurgica, University of Padua, #Clinical Laboratory and Radiology,^Montebelluna Hospital and § Castelfranco V. Hospital, Italy.

Preoperative differential diagnosis of cystic lesions of the pancreas may be difficult because there are no reliable clinical or radiological criteria to assist in making the differentiation. The crucial point is to recognize mucinous and/or malignant tumors, in which a surgical resection is mandatory.Recently, analysis of aspirated cyst fluid for enzymes (amylase, lipase), tumor markers and cytology has been used to provide a preoperative diagnosis of pancreatic cysts.
Aim of the study was to evaluate the utility of serum and cyst fluid analysis for enzymes (amylase and lipase) and tumor markers (CEA, CA 19-9, CA 125, CA 72-4) in the differential diagnosis of pancreatic cystic lesions.
Serum and cyst fluid were obtained from 48 patients with pancreatic cyst (21 pseudocysts, 14 mucinous cystic neoplasms, 6 ductal carcinomas, 7 serous cystadenomas), observed from 1989 to 1994. Preoperatively, a basal sample of blood was collected and the serum was kept frozen until assay. The cyst fluid was collected by percutaneous fine needle aspiration in 20 cases, and by intraoperative aspiration in 28. The tumor markers were measured using commercially available double antibody immunoassaies (CEA: n.v. < 5 ng/ml; CA 19-9: n.v. < 37 U/ml; CA 125 n.v. < 35 U/ml; CA 72-4, n.v. < 4 U/ml).
Serum CA 19-9 levels were significantly ($p < 0.05$) higher in ductal carcinomas (all > 100 U/ml) and mucinous cystic neoplasms. CA 72-4 cyst fluid levels were significantly higher in mucinous cystic tumors ($p < 0.005$) with 95% specificity and 80 % sensitivity in detecting mucinous or malignant cysts. Combined assay of serum CA 19-9 and cyst fluid Ca 72-4 correctly identified 19/20 (95 %) of (pre-)malignant lesions, with only one (3.6 %) false positive. Cytology correctly classified all 21 pseudocysts as inflammatory lesions. Mucin-containing epithelium were found in 8/14 MCN and malignant cells in 3/6 ductal carcinomas. Cytology showed a sensitivity of 48% and specificity of 100%.
Any pancreatic cyst with high serum CA 19-9 values, or positive cytology, or high CA 72-4 in the fluid should be considered for resection.
Study supported by C.N.R., contract nr. 94.01179.PF39.

### F180

### A SCORING SYSTEM FOR EARLY DIFFERENTIATION OF ACUTE PANCREATITIS ETIOLOGY

D.Štimac, M.Rubinič, T.Lenac, D.Kovač, A.Včev,D.Miletič

Clinical Medical Center Rijeka, Internal Clinic, Gastroenterology Division, University of Rijeka, Rijeka, Croatia

The purpose of this retrospective study was to find a new scoring system for early differentiation of the two most common acute pancreatitis etiologies: biliary and alcoholic, because biliary pancreatitis can be treated early by endoscopic sphyncterotomy, whereas such treatment is unnecessary in alcoholics. A hundred and forty-five patients (57 males and 88 females) with diagnosis of acute pancreatitis based on a combination of clinical features, a typical case history, elevation of serum pancreatic enzymes and confirmation with imaging studies (ultrasonography or contrast enhanced computed tomography), satisfied requirements for participation in the study. Seven parameters (serum amylase, aspartate aminotransferase /AST/, alanine aminotransferase /ALT/, alkaline phosphatase /ALP/, urine amylase, lipase/amylase ratio /L/A/and erythrocyte mean corpuscular volume /MCV/ that differ (statistically significant, $p<0.001$) between patients with biliary and alcoholic pancreatitis were included in the scoring system. Each parameter according to its values was counted as 0 or 1, so the patients reached scores from 0 to 7. Score $\geq 4$ differs biliary pancreatitis from alcoholic with sensitivity of 92,92%, specificity of 93,75%, positive predictive value of 98,11% and negative predictive value of 76,92%. We conclude that our new scoring system calculated from routine laboratory parameters could be important support in early differentiation of acute pancreatitis etiology because it is non-invasive, fast and inexpensive.

### F181

### EXPERIENCE WITH 59 CONSECUTIVE SOLITARY PANCREAS TRANSPLANTS (PTX)

R.J. Stratta, R.J. Taylor, I.S. Gill, L.G. Weide, D. Sudan, R. Sindhi, P. Castaldo, J.T. Jerius, K. Cushing, K. Frisbie, M.T. Grune, S.J. Radio
Department of Surgery, University of Nebraska Medical Center, Omaha, Nebraska, USA

From 3/91 through 11/95, we performed 59 solitary PTXs (17 sequential pancreas after kidney, 42 PTXs alone) in 54 adult Type I diabetic patients. Indications for solitary PTX were: 1) the presence of 2 or more overt diabetic complications; and/or 2) glucose hyperlability with hypoglycemic unawareness and impaired quality of life. The recipient group consisted of 28 men and 26 women with a mean age of 38 years (range 25-62) and a mean duration of diabetes of 27 years (range 14-52). The recipient evaluation emphasized the documentation of diabetic complications, as well as adequate cardiac and renal reserve. Organ acceptance was restricted to ideal donors and mandated a minimum of a 2 antigen match (mean ABDR match 2.5). The mean cold ischemia time was 16.6 hours. Whole organ PTX was performed with bladder drainage. Ten patients (17%) received pancreas retransplants. All patients received systemic anti-coagulation intra-operatively, anti-platelet therapy post-operatively, and were managed with either triple or quadruple immunosuppression. Monitoring included prospective urine cytology as well as protocol and clinically indicated cystoscopic transduodenal needle biopsies. Twenty-six cases were managed with either FK506 induction (11) or rescue (15) therapy. The mean initial length of hospital stay was 16.7 days, and mean hospital charges were $100,664. The incidence of rejection, surgical complications, and major infections was 71%, 47%, and 59%, respectively. Actuarial 1 year patient and graft survival is 90% and 62%, respectively. All patients with functioning grafts have excellent metabolic control (mean glycohemoglobin level 5.1%) and have achieved good rehabilitation without major cardiovascular, renal, or progressive diabetic problems. **Conclusion:** Despite morbidity, solitary PTX can be performed with improving success, enhances quality of life, and offers an opportunity to arrest secondary diabetic complications.

### F182

**EXTENDED PANCREATIC RESECTION WITH INTRAOPERATIVE RADIATION (IOR) AND PORTAL CATHETERIZATION (PC) FOR PANCREATIC HEAD CANCER**

S. Takahashi, Y. Ikeda, E. Tamagawa, M. Tomikawa, T. Tsuzuki
M. Kitajima, Y. Ogata
Department of Surgery, Keio University School of Medicine, Tokyo, Japan

Pancreatic cancer is a disease with a poor therapeutic outcome. The frequency of local recurrence and liver metastasis is high even when extended resection (D2) is performed. In this study we assessed the results of performing IOR to prevent local recurrence and PC as a means of preventing liver metastasis.
The subjects of this study were 65 patients with cancer of the head of the pancreas who underwent resection between September 1985 and February 1995. The overall degree of progression was stage I in 7 patients, stage II in 6, stage III in 42, stage IV in 10 (pTNM, UICC). The surgical procedure performed was pancreatoduodenectomy (PD) in 30 patients, PD + portal vein resection in 20 , total pancreatectomy (TP) in 2 , and TP + portal vein resection in 13.
We divided the patients into four groups, group 1 : surgery (D2) alone in 23 patients, group 2 : surgery + IOR in 14, group 3 : surgery + PC in 10, group 4 : surgery + IOR + PC in 18, and assessed the sites of recurrence and survival time retrospectively. Intraoperative radiation consisted of administering 20-30 Gy of electron beam radiation retroperitoneally after performing resection, while portal vein catheterization was used to continuously infuse 5FU, 250 mg/day, for two weeks through the recanalized umbilical vein.

There was no significant difference in the stage distribution in these groups. Investigation of sites of recurrence revealed the following , group 1 : local 87% (20/23), liver 74%(17/23), peritoneal dissemination 4%(1/23) , group 2 : 79%(11/14), 57%(8/14), 29% (4/14) , group 3 : 50%(5/10), 40%(4/10), 0%(0/10) , group 4 : 61%(11/18), 22%(4/18), 33%(6/18). Comparison of the rate of local recurrence in group1 and group 2 , and in group 1 and group 4 , failed to show a significant difference between either pair of groups (P=0.50, P=0.06, respectively), and no preventive effect of intraoperative radiation on local recurrence was detected. Comparison of the rate of liver metastasis in group1 and group 3, failed to show a significant difference (P=0.06), but comparison of group 1 and group 4 revealed a significantly lower liver metastasis rate in group 4 (P=0.001). Mean survival time was 11.8 months in group 1, 9.7 months in group 2, 23.6 months in group 3 and 18.4 months in group 4, and group 4 had significantly longer survival than group 1 and 2. There was a local recurrence preventing effect and a liver metastasis preventing effect in the " surgery (D2) + IOR + PC " group.

### F183

**PANCREATIC ADENOCARCINOMA: OVER 4 DECADES OF SURGICAL EXPERIENCE**

H. Tan, Z. Kanacki, T. Kim, N. Azar, L. Juan, J. Smith, C. Garberoglio
Department of Surgery, Loma Linda University, Loma Linda, California, U.S.A.

A retrospective study of 348 patients (pts) with histologic diagnosis of pancreatic adenocarcinoma from 1950 to 1994 was conducted. The median age and survival are 67.5 yrs and 7.5 mos . Sixty-three pts had curative resections (RSCT), 201 pts had bypass (BYP) and 84 pts had exploratory laparotomy (EXPL). The median and percent 5-yr survival, perioperative mortality and morbidity of pts from the 1950's to the present who had curative resections, bypass and exploratory laparotomy are tabulated below.

| | Median Age(yrs) | Median survival in mos (n) Total | 1950's | 1960's | 1970's | 1980's | 1990's |
|---|---|---|---|---|---|---|---|
| RSCT | 64.3 | 8.7(63) | --- | 4.9(2) | 7.0(20) | 6.6(25) | 17.4(16)† |
| BYP | 70.3 | 4.5(201) | 2.0(14) | 4.4(22) | 4.2(94) | 4.1(49) | 5.5(22) |
| EXPL | 66.7 | 3.3(84) | 0.2(5) | 1.9(5) | 4.0(39) | 3.7(31) | 3.9(4) |

**5-yr survival in % (n)**

| | Total | 1950's | 1960's | 1970's | 1980's | 1990's |
|---|---|---|---|---|---|---|
| RSCT | 9.4(5) | --- | 0.0(0) | 10.0(2) | 8.0(2) | 16.7(1)† |
| BYP | 1.6(3) | 0.0(0) | 4.6(1) | 1.1(1) | 2.0(1) | 0.0(0) |
| EXPL | 0.0(0) | 0.0(0) | 0.0(0) | 0.0(0) | 0.0(0) | 0.0(0) |

**Percent perioperative mortality (n)**

| | Total | 1950's | 1960's | 1970's | 1980's | 1990's |
|---|---|---|---|---|---|---|
| RSCT | 11.1(7) | --- | 0.0(0) | 5.0(1) | 24.0(6) | 0.0(0) |
| BYP | 17.4(35) | 21.4(3) | 13.6(3) | 18.1(17) | 18.4(9) | 13.6(3) |
| EXPL | 23.8(20) | 60.0(3) | 20.0(1) | 20.5(8) | 22.6(7) | 25.0(1) |

**Percent perioperative morbidity* (n)**

| | Total | 1950's | 1960's | 1970's | 1980's | 1990's |
|---|---|---|---|---|---|---|
| RSCT | 49.2(31) | --- | 100.0(2) | 55.0(11) | 52.0(13) | 31.2(5) |
| BYP | 39.3(79) | 35.7(5) | 45.4(10) | 44.7(42) | 34.7(17) | 22.7(5) |
| EXPL | 35.7(30) | 40.0(2) | 0.0(0) | 53.8(21) | 22.6(7) | 0.0(0) |

*Morbidity defined as having at least one of the following: biliary or pancreatic fistula, gastric obstruction or delayed gastric emptying, prolong ileus, hemorrhage requiring more than 2 uPRBCs, sepsis, or pulmonary emboli †$P<0.05$

Our recent experience, as noted above, reveals a 0% perioperative mortality rate in the 1990's of the 16 patients who underwent curative resection with a median survival of 17.4 mos. and a 5-yr survival of 16.7%. This is consistent with previous recent reports that the Whipple operation is safe in the 1990's, with perioperative mortality of less than 1%. The resection of pancreatic cancer does improve these patients' five-year survival.

### F184

**DIVISION OF THE SPHINCTER OF ODDI FOR TREATMENT OF DYSFUNCTION ASSOCIATED WITH RECURRENT PANCREATITIS.**

J Toouli, V Di Francesco, GTP Saccone & J Kollias.
Gastrointestinal Surgical Unit, Department of Surgery, Flinders Medical Centre, Bedford Park, Adelaide, South Australia,Australia.

Increasing evidence suggests that motility disorders of the sphincter of Oddi may lead to episodes of recurrent pancreatitis in a small percentage of patients who are given the diagnosis of idiopathic recurrent pancreatitis. Over the course of 10 years, 35 patients have been identified and treated for this condition. The aim of this study was to assess symptomatic outcome in these patients. Following the exclusion of common causes of pancreatitis the patients underwent sphincter of Oddi manometry. Patients with manometric abnormalities and 3 patients with normal manometry underwent treatment. Initially, patients were treated conservatively and in ten an endoscopic sphincterotomy of the biliary part of the sphincter was performed. Twenty six patients with persistent symptoms underwent total division of the sphincter via an open sphincteroplasty and septectomy. Patients were followed up according to symptoms and classed as cured, mild symptoms or no change. On a median follow up of 24 months (9 to 105), 15 of the 26 patients (58%) were cured. Eight (31%) had only mild symptoms which did not require medical treatment; 3 patients remained unchanged. In the majority of patients having a good clinical outcome, manometry had demonstrated sphincter of Oddi stenosis.
Total sphincter of Oddi division is associated with a good symptomatic outcome in patients with recurrent episodes of pancreatitis and documented sphincter of Oddi stenosis.

### F185

**MANAGEMENT OF NECROTIZING PANCREATITIS (NP) BY REPEATED OPERATIVE NECROSECTOMY: FACTORS AFFECTING MORTALITY**

G. Tsiotos, J. Soreide, and M. Sarr
Department of Surgery, Mayo Clinic, Rochester, MN, USA

Operative treatment of necrotizing pancreatitis at our institution has evolved from open packing to repeated operative necrosectomy/debridement every other day with delayed primary wound closure over drains. We prospectively analyzed our results with operative management of 72 patients (mean age 61 yr; range 20-93) with NP from 1983-1995. **RESULTS**: Overall mortality was 25%. Univariate analysis showed that mortality was increased 1) in patients over age 59 ($p=0.06$), 2) when the preop APACHE II score was > 13 ($p<0.005$), 3) with pancreatic parenchymal versus extrapancreatic necrosis ($p=0.05$), and 4) when the perioperative course was complicated (13 patients) by intraabdominal hemorrhage ($p<0.01$). In contrast, the number of operative debridements (mean = 3, range 0-21) or the development of pancreatic and/or gastrointestinal fistulas (35%; 25 patients) and recurrent intraabdominal abscesses (12 %; 9 patients, 5 of whom were treated percutaneously) were not associated with increased mortality. Multivariate analysis showed that APACHE II score and perioperative hemorrhage maintained significance ($p=0.05$ and 0.03, respectively) as prognostic factors for mortality. **CONCLUSIONS**: NP still carries very significant morbidity and mortality. Planned reoperative necrosectomy with delayed primary closure is associated with a very low incidence of recurrent intraabdominal abscess. Peripancreatic tissue necrosis with preservation of the viability of the pancreatic parenchyma, younger age and especially APACHE-2 score $\leq 13$ and absence of hemorrhage are associated with more favorable outcome.

### F186

**BILIARY AND GASTRIC BYPASS SURGERY IN THE PALLIATION OF PANCREATIC HEAD CARCINOMA; OUTCOME OF 126 PATIENTS.**

**<u>B.A. van Wagensveld</u>, P.P.L.O. Coene, T.M. van Gulik, H. Obertop and D.J. Gouma. Department of Surgery, Academical Medical Center, Amsterdam, the Netherlands.**

**Surgical palliation of unresectable pancreatic head carcinoma is commonly associated with high morbidity and mortality, thus favoring the use of non-surgical (endoscopical) procedures for palliation. More recent reports, however, have shown improved results of surgical palliative treatment. This prompted us to review the results of palliative biliary and gastric bypass surgery in 126 patients who were found to have an unresectable tumor.**
***Patients***: Between 1983 and 1994, 126 patients (m:f = 64:62) with a median age of 64 years (range: 39 to 90 years) underwent a biliary and gastric bypass (n=118), a biliary bypass alone (n=6) or a gastric bypass alone (n=2). Biliary bypass surgery in most cases consisted of Roux-and-Y hepatico-jejunostomy (79%), and gastric bypass of gastro-jejunostomy (GJ). Surgical palliation was undertaken when laparotomy revealed an unresectable tumor (n=44), when endoscopical treatment failed (n=19), when gastric outlet obstruction had occurred (GOO, n=28) or for miscellaneous reasons (n=35). Seventy percent of patients had locally unresectable tumors and 30% had metastatic disease. GJ was performed prophylactically in 92 patients (76.6%). In 89 patients, preoperative, biliary drainage had occurred endoscopically. Median tumor size was 4.3 cm (range: 1.5-12 cm).
***Results***: Postoperative morbidity consisted of local complications (17.5%), general complications (9.5%) and delayed gastric emptying (14.3%). Thirty-days mortality was 0.8% and overall hospital mortality was 2.4%. Median hospital stay was 17 days (range: 5 to 80 days). Eighteen patients (14.3%) were readmitted with mostly a combination of problems: late GOO (n=8), terminal care (n=8), recurrent jaundice (n=7), peritonitis carcinomatosa (n=1) and pain management (n=1). Patients presenting with late GOO (n=8) died within 14-19 days of terminal disease. Median overall postoperative survival was 190 days (range: 14 to 830 days).
***Conclusion***: Roux-and-Y hepatico-jejunostomy combined with a (preventive) gastro-jejunostomy, offers effective palliation for unresectable pancreatic head cancer and can be performed with low mortality (< 2.5%) and acceptable morbidity.

### F187

**THE *IN VITRO* APPLICATION OF CHEMOTHERAPY IN THE TREATMENT OF PANCREATIC CANCER**

<u>S.M. Vickers</u>, M. Rosenfeld, D. Rabens, L.K. Sampson, D.T. Curiel
Departments of Surgery and Biostatistics
University of Alabama at Birmingham, Birmingham, AL

Adenocarcinoma of the pancreas continues to increase in frequency with a current incidence of about 28,000 new cases per year. There has been significant progress in the surgical management of the disease as well as the molecular oncogenesis of pancreatic cancer (p53, *K-ras*). However, little progress has been made in the survival (overall survival < 8% at 2 years). Many strategies for gene therapy have been purposed to achieve selective tumor toxicity and improve prognosis. Molecular chemotherapy is one such approach, which is designed to achieve selective eradication of tumor cells via toxin gene expression. Thus, we have applied this principle to pancreatic cancer in the following study. **<u>Methods:</u>** Pancreatic cell line (BxPc and Panc-1) were maintained under standard cell culture conditions. These cells were transfected at 80-90% confluency with the recombinant adenovirus, AdCMVLacZ (containing the E. Coli β galactosidase gene) and AdCMVHSV-TK containing the HSV-thymidine kinase gene, at 100 pfus/cell at 37° C. AdCMVLacZ infected cells were evaluated by fluorescent activated cell sorting (FACS). AdCMVHSV-TK transfected cells were treated with 20 μm ganciclovir (GCV). Cell viability was determined by trypsin blue and MTT assay. **<u>Results:</u> Both cell lines demonstrated highly efficient transfection (55-60%) with AdCMVLacZ by FACS. AdCMVHSV-TK transfected cells were highly sensitized to the toxic effect of 20 μm GCV x 96 hours. When these cells were reconstituted with non-transfected cells they demonstrated a "bystander effect".** **<u>Conclusion:</u>** Human pancreatic cancer cells can be transfected with a high efficiency with recombinant adenovirus demonstrating marked cytotoxicity to 20 μm GCV as well as having a bystander effect on non-transfected cells. This may provide the basis for novel means of treating pancreatic cancer.

### F188

OVEREXPRESSION OF WILD-TYPE P53 IN CYTOLOGICAL SPECIMENS FROM PANCREATIC JUICE:
IMPLICATIONS OF CHRONIC INFLAMMATION FOR THE PATHOGENESIS OF PANCREATIC CANCER

<u>I.Vogel</u>, H.Maacke, A. Keßler, W. Schmiegel, D. Henne-Bruns, B. Kremer, H.Kalthoff
Department of Surgery, University of Kiel, Kiel, Germany

Pancreatic cancer is characterised by frequent mutations in the p53 gene, leading to p53 overexpression. Detection of p53 overexpression strongly correlates with neoplasia in many cytological specimens.
In order to test the usefullness of p53 assessment in detecting early stages of pancreatic cancer we analysed cytological specimens of pancreatic juice samples for p53 expression.
16 out of 27 cytological specimens from patients with pancreatitis (59%) and 10 out of 15 specimens from patients with pancreatic carcinoma (67%) were positive for p53 expression. The wildtype (wt) p53 specific monoclonal antibody PAb1620 was positive in 11 out of 27 specimens from patients with pancreatitis and 10 out of 13 specimens from patients with pancreatic carcinoma.
The results clearly indicate, that p53 overexpression is observed in cytological specimens of patients with pancreatic cancer as well as patients with pancreatitis, whereas the expression of p53-protein could never be observed in normal pancreatic tissue.
The expression of heat shock protein 70/72 was analysed in parallel:
8 out of 14 (pancreatitis) and 8 out of 13 (pancreatic carcinoma) specimens showed a overexpression of HSP 70/72.
Double immunofluorescence analyses revealed a co-localisation of p53 and HSP 70 in those cytological specimens. In vitro, wt p53 expression was inducible in pancreatic adenocarcinoma cells by TNF-α treatment, followed by apoptotic cell death as revealed by in situ "Terminal Transferase Test". A corresponding result was achieved by analyzing cytological specimens from patients with pancreatitis.
Our findings suggest, that TNF-α (increased levels during inflammation and accompanied by a $O^{-}$- release) is a factor that stimultates p53 expression. This might be one reason for wt p53 overexpression in cytological specimens of pancreatic juice samples from patients with pancreatitis. Chronic inflammation might therefore exhaust the function of wt p 53 as the "guardian of the genome" and/or increase the risk of p53 mutations itself.

### F189

PANCREATIC DUCT OCCLUSION (PDO) WITH FIBRIN SEALANT (FS) FOR THE PROTECTION OF THE PANCREATIC-DIGESTIVE ANASTOMOSIS FOLLOWING PANCREATIC HEAD RESECTION IN PANCREAS CARCINOMA

**<u>Waclawiczek H.W.</u>**
Ist Surg. Dept. and L.-Boltzmann-Institute for Exp./Gastroenterol. Surg., Landeskrankenanstalten Salzburg, A-5020 Salzburg, Austria

The pancreatic-digestive anastomosis is then highly endangered when a normal lienal pancreatic tissue after pancreatic head dissection has to be anastomosed with the jejunum. In the literature many procedures for the permanent as well as the temporary elimination of the exocrine pancreatic secretion are known which in most cases is responsible for the anastomosis-related complications. In animal experiments we could show the efficiency of PDO with FS for the protection of this anastomosis, in the meantime we could gain great experience with this method in clinical trials.
Since 1987 87 patients underwent partial duodenopancreatectomies (Whipple's procedure) due to pancreatic head or peri-ampullary carcinomas. After typical pancreatic head resection the duct of the remaining lienal pancreas was intubated with a thin catheter and then occluded with an average of 2 (± 0.4) ml FS. A high concentration of aprotinin (antifibrinolytic substance) of 20.000 I.U./ml added to the sealant is required to prevent a premature dissolution prior to the 5th postoperative day. Then the remaining pancreas was anastomosed in single-layer fashion.
**Results:** The postoperative lethality rate amounted to 2.4% (n = 2) and was not method-related. In 3 cases bile fistulas occurred, in one a partial liver necrosis and in another one a colonic necrosis, which required relaparotomies. No pancreatic fistulas occurred. A remarkable fact is that postoperatively only 5 of the patients suffered from exocrine and only 3 from endocrine, insulin-dependent insufficiencies.
**Conclusion:** The PDO with FS represents a safe and effective method for the protection of the pancreatic-digestive anastomosis and can therefore be recommended for Whipple's procedure.

### F190

A SPECIFIC DEFICIT IN ACINAR CELL SECRETORY POLE SECOND MESSENGER CALCIUM SIGNALLING DURING ACUTE PANCREATITIS.

J. B. Ward, H. Morris, M. Hashmi, R. Sutton and O. H. Petersen.
Department of Surgery and The Physiological Laboratory, University of Liverpool, UK.

Disruption of stimulus-secretion coupling may be an important event in acute pancreatitis. Cytosolic free ionised calcium ($[Ca^{2+}]_i$) is a key second messenger within the acinar cell, as secretory pole $[Ca^{2+}]_i$ oscillations following secretagogue stimlation initiate normal exocytosis. We have previously shown that physiological oscillatory $[Ca^{2+}]_i$ signals are lost during experimental pancreatitis. This study examined the spatial aspects of $[Ca^{2+}]_i$ signals and the state of intracellular $[Ca^{2+}]_i$ pools during experimental pancreatitis.
Mice received hourly intraperitoneal injections of caerulein (50μg/kg) or saline (paired controls). Pancreata were harvested after injections 1, 3, 5 and 7, and acini isolated by collagenase digestion. Cells were loaded with fura-2 and the pattern of $[Ca^{2+}]_i$ release in response to stimulation with 500nM acetylcholine (ACh) assessed using digital imaging microfluorimetry. The intracellular pools and pattern of $[Ca^{2+}]_i$ extrusion were also assessed by perifusion with the endoplasmic reticulum (ER) $Ca^{2+}$-ATPase inhibitor thapsigargin
Upon ACh stimulation the proportion of experimental cells maintaining a normal polarised increase in $[Ca^{2+}]_i$ progressively diminished: 16 of 17 after 1, 12 of 18 after 3, 6 of 13 after 5,and 3 of 6 after 7 injections respectively ($X^2_{trend}$=7.38, $p$<0.01). A high proportion of control cells maintained a normal signal throughout. Following addition of thapsigargin there was no significant difference between experimental and control cells in the amplitude of $[Ca^{2+}]_i$ increase: between 154±12.9nM (mean ± SE) and 346±34.1nM in experimental cells, and between 243±10.5nM and 382±17.6nM in controls, suggesting that these stores were not depleted. Nor was there any difference in the rate $Ca^{2+}$ extrusion.
These data suggest that during early caerulein hyperstimulation $Ca^{2+}$ reuptake into the ER is preserved but there is a specific disruption of physiological $[Ca^{2+}]_i$ signalling in the secretory pole of the acinar cell. These changes may contribute to the disruption of secretion that occurs in the pathogenesis of acute pancreatitis.

### F191

**FUNCTIONAL RESULTS OF PANCREATOGASTROSTOMY AFTER WHIPPLE PROCEDURE**

**A. Weimann, H. Dralle, J. Klempnauer, H.J. Balks*, K.F. Gratz**, V. Fock, O. Löffge, R. Pichlmayr**

**Klinik für Abdominal- und Transplantationschirurgie,**

**Abt. Endokrinologie* und Nuklearmedizin****

**Medizinische Hochschule Hannover,Hannover,Germany**

**Introduction:**
Pancreatogastrostomy has been recommended as a feasible mode of reconstruction after Whipple procedure in order to reduce anastomotic leakage and postoperative mortality rate. Just a few reports are dealing with functional results after Whipple procedure.
**Patients and Methods**
In our institution from 1988-1994 reconstruction after Whipple operation was performed in 61 cases by pancreatogastrostomy (PG) and in 76 by pancreatojejunostomy (PJ). For follow-up after 14.3±13.3 months 19 patients (PG: 11, 7 male, 4 female, median age: 55, 34-78yrs; PJ: 8, 4 male, 4 female, median age 52, 17-73yrs) underwent assessment: nutritional status (body mass index), resting energy expenditure, endocrine (glucose tolerance, HbA1c, basal serum level of insuline, C-peptid, glucagon and pancreatic polypeptid) and exocrine (serum ß-carotin, pancreolauryl test) pancreatic function, gastric and bowel transit (radionuclid tracer meal), bile flux (cholescintigraphy) as well as Dumping symptoms (Sigstad-score) and quality of life (GIQLI and Spitzer index). In case of surgery for malignancy tumor recurrence was excluded by diagnostic imaging and tumor markers. Statistics: Chi square- and Mann-Witney U-test: p<0.05
**Results:**
Leakage of PG occured in 3/61 (4.9%) and PJ in 4/76 (5.3%) patients (n.s). Functional results did not reveal any statistical differences between PG and PJ. Nutritional status and quality of life were good in both groups. Endocrine insufficiency of the pancreatic remnant could be avoided in most cases: just one PG-patient with insulin-dependant diabetes. However, exocrine insufficiency occured in both groups with a tendency to be more severe after PG. PG patients showed a tendency for less dumping symptoms and higher quality of life.
**Conclusion:**
Pancreatogastrostomy can be considered an adequate functional option for pancreatic anastomosis in patients undergoing Whipple procedure.

### F192

AN ANALYSIS ON FAT DIGESTION IN THE UPPER SMALL INTESTINE AND THE EFFECT OF PANCREATIC ENZYME SUBSTITUTION IN PATIENTS WITH EXOCRINE PANCREATIC INSUFFICIENCY

N. Yamada, T. Nakamura, Y. Tando, Y. Arai, A. Terada, T. Suda
The Third Department of Internal Medicine, University of Hirosaki, Aomori, Japan

The patients with exocrine pancreatic insufficiency of maldigestive state is sometimes severe and causes to death. To clarify the mechanism of fat maldigestion in patients with exocrine pancreatic insufficiency, we analysed upper small intestinal contents and investigated the effect of pancreatic enzyme on maldigestion.
Lipase activities, pH, and micellar lipids in the upper small intestinal contents were studied after intragastric infusion of test meal in 9 Japanese patients with exocrine pancreatic insufficiency and 8 healthy subjects. As the same way, we infused the patients test meal with pancreatic enzyme. The upper small intestinal pH was slightly less in patients with exocrine pancreatic insufficiency than healthy controls. Lipase activities and micellar lipids were significantly decreased in patients with exocrine pancreatic insufficiency compared with those in healthy controls. There was a significant correlation between serum cholesterol and micellar cholesterol concentrations. In micellar lipids, the rate of monoglyceride was decreased and triglyceride was increased.
Lipase activities and micellar lipids, especially monoglyceride were increased after the administration of pancreatic enzyme to the test meal.
The results suggest 1) that in patients with exocrine pancreatic insufficiency there is maldigestive state due to decrease in lipase secretion, 2) that insufficiency of lipase secretion disturbs hydrolysis of triglyceride, prevents micelle formation and leads to decrease in uptake of cholesterol into micellar phase, and 3) that such decrease is reflected in serum cholesterol.

### ORAL PRESENTATIONS

### Topic: BILIARY

### F193

### KLATSKIN TUMOUR: ANALYSIS OF 78 CASES

S. Alfieri, PF. Crucitti, GB Doglietto, G. Costamagna, F. Pacelli, M. Mutignani, C. Carriero, F. Crucitti
Department of Surgery - Catholic University - Rome - Italy

AIM of this study is to report short and long term results of a series of patients with Klatskin tumour (KT) treated with the same homogeneous preoperative investigations and therapeutic approach. MATERIALS AND METHODS: Seventy eight patients with KT were consecutively observed in our unit between November 1987 and July 1995. There were 48 men and 30 women, with a mean age of 66 years. To evaluate origin, level and state of advancement of tumour spread, all patients routinely underwent US, CT-scan and ERCP. Associated major pre-existing disease, advanced age and poor general conditions were never considered as contraindication of endoscopic procedure. According to Bismuth modified classification, tumours were distributed as follow: 16 type I, 24 type II, 36 type III and 2 type IV. With the exception of 4 patients, a preoperative drainage by endoscopic placement of one or more endoprostheses has been always performed. Advanced aged (>75 years), poor general conditions, regional metastases, bilateral involvement of the hepatic ducts beyond the secondary branches, involvement of the main trunk of the hepatic artery, bilateral involvement of the portal vein branches or a combination of vascular involvement to one side of liver with the extensive cholangiographic involvement on the other, were considered contraindications to resection. RESULTS On the basis of the above criteria, 10 patients out of 78 were considered suitable for resection of the tumour. At laparotomy, resectability was confirmed in 8 patients. Local excision of hilar tumour was performed in 5 cases; local excision plus liver resection was performed in other 3 cases: left hepatectomy was required in 2 Type IIIb cases and 1 patient underwent right hepatectomy plus excision of segment 1 because there was a local disease spread suspected to this segment. In all cases all loco-regional lymphatics and areolar tissue was excised. The morbidity and mortality rate was 62% and 12% respectively. Macroscopic and microscopic tumour clearance was obtained in patients. The overall mean survival was 21 months (range 3-73); 4 patients are still alive, disease free, 3,16,21 and 39 months respectively. For the remaining 70 patients considered unresectable for cure stenting by endoscopic mean was the only palliative treatment performed but in 8 cases PTBD was associated to complete endoscopic intrahepatic drainage. Most patients had placement of plastic endoprosthesis; in 41% of patients drainage was obtained with 1 endoprosthesis, 54% of cases required 2 endoprosthesis while only in 4% of cases 3 stents were placed. The morbidity rate and the direct procedure related mortality was of 14% and 1% respectively with a mean survival of 10 months (range1-24). CONCLUSION: from the present study is justifiable to conclude that long term survival and potential cure can be achieved, in selected cases, by radical surgical resection. Endoscopic insertion of single or multiple endoprostheses for patients unsuitable to surgery is a safe procedure and provides good palliation.

### F194

### EFFECTS OF ORAL ERYTHROMYCIN ON GALLBLADDER EMPTYING FOLLOWING TOTAL OR SUBTOTAL GASTRECTOMY FOR CANCER.

*DF Altomare, L LupoS Farese, M Rinaldi, L Caputi, V Memeo*

Istituto di Clinica Chirurgica -Università degli Studi di Bari

Gastric surgery increases the risk of cholelythiasisis due to impaired gallbladder motility secondary to denervation and/or loss of the coordination with gastro-duodenum motility. Erythromycin, a macrolide antibiotic with powerfull prokinetic effect on the GI tract has been shown to promote gallbladder emptying in normal subjects and in those with gallstones.
We investigated the effect of oral Erythromycin on gallbladder emptying in 15 patients (7M, 8F, median age 63y) subjected to total (8) or subtotal (7) gastrectomy for cancer with a median follow-up of 18 months and free of disease. Five healthy subject, were considered as control.
After an overnight fasting, the gallbladder volume (GV) was measured before and 1h after 500mg of Erythromycin and before and 1h after a standardized meal. The GV was calculated ultrasonographically using the 3 Dodds' formula.
A wide variation in GV at fast was seen, particularly in totally gastrectomized patients. After meal, the GV decreased significantly in both groups of patients: from 36.4±21ml to 23.5±22ml (p=0.025) in patients with total gastrectomy and from 43.4±12ml to 19.1±13ml (p=0.018) in patients with subtotal gastrectomy. In the controls the GV decreased from 25.2ml ± to 6.5±2ml. After Erythromycin the GV decreased clearly in controls (from 23.3±10ml to 16±5.6ml), while in patients with total gastrectomy did not change significantly from 41.8±31ml to 38.8±23ml, and in patients with subtotal gastrectomy increased from 31±7ml to 45±17ml (p=0.06).
Two patients in the group of total gastrectomy and one in the group of subtotal gastrectomy had developed gallbladder stones after gastric surgery. Gallbladder motility is only partially impaired by the parasimpathetic denervation after total or subtotal gastric removal for cancer since the gallbladder is still able to empty in response to physiological stimuli. However it does seem to become insensitive to the effects of oral administration of Erythromycin within one hour.

### F195

### EXPERIENCE WITH CHOLEDOCHAL CYSTS

M.AZFAR, P. DHAR, A.SACHDEV, A.CHAUDHARY,
Deptt., of G.I. Surgery, G.B Pant Hospital,
New Delhi-1102, INDIA.

Choledochal cysts are unusual lesions of the biliary tract. Over the last 8 years (1988-95), we treated 64 patients with bile duct cysts, 25(39%) of these were children, 39(61%) adults, 47(73%) females and 17(27%) males. The common presenting symptoms were abdominal pain and jaundice. Adult patients had a higher incidence of cholangitis. Eight (13%) adult patients had undergone a cyst related operation in the past. Sixty two patients underwent an ERCP, and two T-tube cholangiogram. The 38 patients (59%) had type 1 cyst. Twenty five (39%) had type IVa cysts and one(2%) had type IVb cysts. Though type I cyst was commonest in both children and adults,type IVa cysts were seen mainly in adults. Fourteen (22%) patients, all adults, had complications of choleduchal cyst like cholangitis, portal hypertension,cystolithiasias, spontaneous perforation,liver abscess, hemobilia and malignancy. Five patients had emergency surgery beacause of cholangitis and perforation and 59 had planned intervention. Cyst excision and hepaticojejunostomy was done in 61(95%) patients. The choledochal cyst could not be excised in 3 patients each due to the pesence of malignancy, portal hypertension and pericystic adhesions. There was no mortality and no major postoperative complications. Choledochal cysts in adults have potential for complications and in most cases excision of the cyst is possible and gives the best results. Choledochal cysts should be excised as soon as they are detected to prevent the development of complications.

### F196

### INCIDENCE, MANAGEMENT AND OUTCOME OF SILENT COMMON BILE DUCT STONES

C.Bianchi, MD, C.Garberoglio, MD and A.Robles, MD.
Department of Surgery, Loma Linda University Medical Center.
Loma Linda, California

The routine use of intraoperative cholangiogram during laparoscopic surgery is still controversial. Its use in laparoscopic surgery at our institution has been useful in identifying common bile duct stones in patients who had no clinical, laboratory or ultrasonographic evidence of choledocholithiasis.
We have performed this retrospective study in an effort to identify the incidence of "silent common bile duct stones"
**Methods:** We have reviewed seven hundred and twenty patients that underwent laparoscopic cholecystectomy since August 1990 .
The inclusion criteria for "silent common bile duct stones" were 1)the absence of clinical features(jaundice, cholangitis) 2) Normal laboratory (liver function test) 3) and no suggestion of choledocholithiasis (stone or dilation) by ultrasound. Patients having any of the above indicators were recorded in the category "probable common bile duct stones" and excluded for the purpose of this study.
**Results:** .
The total prevalence of common bile duct stones was 11.52 %(83 patients)
Thirty two of these patients were considered to have "silent common bile duct stones" and this represents 38.6% of all patients with choledocholithiasis in this series.
This review has established that three different approaches have been made at the time of finding the stones in the intraoperative cholangiogram. (see the following table)

| | Total pat./ | Approach | Size Stone IOC | Average follow up | ERCP Stone retrival % |
|---|---|---|---|---|---|
| I | 7 | Observation | <4 mm | 13.5 months | |
| II | 8 (*) | Postop. ERCP | <4 mm | 11 months | 37 % |
| | 6 | Postop.ERCP | >4 mm | 11 months | 66 % |
| III | 5 (*) | Flushing | <4 mm | 11.2 months | |
| | 2 | CBD explor. | < 4 mm (impacted) | 34.5 months | |
| | 4 | CBD explor | > 4mm | 10 months | |

Outcome of every group showed no complications or recurrent symptoms.
**Conclusions:**
"Silent common bile duct stones" represent almost 40 % of choledocholithiasis identified in this series.
Some patients(*)(stone < 4 mm, non impacted) may have received unnecessary intervention therefore increasing the total cost of care.
Observation only may be a reasonable approach.
At the present time we are trying to elucidate this hypothesis by a prospective randomized trial.

### F197

### ENDOCRINE RESPONSE TO CHOLECYSTECTOMY COMPARATIVE STUDY BETWEEN STANDARD SURGICAL MANAGEMENT AND VIDEOLAPAROSCOPY

Flora M B Bisinotti, Heller P Chaves, Jose Alves No. , Eduardo Crema
From the Departments of Anesthesiology and Digestive Surgery of Hospital Escola of Faculdade de Medicina do Triângulo Mineiro - Uberaba - MG - Brazil

Cholecystectomy through laparoscopy has become the first choice for procedures of gallbladder and bile duct because of the rapid recovering of patients, which may be associated to less anesthetic-surgical trauma. We compared endocrine response in 30 patients undergoing operative procedures for cholecystectomy using standard technique with subcostal incision (n = 16) and videolaparoscopy (n = 14) through plasmatic dosages of cortisol and adrenocorticotrophic hormone (ACTH) during surgery and at early postoperative period.

Results showed that plasma concentrations of cortisol and ACTH showed no significant differences between the two techniques during surgery and at the first 6 hours postoperatively; only after 12 hours postoperatively there was a trend to significance ($0.05 < P < 0.10$) relating to cortisol, being greater in the standard group.

Analysis of different periods in each group showed significant increasing of cortisol postoperatively compared to initial values for both groups; and ACTH showed significant difference after extubation and 12 hours postextubation for standard group; for videolaparoscopy group it was evident 12 hours postoperatively.

Because these results shows that plasma concentrations of ACTH and cortisol were not significantly different between both groups we concluded that neuroendocrine stimulation induced for both procedures is similar. Some elements are likely to be involved and may be responsible for the rapid recovering of patients undergoing cholecystectomy through videolaparoscopy. Also, pneumoperitoneum and videosurgery were not different from standard procedure relating to the endocrine changes studied.

### F198

### ECONOMIC EVALUATION OF THE LAPAROSCOPIC CHOLECYSTECTOMY.

J.G.Borda;T.Butron;R.Ramos;M.G.Padros;MJ.Castillo;A.G.Carranza; O.G.Villar;ML.Martin;M.Lomas;M.R.Vilariño.
Department of Surgery. Doce de Octubre University Hospital, Madrid, Spain.

Indications of laparoscopic cholecystectomy (LC) are well established and are not anymore object of controversy. The advantages of LC are evident. Our objetive is add the economical aspect as advantage of LC opposite to open cholecystectomy (OC).

MATERIAL AND METHODS. We compare 200 cholecystectomies, 100 LC and 100 OP. Both groups are homogeneous in relation to age, sex, and preoperative test. We analyse the cost of procedure, hospital stay and temporary work incapacity.

RESULTS. The cost of procedure was upper in the LC group than in the OC because the higest cost of disposable instruments in the LC group, LC= 821 U$ or 612 ECUs vs OC= 89 U$ or 67 ECUs. The hospital stay cost was LC= 333 U$ x 1.3 days= 434 U$ or 323 ECUs and OC= 333U$ x 5 days= 1667 U$ or 1242 ECUs. Duration of temporary work incapacity was 14 days in the group LC and 40 days in the OC group. The cost was 228 U$ or 170 UCUs and 650 U$ or 484 ECUs, respectively. Total cost was 1482 U$ or 1104 ECUs for every LC and 2406 U$ or1794 UCUs for every OC. It mean that in the LC group was save 92.304 U$ or 68.798 ECUs if we will compare with the total amount of the OC group.

CONCLUSIONS. LC is more confortable than OC to the patient. Relation cost/benefit was superior in LC group than in OC group, with both lower hospital stay and temporary work incapacity in the first group. If it was used reusable instruments, ecomomical differences would be greater.

### F199

### SURGERY OR ENDOSCOPIC SPHINCTEROTOMY FOR COMMON BILE DUCT STONES ? A MULTICENTER RANDOMIZED TRIAL.

M-J Boudet, B Suc, G Zeitoun, G Fourtanier, F Lacaine, J Escat and French Surgical Research Associations, 8 Avenue des Peupliers, 92270 Bois Colombes.

Surgery and endoscopic sphinctarotomy can both be proposed as definitive treatment for patients with common bile duct stones. However, the choice between the two procedures has not yet been well established. The aim of this randomized trial was to compare surgery and endoscopic sphincterotomy for common bile duct stones, with special reference to operative and short term results.

From 1989 to 1994, 204 patients (64 men and 140 women; mean age 67 ± 18 years; range 25-90) were included in the study. One hundred and seven patients were operated on and 97 underwent endoscopic sphincterotomy. Before treatment, the two groups of patients were not significantly different as regards for mean age, sex ratio, ASA score, previous cholecystectomy, jaundice, cholangitis, or pancreatitis. In the surgery group, stones were found in 72 % of cases, 10.5 % of the patients had a negative exploration of common bile duct. In the spincterotomy group, papilla was seen in all instances. Common bile duct catheterism was possible in 95 %, and sphincterotomy in 82 % of cases. Four post operative deaths were reported: one (1 %) in the surgery group and 3 (3 %) in the sphincterotomy group. Median hospital stay was 16 days and 12 days respectively (ns). Residual stones were diagnosed early after treatment in 8 patients of surgery group (7 %) and in 19 patients of sphincterotomy group (20 %) -$p<0.02$-. Early operation was necessary in 2 patients of surgery group (2 %) and in 18 patients of sphincterotomy group (20 %) -$p<0.0001$-. Although operative mortality was not significantly different between the two procedures, surgery allowed a significant lower reoperation and residual stone rates than endoscopic sphincterotomy. This study showed that surgery should be prefered to endoscopic sphincterotomy, which should be restricted to patients with a significant operative risk.

### F200

### BLACK PIGMENT MICROSTONES FOUND WITHIN THE ROKITANSKY-ASCHOFF SINUSES OF THE GALLBLADDER CAN FORM THE PIGMENTED CENTRE OF MIXED CHOLESTEROL STONES. A STUDY BY SCANNING ELECTRON MICROSCOPY.

A. Cariati. *F. Cetta, °P. Romano, °°F. Conti, °D. Zaccheo.
Institute of Surgical Clinics, University of Genoa and *Siena. °Institute of Human Anatomy and °°Institute of Pediatrics, University of Genoa. Italy.

**Introduction.** We have previously observed that: I) multiple black gallstones (GS) are frequently associated with an increase in number and depth of the Rokitansky-Aschoff (R-A) sinuses of the gallbladder (GB) (1); II) black micronuclei can act as initial nuclei for the formation of multiple cholesterol GS (2). The aim of this study is to analyse by scanning electron microscopy (SEM) black pigment microstones found within R-A sinuses and the pigmented center of the multiple cholesterol GS which were concomitantly pres[illegible] in the same gallbladder looking at microstructural differences or analogies. **Material and Methods.** During the prospective study of 168 consecutive patients who had systematic stone and bile analysis and histologic examination of GB wall, 32 patients were found with adenomyomatosis (ADM), i. e., an increase in number and depth of R-A sinuses, and black stones alone or in association with single cholesterol (n= 7) or multiple mixed (n= 4) stones. In 4 patients with intraparietal black microstones and multiple mixed cholesterol GS, SEM analysis of stones and GB specimens were performed. In addition to x-ray diffractometry, x-ray fluorescence elemental analysis of stones also was performed in 2 of these cases. **Results.** SEM analysis of GB specimens has demonstrated that: 1) both black pigment microstones within the R-A sinuses and mixed cholesterol GS in the main lumen of GB can growth concomitantly in the same GB; 2) black pigment microstones are mostly built up from granules of calcium bilirubinate rather than glassy masses of bilirubinate. SEM analysis of the pigmented center of multiple cholesterol GS showed the presence of a nidus of granules of calcium bilirubinate alone or in association with calcium phosphate in all the observed stones. The presence of calcium phosphate and other calcium salts in the pigmented center of mixed cholesterol stones was also demonstrated by x-ray fluorescence elemental analysis. **Conclusions.** It is suggested that: i) both black microstones and mixed cholesterol GS can growth together in the same GB; ii) microstructures of black pigment microstones and of the pigmented center of mixed cholesterol stones are similar and mainly consist of granules of calcium bilirubinate; iii) multiple cholesterol stones can form by deposition of cholesterol crystals over a black pigment nucleus.

1) F. Cetta, A. Cariati et al. Gastroenterol. 1993; 104: A 353. 2) F. Cetta et al. Gastroenterol. 1993; 104: A356.

### F201

**DIAGNOSTIC YIELD OF ERCP AND INTRAOPERATIVE CHOLANGIOGRAPHY IN 1847 PATIENTS UNDERGOING LAPAROSCOPIC CHOLECYSTECTOMY.**

D.L.Carr-Locke, T.C.K.Tham, J.Vandervoort, R.C.K.Wong, D.R.Lichtenstein, J.Van Dam, F.Ruymann, F.Farraye, D.Brooks. Gastroenterology Division, Brigham & Women's Hospital, Harvard Medical School, Boston, MA.

With the advent of laparoscopic cholecystectomy (LC), the optimal management of common bile duct stones remains controversial. AIM: We report our experience of selective ERCP and intraoperative cholangiography (IOC) in the management of common bile duct stones in a large series of LC from a single center. METHODS : 1847 consecutive LC performed from 1990-1995 were analyzed in terms of ERCP and IOC involvement. A high likelyhood for risk of CBD stones was considered an indication for pre-operative ERCP and was defined as either presence of bilirubin >2mg%, alkaline phosphatase (ALP) >150U/l, present/recent jaundice/pancreatitis or dilated CBD/stone on ultrasound or CT-scan. Selective IOC was performed for intermediate risk based on either bilirubin 1.5-2, ALP 110-150, ALT/AST greater than twice normal or remote history of jaundice/pancreatitis. Post-operative ERCP was performed in patients with suspected retained stones or bile duct injury. RESULTS : Pre-operative ERCP was performed in 143 (7.7%) of patients, was successful in 141 (98.6%) and demonstrated CBD stones in 43 (30%) which were successfully extracted. Of 36 patients with mild gallstone pancreatitis, stones were found only in 6 (17%). Selective IOC was performed in 87 (5%) and stones were found in 4 (5%). Post-operative ERCP was performed in 66 (3.6%). Bile leaks were found in 21 (32%), stones in 20 (30%), ductal injury in 3 (4%), papillary stenosis in 3(4%) and pancreatic duct stricture in 2 (3%). Bile leaks, injuries and stones were all managed endoscopically. Complications were pancreatitis in 6 (4.2%), bleeding in 2 (1.4%), fever in 1 (0.7%) and all settled with conservative treatment. CONCLUSIONS : Even in selected patients considered likely to have CBD stones, the positive diagnostic yield of pre-operative ERCP is low. Mild gallstone pancreatitis is associated with a low incidence of CBD stones. There is a higher incidence of positive findings with post-operative ERCP following selective IOC.

### F202

DIAGNOSTIC VALUE OF MR-CHOLANGIOGRAPHY FOR INVASION TOWARD THE CAUDATE BILE DUCT BRANCH IN CARCINOMA OF THE HEPATIC HILUM

M. Cho, M. Ryu, T. Kinoshita, N. Kawano, M. Konishi, H. Tanizaki
Department of Surgery, National Cancer Center Hospital East, Chiba, Japan.

Objective: In order to identify intrahepatic bile ducts and their anatomical modalities and to comprehend accurately the extent of bile duct cancer in each segmental duct including caudate branches, we attempted to make 3 dimensional images of intrahepatic bile ducts which were reconstructed from MR-cholangiography using maximum intensity projection (MIP).
Materials and Methods: 8 patients with hilar bile duct carcinoma underwent imaging with a MR imager (Magneton H15 Siemens, Erlanger, Germany) and a surface coil. A turbo spin echo pulse sequence (8000 msec / 91 msec TR / TE) was used for data acquisition, with 35 seconds breath holding, 280 mm field of view, and a 120 × 256 matrix. These images sections were processed by using a standard MIP algorithm to obtain views of the entire hepatic biliary tree.
Result: Subsegmental bile ducts and their modalities could be visualized on MR-cholangiography. Some caudate branches which could not be visualized on conventional cholangiography because they were located behind the hepatic hilum could be visualized on MR-cholangiography. Other caudate branches which could not be filled of contrast medium because of the invasion of cancer also could be visualized.
Conclusion: MR-cholangiography provides a noninvasive technique. This is useful for comprehesion of bile duct division and detection of the invasion of cancer because some bile ducts infiltrated by cancer and unfilled of contrast medium can be visualized.

### F203

"KLATSKIN TUMOR: RESECTION OR NOT RESECTION? "

Ciferri E., Bondanza G, Filauro M., Bagarolo C., Gazzaniga G.M.
1st Surg. Dept. S. Martino Hospital Genoa - Italy

Most of updated literature shows an increase in resectability rate of the Klatskin tumour. The problem of this pathologism is represented by the preoperative work-up that is often not able to settle the real staging of the neoplasm and to decide for a surgical or not surgical approach. Between January 1970 and May 1995, 139 cases of Klatskin' tumours were treated in our department. At admittance clinical examination and biochemical data are assumed to assess a risk score in jaundiced patients; then US B-mode liver examination combined with echo-doppler on vascular structures of hepatic pedicle and intrahepatic vessels are performed. Spiral CT completes the diagnostic algorythm. PTC-PTBD are performed in order to define biliary map, to obtain bile ducts decompression and to reduce, if present, obstructive cholangitis. The exact intraoperative staging of the neoplasm was established after a careful histological examinations of the frozen sections of the resected biliary margins to evaluate the presence of microscopical infiltration along the bile duct wall. All patients are staged according Gazzaniga and coll. classification proposed in 1984: Ist stage 18 pts., IInd stage 18 pts., IIIrd stage 4 pts., IVth 99 pts. Among 139 patients, 64 were operated; resectability rate was 46.5%, but radical operations represented only 29.0%. Fourty resections were performed with a curative aim (absence of residual neoplastic tissue), 24 were palliative. Operative mortality rate was 9,5%. Post-operative morbidity rate was 46%. Actuarial five years survival rate for curative surgery was 18,9%. Palliative resections had maximum survival of two years. The remaining 75 cases were treated as follows: decompression of the bile duct with external-internal or external PTBD (46 cases), percutaneous self-blocking endoprosthesis placement (6 cases); surgical palliation included 14 transtumoral stentings and 2 Soupault-Coinaud anastomoses; explorative laparotomy (5 cases); no treatment (2 cases). Surgical treatment, when it is possible, is the therapy of choise because of sensible improvement of survival and quality of life.

### F204

**Sequential treatment of Common Bile Duct and gallbladder Stones by Endoscopic Sphyncterotomy and laparoscopic cholecystectomy: results of a prospective series of 420 patients.**

G. Costamagna, D. Borzomati, V. Perri, M. Mutignani, M. Marciano, F. Crucitti.
Istituto di Clinica Chirurgica Universita' Cattolica del S. Cuore, L.go A. Gemelli 8, 00168 Roma.

The sequential treatment -Endoscopic Sphyncterotomy (ES) and Laparoscopic Cholecystectomy (LC)- for patients with symptomatic gallbladder and Common Bile Duct (CBD) Stones is a widespread and well experienced therapeuthic strategy. From July 1990 through April 1995 indication for a LC was present in 420 symptomatic patients. Ninety-six patients (22.8%) patients underwent a preoperative ERCP because of at least one of the following criteria of suspected CBD Stones:
- clinical history of jaundice and/or acute cholangitis and/or acute biliary pancreatitis;
- increase (at least x2) of total bilirubin, ALT, AST, GGT and alk. phosp.;
- US evidence of CBD or intrahepatic stone/s and/or a CBD size >7mm;

Endoscopic cannulation of the Bile Duct was successful in every patient. Fifty-two patients (i.e. 54% of those undergoing ERCP and 12.4% of the whole group) underwent ES and extraction of stones and/or of biliary sludge was executed in all patient but one. There was no mortality and the morbidity rate was 0.2% (one case of mild acute pancreatitis). All these patients subsequently underwent LC at a mean interval time of 11.2 days (min. 0 days, max 150 days). Intra Operative Cholangiography was never performed. CBD injury, intra/postoperative major morbidity and conversion rate were in 0.2%, 0.7% and 5.8% respectively. More than 95% of all the patients have been followed up by a self-administered postal questionnaire or telephonic interview. 0.4% (1 case of CBD and 1 case of intrahepatic stone) is the residual stone rate after a median follow-up of 21.4 months (range 1-44 months). These results support the safety and the efficacy of the sequential treatment. The high number of negative preoperative ERCP is counterbalanced by a very low rate of residual CBD stone.

### F205

IMMUNE SYSTEM CHANGES AFTER LAPAROSCOPIC AND OPEN CHOLECYSTECTOMY. A PROSPECTIVE AND COMPARATIVE STUDY.

M. Cristaldi, S. Gerlinzani, B. Molteni, H. Kurihara, M. Mezzabotta, M. Rovati, R. Gornati and A.M. Taschieri.
*Departement of Surgery. University of Milan - L. Sacco Hospital, Milan, Italy.*

Up to date it's unclear if laparoscopic surgery can determine less immunosuppressive effects than traditional laparotomic procedures. We determined in a siries of 38 patients affected by symptomatic gallstone disease and operated either by laparoscopic (Group 1) and by traditional open surgery (Group 2), the changes of lymphocyte subpopulations in the postoperative, compared to preoperative period, on different time point up to day 30 after surgery. We collected 15 ml of venous blood from all patients in both group 1 and 2, on day -1 (1 day before surgery) and on postoperative day 1,7,15 and 30. A single blood sample from a control group (Group 3) formed by 56 healty volunteers was obtained. In the patients submitted to open cholecystectomy we observed a significant fall in total lymphocyte count in postoperative day 1. Basal levels of lymphocyte subpopulations did not show any statistical significant difference (Wilcoxon test) between study and control groups (results with P<0,01 are considered significant). No differences were found in preoperative lymphocyte cell count between the two groups submitted to cholecystectomy. Pan T cell (CD3) showed a marked statistically significant reduction throughout the observation period. The count of helper (CD4), suppressor (CD8) and NK (CD16) T cells was reduced on postoperative day 1, NK cell (CD16) count remaining low until posfoperative day 30. B lymphocytes group showed no postoperative reduction. In patients submitted to laparoscopic cholecystectomy a significant postoperative fall of total lymphocytic count, CD3, CD4 and CD8 subpopulations was observed on day 1 only. No reduction of CD16 and CD19 subpopulations was noted. A comparative statistical analysis between lymphocyte subpopulations inthe two groups was carried out: in the open cholecystectomy group as compared to laparoscopic group, CD3, CD4, CD8 and CD16 lymphocyte subpopulations showed a marked reduction on different time points. In particular, statistically significant differences were found in CD3 levels from postoperative day 30, in CD4 from day 1 through day 7 and in CD8 and CD16 only on day 1.

### F206

**MORPHOLOGICAL FEATURES INFLUENCING PROGNOSIS OF PATIENTS WITH BILE DUCT CARCINOMA.**

A D'ERRICO, A. PRINCIPE, M.L. LUGARESI, E. BERTI, I. BICCHIERRI, M.C. GALLO, A. CAVALLARI
Department of Surgical Pathology and II Department of Surgery, Bologna University, Italy.

Many discrepancies are reported in the literature about the clinico-pathologic factors that can influence survival of patients with bile duct carcinoma.
**Material and Methods:** We performed a revision of 37 bile duct carcinomas surgically resected in the II Department of Surgery, Bologna University, from 1982. The 37 carcinomas were classified as middle (5 cases, 13,5%), lower (11 cases, 29,7%) and hilar (21 cases, 56,7%) bile duct carcinomas. In each case we evaluated the following clinico-pathologic variables: age, sex, location of primary tumor, serosal invasion, peritoneal dissemination, hepatic metastasis, lymph-node metastasis, pancreatic invasion, duodenal invasion, microscopic vessels involvement, perineural invasion, resected proximal and distal margin involvement, histologic type, depth of cancer invasion and survival.
**Results and follow-up:** *Lower bile duct carcinomas:* All but two pts died for neoplastic progression (mean survival = 19,6 months), two pts are alive and free of disease ( ms= 90 months). *Middle bile duct carcinomas:* 3 pts died for neoplastic disease (ms= 15,3 months) and 2 pts are alive (ms= 96 months). *Hilar bile duct carcinomas:* 2 pts died after surgery, 10 pts died for neoplastic disease (ms= 11,8 months), 4 pts are alive at 13 months, 2 pts died at 51,5 months (ms), and 3 pts are free of disease at 56 months (ms). The main clinico-pathologic factors that seem to correlate with prognosis are location of primary tumor, size of tumor, depth of cancer invasion, histologic type, lymphatic or perineural invasion, lymph-node metastasis, hepatic or duodenal diffusion, and pancreatic metastasis.
**Conclusions:** On the basis of this retrospective revision morphological features seem to be closely related to the clinical behaviour of bile duct carcinoma, and they can be evaluated only by the mean of an accurate intraoperative staging.

### F207

BILIARY DUCT INJURIES SECONDARY TO LAPAROSCOPIC CHOLECYSTECTOMY: A NATIONAL SURVEY

E. de Santibañes, S. Perera, R. Sendin, J. Grondona.
**HPB Surgery Section, General Surgery Service, Hospital Italiano, Buenos Aires, ARGENTINA**

**A national survey of the surgical biliary duct injuries secondary to video laparoscopic cholecystectomy was performed in the Argentine Republic. A total of 116 cases were reported. Fifty one surgeons were surveyed, and 20 of then sended the casuistic of 12 hospitalary centers and 31 the own experience. Only the 50% of the surgeons who injured the biliary duct had training in laparoscopic surgery. The 77% of the preoperative diagnosis were cholelithiasis, and the lesion occurred in the 98% of the cases during a simple cholecystectomy. Forty four percent of the injuries were advised and 56% passed innoted. Only in the 12% of the cases the intraoperative colangiography was performed, and the intent of immediate repair were in 18 of the 116 patients (5,51%). The main surgical procedures performed choledoco-choledoco anastomosis with T-tube (19.82%), double hepaticoyeyunostomy (17,24%) and triple hepaticoyeyunostomy (4,3%). The type of lesion on the Bismuth classification were: I= 21,51%; II=26,72%;III=14,65%;IV=23,27%;V=5,17% and unknown=8,62%. It is mentioned that in Argentinean bibliography it is low the rate of injuries of the biliary duct during laparoscopic cholecystectomy. We conclude that the knowledge of the mechanisms of biliary duct injuries is the best way to prevent then. The intraoperative cholangiography help the surgeon to identify the biliary anomalies, but no substitute the carefully dissection and the delineation of the anatomy. Further not prevent the lesion but advise about it, avoid serious injuries and permit the intent of the immediate repair**

### F208

THE FEASIBILITY OF LAPAROSCOPIC CHOLECYSTECTOMY IN PATIENTS WITH PREVIOUS ABDOMINAL SURGERY

Dr. J. Diez *
Dr. R. Delbene **
Dr. A. Ferreres*
* Department of Surgery - University Hospital - B.A.- Argentina.
** Dragones Clinic.

A retrospective study was carried in 1500 patients submitted to elective laparoscopic cholecystectomy to ascertain its feasibility in patients with previous abdominal surgery.
In 411 patients (27,4%) previous infraumbilical intraperitoneal surgery had been performed, and 106 of them (7,06%) had 2 or more surgeries.
The most common was a cesarean operation. In this group the first trocar was placed in the umbilicus.
Twenty five patients (1,66%) had previous supraumbilical intraperitoneal operations (colonic resection, hidatic liver cysts, gastrectomies, etc.). Three of them had been operated 3 times. In this group the first trocar and pneumoperitoneum were performed by open laparoscopy.
In 2 patients a Marlex mesh were present due previous surgery for supraumbilical hernias.
Previous infraumbilical intraperitoneal surgery offered no inconvinience for performing laparoscopic cholecystectomy, even in patients with several operations. No morbidity was due to the Veres needle or trocars.
In the 25 patients with infraumbilical intraperitoneal operations laparoscopic cholecystectomy was performed in 22.
In 3, adhesions to the abdominal wall, liver, etc. prevented the dissection of the gallbladder and the patients were converted.
The Marlex mesh in 2 patients because of adhesions to abdominal organs made necesary to convert them to open surgery.
Conclusion: Previous intraperitoneal infralumbilical operations was not in our patients contraindications to laparoscopic cholecystectomy and no morbidity was related to them.
Supraumbilical intraperitoneal surgery is no more a contraindication for laparoscopic cholecystectomy. It should be performed by open laparoscopy and carefull disection. In some patients, specially those with reoperations for peritonitis, or hemoperitoneum, adhesions may prevent its performance, intraperitoneal Marlex meshes, because of the adhesions they provoque may be a contraindication for laparoscopic cholecystectomy.

### F209

### ROLE OF EXTENDED LYMPH NODE DISSECTION FOR ADVANCED GALLBLADDER CANCER

I. Endo, S. Togo, A. Nakano, H. Sekido, K. Goh, A. Takimoto, T. Takahashi, , H. Shimada
Second Dept. of Surgery, Yokohama City Univ. School of Med. Yokohama, Japan

The aim of this study is to elucidate the role of extended lymph node dissection for advanced gallbladder cancer. Forty-one consecutive patients with advanced gallbladder cancer(tumor extended more than subserosal layer) were underwent extended lymph node dissection combined with hepatectomy and resection of bile duct. The range of lymph node dissection covered N1, N2 of UICC and para-aortic region. Lymph node metastasis was found in 28 out of 41 patients(%). Metastatic rate of each regional lymph node were 29%(cystic node), 45.2%(pericholedochal node), 45.2% (posterosuperior pancreaticoduodenal node), 25.8%(retroportal node), 19.4% (around the common hepatic artery). 8 out of 28 patients underwent para-aortic lymph node dissection. Jumping metastasis(means N1 negative and N2 positive) was observed in 2 cases. Cumulative survival rates of patients underwent curative resection were significantly higher than non-curative cases. Overall survival rates at 1, 3 and 5 years were 87.1, 62.4 and 32.8% respectively. There was a statistical significant defferrence between the survival rates of patients without metastasis and with N2 metastasis. There was no case which recurred in para-aortic region in N1 positive patients. On the other hand 7 out of 11 patients with N2 and para-aortic nodes metastasis recurred in para-aortic region. Recurrence sites were out of the area whitch dissected previously such as retrocaval area and hilus of left kidney. Six out of 11 patients with N2 and para-aortic nodes metastases survived more than 2 years. We conclude that extended lymph node dissection is benefitial for precise estimation of tumor extention and prolonged survival periods.

### F210

### MR Cholangiopancreatography (MRCP) in Failed or Incomplete ERCP

Joseph T. Ferrucci, m.D.
Department of Radiology
Boston University Medical Center
Boston, MA 02118

**Purpose:** To assess the value of MR cholangiopancreatography (MRCP) in patients in whom ERCP fails to cannulate the biliary or pancreatic ducts or does not provide complete delineation of ductal abnormalities.
**Methods:** MRCP using a respiratory-triggered multi-slab three-dimensional fast spin echo sequence was performed in 37 consecutive patients referred during an 18 month period because of an unsuccessful ERCP (n=20), presence of post-surgical biliary-enteric anatomy (n=10) or evidence of complete pancreatic duct obstruction on ERCP (n=7). MR examinations were acquired in the coronal plane on a 1.5T system (Philips ACS Gyroscan II) using the following parameters: TR 2500-5000 msec, TE 240 msec, field of view 240 mm, 1 or 2 signal averages, matrix 186 x 256, echo train length 31, slice thickness 2 mm. Eight or 10 slabs were obtained as part of the multi-slab acquisition with 5 or 6 slices per slab (total slices 40 to 50). Subsequent course and impact on clinical management was determined.
**Results:** Satisfactory MRCP studies were obtained in 100% of patients. Findings on MRCP were: normal biliary and pancreatic ducts (n=11), bile duct dilatation without stones (n=12), changes consistent with chronic pancreatitis (n=13), choledocholithiasis (n=4), pancreatic tumor (n=2), isolated pancreatic duct stricture (n=1) and biliary stricture (n=1). Multiple findings were present in 7 patients. Based on MRCP results, patients subsequently underwent laparotomy (n=11), therapeutic ERCP with precut papillotomy (n=3), therapeutic PTC (n=2), diagnostic PTC (n=1) or ultrasound-guided biopsy (n=1). The 11 patients with normal findings on MRCP required no intervention. The remaining 8 patients had abnormalities detected on MRCP but were followed clinically.
**Conclusion:** MRCP plays an important role in the management of patients when ERCP is unsuccessful or incomplete and in patients in whom technical difficulties can be anticipated. Failed ERCP represents one of the main clinical indications for performing MRCP.

### F211

### GALLBALDDER MOTILITY AND GALLSTONE FORMATION DURING RAPID WEIGHT LOSS INDUCED BY VERY LOW CALORIE DIETS: A PRESENT CONFIRMATION OF AN OLD CLINICAL OBSERVATION.

D. Festi., M. Orsini*, A. Colecchia*, S. Sottili*, A. Sangermano*, G. Mazzella *, N. Villanova*, ML. Petroni **, S. Pavesi **, E. Roda *.
Istituto di Fisiopatologia Medica, Universita' G. D'Annunzio , Chieti; * Cattedra di Gastroenterologia, Universita' di Bologna; ** Istituto Scientifico Ospedale S. Giuseppe, Centro Auxologico Italiano, Piancavallo, Verbania; Italy.

Very low calorie diets (VLCD's) have increasingly been used in the treatment of morbid obesity; however, an increased risk of gallstone formation has been reported during rapid weight loss. Impaired gallbladder motility is considered an important pathogenetic factor for gallstone development. Since diet composition modulates gallbladder contraction and most VLCD's are characterized by a low fat content, this study was aimed at evaluating whether VLCD's with low and higher fat content might influence gallbladder motility and as a consequence modify the risk of gallstone formation during weight loss. Sixteen obese gallstone-free subjects (4 males, 12 females, age: 35.5±2.8 yrs, BMI: 41.3±1.4 $kg/m^2$, mean±SE) were studied. Gallbladder motility was evaluated by ultrasonographic technique (US), on three different days, in response to a **liquid test meal** (375 kcal, 12 g fats) and to two different VLCDs administred as follow: **diet A** (515 kcal with 3 g fats; breakfast 123 kcal, 1.6 g fats; lunch: 242 kcal, 1.1 g fats; dinner: 150 kcal, 0.3 g fats); **diet B** (565 kcal with 12.4 g fats; breakfast: 123 kcal, 1.6 g fats; lunch: 267 kcal, 5.8 g fats; dinner: 175 kcal, 5 g fats). Diet A was administred to 8 subjects for 45 days and diet B to the remaining 8 subjects, always for 45 days. At the end of the diet period, the appearance of gallstones was investigated by US and a gallbladder motility study repeated as at baseline. In the statistical analysis Mann–Whitney test (independent samples) and Wilcoxon test (paired samples) were used to compare data. Results are reported in the following table:

| Diet | Time | BMI | Diet A | | | | | | Diet B | | | | | | Test meal | | p | |
|---|---|---|---|---|---|---|---|---|---|---|---|---|---|---|---|---|---|---|
| | | | Breakfast | | Lunch | | Dinner | | Breakfast | | Lunch | | Dinner | | | | %A vs %B | %B vs %TM |
| | | | BV | % | BV | % | BV | % | BV | % | BV | % | BV | % | BV | % | | |
| A | 0 | 40.3 ± 1.5 | 35.7 ± 4.7 | 27.6 ± 2.5 | 34.6 ± 4.5 | 41.2 ± 5.5 | 32.3 ± 4.4 | 43.4 ± 3.3 | 38.5 ± 5.4 | 28.9 ± 2.5 | 35.3 ± 6.0 | 61.1 ± 5.1 | 33.o ± 5.3 | 64.0 ± 3.8 | 31.6 ± 5.3 | 56.6 ± 4.2 | <0.04 | <0.05 |
| | 45 | 34.8 ± 1.2 | 26.0 ± 4.0 | 28.7 ± 2.1 | 25.8 ± 3.7 | 53.1 ± 4.8 | 19.2 ± 2.7 | 51.0 ± 2.8 | 24.4 ± 4.7 | 21.9 ± 4.0 | 25.4 ± 3.7 | 72.5 ± 4.5 | 21.9 ± 2.7 | 75.9 ± 3.7 | 26.9 ± 4.8 | 71.1 ± 3.5 | <0.05 | <0.05 |
| B | 0 | 42.5 ± 2.0 | 35.4 ± 5.7 | 22.4 ± 2.0 | 35.8 ± 5.8 | 44.6 ± 2.7 | 33.1 ± 5.6 | 39.3 ± 2.8 | 34.6 ± 4.9 | 24.3 ± 1.6 | 33.9 ± 4.9 | 61.9 ± 4.1 | 29.0 ± 5.7 | 60.6 ± 4.1 | 33.7 ± 4.6 | 56.4 ± 4.5 | <0.03 | <0.05 |
| | 45 | 37.4 ± 1.6 | 23.4 ± 5.4 | 27.0 ± 1.7 | 23.3 ± 5.5 | 48.4 ± 2.5 | 22.0 ± 5.3 | 44.9 ± 2.3 | 22.9 ± 5.3 | 27.7 ± 3.0 | 22.4 ± 5.3 | 68.0 ± 4.0 | 21.3 ± 5.2 | 69.1 ± 2.5 | 23.7 ± 5.3 | 72.4 ± 3.2 | <0.02 | <0.02 |

In both groups, BMI significantly decreased after VLCD's. Three out of the 8 subjects (37.5%) who followed diet A, but none with diet B, developed asymptomatic gallstones. Diet A induced a significantly lower gallbladder emptying in respect to diet B both before and after the diet period. These results confirm the role of diet characteristics in influencing gallbladder motility and suggest that higher fat content of a VLCD could prevent gallstone formation during weight loss.

### F212

### RESULTS OF 210 CONSECUTIVE LAPAROSCOPIC BILE DUCT EXPLORATIONS

**GA Fielding, NA O'Rourke, IS Bailey, M Rhodes, LK Nathanson.**
**Royal Brisbane Hospital and Wesley Hospital, Brisbane, Australia.**

**Experience with laparoscopic bile duct exploration is slowly increasing. We report the results of 210 consecutive laparoscopic bile duct explorations performed since August 1991. Utilising a variety of techniques 185/210 (88%) patients bile duct was cleared laparoscopically. Eleven patients were converted to an open procedure, 14 had stones cleared by post-operative ERCP and 7 had normal ERCP for post-operative pain. One hundred and thirty-two patients had transcystic exploration and 79 patients had choledochotomy. One patient died during the post-operative period. Eight of 46 (15%) of laparoscopic choledochotomies closed with T tubes had T tube related morbidity. One patient has had a bile duct stricture post-operatively requiring reconstruction. Laparoscopic bile duct exploration is technically satisfactory and successful in 88% of patients with 0.5% mortality and relatively little morbidity.**

F213

MOLECULAR BIOLOGICAL EXAMINATION IN PANCREATICOBILIARY MALJUNCTION

Funabiki, T., Matsubara, T., Sasayama, Y., Hori, H., Hasegawa, S., Kamei, K., Sakurai, Y., Marugami, Y., Ochiai, M.
Department of Surgery, Fujita Health University School of Medicine, Toyoake Aichi 470-11, JAPAN

Pancreaticobiliary maljunction (PBM) that was initially reported as a disease accompanied by choledochal dilatation has recently drawn an attention because it appears to be frequently associated with biliary carcinoma. However, there are little studies of carcinogenesis in PBM using molecular biological techniques.
In order to obtain an insight into the molecular biological aspect of the carcinogenesis in PBM, we investigated point mutation of K-*ras* oncogene and the expression of p53 protein in cancerous and noncancerous biliary epithelia of patients with PBM.
MATERIALS AND METHODS: Twenty cases of patients with PBM (5 cases associated with biliary carcinoma and 15 cases not associated with carcinoma) as well as 18 cases with gallbladder carcinoma without PBM and 7 cases with slight cholecystitis without PBM as control were examined. Point mutations of K-*ras* oncogene were examined in cancerous tissues and noncancerous biliary epithelium, hyperplasia, metaplasia, and inflammation, that were separately scraped out from formalin-fixed paraffin-embedded histological sections. p53 protein in these tissues were immunohistochemically stained using anti-p53 monoclocIonal antibody.
RESULTS: Incidences of K-*ras* point mutation were 80% in biliary carcinoma with PBM, 58% in hyperplasia/metaplasia and 38% in inflammation in PBM, while 53% in carcinoma without PBM and 0% in control gallbladder epithelium. Positive rates of p53 protein were 100% in carcinoma with PBM, 18% in non-cacerous epithelium with PBM, while 92% in carcinoma without PBM and 0% in control.
CONCLUSION: Biliary epithelium in cases with PBM may be in precancerous state, which should be considered for the treatment of PBM.

F214

LAPAROSCOPIC HEPATICOGASTROSTOMY FOR MALIGNANT BILIARY OBSTRUCTION.

M. Gagner, MD, G. Soulez, MD, E. Thérasse, MD, E. Deslandres, MD, A. Pomp, MD. The Cleveland Clinic Foundation, Cleveland, Ohio and Hotel-Dieu de Montreal, Montreal, Quebec.

The purpose of this study was to evaluate the potential role and benefit of laparoscopic biliary decompression in patients with malignant biliary obstruction.

Since 1/92, 19 patients (14 F, 5 M) with a mean age of 65 (range 43-91), underwent laparoscopic hepaticogastrostomy. After transhepatic catheterization of a segment II or III bile duct, the left lobe of the liver and the lesser curvature of the stomach were perforated under fluoroscopic and laparoscopic guidance using three trocars. Anastomosis between the biliary tree and the stomach was maintained with a gastrostomy tube placed across the tract. After 2 weeks, the tube was removed and patency of the tract was preserved with a metallic stent.

Two-thirds of the patients had a hilar level of obstruction, and 65% of patients had an unresectable cholangiocarcinoma or pancreatic adenocarcinoma during laparoscopic staging. One-fourth of patients palliated also had biliary obstruction due to metastatic, colon adenocarcinoma, or gastric adenocarcinoma. The total bilirubin fell from 271 to 32 ($p<0.001$) in less than four weeks. The mean hospital stay was 17 days. After a follow-up period of 47 months, the mean survival was 7-months with 35% of patients surviving more than 12 months. Two patients died of septicemia and pneumonia in the hospital. Early complications were cholangitis (3), subcapsular hematoma (2), and gastric outlet obstruction (1). No reintervention or endoscopic procedures had to be performed. The recurrence of jaundice was only seen in two patients due to liver failure.

A high level of malignant biliary obstruction can be palliated effectively with the laparoscopic method with a relatively low morbidity and mortality rate in this selected group of patients.

F215

CHANGES OF PMN-ELASTASE AND C-REAKTIVE PROTEIN FOLLOWING TRADITIONAL AND LAPAROSCOPIC CHOLECYSTECTOMY

I. Gál, L. Lantos*, E. Röth*

Dept. of Surgery, Bugát Pál Hosp. Gyöngyös, Dept. of Experimental Surgery,Univ. Med. School of Pécs* Hungary

The magnitude of metabolic response to injury has been shown to be proportional to the degree of surgical trauma. For this reason many investigatior have henceforth tried to find ways of reducing the metabolic response to surgery. Laparoscopic surgery is a minimal invasive procedure becoming the operation of choice in many field of surgery. In this study of patients undergoing open cholecystectomy (OC) and laparoscopic cholecystectomy (LC) we aimed to determine, whether or not there are any indications of postoperative inflammatory reaction by measuring serum concentration of C-reaktive protein (CRP) and polymorphonuclear leukocytes (PMN) - elastase as the most sensitive biochemical markers of the granulocyta stimulation. An unselected number of patients (pts) were investigated and divided into two groups. Group I. contained 15 pts operated by OC; Group II. involved 15 pts with LC. The indication for surgery was chronic cholecystitis in all cases. Anaesthetic and operative procedures were standardised in both groups. Venous blood samples were taken 3 hours before surgical intervention and every day for 4 days following the operation. CRP level was measured by immonological assay (CRP-latex aggutination slide test), the PMN-elastase was measured by a heterogeneous enzyme immunoassay (Reagent kit, MERCK) to specific determine its complex formatione with $\alpha$1-proteinase inhibitor.The postoperative CRP concentration rose markedly following OC, and reached a peak of 84,1±14,32 ng/ml on the second day. This elevation was highly significant ($p<0{,}001$), while in the LC group only a moderate increase to 52,7±17,70 ng/l was shown, which was not statistically significant. The level of CRP in the OC group remained higher during the whole observation period, while in the LC group it decreased to the starting value 19±4.93 ng/l by the 4th day. Our results showed a significant PMN-elastase elevation in both groups on the first postoperative day (135,1±9,1 ng/l, $p<0{,}001$; 150,5±24,8 ng/l $p<0{,}05$ respectively). However there was a marked difference between the two groups by the 3rd postoperative day. While the value of PMN-elastase in the LC group decreased considerably (116,5±20,9 ng/l) it maintained a high level in OC group (164,6±18,9 ng/l; $p<0{,}01$). Following the 4th postoperative day we still found an elevated enzyme level in the OC group (152,8±9,3 ng/l; $p<0{,}01$), while in the LC group a continuous fall towards the baseline value was noted (87,2±10,5 ng/l).These results provide us with biochemical evidence supporting the clinical observation that LC is far less traumatic to the patients than OC.

F216

EXPERIENCE OF THE 100 OPERATIONS WITHOUT STENTING FOR BENIGN (BISMUTH 3-5 TYPE) HEPATIC DUCT STRICTURE

E.Galperin, N.Kuzovlev, A.Chevokin
Department of Hepatic Surgery, Moscow Medical Academy,Russia.

100 surgical repairs without stenting were performed for benign bile duct strictures (Bismuth 3-5 types) from 1987 through 1994. There were 78 women and 22 men with ages ranging from 23 to 69 years. The most common cause of the stricture (85,7% of the patients) was the iatrogenic trauma during cholecystectomy or resection of the stomach. Many of our patients had undergone previously 3, 4 and 5 operations on the bile ducts, and suffered from long-term jaundice and cholangitis (69% of the patients). 25 patients had the stricture below the bifurcation of the hepatic duct( less then 1 cm), 49 patients had the stricture of the confluence, 19 patients had biductal stricture, and 7 patients had monoductal stricture. Performing high mucosa-to-mucosa biliary enteral anastomosis, we tried to free the hepatic ducts above the stricture. In our report we shall present some variation of the "platform of the duct branches" for biliary enteral anastomosis. No fatal outcome was noted and the 17 complications were: subphrenic abscess - 1 case, temporary biliary fistulae - 6 cases, intraabdominal bleeding - 3 cases, gastrointestinal bleeding - 6 cases, intestinal ileus - 1 case. Two patients had been operated over the long-term follow-up period ( from 6 months to 9 years): recurrent stricture - 1 case, intrahepatic cholangiolitias -1 case. Our experience shows, that in benign biliary (type 3 - 5) stricture thorough dissection of the bile ducts above the stricture permits precise anastomosis to be constructed without stenting.

F217

### BILIARY TRACT INJURY (BTI) DURING LAPAROSCOPIC CHOLECYSTECTOMY (LC) : RESULTS OF A NATIONAL SURVEY.

J.F. Gigot and the Belgian Group for Endoscopic Surgery (BGES)

Department of Digestive Surgery, Louvain Medical School

A retrospective anonymous survey was conducted in Belgium in a multicentric group of general surgeons about 9959 LC. The incidence of BTI was 0.5 %. The prevalence is 1.3 % below 50 cases of experience compared to 0.35 % ($p < 0.0001$) over 50 cases of experience. 36% of these BTI have been performed by a surgeon with more than 100 cases of experience. Predisposing local risk factors were present in 61 %. Severe BTI occured in 42 %. Intraoperative detection of BTI occured in 44 %, mainly related to the performance of intraoperative cholangiography. In patients with biliary peritonitis, the overall mortality and late biliary stenosis were 19 % and 38 % respectively, compared to 4 % and 12 % in patients with intraoperative detection of BTI. Hospital mortality was related to the occurrence of biliary peritonitis, while late biliary stenosis was related to the occurrence of postoperative biliary complications after initial biliary repair.
In conclusion, mortality and morbidity of BTI during LC are high. Intraoperative detection - mainly with the use of IOC - is an essential factor to improve outcome.

F218

### MODIFIED MINI-CHOLECYSTECTOMY: A MINIMALLY INVASIVE PROCEDURE, EXPERIENCE WITH 250 CONSECUTIVE CASES

I. Hamdi, A. Khamag, J.Faitori
Department of Surgery, Arab Medical University, Benghazi, Libya

Minimally invasive surgery is being increasingly employed and extended to various procedures, it reduces hospital stay and shorten recovery interval, with excellent cosmetic results. Short incisions tend to be associated with less post-operative pain. Tissue destruction is minimised and the risk of wound complications is probably diminished as a result.

We report the results of modified mini-cholecystectomy on 250 patients (212 women, 38 men). The mean age of the patients was 39 years (range 16-72 years), while the mean weight was 80.2 kg (range 61-135 kg). Eighty percent of patients were overweight (5-98% in excess of standard chart based on height and weight). The incisions used were 2 to 4 cm long, and the procedure was carried out employing selected laparoscopic instruments. Forty-two patients had mucocele and 17 had empymatous gall bladders. The incision had to be extended in 22 patients due to obscured anatomy (10 patients) or for unanticipated exploration of the common duct (12 patients). Nasogastric tubes were not employed, peritoneal drainage was instituted for cases with infected gall bladder. All patients were allowed oral intake after 6 hours from operation. The mean period of hospital stay was 1.9 days (range 1-5 days). The operative time ranged for 20-82 minutes, generally tending to get shorter towards the end of the study period, presumably a reflection of the learning-curve effect. No major postoperative complications were encountered in any of our patients during the follow-up period (3-31) months.

We conclude that modified mini-cholecystectomy is a simple and safe procedure. In our estimate the operation is applicable to over 95% of patients scheduled for elective cholecystectomy.

F219

### CLINICAL REVIEW ON PANCREATICOBILIARY MALJUNCTION - ADULTHOOD VS CHILDHOOD -

H. Hara,K. Okajima,H. Isozaki,S. Morita,T. Ishibashi,S. Sako, K. Fujii
Department of Surgery,Osaka Medica College,JAPAN

**Purpose:** The present study was designed to review the clinicopathological items of pancreaticobiliary maljunction in adults and children. **Patients and methods :** In sixty four patients (42 patients of adults, 22 patients of children), who were surgically treated during the past 16 years in the department of surgery, Osaka Medical College, the following items were reviewed: 1) morphological classification, 2) operative method, 3) postoperative complications, 4) cell kinetics (proliferating cell nuclear antigen (PCNA) of the epithelium of bile duct and gallbladder). **Results:** [Morphological classification ( TOTANI's Classification) of congenital biliary dilatation] Type Ia was found in 5 patients, type Ib in one, type Ic in 10, type IV-A in 18, type IV-B in one, and no dilatation in 7 of 42 adult patients and type Ia in 5, type Ic in 11, and type IV-A in 6 in 22 children.Cancer was observed in 9 patients in adults (6 patients of gallbladder cancer, 3 patients of bile duct cancer) .**Operative method:** In the cancer-free adults, biliary reconstruction after resection of extrahepatic bile duct was performed by 1)choledochoduodenostomy in 8, 2) Roux-en-Y's fashion in 16, 3) jejunal interposition in 8, and 4) panceraticoduodenectomy in 1. In children, choledochoduodenostomy was performed in 17, Roux-en-Y's fashion in 3, jejunal interposition in 2. In patients with gallbladder cancer, 2 patients underwent cholecystectomy with lymph node dissection (LN), 3 patients underwent cholecystectomy with LN with partial hepatectomy, and the other one case was nonresectable. In patients with bile duct cancer, one underwent pancreaticoduodenectomy with portal vein resection and LN, one underwent extrahepatic bile duct resection with LN, one was nonresectable. Five of 6 patients with gallbladder cancer are alive, but all the three patients with bile duct cancer have already died. **Postoperative complications:** Postoperative cholangitis was observed in 7 patients (6 patients in adults, one patient in child). In 6 adult patients with postoperative cholangitis, 4 patients were mildly ill, but, 2 were severely ill, requiring surgical treatment with jejunal interposition.one child patient with postoperative cholangitis was mildly ill without surgical treatment. **Cell kinetics** (PCNA of the epithelium of bile duct and gallbladder) : Labelling index of PCNA was higher in adults than in children. **Conclusion:** 1) The incidence rate of carcinogenesis and postoperative cholangitis were higher in adults than in children. 2) The earlier detection and treatment of pancreaticobiliary maljunction should be desirable, especially in childhood.

F220

### EFFECTIVENESS OF BILE CYTOLOGY IN THE DIAGNOSIS OF GALLBLADDER CARCINOMA

LA Hidalgo, JM Badia, R Muns*, C Admella*, J Feliu, A Bianchi, JM Gubern.
Department of Surgery and Department of Pathology*.
Consorci Sanitari de Mataró. Mataró, Barcelona, Spain.

Non-advanced gallbladder carcinoma (GC) usually remains unsuspected at operation and diagnosis is made postoperatively by the pathologist. However, the entire organ is not included for pathological examination in a macroscopically normal gallbladder.
***Aim***: To analyze the value of bile cytology in the diagnosis of gallbladder carcinoma.
***Methods***: Bile samples obtained intraoperatively by fine needle aspiration (FNA) from 311 patients were studied (99 men, 215 women; mean age 65 years, range 27-95). All the patients were operated either by laparotomic or laparoscopic approach for gallbladder disease (mainly cholelithiasis). Ultrasonography was suspicious of GC in 4 patients. Sample A was obtained through aspiration of 5 mL of gallbladder bile. Sample B was obtained after infusion of 5 mL of physiologic saline in the gallbladder lumen and subsequent aspiration. Early cytologic diagnosis was done intraoperatively (Dif-quick stain), but definitive diagnosis was delayed until a Papanicolau technique was performed. Routine pathologic examination of the gallbladder was carried out by the pathologist without previous knowledge of the cytologic result.
***Results***: Cytologic examination was positive for malignant cells (GC) in 11 patients. The 4 cases of preoperative suspicion of carcinoma were confirmed. There was a false positive in a case of chronic reactive dysplasia. The remaining 6 cases of GC were deemed chronic or acute cholecystitis by surgeons at operation. No differences were observed between bile samples A and B, neither between Dif-quik and Papanicolau methods. Pathologists were unable to identify 2 cases of GC in situ in the first microscopic examination. The diagnosis was made in a second evaluation after the cytologic results were disclosed.
***Conclusions***: 1) Intraoperative bile cytology obtained by FNA of the gallbladder allows an early diagnosis of GC; 2) Routine pathologic examination of the gallbladder can miss a small number of GC cases that can be diagnosed by bile cytology; and 3) Intraoperative bile cytology is indicated when macroscopic appearance of the gallbladder suggests GC.

### F221

### EXTERNAL AND INTRA-LUMINAL RADIATION THERAPY (ILRT) FOR PROXIMAL BILE DUCT CARCINOMA - LONG TERM RESULT

YY Jan, MF Chen, WM Leung*
Department of Surgery, Department of Radiation Therapy*,
Chang Gung Memorial Hospital, Taipei, Taiwan

Between January 1985 and June 1995, 16 patients with hilar cholangiocarcinoma were treated by external or/with intraluminal radiation therapy (ILRT). The mode of radiation therapy were: external radiation only in 6 patients, external + ILRT in 9 patients and another one ILRT only. The clinical status of 16 patients with hilar carcinoma are: unresectable or unsuitable resection in 9 patients, post-resection residual tumor in 3 and postoperative recurrent cancer in another 4. Radiation effects were assessed on the basis of clinical respone, cholangiographic change, choledochoscopic finding and survival times.
Clinical response after radiation are: subsided of mucinous substance from bile duct in 3 patients, general condition deteriorated in two patients and other eleven patients with stationary condition after radiation. Post-treatment. cholangiogram revealed patency of bile duct, decrease size or diminished the filling defect of bile duct in six patients (37.5%). Post-treatment choledochoscopic finding revealed nodular or papillary tumor necrosis with decreased tumor size in six patients (6/13; 46.1%), tumor necrosis only in 4 patients and dutal fibrosis in another 3. The survival times of 16 patients with hilar carcinoma after complete radiative therapy are ranged from 3 to 112 months (mean 29.8M, median 15.5 M) with 1-y, 3-y and 5-y survival rates are 56.2%, 37.5% and 25% respectively.

### F222

### TRANSFERRIN, IRON AND CHOLESTEROL GALLSTONE PATHOGENESIS.

S.M. Johnston, M.K. Fox-Talbot, S.A. Martin, P.A.Lipsett, K.D.Lillemoe, H.A.Pitt. Department of Surgery, The Johns Hopkins Medical Institutions. Baltimore ,MD.

Transferrin is a nonmucin glycoprotein which binds and transfers iron. We have recently reported that the 84kDa nonmucin glycoprotein which is a potent nucleator of cholesterol crystals, is biliary transferrin (Hepatology 1995; 16:110A). However, no data are available on the levels of transferrin or iron in patients with or without cholesterol gallstones. Therefore, we tested the hypothesis that biliary transferrin and iron levels are elevated in patients with cholesterol gallstones. Gallbladder bile was collected from 13 control patients without gallstones and 23 patients undergoing cholecystectomy for symptomatic, multiple, cholesterol gallstones. Control and gallstone patients were similar with respect to age, gender and race. Neither the controls nor the patients had acute cholecystitis or abnormal liver function tests. Biliary transferrin (Tf) levels were measured by a serial radial immune diffusion assay. Total protein (TP) was measured by a Peterson / Lowery assay. Biliary total iron and unbound iron binding capacity levels were measured by a spectrophotometric assay modified to prevent interference by bilirubin. Percent iron saturation (SAT) was calculated from these measurements. Results were:

| | N | Tf $\mu$g/ml | Tf/ TP % | Total Iron $\mu$g/dl | SAT % |
|---|---|---|---|---|---|
| CONTROL | 13 | 110±39 | 1.6±0.6 | 171±64 | 38±7 |
| CHOLESTEROL | 23 | 293±44* | 6.2±1.7† | 390±107‡ | 70±8† |

*$p<0.01$, †$p<0.02$ ,‡$p<0.05$ vs Control

These data suggest that patients with multiple cholesterol gallstones have significant increases in gallbladder bile transferrin, percent transferrin, total iron ,and percent iron saturation. We conclude that an alteration in iron metabolism may be linked to the pathogenesis of multiple cholesterol gallstones.

### F223

### BILIO-BILIARY FISTULA AND CONFLUENCE STONE --- WITH OR WITHOUT CARCINOMA

J. Kamiya, Y. Nimura, N. Hayakawa, S. Kondo, M. Nagino, M. Kanai, M. Miyachi
The First Department of Surgery, Nagoya University School of Medicine, Nagoya, Japan

Stones in the gallbladder with inflammation or carcinoma make bilio-biliary fistula (BBF) or confluence stone (CS) in some cases. Although the differentiation of malignant and benign lesions in the gallbladder is important for a rational treatment, it is not easy to achieve a definite diagnosis due to interference of the stone in or around the fistula.
In our clinic, percutaneous transhepatic biliary drainage (PTBD) has been performed for patients with obstructive jaundice routinely since 1975 in order to obtain high quality cholangiograms for diagnostic purposes and to relieve jaundice. Furthermore, since 1977, we have adopted percutaneous transhepatic cholangioscopy (PTCS) to obtain a more accurate preoperative diagnosis. In the following, we review our experiences with BBF and CS.
1. BBF and CS in patients with gallbladder carcinoma
One hundred patients with gallbladder carcinoma underwent PTBD, 49 of them did PTCS. Five had BBF, one CS. Five of six cases had gallstones. Preoperative diagnosis of BBF or CS was achieved in all cases and cholangioscopic biopsy from the gallbladder through the fistula verified carcinoma in four cases. Carcinoma was highly suspected by the imaging diagnosis in the other two cases, though large stones made it impossible to take biopsy of the gallbladder. All cases underwent curative resection.
2. BBF without carcinoma
Seven patients with benign stenosis at the proximal bile duct underwent PTCS (excluding patients with primary sclerosing cholangitis). All had stones in the gallbladder. Three had BBF, two of which were revealed by PTCS. The cholangioscopic biopsy of the gallbladder through the fistula was useful in these two cases.
3. CS without carcinoma
One hundred and forty-eight patients with bile duct stone underwent PTCS. Ten cases (6.7%) were CS. Cholangioscopic lithotomy were successful in all patients. After lithotomy cholangioscopic biopsy of the gallbladder showed no carcinoma in any case. Cholecystectomy was not indicated in nine cases because the gallbladder shrinked remarkably after PTCS.
In conclusion, it is recommended that PTCS should be used for definitive diagnosis and non-surgical treatment of BBF and CS.

### F224

### CHANGES IN THE LEVELS OF BILE ENDOTOXIN AND CYTOKINES AFTER BILIARY DRAINAGE FOR ACUTE CHOLANGITIS

A. KANAZAWA, H. KINOSHITA, K. HIROHASHI, S. KUBO, T. TSUKAMOTO, T. SHUTO
Second Department of Surgery, Osaka City University Medical School, Osaka, Japan

To assess the effects of bile endotoxin on the pathophysiology of acute cholangitis (AC), we investigated changes in the levels of bile endotoxin and serum cytokines after biliary drainage for obstructive jaundice with or without AC. Patients who underwent percutaneous transhepatic cholangiodrainage (PTCD) for obstructive jaundice were classified as those with AC (group A; n = 5); those with a history of AC (group B; n = 4); and those without a history of AC (group C; n = 10). The concentrations of bile and serum endotoxin, the serum inflammatory cytokines interleukin 6 (IL-6), and IL-8, and IL-1 receptor antagonist (IL-1ra) were measured before PTCD, 5 h after, and 1, 3, 5, 7, 10, and 14 days after. Endotoxin was assayed by the Endospecy method. The levels of IL-6, IL-8, and IL-1ra were measured with kits for enzyme-linked immunosorbent assays. Results for serum endotoxin were positive (at above the cut-off value of 3 pg/ml) in 3 patients in group A, one patient in group B, and 2 patients in group C. The median concentration of bile endotoxin was higher in groups A and B than in group C. Serum endotoxin levels, if high, decreased to below the cut-off value after PTCD. Bile endotoxin levels in group C, if high, had decreased by 5 hours. This concentration in group A, if high, decreased slowly or stayed high after PTCD. In patients with poor drainage, bile endotoxin decreased only transiently. Intestinal flora including *Escherichia coli* and *Enterococcus* were isolated from the bile in all patiehts in groups A and B. The median serum levels of IL-6 and IL-8 before PTCD were higher in group A than groups B and C. These levels in group A increased after PTCD, peaking before 1 day, and then decreased. The serum levels of IL-6 were significantly correlated to the concentration of bile endotoxin at 5 hours. The median serum levels of IL-1ra before PTCD were higher in group A than in groups B or C. Increases in bile endotoxin and serum cytokines may be important in the pathophysiology of AC. Biliary drainage eventually decreases the concentrations of these substances.

F225

### THE EFFECT OF COMMON BILE DUCT OBSTRUCTION ON BACTERIAL TRANSLOCATION FROM BILIARY TRACT TO BLOODSTREAM AND LYMPHATIC SYSTEM

T.M.Karsten, T.v.Gulik, L.Spanjaard, A.Bosma, P.Dankert, D.Gouma
Depts. of Surgery and Bacteriology, AMC, Amsterdam, The Netherlands.

***Background***: Prolonged biliary obstruction results in bile duct proliferation and disruption of the hepatocyte tight-junction at the level of the bile canaliculi. ***Aim***: To evaluate the respective roles of biliary obstruction and intrabiliary pressure in passage of bacteria from the biliary tract to the bloodstream and lymphatic system.
***M&M***: 37 male Wistar rats underwent distal common bile duct (= CBD) ligation or a sham operation. After 2 weeks a laparotomy was performed and the CBD, the caval vein and the thoracic duct were canulated. Next, a broth containing $10^8$ bacteria/ml of a specific pathogenous E.coli strain was retrogradely infused in the CBD at 5 or 20 cm $H_2O$ above baseline biliary pressure. After 5 minutes perfusion, blood and lymph samples were collected for quantitative culture analysis. Subsequently the liver was fixed for light microscopy. The infused E.coli could be visualized in the liver sections by immunohistological staining with specific monoclonal antibodies. ***Results:*** A higher biliary infusion pressure resulted in more colony forming units E.coli per ml blood in both the sham operated rats (5 cm $H_2O$, n = 10, mean 1.99 x$10^4$ *vs* 20 cm $H_2O$, n = 9, mean 11400 x$10^4$ ;p = 0.0015, MannWhitney(MW)) and the bile duct ligated rats (5 cm $H_2O$, n = 9, mean 3.5 x$10^5$ *vs* 20 cm $H_2O$, n = 9, mean 330 x $10^5$; p = 0.034, MW). Bile duct obstructed rats showed, at infusion pressures of 5 cm $H_2O$, more bacterial reflux to the bloodstream as compared to the sham operated rats (n = 10, mean 1.99 x $10^4$ *vs* n = 9, mean 35 x$10^4$ ;p = 0.0092, MW). However, at 20 cm $H_2O$ infusion pressure, there was no significant difference between the two groups (n = 9, mean 1.14 x $10^8$ *vs* n = 9, mean 33.2 x $10^8$ ;p = 0.7, MW). At 20 cm $H_2O$ infusion pressure, 2 of 9 lymph cultures were positive in the sham operated group and 1 of 9 in the CBD ligated group. None of the lymph cultures showed growth at 5 cm $H_2O$ infusion pressure in both groups.
***Conclusion:*** This study confirms the increase of translocation of bacteria from biliary tract to bloodstream with higher intrabiliary pressures. Increased translocation, however, is also present at low intrabiliary pressures after a period of biliary obstruction. Bacterial migration to the lymphatic system is of no major significance in the early phase of bacterial infusion in the biliary tract.

F226

### RESULTS OF PROSPECTIVE STUDY OF AGGRESSIVE SURGERY FOR ADVANCED CARCINOMA OF THE GALLBLADDER

S. Kawamoto, T. Hiraoka, Y. Kamimoto, K. Kanemitsu, K. Tabaru and *S. Tashiro
1st Dept of Surgery, Kumamoto University, Kumamoto, Japan
*1st Dept of Surgery, Tokushima University, Tokushima, Japan

We have adopted the radical resection for the patients with gallbladder carcinoma according to our strategy, corresponded to modes of spread of the carcinoma since 1985. We present results of our prospective study.
A total of 101 patients who underwent the resection from 1965 to 1995 was divided into non-protocol group (A) (n=60) and protocol group (B) (n=41). The mean age of the patients was 61 years-old in both group. In group B, intraoperative ultrasonography was adopted for the diagnosis of the tumor spread in depth of the wall and the invasion into the liver according to TNM system. In the protocol, a wedge resection of the liver with regional lymphadenectomy for T1b, a wedge resection and bile duct resection with regional and paraaortic lymphadenectomy for T2 was adopted. A segmentectomy of the liver including S5 and S4b was added to the case with the direct invasion into the liver within 20 mm. A pancreatoduodenectomy (PD) was performed on the case with regional nodal involvement diagnosed by the frozen biopsy. In overall, 30 of segmentectomy, 6 of extended right lobectomy and 23 of PD were performed. Tumor was as follows, in group A and B; pT1a, 6 and 1; pT1b, 3 and 2; pT2, 14 and 10; pT3, 23 and 12; pT4, 14 and 16.
In pT2 patients, 10 of group A died from recurrence (8 local recurrence and 2 distant hepatic metastases), whereas only one died form distant hepatic metastases in 14 of group B. The 5-year survival rate of pT2 tumr was 21% in group A, and 88% in group B (P=0.006). In pT3 tumor, the 3 and 5-year survival rates were 23% and 15% in group A, and 38% and 28% in group B, respectively. Six of 8 patients with recurrence in group B died from the blood-borne disease; distant hepatic (4) and lung (2) metastases without any recurrences at the hepatic resection margin. In pT4 tumor, there was no survival more than 2 years in group B, in which three patients died in hospital and the modes of recurrence was various. In patients with the extended lymphadenectomy for pT2 and pT3 tumor, a half of patients with involvement of nodes in the hepatoduodenal ligament had the involvement of distant lymph nodes, such as retropancreatic or paraaortic nodes.
In conclusion, a radical resection according to our protocol was adequate for pT2 and pT3 tumor. A bile duct resection with regional lymphadenectomy should be indicated for the case with no involvement or only involvement of pericholedochal nodes. An extended surgery including PD is necessary for the case with involvement of nodes in the hepatoduodenal ligament. Majority of blood-borne recurrence in pT3 tumor imply a possibility of the adjuvant chemotherapy to improve the survival. An aggressive surgery should not be indicated for pT4 tumor.

F227

### BILIARY ENDOPROSTHESIS WITH POLYURETHANE-COVERED METALLIC STENTS FOR MALIGNANT BILIARY STRICTURES.

Y.Kawase, H.Kawa, T.Mori, T.Suzuki, N.Igaki, T.Yamasaki, H.Kitamura and M.Fukuda.
Department of Internal Medicine and Radiology, Public Shiso County Hospital, Hyogo, Japan

Recent reports on implantation of metallic stents for malignant biliafy strictures have shown favorable results. But the problem of stent occlusion by tumor ingrowth has not been completely solved. We report our experience with polyurethane-covered metallic stents and uncovered metallic stents for malignant biliary strictures. PATIENTS AND METHODS:Thirty-one patients(13 males, 18 females, mean age 71) with malignant biliary strictures have been treated by implantation of polyurethane-covered metallic stents(6 Z stents, 13 tantalum Strecker stents, 2 nitinol Strecker stents, 1 Wallstent) and uncovered metallic stents (8 Z stents, 2 tantalum Strecker stents). Causes of obstruction were pancreatic carcinoma(6/31,19%) and biliary neoplasm(25/31,81%). We designed home-made polyurethane membrane for metaltic stents. RESULTS:Stent placement was technically successful in all patients. None of procedure -related complication was confirmed. Thirty day mortality was identical for both groups(0%). 25 weeks survival rates were 60% of polyurethane -covered and uncovered metallic stents. Stent obstruction prior to death or last follow up occurred in 4/10(40%) of uncovered metallic stents with a median time to obstruction of 24 weeks and 0/11(0%) of polyurethane-covered metallic stents. The causes of obstruction of uncovered metallic stents included tumor ingrowth, confirmed by cholangioscopy. CONCLUSION: The polyurethane-covered metallic stent is effective alternative to palliation of malignant biliary strictures.

F228

### MODE OF THE LYMPHATIC SPREAD IN CARCINOMA OF THE GALLBLADDER

Kazuhiro Tsukada, Isao Kurosaki, Yoshio Shirai, Katsuyuki Uchida, Tetsuya Ootani, Yasuhiro Oohashi, Katsuyoshi Hatakeyama.
The First Department of Surgery, Niigata University School of Medicine, Niigata City, Japan

**Purpose:** To evaluate the mode of lymphatic spread of the advanced gallbladder carcinoma and to determine standard lymph node dissection are the purpose of this study.
**Methods:** The resected specimen, intraoperative findings and prognosis in 107 patients who underwent radical surgery were studied clinicopathologically using modification of the pTNM stage of AJCC. Primary tumor was classified into three stage of pT1(N=16), pT2(N=46) and pT3-4(N=45). Paraaortic lymph node belonged to pN2 group.
**Results:** The frequency of nodal involvement (0% in pT1, 48% in pT2, and 73 % in pT3-4) was significantly different among three groups based on the stage of primary tumor. Distribution of the lymph node metasases was more widely in pT3-4. The paraaortic node metastasis was recognized as a 12% in patients with pT2 and a 23% in patients with pT3-4 and other clinicopathlogical findings were positive in most patients with pT3-4 and some patients with pT2. The patients with pN2 metastases had significantly poorer survival (16 % of 5 year) than those with pN0 (65 %) or pN1 metastases (55 %). However, 5 patients with pN2 metastases survived more than 36 months. **Conclusions**: Understanding the characteristics of the mode of lymphatic spread is very helpful for achieving the appropriate dissection of the lymph nodes. Dissection of regional lymph nodes including the paraaortic lymph node is recommended for T2-4 carcinoma of the gallbladder.

F229

FIFTEEN CONSECUTIVE HEPATIC RESECTION FOR HILAR CHOLANGIOCARCINOMA WITHOUT MORTALITY

S.W. Kim, S. Huh, K.S. Suh, Y.H. Park
Department of Surgery, Seoul National University College of Medicine, Seoul, Korea

Without hepatic resection, curative surgery for hilar cholangiocarcinoma is difficult due to invasion of perihilar soft tissues and adjacent main vascular structures and extensive metastasis to regional lymphnodes. But high postoperative mortality and morbidity rates have been reported with such an extensive operation, and the operator should select patient and determine reasonable extent of resection. In this study, we analyzed the short-term results of hepatic resection for hilar cholangiocarcinoma and tried to establish rational preoperative assessment to determine resectability and extent of resection. From October 1993 to October 1995, 15 patients had undergone hepatic resection for hilar cholangiocarcinoma. CT and cholangiography were done as preoperative assessment of tumors. In some patients, angiography and/or choledochoscopy were added. In 13 patients, jaundice had relieved preoperatively with percutaneous transhepatic biliary drainage. In 4 cases of Bismuth type IIIA, 3 extended right lobectomies and 1 right lobectomy were performed and in 8 cases of Bismuth type IIIB, left lobectomies were done. In 3 cases of Bismuth type IV, 2 left lobectomies with portal vein resections and 1 left lobectomy were carried out. Among these 15 cases, 13 caudate lobectomies were combined. Early postoperative complications developed in 7 cases including 1 case with arterial bleeding and 1 case with portal vein thrombosis, which were managed operatively. Others were controlled with conservative managements and all the complicated cases had improved without any sequalae. To summarize, hepatic resections were performed in 15 consecutive patients with Bismuth type III and IV hilar cholangiocarcinomas without operative mortality. In 2 cases, proximal resection margins were postive microscopically and in another 2 cases, disease recurred at 10 and 14 postoperative months, respectively. In 1 case, anastomotic site obstruction developed as a late complication and reanastomosis was performed without evidence of recurrence. All the patients are alive(mean follow-up of 13 months) and disease free survivors(13/15) have good quality of life. In conclusion, lobectomy or extended lobectomy with caudate lobectomy is safe for most hilar cholangiocarcinomas with Bismuth type III and even Bismuth type IV in selected cases, and more extensive surgery for advanced hilar cholangiocarcinoma should be considered whenever resection is feasible.

F230

**THE EFFECT OF RECOMBINANT $BPI_{21}$ ON THE ENDOTOXIN INDUCED MORTALITY IN RATS WITH BILE DUCT LIGATION.**

A.N.Kimmings[1,2], S.J.H.van Deventer[2], H.Obertop[1], D.J.Gouma[1]. Department of Surgery[1] and Inflammation Research[2], Academic Medical Center, Amsterdam, the Netherlands.

Surgery in patients with obstructive jaundice (OJ) is associated with higher morbidity than in non-jaundiced patients, due to increased susceptibility to endotoxin (LPS) resulting in the inflammatory cascade. Different interventions have been studied to reduce endotoxemia and cytokine induction and the resulting complications. Bactericidal/permeability increasing protein (BPI) is a naturally occurring endotoxin binding and neutralizing protein, released from the primary granules of neutrophils. It binds endotoxin, neutralizing the activity and therefore inhibiting cytokine production by mononuclear cells. In animal studies and in healthy human volunteers BPI has a protective effect in experimental endotoxemia.
The aim of this study is to determine if BPI can protect against the increased endotoxin sensitivity in rats with OJ and, by that, reduce mortality.
Male Wistar rats were used, weighing approximately 250g. A dose of 2.0 mg/kg intraperitoneal E-coli 0111 B4 LPS was chosen, given 1 week after Sham operation or bile duct ligation (BDL). Three groups of rats were studied : Sham, BDL with saline, and BDL with recombinant $BPI_{21}$ (recombinant 21 kD protein). All BDL rats were clearly jaundiced, as shown by bilirubin levels (mean ± SEM) 1 week after operation of 186 µmol/l (10), with no difference between BDL rats without 178 (25) or with BPI intervention 193 (17) (p=0.59). Levels remained <1µmol/l in Sham rats (p=0.027). Endotoxin levels, estimated by the Limulus assay, were 3.4 pg/ml (0.5) in Shams and 3.1 pg/ml (0.5) in BDLs with or without BPI just before LPS administration (ns). Two hours after LPS administration levels were ± 800 ng/ml (390) in Shams and BDLs with saline, and appeared reduced in BDLs with BPI to 40 ng/ml (34) (p= 0.09). 24 hour mortality was 1/6 in Sham rats (15%) versus 8/11 in BDL rats (75%). BPI intervention directly after LPS administration, reduced the mortality to 1/12 BDL rats (8%) (p=0.003).
Conclusions: Intraperitoneal recombinant $BPI_{21}$ treatment in BDL rats reduced the endotoxin induced mortality from 75% to 8%, a mortality rate comparable to that in non-jaundiced rats. BPI could be an interesting perioperative treatment possibility in olinical OJ.

F231

TREATMENT OF POLYPOID LESIONS OF THE GALLBLADDER IN THE ERA OF LAPAROSCOPIC CHOLECYSTECTOMY

K. Kubota, Y. Bandai, T. Noie, Y. Ishizaki, M. Makuuchi
Second Department of Surgery, University of Tokyo, Tokyo, Japan.

A retrospective study was carried out to define definitive criteria for choosing the most appropriate treatment for each type of polypoid lesion of the gallbladder (PLG). The shapes and sizes of PLGs were evaluated using ultrasound in 82 patients who had undergone surgery. Histologic examinations showed cholesterol polyps in 55 patients, adenomas in 9, cancers in 16, an inflammatory polyp in 1 and a hyperplastic polyp in 1. The diameters of 58% of the benign PLGs were less than 10 mm, whereas those of 88% of the cancers were more than 10 mm; 78% of the former were pedunculated and 56% of the latter were sessile. 7 of 8 early-stage cancers had diameters less than 18 mm, whereas those of all 8 advanced cancers were greater than 18 mm. 5 of the 8 early-stage cancers were pedunculated, and 6 of the more advanced cancers were sessile. Cholecystectomies with or without full-thickness dissection (removal of the entire connective tissue layers of the gallbladder bed to expose the liver surface) were main surgical procedures used to resect benign PLGs and early-stage cancers, whereas cholecystectomy with partial liver resection was used for more advanced cancers. Laparoscopic cholecystectomy was performed in the recent 42 patients, 4 of whom had early-stage cancers. In 17 of the 42 patients, the procedure with full-thickness dissection was performed. 8 patients with early-stage cancer and 5 with more advanced cancers were alive with no signs of recurrence after respective observation periods of 1.8 to 17.5 years and 1.8 to 16.5 years. In conclusion, a PLG with a diameter of less than 18 mm is a potential early-stage cancer and therefore can be resected by laparoscopic cholecystectomy with full-thickness dissection. However, when cancer invades the subserosal layer or beyond, a second-look operation is necessary. A PLG with a diameter of greater than 18 mm may be an advanced cancer and should be removed by cholecystectomy with partial liver resection or a more extended procedure with lymph node dissection.

F232

**CYTOLOGYOF MALIGNANT BILIARY STRICTURES IN RELATION TO TUMOUR TYPE AND DIFFERENTIATION**

Kurzawinski TR, Ahmed SW, Distante V, Deery A, Dooley JS, Hobbs KEF, Davidson BR.

University Department of Surgery, Royal Free Hospital, London, UK.

Cytology despite becoming a standard method for diagnosing the malignant nature of biliary strictures, has never been correlated with tumour type and differentiation. The aim of the study was to compare cytology results with tumour type and differentiation.
The study included 79 patients with biliary strictures (50 M, 29 F, median age 65yrs, range 19-85), who had both biliary cytology (92 samples taken at ERCP) and tissue biopsy for histology. Cytology was reported as positive or negative for malignant cells. Tumour type and differentiation was obtained by histology of resected specimen (n=30), percutaneous or intraoperative biopsy (45) or post mortem examination (4).
23 patients had pancreatic, 29 bile duct, 20 ampullary and 6 gallbladder cancer. In 1 case histology of resected bile duct showed no malignant, but cytological examination was positive for malignant cells. Cancers were graded as well (20), moderately (27) and poorly differentiated (1 carcinoma in situ, 9 differentiation not known).
Overall sensitivity of cytology was 55% (43/78) and positive predictive value 98%. There was no association between positive biliary cytology and the degree of tumour differentiation. Sensitivity of cytology for well, moderately and poorly differentiated tumours was 65% (13/20), 52% (14/27) and 48% (10/21) respectively (chi square test, p>0.5). However, there was an association between tumour type and positive cytology (p>0.02). Sensitivity for bile duct and ampullary cancer was 59% and 80% for pancreatic and gallbladder cancer 30% and 50%.
Sensitivity of biliary cytology depends on tumour type and is highest for ampullary and bile duct cancer, but unexpectedly not on degree of tumour differentiation.

### F233

**ENDOSCOPIC ULTRASONOGRAPHY AND BILIARY LITHIASIS IN THE ERA OF LAPAROSCOPIC CHOLECYSTECTOMY : PROGRESS OR FASHION ?**

F. Lacaine, T. Montariol, M-J. Boudet, C. Rey, A. Charlier, A.Fingerhut, J-M. Hay and the French Associations for Surgical Research,

8 Allée des Peupliers, 92270 Bois Colombes, France.

If operative cholangiography (OC) was a widely accepted and feasable method, there would be no need for a preoperative exam to acertain the presence or absence of a common bile duct (CBD) stone. In the era of laparoscopic cholecystectomy, as this exam is not 100 % feasable and/or interpretable, preoperative endoscopic ultrasonograpy (EU) has been proposed to detect CBD stones. Because of its drawbacks (cost and need of general anesthesia), use of EU is still controversial.The aim of this multicentric prospective study was determine the place of EU in patients for whom laparoscopic cholecystectomy is planned.
Patients operated on for cholelithiasis were selected on the basis of a preoperative score to be at risk of having CBD stones. Preoperative EU was performed within 10 days before the operation and compared with OC. Presence of CBD stones on one of these exams was systematically confirmed (or infirmed) by operative exploration.
Two hundred and fifty patients were included in the study. EU and OC were feasable in 99 % and 91 % of cases, respectively. Of 225 cases available for analysis, 206 were concordant (92 %) and 19 were discordant (8 %). When both exams were in favor of CBD stones, the presence of stones was confirmed operatively in each case (n=45). When both exams were not in favor of CBD stones, follow up confirmed the absence (n=161). In 12 instances, EU was in favor of CBD stones and OC was not : 10 were EU false positive for CBD stone diagnosis and 2 were OC false negative. In 7 instances, OC was in favor of CBD stones and EU was not : 6 were EU false negative for CBD stone diagnosis and 1 was OC false positive.
In our study : 1/ EU was more often feasable than OC ; 2/ in patients at risk of having CBD stones, performance of EU was very similar to OC ; 3/ if any, its use should decrease with the feasability of OC in laparoscopic cholecystectomy.

### F234

RELIABILITY OF PREOPERATIVE BIOPSY IN THE DIAGNOSIS OF AMPULLARY MALIGNANCY

B. Launois, A. Dorandeu, B. Meunier, B. Chareton, C. Stasik, G. Spiliopoulos, J.F. Bretagne, M. Gosselin.
Department of Digestive Surgery and Transplant Unit, CHR Pontchaillou, Rue Henri Le Guilloux, 35033 Rennes, France

The choice treatment of malignant ampulloma is pancreaticoduodenectomy. Before engaging in such major surgery, some clinicians prefer to have histological confirmation of malignancy. In case of negative biopsies, surveillance, ampullectomy and endoscopic sphincterotomy have been advocated.
**Patients and methods** : 35 patients with adenocarcinomas of the Ampulla of Vater confirmed by pathological analysis of the surgical specimens (3 ampullectomies, 31 pancreaticoduodenectomies, 1 total pancreatectomy) underwent preoperative endoscopic biopsy. There were 22 males and 13 females with a mean age of 62.5±9.5 years. Jaundice, abdominal pain, poor general health status and gastrointestinal bleeding were present in 19, 21, 22 and 8 patients respectively.
**Results** : Specimens showed protuberant and hemorrhagic papillary tumor (n = 17), a pseudovillous tumor (n = 2), an enlarged papilla (n = 5), a common bile duct dilatation (n = 4), a common bile duct nodule (n = 4) and a papillary obstruction (n = 1). Data was not available for 2 patients. 17 of 35 biopsies showed infiltrating adenocarcinoma, 1 biopsy having been obtained only after sphincterotomy. Biopsies showed an adenovillous tumor suspect of malignant transformation, an unspecified suspect lesion and a positive smear in 1 case each. Other biopsies showed mild (n = 1), medium (n = 2), or severe dysplasia (n = 4), benign tumors (n = 4), inflammation (n = 1) and hyperplasia (n = 1). No anomalies were noted in 2 patients.
**Conclusion** : In some patients preoperative endoscopic biopsies are capable of ascertaining malignancy for tumors of Vater's ampulla. However the possibility of malignancy should not be discarded in the presence of a negative biopsy and patients should be denied the benefits of resective surgery solely on the basis of a negative biopsy.

### F235

CURATIVE SURGICAL MANAGEMENT OF KLATSKIN TUMORS : THE PLACE OF LIVER RESECTION

B. Launois, J. Terblanche, JM. Catheline, JP. Campion, E. Bardaxoglou, B. Chareton, O. Azzis, S. Landen
Department of Digestive Surgery and Transplant Unit, CHR Pontchaillou, Rue Henri le Guilloux, 35033, FRANCE

The place of liver resection in the management of Klatskin tumors remains controversial with some authors suggesting that liver resection should be the rule. We assess this attitude in the light of our personal experience with Klatskin tumors.
**Patients and methods** : Between 1974 and 1993, 40 patients underwent resections for Klatskin tumors. The group comprised 23 males and 17 females with a mean age of 60 years (range : 34-81 years). The majority of tumors were stage T3 (n = 24) and 25 presented with type III biliary extension. The resectability rate was 42.5%. Surgical procedures included 11 tumor resections, and 27 combined tumor and liver resections. The latter included 7 extended right hepatectomies and in 8 cases resections were supplemented by regional vascular resections. There were also 4 liver transplantations of which 2 were preceded by organ cluster-type resections.
**Results**: There was no operative mortality among patients having undergone tumor resections, combined tumor, liver and vascular resections, or transplantation. There were 4 hospital deaths in the group having had combined tumor and liver resections. Following resections considered to be curative, the median time of survival was 23 months. When the site of the tumor was considered, mean survival was greatest for type II lesions (52 months). When TNM staging was considered, mean survival was 5 years for Tis and T1 lesions. Survival was as high as 26.7 months for type III lesions. When surgical procedures were considered, 5-year survival was excellent following tumor resection (27%) but was poor following liver resection (7%) although the latter increased resectability.
**Conclusions**: Resectability of Klatskin tumors is increased by adding liver resection. Median survival time for "curative" procedures is 23 months. Mean survival for Tis and T1 lesions is excellent (beyond 5 years) and remains good for T3 and type II lesions. However, long-term results of liver resection are disappointing when compared to those achieved by simple tumor resections.

### F236

PANCREATODUODENECTOMY(PD) FOR PERIAMPULLARY CARCINOMA -320 CASES EXPERIENCE.

Yong-feng Liu, Li-ming Wang, Jian Liang Zhang, San-Guang He
Department of surgery, First University Hospital
China Medical University, Shenyang 110001, P. R. China.

There were 1200 patients of periampullary carcinoma who had laparotomy from 1962 to 1994 in our department . 320 cases of them underwent PD, the resection rate was 25.8%, hospital mortality were 23 cases,and the mortality rate was 7.19%, hospital morbidity were 69 cases, the morbidity rate was 21.9% , Of the 320 PD, 214 were male, 106 female , The ages of the patients ranged from 26 to 73 years with a median of 52.9 years. The lesions of the 320 cases were that carcinoma in the head of pancreas were 81 cases , in common bile duct were 85 , in ampulla of Vater were 104, in duodenum were 50 . The method of resection we preferred is to follow the order of gallbladder, bile duct , stomach , proximal jejunum and duodenum initially and leave the pancreas at last , this method provides excellent exposure of uncinate process and controls bleeding easily .Gastrojejunual anastomosis was retrocolic procedure . The end of the jejunum is brought into upper abdomen in a retrocolic position, but anterior to the mesenteric vessels . Pancreatic fistula is a common and severous complication following PD . From 1962 to 1970, 43 PD with end-to-side anastomosis without pancreatic drainage were performed , the fistula occurred in 10 patients , 6 deaths after that . So we change the method to end-to-end anastomosis between the pancreas and jejunum , 14 patients with internal drainage by using of short tube ,fistula occurred in 2 cases, then we changed to a long catheter external drainage for 237 cases , fistula only occurred in 3 cases. PPPD were performed in 28 cases, gastric stasis occurred in 5 patients , it was cured by nasogastric drainage and drugs within a week. From our data shows that postoperative survival rate is poor for pancreatic carcinoma, one year survival rate is no more than half, and the three years is only 13.58%, but for carcinoma of ampulla of Vater and common bile duct , the result is better , five years survival rate for carcinoma of ampulla of Vater is 41.54 % which is the best.

F237

**BODY WATER DISTRIBUTION IN OBSTRUCTIVE JAUNDICE PATIENTS ASSESSED BY BIO-IMPENDENCE ANALYSIS ( BIA )**

*L. Lupo, O. Pannarale, P. Ricci, L. Caputi, A Panebianco, V. Memeo*

Istituto di Clinica Chirurgica, Università di Bari, Italy

Renal dysfunction frequently occurs in patients with obstructive jaundice (OJ) and changes of the total volume and distribution of body water are probably involved in its pathogenesis[1] and were detectedby invasive methods[2]. Bio-impedence analysis ( BIA ) was reported as a reliable and non invasive method to assess the total bodywater ( TBW ), the intracellular (ICW) and the extracellular (ECW) distribution in normal and pathologic conditions, by measuring the body resistance (R) and reactance (Xc) [3,4].
This study was undertaken to determine the total body water and its distribution in patients with OJ ( Bilirubin > 200 μmol/l for > 1 week ) with no evidence of renal dysfunction ( Creatinine < 120 μmol/l ). Three repeated measurements were taken at steady state in 5 cancer OJ patients before treatment and in 5 control subjects matchedfor sex, ageand weight.
Results Significantly different values of R and Xc were found between OJ patients and controls: R: 602 (498-652) vs 437 (425-468), p< 0.001; Xc: 52 (47-84) vs 43 ( 41-50), p< 0.001. TBW was reduced in OJ patients compared to controls: 33.7 ( 28.7 - 38.9 ) vs 46.5 ( 37.2 - 48 ), p<0.02 ( median value and range; Mann Whitney U test ). The depletion was present in both compartments: ECW 13.7 ( 11.7 - 17.4 ) vs 18.9 ( 17.1 - 21.5 ), p<0.02 and ICW 19.8 ( 15 - 23.4 ) vs 26.5 ( 20.1 - 27.6 ), p< 0.03, with no change of the ICW/ECW ratio.
In conclusion, the mesurement of body water in OJ patients by BIA gave results similar to those obtained with invasive methods and confirmed the alterations of water volume and distribution in these patients.

1. Br.J.Surg. 1995, 82: 877 2. Br.J.Surg.1992, 79: 553
3. J of Trauma 1992, 33: 665 4. Am.J.Clin Nutr 1994, 60: 159

F238

***LAPAROSCOPIC AND MINILAPAROTOMY CHOLECYSTECTOMY: PHYSIOLOGICAL AND METABOLIC RESPONSES.***

**A.Monaco, C.Verna+, G.Moncelli, L.Gastaldi, G.Viberti° D.Zanella°, E.Baricocchi*, M.Piaccavento* and M.Vajo**

*Dpt of Surgery, Servs of Anesthesia* and Laboratory°, Giaveno H.,Turin; II Dpt of Surgery+, Asti H., Italy*

Though laparoscopic cholecystectomy has become the operation of choice for uncomplicated gallstones, minilaparotomy is shorter, without the disadvantages of the carbon dioxide pneumoperitoneum, and with a more rapid postoperative recovery compared with use of a standard incision (Surg.1994;115:533-9 and Br.J.Surg. 1992;79: 1061-4). The aim of this study is to compare the two methods evaluating respiratory and metabolic functions in patients (pts) undergoing elective laparoscopic (group I =30) in the Giaveno Hospital and minilaparotomy (group II=30) cholecystectomy in the Giaveno and Asti Hospitals for symptomatic cholelithiasis. Minilaparotomy was performed by use of the smallest feasible transverse subcostal incision (7-12 cm.) depending on the habitus of the patient. Arterial blood gases; full blood count; serum cortisol; urinary vanillylmandelic acid (VMA), epinephrine (EN), norepinephrine (NEN) and catecholamine (CCA); serum C-reactive protein (CRP), fibrinogen, erythrocyte sedimentation rate (ESR) and serum electrolytes (Na+, K+ and Ca++) were assessed in the preoperative and the immediate postoperative time and 24 hs afterwards. The data were analyzed by "t"student test. Blood gas data demonstrated a more significant decrease in arterial oxigen pressure in pts of group II compared with those of group I, 24 hs later (P< 0.000), reflecting poorer respiratory performance. Serum fibrinogen, electrolytes and cortisol dosages showed no significant differences in both groups, while urinary VAM, EN, NEN, CCA were significantly less for pts of group I (*P<0.04; P<0.04; P<0.01 and P<0.008*,respect.). Also acute-phase responses were greatest in patients undergoing minilaparotomy as determined by ESR and CRP levels (*P<0.01* and *P<0.01*, respect.). Moreover, the decrease in hospital stay after surgery, could be observed in the group I. These findings suggest that laparoscopic cholecystectomy may result in a reduced risk of postoperative complications.

F239

**SURGICAL TREATMENT OF CICATRICIAL BILIARY STRICTURES**

J.E.Monteiro da Cunha, M.C.C. Machado, P.Herman, T. Bacchella, S.Penteado, E.Abdo, J. Jukemura, A Montagnini. H. W. Pinotti - Department of Gastroenterology, Surgical Division, São Paulo University Medical School. São Paulo, Brazil.

BACKGROUND: Cicatricial biliary strictures are usually associated with high morbidity and mortality rates, frequently related to technical difficulties of the surgical repair, mainly in the hilar lesions and those complicated with portal hypertension. Extended follow-up is needed to adequately evaluate results achieved with appropriate surgical repair techniques.
METHODS: The medical records of 45 patients surgically treated for cicatricial biliary strictures between January 1984 and July 1992 were reviewed and the immediate and long term results retrospectively analyzed.
RESULTS: There were 34 females and 11 males. The average age was 42.7 years (11-72). The cause of the biliary lesion was: cholecystectomy in 18; cholecystectomy with duct exploration or reoperations for biliodigestive anastomosis in 25 and trauma in two. Thirty-seven patients. (82.2%) presented episodes of jaundice after the lesion and 31 (68.8%) presented cholangitis. Plasma bilirrubin levels were high in 31 patients. (68.8%) and alkaline phosphatase was elevated in 37 (82.2%). Diagnosis was possible by ecography in 25/33 cases with a sensitivity (S) of 75.7%, by ERCP in 10/11 (S=90.9%) and by transhepatic cholangiogram in 21/22 (S=95.4%). In 24 cases (53.3%) the stricture was located at the upper third of the bile duct, in 20 (44.4%) at the middle third and in one case (2.2%) it was low. All patients were submitted to Roux-en-Y hepaticojejunostomy with mucosa apposition. No transanastomotic stents were used. Six patients (13.3%) presented eight postoperative complications: biliary fistula (3); duodenal fistula (1) and wound infection (4). Average hospital stay was 10.8 days and there was no mortality. Only three patients developed secondary biliary cirrhosis, one with ascites, after a follow-up period of two to 10.5 years (average three years). There were no episodes of cholangitis in the late postoperative period.
CONCLUSION: Roux-en-Y hepaticojejunostomy with mucosa apposition without transanastomotic stent is a safe and efficient method for the surgical treatment of cicatricial biliary strictures.

F240

MANAGEMENT OF EARLY GALLBLADDER CARCINOMA IN THE LAPAROSCOPIC ERA.

M.Morino,C.Miglietta,C.Garrone,V.Festa,G.Cavuoti.
Istituto di Clinica Chirugica I,University of Turin.

Laparoscopic cholecystectomy (LC) is actually considered the treatment of choice for gallbladder lithiasis. Since 1991 many cases of inapparent gallbladder carcinoma (GBC) discovered after LC have been reported. Abdominal wall metastases, expecially at the port sites, and peritoneal metastatic diffusion have been described as complications of this new technique.
From March 1990 to October 1995 11 cases of inapparent GBC have been observed: 3 out of 1492 LC personally performed for cholelithiasis (0.2%) and 8 referred to our institution by other surgeons. At the time of the first operation 3 patients were classified stade I, 5 stade II and 3 stade III . The median interval between LC and re-exploration was 211 days : at this time 6 patients (2 stade I,3 stade II and 1 stade III) were submitted to a 4th-5th bisegmentectomy with radical pedicle lymphadenectomy and umbilical resection. The others 5 patients (1 stade I, 2 stade II and 2 stade III) had unresectable tumor and received a palliative treatment: 2 had diffused liver metastasis, 2 had an invasion of the common bile duct, 1 had a peritoneal carcinosis. Among the 6 resected patients one (stade I) developed a peritoneal carcinosis and died of recurrence 6 months after the hepatic resection . A second patient (stade II) developed a cutaneous metastases on the surgical limb. After the removal of this seeding he's alive without tumor recurrence 40 months after LC. 4 patients are alive 20,9,7 and 6 months after hepatectomy without recurrence. In the group with unresectable tumor median survival time was 8 months.
We suppose that LC may be involved in the early appearance of abdominal and cutaneous recurrences: to confirm this hypothesis we have organized a multicentric group to study gallbbladder carcinoma and the consequence of laparoscopic procedure.
At present, according to the literature and to our personal experience: 1. we avoid to perform LC if a GBC is suspected preoperatively; 2. we abandon laparoscopy if a GBC is diagnosed intraoperatively; 3. we perform a 4th-5th bisegmentectomy, a radical pedicle lymphadenectomy and an umbilical resection whenever a GBC is postoperatively diagnosed.

### F241

### CHOLIC ACID PROMOTES NITROSAMINE-INDUCED CHOLANGIOCARCINOMA

A. Nakeeb, S.A. Martin, M. Radosevich, R.H. Hruban, Y. Gusev, W.C. Dooley, P.A. Lipsett, K.D. Lillemoe, H.A. Pitt. Department of Surgery, The Johns Hopkins Medical Institutions, Baltimore, MD, USA

In the hamster diisopropanolnitrosamine (DIPN) induces papillary hyperplasia in the biliary epithelium which progresses to cholangiocarcinoma. In rodent models of colon cancer diets high in cholic acid have been shown to promote carcinogenesis. However, the effect of dietary cholic acid in the pathogenesis of cholangiocarcinoma has not been investigated. Therefore, we tested the hypothesis that dietary cholic acid would increase the incidence of bile duct hyperplasia in DIPN treated hamsters. Eight week old male Syrian Golden hamsters were fed either a control chow (CHOW) or a 0.5% cholic acid enriched (CA) diet. In each group, animals underwent weekly subcutaneous injection with either normal saline (NS), or DIPN (500 mg/kg) for 10 weeks. At thirty weeks, livers were harvested, hepatic bile and serum were collected. The incidence of bile duct papillary hyperplasia, the liver function tests, and the percent of glychocholic acid in hepatic bile were:

| | N | Papillary Hyperplasia | AST (IU/L) | ALT (IU/L) | Alk Phos (IU/L) | Glycocholic acid |
|---|---|---|---|---|---|---|
| CHOW + NS | 11 | 0 | 24±4 | 26±5 | 47±4 | 28%±9 |
| CA + NS | 11 | 9% | 30±7 | 29±7 | 72±7† | 41%±7 |
| CHOW + DIPN | 13 | 15% | 50±6† | 128±31‡ | 51±8 | 39%±7 |
| CA + DIPN | 9 | 77%* | 51±7† | 125±31‡ | 75±5† | 62%±5^ |

*p<0.01 vs others, † p<0.05 vs CHOW+NS,

‡p<0.05 vs CHOW+NS,CA+NS, ^p<0.05 vs others

These data suggest that 1) a cholic acid enriched diet significantly increases the incidence of papillary biliary hyperplasia in DIPN treated hamsters, 2) DIPN causes hepatotoxicity, and 3) cholic acid results in biliary stasis. We conclude that cholestasis caused by cholic acid promotes nitrosamine induced cholangiocarcinoma.

### F242

### PREVELANCE OF PORT SITE METASTASES AFTER DIAGNOSTIC LAPAROSCOPY IN GASTROINTESTINAL MALIGNANCIES.

E.J.M.Nieveen van Dijkum, L.Th.de Wit, H.Obertop, D.J.Gouma. Department of Surgery, Academic Medical Center, Amsterdam, The Netherlands.

Diagnostic laparoscopy and laparoscopic ultrasonography is increasingly used for staging of gastrointestinal malignancies. Recently many reports on port site metastases after laparoscopic colonresections and laparoscopic cholecystectomies for occult galbladder carcinoma were published and induction of port site metastases after diagnostic laparoscopy is also suggested. Therefore the prevelance of port site metastases after diagnostic laparoscopy was assessed in this study.

**Patients and Methods:** All records of patients, who underwent a diagnostic laparoscopy between January 1992 and July 1995 for staging of a gastrointestinal malignancy, were retrospectively analysed for the appearence of port site metastases in the trocar-scars. Included were 250 patients; patients with an esophageal tumor (n=66), a periampullairy tumor (n=121), a proximal bile duct tumor (n=26), a livertumor (n=24) or other intra-abdominal malignancies (n=13).

**Results:** Seven patients (2,8%) were lost from follow up. Four patients developed port site metastases, one with a neuro-endocrine tumor, one with a proximal bile duct tumor and two with pancreatic head tumors, respectively 2, 3, 5 and 10 months after diagnostic laparoscopy. Two patients had atypical, but no malignant cells in the peritoneal lavage fluid of the diagnostic laparoscopy and during the procedure biopsies were taken in 2 patients to prove irresectable disease. None of the 4 patients underwent tumor resection, the 2 patients with pancreatic head malignancies both underwent laparotomy with a palliative bypass. These patients did not develop metastases in their laparotomy scars. All 4 patients were in an end stage of their disease and underwent only palliative treatment. One patient is still alive one month after detection of his port site metastases, the other 3 patients died within 3 months after development of port site metastases.

**Conclusion:** Port site metastases occured in 1,6% (4/250 patients) as a late complication of diagnostic laparoscopy for gastrointestinal malignancies. Remarkable is the fact that no metastases were found in the laparotomy scars, indicating that not only dissiminated intra-abdominal disease but also a laparoscopy related-factor, for instance the pneumoperitoneum, must be responsible for this phenomenon. Although the precise mechanism for this complication is unclear, the occurence in potentially curable disease should be prevented. Therefore during laparoscopy biopsies should only be taken to prove incurable disease and resectable tumors should not be biopsied.

### F243

### Laparoscopic treatment of residual stones after cholecystectomy.

*L. Novellino; M. Longoni; M. Vitellaro; L. Spinelli; A. Casati; M. Andretta; G. Faillace; A.. Piazzini Albani; G. Perrucchini; L. Campanati.*

Department of general and Minimally-Invasive Surgery - Policlinico San Marco - Zingonia (BERGAMO) - ITALY

Cholecystectomy carried out with the video-laparoscopic technique, today considered a tested and codified operation, not only has the merit of having introduced a new method in the field of surgery, but, above all, of having proposed a new way of interpreting and dealing with surgery: mini-invasive surgery or, as it has been also called, "respectful" surgery, could in the next few years be increasingly performed and have a wider and more important application

When a common bile stone is encountered after laparoscopic cholecystectomy, two options are available to the surgeon: to perform a ERCP-PTSE or surgical treatment.

In the period between February 1992 and October 1995, 1041 consecutive laparoscopic cholecystectomies were performed. In four patients were found choledochal calculosis after surgical treatment and underwent ERCP a choice that we still consider optimal when is sufficient.

In two patients was not possible to remove the stones by endoscopic tecnique and we underwent the patients to a laparoscopic choledochotomy.

Direct laparoscopic choledochotomy, after laparoscopic cholecystectomy, requires a very carefully dissection to define the anterior common duct wall, usually a fibrotic tissue can be found. Then, a longitudinal incision is made in the common duct for a distance of about 1 cm, and exploration can proceed. At the conclusion of the exploration, a T-tube is placed and the duct is securely closed with intracorporeal suturing techniques. It is racommended that a small drain be placed in the region of Morrison's pouch to control any liquid collection.

The patients had a regular post-operative course; intracholedochal drainage was removed 13 days after the operation, after x-ray check-up; this patient was dismissed eighteen days after the operation.

At present we believe that the laparoscopic cholecystectomy can be considered the "gold standard"; if a residual choledochal calculosis appear the patient can can be candidate to a endoscopic treatment and in case of failure a laparoscopic choledochotomy can be performed.

### F244

### ENDOSCOPIC RESECTION OF TUMORS IN THE PAPILLA OF VATER

K.Ohhashi, T.Furukawa

Department of Gastroenterolgy, Aichi Cancer Center Hospital, Nagoya, JAPAN

(Purpose)
This study aims to reveal clinical benefits of endoscopic resection of tumors in papilla of Vater.

(Subject and Method)
A total of 12 cases of tumor in papilla of Vater was endoscopically resected. Indication was decided by duodenoscopic findings, biopsy, ERCP, hypotonic duodenography and intraductal ultrasonography(IDUS). Tumor resection was performed only in the case of adenoma or carcinoma limited in mucosal layer by using snare and high-frequency current under endoscopic control. Clinical follow-up including pain, GI tract bleeding and laboratory data was continued until recovery.

(Result)
Tumor consisted of 1 adenocarcinoma and 11 adenomas ranging 40mm to 8mm in size as a result of pathological study. Repeated endoscopy showed complete removal of the tumor in all cases. In 2 cases, GI bleeding necessitated blood transfusion was noted. Transient elevation of serum amylase continued for a few days. In 1 case, post operative jaundice occured and subsided spontaneously a week later. No other complication was noted.

(Conclusion)
Endoscopic resection of tumor (adenoma and carcinoma in mucosal layer) in papilla of Vater indicates safe and less invasive procedure compared with surgical pancreatoduodenectomy.

F245

### CAN THE NEGATIVE ERCP RATE IN PATIENTS UNDERGOING CHOLECYSTECTOMY BE REDUCED?

R.Oodit, PC Bornman, J.Krige, J.Terblanche, MEC van Wyk

Department of Surgical Gastroenterology, Groote Schuur Hospital, University of Cape Town, South Africa.

Despite a selective approach to preoperative ERCP only 31% are positive in our experience.A retrospective study was done on all patients who had successful ERCP'S preoperatively between 01 December 1990 and 30 June 1995 one. The indication for ERCP was based on either one or a combination of the following "markers":- an elevated - total bilirubin and/or alkaline phophatase and ultrasound features of a dilated commom bile duct (CBD) >6mm and/or the presence of CBD stones. Patients were grouped according to their pre-operative diagnosis :- biliary colic(BC), gall stone pancreatitis (GSP) and acute cholecystitis (AC). The results were analysed using t and Fisher tests.There were 167 patients :-115 with BC, 33 GSP, 19 AC. The age ranged from 16 to 93, mean 49.3.There were 135 (81%) females and 32 males. 53/167 (31%) had positive ERCP's. 44 in the BC, 4 in the AC and 5 in those with GSP. The most useful predictor of CBD stones (CBDS) in the biliary colic group was the presence of a dilated CBD on ultrasound :- 43 of 45 patients with dilated ducts had CBDS confirmed at ERCP.(positive predictor value 88, negative predictor value 98) and a sensitivity of 98% and specifity of 92%. In conclusion the presence of a dilated CBD in those with biliary colic is an accurate marker of CBDS and this could be used to reduce the rate of negative ERCP's in this subgroup of patients. None of the other "markers", either in isolation or combination, proved to be reliable predictors of CBD stones in any of the other subgroups.

F246

### MANAGEMENT OF MALIGNANT HILAR BILIARY OBSTRUCTION - EXPERIENCE IN 58 CASES

RTA Padbury, A Loh, GW Dyke, TG Wilson, R Davies* and J Toouli.
Departments of Hepatobiliary Surgery and Radiology*, Flinders Medical Centre, Bedford Park, S A 5042

Palliation of jaundice caused by malignant high bile duct obstruction remains a clinical dilemma. A retrospective analysis of 58 patients treated over a 7 year period (1987-1994) is presented. The median age was 66 years (range 43 to 92 yrs) with 23 men and 35 women. The median survival for all patients was 5 months.
The primary diagnostic modality was endoscopic retrograde cholangiography (ERC) in 55 patients and percutaneous transhepatic cholangiography (PTC) in 3. Endoscopic stenting was attempted in 39 patients with technical success in 25. 11 stents failed due to blockage, incomplete drainage or infection or infection requiring either a percutaneous drainage procedure (8) or surgical bypass (3). Thus, endoscopic stenting alone was successful in 36% of those in whom it was attempted. A variety of percutaneous transhepatic biliary drainage (PTBD) procedures (external drains, internal/external drains and internal Wall stents) were employed successfully in 30 patients (52%). 20 patients had not undergone a previous decompression procedure and 10 had PTBD for failed endoscopic stents or recurrence after surgical bypass. Surgical bypass was performed in 8 patients (median survival -14 months); 2 required percutaneous procedures for recurrent jaundice at a later date. The most effective modality for relief of jaundice was surgical bypass. Normal bilirubin levels were rarely obtained (12% of patients) after endoscopic stenting.
In conclusion, malignant hilar obstruction is best managed by a multidisciplinary team utilising endoscopic, radiological and surgical approaches. Endoscopic stenting alone was successful in a quarter (14/58) of patients. Percutaneous techniques can be considered for first line management or for rescue following endoscopic or surgical procedures. Hepaticojejunostomy in selected patients may give better palliation for potential longer term survivors.

F247

### FEASIBILITY, SAFETY AND EFFICACY OF LAPAROSCOPIC CHOLECYSTECTOMY AND COMMON BILE DUCT (CBD) EXPLORATION IN UNSELECTED PATIENTS

A. Paganini, F. Feliciotti, *F. Carlei, *D. Lomanto, M. Guerrieri, *M. Nardovino *M. Sottili, A. Tamburini, E. Lezoche
Istituto di Scienze Chirurgiche, Università di Ancona. *I.N.I. Canistro, L'Aquila. Italy

Feasibility, success rates, safety and short-term results were prospectively evaluated in a consecutive series of unselected patients undergoing laparoscopic cholecystectomy (LC) and common bile duct exploration (LCBDE) at the same operative session. Routine intraoperative cholangiography demonstrated CBD stones in 155 patients (97 females, 58 males, mean age 55.1 years, age range 12-94 years) out of 1300 with gallstones undergoing LC. CBD stones were unsuspected in 68 patients (5.2% of 1300; 43.8% of 155). Thirty-two patients had been referred for surgery after failed endoscopic sphincterotomy (ES) performed elsewhere. Laparoscopic trans-cystic duct CBD exploration was attempted first in all patients. When this was not feasible, laparoscopic choledochotomy was resorted to. Biliary tubes were employed selectively. Laparoscopic treatment of CBD stones was completed in 151 patients (97.4% success rate), by the trans-cystic route in 102 (no biliary drainage employed in 75) and after choledochotomy in 49 (biliary drainage in 48). Four patients were converted to open surgery (2.6%), 2 of whom were among the first 5 patients of the series. Retained stones were observed in 10 patients and were treated by ES in 3 cases and by percutaneous endo/fluoroscopic lithotripsy in 6 (with ESWL in 1). Minor morbidity included biloma (2), port site infection (2), hyperamylasemia (3) and subumbilical hematoma (1) (5.2%). Major morbidity was cystic duct bile leakage and haemoperitoneum in 3 cases each (3.9%), the latter requiring reoperation in 2 cases (laparoscopic 1). Mortality was observed in 1 (0.6%) elderly, ASA 4 patient referred for cholangitis after several failed attempts of endoscopic clearance, who died from cardiogenic shock 3 days after successful laparoscopic treatment. LCBDE during LC was feasible, safe and effective with short-term results that are not worse than those reported after ERCP/ES and LC.

F248

### LAPAROSCOPIC CHOLECYSTECTOMY IN CIRRHOTIC LIVER

C. PALANIVELU *M S Mch.*, P S Rajan, S V Sivakumar, K Sendhilkumar, R Parthasarathi, Dept. of Surgical Gastroenterology, Coimbatore Medical College and Hospital, INDIA.

Liver cirrhosis which was considered as a relative contraindication to Laparoscopic Cholecystectomy is now being successfully managed by the same. Since July 1991, over 1950 LC's have been performed among which 94 were cirrhotics. 40 patients presented with features of acute cholecystitis and 54 with chronic cholecystitis. By avoiding Cholecystostomy and open conversion, morbidity has been very much reduced. The average duration of hospital stay was 2-3 days.

**Problems encountered :** 1) Difficult Liver retraction 2) Deeply placed hilum due to nodular hypertrophy of Liver 3) Increased abnormal vessels due to portal hypertension 4) Hard, fibrotic liver bed 5) Extensive inflammatory phlegmon and adhesions 6) Extreme laterally placed gallbladder due to shrunken right lobe.

**Procedures adopted :** 1) Adequate exposure of hilum was obtained by using **additional trocars** for lifting of liver with dipping retractors (28) and downward traction (50) of duodenum and omentum. 2) **Modified subtotal cholecystectomy :** Type I LC was performed in cases of nodular liver bed where posterior wall of gall bladder was left intact with the liver after mucosectomy or electrocoagulation of the mucosa and thus avoiding uncontrollable haemorrhage. Type II LC was performed in the presence of PHT, extensive pericholecystic fibrosis, increased phlegmon and deeply placed hilum. Here, the infundibulum was divided circumferentially and muscular flap was sutured to cover the cystic stump after mucosectomy, thus avoiding undue bleeding and injury to CBD or duodenum 3) **Retrograde cholecystectomy** was performed in cases of difficult or inadequate hilar exposure.

**Conclusion :** Laparoscopic Cholecystectomy either standard or its modifications may be safely performed in cirrhotic patients. *Policy of keeping away from the danger zone* makes the procedure safer and avoids undue bleeding and CBD injury.

### F249

### MORPHOMETRIC EVIDENCE OF GUT MUCOSAL INJURY IN OBSTRUCTIVE JAUNDICE

RW Parks, WDB Clements, C Gannon[#], C Pope, BJ Rowlands, T Diamond.
Departments of Surgery and Pathology[#], The Queen's University of Belfast, Northern Ireland.

Gut barrier dysfunction has been implicated in the development of the complications seen with obstructive jaundice. Physical injury of the gut mucosa may promote bacterial translocation (BT). This study investigates histological changes of the small and large bowel mucosa in relation to BT in obstructive jaundice. Three groups of Wistar rats were studied {controls, sham operation, bile duct ligation (BDL)}. After one week, bacteriological cultures of portal blood, mesenteric lymph nodes, liver and spleen were performed. Segments of jejunum, ileum, caecum and colon were assessed morphometrically using a computerised image analysis system. Significant BT [68.8% BDL vs 6.3% Sham vs 0% Control; P<0.001)] was demonstrated following BDL. There was no significant alteration of the jejunal or large bowel mucosa, however morphometric evidence of ileal mucosal injury was demonstrated. Results are expressed as mean (SEM).

| | Ileal measurements (μm) | | |
|---|---|---|---|
| | Mucosal thickness | Villous height | Crypt depth |
| Control | 744 (95) | 559 (79) | 183 (19) |
| Sham | 731 (27) | 515 (18) | 193 (11) |
| BDL | **650 (23) *** | **451 (20) *** | 180 (8) |

[*P<0.02, Mann-Whitney U test]. These data demonstrate morphometric evidence of ileal mucosal injury and BT following BDL, supporting the hypothesis of gut barrier dysfunction in obstructive jaundice.

### F250

### COMBINED ENDOSCOPIC AND LAPAROSCOPIC APPROACH TO CHOLELITHIASIS AND CHOLEDOCHOLITHIASIS.

D. Parolini, L. Giangreco, S. Spagnolo, M. Pansera, L. Fanti°, E. Masci°, C. Staudacher
Dept. of Emergency Surgery, °Dept. of Endoscopy, S. Raffaele Hospital, University of Milan, Milan, Italy

The treatment of cholelithiasis with concomitant choledocholithiasis (CCL) has recently completely changed, thanks to development of laparoscopic and operative retrograde endoscopic techniques on the biliary system. Aim of our study was to compare combined endoscopic and laparoscopic treatment (CT) with traditional surgical treatment.
Between June 93 and August 95 we observed 37 patients with CCL (mean age 53.1±17.3, range 17-84). All these patients were candidated to CT, but one out of them had a severe necrotizing acute pancreatitis post-ERCP and underwent cholecystectomy during laparotomy for pancreatic necrosis debridement. All the other 36 patients underwent CT which consists of ERCP with common bile duct (CBD) stones removal and laparoscopic cholecystectomy. We compared them with a second group of 53 patients with CCL previously admitted and treated with open surgery (mean age 62.8±14.6 years, range 28-89), with particular regard to hospital stay and complications. In the second group cholecystectomy was associated to transcystic removal of CBD stones in 16 cases, choledochotomy in 16 cases, transduodenal sphincterotomy in 17 cases and biliary-enteric by pass in 4 cases. External drainage of the CBD was provided in 30 patients. Complication rate of the first group was 2.7% (1 case of hemobilia without need of blood transfusions), and mean hospital stay was 11.5±5.2 days. Seven out of the second group patients had complications (13.2% p=0.005): 5 had residual CBD stones, 1 had wound infection and 1 had oedematous pancreatitis. Mean hospital stay was 18.3±6.7 days (p=0.0001). In conclusion CT represents the gold standard of treatment of CCL. It provide a significative lower rate of complications, shorter hospital stays and postoperative recovery than surgical traditional treatment.

### F251

### Unsuspected gallbladder cancer and laparoscopic cholecystectomy

J.Pekolj, A. Aldet, R. Sendin, J.Sívori, M.Ciardullo, E. de Santibañes

HPB Surgery Section, General Surgery Service, Hospital Italiano, Buenos Aires, ARGENTINA.

Laparoscopic cholecystectomy (LC) is the "Gold Standard" of the treatments for symptomatic gallstones, and its contraindications has sharply decreased. Nevertheless, gallbladder carcinoma (GC) is still a formal contraindication. Its incidence is 1,5 - 2 % among the patients operated for gallstones, and the preoperative diagnosis is routinly difficult, except in advanced cases.
We reviewed 13 patients with GC and LC treated between september 1991, and november 1995. Ten were of our serie, among 1231 LC (0,8%), and 3 were sended from other hospitals. Eight were female, and the mean age was 63,5 years ( 50 - 88 ). No case was diagnosed before LC. One was suspected after initial laparoscopic exploration, and other after LC, in "ex situ" exploration of the gallbladder was confirmed by frozen section. In all the restant cases, the diagnosis was performed after delayed microscopic study.
In tree cases was performed resection of segments IV B and V of the liver, lymph nodes and trocar sites. In four cases a paliative procedure was done. In one, only exploratory laparotomy was performed. In five cases no other procedure than LC was realized. In four cases tumor recurrence in port sides were observed. In tree, the recurrences were excised.One was reexcised after recurrence in the scar of subcostal incision for liver resection. In two cases was not evidence of extense disease and are still alive after 6 and 11 months of follow up.
**Conclusion:** The GC and LC, is still a not resolved problem, because the preoperative diagnosis is uncommon feature. However if GC is suspected, open cholecystectomy must be performed. The recurrences in trocar sites are a "new" complication, and does not mean in all the cases a sign of diffuse metastasis or incurable stage. The resection of recurrences is the treatment to resolve the local pain and discomfort; and is the way in wich we can somewhat extend the survival time.

### F252

### Laparoscopic transcystic choledocholithotomy (LTC)

J.Pekolj, R Sendin, A. Aldet, J.Sívori, E. de Santibañes.
HPB Surgery Section, General Surgery Service, Hospital Italiano
Buenos Aires ARGENTINA.

Since the introduction of laparoscopic cholecystectomy (LC), there has been widespread debate about the best treatment for common bile duct stones (CBDS), associated to gallstones. Different surgical series demostrated a high effectiveness of the laparoscopic approach for the treatment of CBDS, and the transcystic access is the most accepted.
Between september 1991 and november 1995, we perform 1231 LC, with sistematic indication for intraoperative cholangiogram. It was possible in 96% and were diagnosticated 98 cases ( 8 %) of CBDS (39 unsuspected, and 59 suspected). The LTC was considered the first option, combining flushing extraction with Dormia baskets under radioscopic guide, mechanical lithotripsy and selective chologodochoscopy. We retrospectively revised two series. **Serie 1:** from september 1991 to march 1994, with 33 patients ( 20 unsuspected, and 13 suspected).The first 4 cases were coverted to perform open exploration,.and of the remnant 29, in 23 ( 80%), the stones were resolved with LTC.In one patient was performed a laparoscopic choledochotomy with trascystic drainage, and 5 open CBD explorations. In this period, the aplicability of LTC was 23 %. **Serie 2:** from april 1994 to november 1995, with 64 cases ( 19 unsuspected, and 45 suspected). The LTC was effective in 93% ( 95 % for unsuspected, and 92 % for suspected). The aplicability was 70%. In 5 cases (6 %) laparoscopic choledochotomy without T tube (LCWTT) was performed, and 1 open exploration was necessary. In the last 6 months, 90 % of CBDS were resolved by laparoscopic approach.The morbidity of LTC was 6,6 % (2 bile leaks, 1 perforation of CBD, 1 residual stone, 1 pancreatitis, 1 hemoperitoneum), and was not mortality. The mean hospital stay was 1.8 days (1-24).
**Conclusion:** LTC is a technically feasible procedure, with high effectiveness and low complication, mortality and length hospital stay rates. It require adequate selection of patients, training in variety of techniques and equipment. In the unsuccessful and unapplicable cases of LTC the LCWTT is our treatment before the open procedure.

### F253

### PATHOPHYSIOLOGY OF ACUTE ACALCULOUS CHOLECYSTITIS

F.M. Polignano, S. Ferrarese
Cattedra di Fisiopatologia Chirurgica e Clinica Chirurgica I - Università di Bari

We report 12 patients suffering from AAC, observed between 1987 and 1994. These represented 9.4% of all acute cholecystitis and 1.5% of all admissions for biliary-related pathologies in the same period. There were 7 men and 5 women, whose average age was 64.3 years (range 31 to 89). APACHE II score, for assessment of the severity in patients with serious intra-abdominal sepsis, was evaluated on admission, before any therapeutic intervention had been performed. Scores ranged from 6 to 27 (average 13.1). 7 patients were admitted with a diagnosis of AAC; 5 were admitted with unrelated diagnosis and had symptoms develop while in the hospital. Temperature was higher than normal in 7, leukocytosis was present in 10 and leukopenya in 1 (suffering from myelodysplasic syndrome); serum bilirubin level was abnormal in 5 (greater than 5 mg% in 2) and serum amylase in 4 (greater than 1000 UI/L in 3). On admission to the hospital 5 patients were so ill that they were brought to operatory room immediately after resuscitation. On the opposite, reidratation and antibiotics caused a quick resolution of symptoms and of ultrasonographic findings in 3 patients (2 with associated inoperable colon cancer with liver metastasis and 1 with acute partial thrombosis of portal and superior mesenteric veins); they were monitored by means of real time ultrasonography and operation was postposed. Diagnosis was obtained by US in 5 patients and by CT in 3 (2 having serum amylase level greater than 1000 UI/L). Most common ultrasonographic findings were the presence of sludge, distension, thick wall, pericholecystic fluid. Underlying, associated pathologies had been: cancer and metastasis (3), severe cardiopathy (3), diabetes (3), thrombosis of abdominal or periferic vessels (2), history of gastric resection for complicated ulcer (2), neurologic disturbance with syncope (1), haematologic disease (1), cirrhosis (1), rupture of aortic aneurysm (1), anastomotic bleeding ulcer (1), peritonitis due to gunshot multiple bowel laceration (1). Potential etiologic factors had been: shock (3) and massive blood transfusion (3), parenteral hyperalimentation (1), acute renal failure (2). Activation of factor XII had been a reasonable hypotesys which might have occurred in 8 out of 12 patients. Gangrenous cholecystitis with perforation of the gallbladder occurred in 5 (average APACHE II score 15.8) out of 9 patients which underwent undelayed surgery, testifying the quick evolution of this condition and its dangerousness. Operative treatment consisted of cholecystectomy in 6 patients and of cholecystostomy in 3 (2 perforated and 1 empiematous - average APACHE II score 14.6). The overall mortality rate was 8.3% (1 patient out of 12, whose APACHE score was the highest) and that limited to those operated was 11.1%.

### F254

### KLATSKIN'S TUMOR: ASSESSMENT OF RESECTABILITY AND THERAPEUTIC ALTERNATIVES

A. Principe, M.L. Lugaresi, M.C. Gallo, I. Bicchierri, B. Nardo, A. Mazziotti, A. Cavallari
2° Department of Surgery - Policlinico S.Orsola - University of Bologna - ITALY

The severe prognosis of Klatskin's tumor is strictly correlated with it's site and it's extensive diffusion. The purpose of this study is to analyse the results obtained after adoption of an accurate pre and perioperative diagnostic algorhytm in order to localize the site of the tumor, achieve a correct staging and determine resectability. **Material and Methods-** Since 1982, 72 patients have been observed. The preoperative work-up was carried out in all cases through US, CT, and only in 17 cases with Angiography. PTC and/or ERCP allowed decompression of the bile ducts and identification of the exact site of the tumor (Bismuth classification). Two patients were excluded from this study because of death, before intervention, due to hepato-renal insufficiency, despite transhepatic biliary drainage. Preoperative staging was modified during operation in 23 cases (33%) because perioperative US and frozen sections of the margins demonstrated unexpected diffusion. A curative resection was possible only in 21 cases (R.I. 30%). Five patients were submitted to tumor resection. Of the other 16 pts, 8 were treated with a simple Hepatectomy, and 8 with an extended Hepatectomy, associated in 7 cases with resection of the caudate lobe and in 1 case with resection of the vena porta. The remaining 49 patients (70 %), were treated following three different palliative modalities: 11 patients with an Intrahepatic Bilio-Digestive-Anastomosis (IBDA), and 18 with a Surgical Biliary Drainage (SBD), 8 externally and 10 transtumorally. On 20 patients no surgical intervention was possible and they were reclosed with the Non Surgical Biliary Drainage (NSBD), positioned before surgery. **Results-** Curative procedures: there were 2 p.o. death (mortality rate 9.5%) (St II and IVa). Eleven pts (1 St.I; 4 St.II; 6 St.IVa) are currently alive (mean FU 42 months; Range 6-156 mo). The remaining 8 pts (6 St.IVa; 2 St.IVb) had a mean FU of 19 months.(Range 2-55 mo). Palliative procedures: operative mortality rate was 12.2 % (6 pts). Mean survival according to the type of biliary derivation was: 7 months for IBDA (Range 1-18 mo.); 8 months for external SBD (Range 1-30 mo) and 10 months for internal SBD (Range 2-38 mo); 4 months for NSBD. (range 1-9 mo). Survival between curative versus palliative procedures was statistically different (p=0.0005). **Conclusions-** The results suggest that extensive diffusion of the tumor is not often evidenced by the preoperative work-up (33%). Curative resection, wich can only planed with intraoperative staging, must be as wide as possible in order to achieve significant survival. Surgical transtumoral biliary intubation is the better therapeutic alternative to improve survival and quality of life in these patients.

### F255

### RESULTS AND COMPLICATIONS OF LAPAROSCOPIC CHOLECYSTECTOMY FOR ACUTE CHOLECYSTITIS

G. Ramacciato, A.M. Balesh, W. Clazzer
I Department of Surgery, University of Rome "La Sapienza," Rome, Italy

The purpose of the present study was to evaluate the benefits and effectiveness of laparoscopic cholecystectomy (LC) for acute cholecystitis (AC). A total of 800 patients, 60 of whom affected by AC, underwent the procedure from July 1991-June 1995. Average age was 53 years (range 25-76). Successful LC was performed in 79.2% cases. Average operative time significantly increased when AC was present (180±20 min) vs. in its absence (100±20 min) (p<0.01). AC also significantly lengthened postoperative hospital stay (3.5±1 days, range 1-18), in keeping with that reported by Lujan et al. (1), vs. in its absence (2.5±1 days, range 1-7). A conversion rate of 20.8% occurred in cases of AC, with the most frequent cause of conversion being technically difficult dissection of Calot's triangle due to pericholecystitis. Injury to the principal bile duct system occurred in 3.3% (2/60) cases of LC for AC vs. significantly lower incidence in elective LC (0.3%, 2/740). In the former cases, injury was immediately discernible and involved detachment of the cystic duct from the principal bile duct system. In conclusion, the effectiveness of LC for AC must be weighed against a high conversion rate, with the balance appearing to tip in favor of LC.

References

1. Lujan JA, Parrilla P, Robles R. Laparoscopic cholecystectomy in the treatment of acute cholecystitis. J Am Coll Surg 1995; 181: 75-7.

### F256

### OCTREOTIDE INCREASES BILE LITHOGENITY IN TPN-FEEDED RABBITS

Rodriguez, J, Targarona, EM*, Martínez, J,* Poca, E+, Vela, M+, Ros, E,+ Massó*, E, Nadal, J#,Marco, C, Trias, M+. *Serv. of Surgery, +Gastroenterology and#Pathology, Hosp. Clinic. Serv. of Surgery, Hosp Mutua de Terrassa, Univ of Barcelona. Barcelona, Spain

The development of gallstones is a well recognized complication of long-term treatment with Octreotide (SMS). Also, parenteral nutrition (TPN) is associated with several hepatobiliary complications including cholestasis, jaundice, biliary sludge or stones. The association of both treatments has been proposed for treatment of digestives fistulaes, but the biliary effects has not been evaluated. AIM To study the modifications of bile composition during treatment with TPN plus SMS. METHODS: 32 New Zealand rabbits were studied. Fourteen served as controls (Group I), and 6 received SMS (Group II) (1μg/kg tid sc), NPT (190 cc/kg/d, 340 Kcal/d) (Group III), or SMS + NPT (Group IV) for 2 weeks. Gallbladder bile was obtained and phospholipids, cholesterol, bile salts, total bilirrubin, total protein, and total calcium, were measured.

| RESULTS: | Control | SMS | TPN | TPN+SMS |
|---|---|---|---|---|
| Phospholip. | 10.4±3.9 | 36.3±3.8* | 17.6±4.7+ | 36.4±4.2*# |
| Cholesterol | 0.6±0.1 | 0.6±0.10 | 1.7±0.1*+ | 2.6±0.5*+ |
| Bile salts | 11.5±2.5 | 57.0±6.1* | 26.5±7.1*+ | 15.1±2.2+ |
| Bilirrubin | 10.2±2.3 | 8.6±0.7 | 23.7±1.6*+ | 29.9±3.9*+ |
| Proteins | 14.3±2.8 | 8.2±0.4 | 19.6±2.6+ | 22.3±7.0 |
| Total calcium | 44.6±12.4 | 79.3±7.0* | 185.9±20.2*+ | 91.4±17.8*# |

(X ± SE). *<.05 vs control, +<.05 vs SMS, #<.05 vs TPN

SMS induced a significant rise of phos, bile salts and Ca, and TPN was associated with an increase of chol, bile salts, bi and, Ca. The simultaneous administration of both treatments enhnaced increase of phos, chol, bi, Ca and decreased bile salts levels. CONCLUSION: 1. This study confirms that SMS or TPN administered alone, induce significative changes of gallbladder bile composition. 2. The associated administration of SMS enhances the lithogenic changes induced by TPN. 3. Biliary function must be monitored in patients receiveing both treatments in order to prevent biliary complications.

### F257

### UTILITY OF ENDOSCOPIC COMMON BILE DUCT STENTING IN PATIENTS WITH SUSPECTED SPHINCTER ODDI DYSFUNCTION

P. Rolny
Department of Medicine, Östra Hospital, Gothenburg, Sweden

**Introduction.** As Endoscopic sphincterotomy (ES) for sphincter of Oddi dysfunction is relatively risky (SOD) only patients who are likely to benefit should be selected for the procedure. Endoscopic sphincter of Oddi (SO) manometry is at present an established tool for that purpose. However the method is neither universally accurate, nor generally available. In contrast endoscopic stent placement (ESP) into the common bile duct is feasible in most hospitals. Here, we explored the utility of ESP as an alternative for selection of patients with suspected sphincter of Oddi dysfunction for ES. **Patients and methods:** The paper comprises 23 post-cholecystectomy patients aged 37-68 (mean 45) years, all byt two women who suffered from symptoms consistent with SOD and in whom alternative causes for symptoms were excluded. All patients underwent SO manometry (SO pressure > 40 mmHg is abnormal), followed by ESP and finally by ES. The patients were then followed up in the outpatients clinic for 6-16, median 12 months. Improvement was defined as complete freedom from analgetic drugs. **Results.** After ESP 17 (75%) of the 23 patients were improved and after subsequent ES eleven (65%) remained pain free long term, whereas in 6 the symptoms recurred. However, three of these have had the stent for only < 11 weeks (as opposed to 12-16 weeks in the remainder) and two had relapse of pain during stenting. Six patients had no pain relief after ESP and none of these benefited from ES. All patients with elevated SO pressure improved on ESP and they with one exeption also benefited from ES. The 5 patients with normal SO pressures who had a sustained improvment during ESP were also long term improved after ES. **Summary and conclusion.** Most of the patients with sustained response to ESP were irrespected of SO pressure rendered symptom-free after ES. If confirmed in larger trials ESP could be useful for selection of patients with suspected SOD for ES.

### F258

### RELATIONSHIP BETWEEN LYMPHNODE METASTASIS AND MODE OF RECCURENCE, PROGNOSIS IN CANCER OF THE BILE DUCT.

-Lymphnode metastasis is related to postoperative liver metastasis-

R. Sasaki, S. Kanno*, H. Nitta, M. Murakami, Y. Hayakawa, Y. Shimada, H. Kawamura, T. Suto and K. Saito
*Department of Surgery I, Iwate Medical University, Morioka and Department of Surgery, Iwate prefectural Kuji Hospital*, Kuji, Japan*

In order to reveal the relationship between lymphnode metastasis and mode of reccurence, prognosis in cancer of the bile duct, the number and degree of lymphnode metastasis were investigated about the relation to mode of reccurence and prognosis in sixty-nine resected patients of bile duct cancer.

RESULTS:

1) Lymphnode metastasis was observed in 24 of 69 patients (34.8%). There was significant correlation between the number of lymphnode metastasis and degree of lymphnode metastasis (p<0.05, chi-square test).

2) Postoperative reccurence occured 15 of 45 cases (33.3%) in patients with no lymphnode metastasis (n(-)). The site of reccurence was liver metastasis in 7 cases (15.6%), peritoneal dissemination and lymphnode reccurence in 4 cases (8.9%), local reccurence and metastasis at abdominal wall in one case (2.2%), respectively. On the other hand, in patients with lymphnode metastasis ((n(+)), reccurence occured in 13 of 24 cases (54.2%). The mode of reccurence was liver metastasis in 10 cases (41.7%), peritoneal dissemination in 3 (12.5%), lymphnode reccurence, bone metastasis, brain metastasis and pleural metastasis in one case (4.2%), respectively. Postoperative liver metastasis occured more frequently in n(+) patients than n(-) patients (p<0.05, chi-square test).

3) Five years survival rate was 54.6% in n(-) patients. Three years survival rate was 11.5% and no 5 years survivor in n(+) patients. Prognosis in n(+) pattients was significantly worse than in n(-) patients (p<0.0001, log lank test). According to the number of positive lymphnode, mean survival period was, one positive lymphnode: 575.9±121.7 days, 2 to 7: 658.5±174.4 days and more than 8: 356.3±167.8 days and n1: 625.5±99.8 days, n2: 726.7±263.2 days n3: 408.0±280.7 days and n4: 361.4±117.4 days according to the degree of lymph node metastasis. So, prognosis in n(+) patients was poor, even if the number of lymphnode metastasis was one or degree of lymphnode metastasis was n1.

CONCLUSION: These results suggest that postoperative liver metastasis occured frequently and prognosis was poor in patients with lymphnode metastasis, it is necessary to do the postoperative adjuvant therapy against the liver as a target organ for the lymphnode positive patients of cancer of the bile duct.

### F259

### PANCREATICODUODENECTOMY FOR ADVANCED GALLBLADDER CARCINOMA: LONG-TERM RESULTS

Y. Shirai, K. Tsukada, T. Ohtani, K.Hatakeyama
Department of Surgery, Niigata University School of Medicine, Niigata City, Japan

***BACKGROUND:*** The aim of this retrospective analysis was to clarify the role of pancreaticoduodenectomy (PD) in the management of gallbladder carcinoma (GBC) in a 5-year study. ***METHODS:*** Between 1983 and 1993, 19 consecutive patients with GBC underwent a classical Whipple PD combined with extended cholecystectomy. To analyze the long-term results, 17 patients who had such surgery before December 1990 were included in this study. PD was indicated for direct invasion of the pancreaticoduodenal region and/or peripancreatic nodal disease. Hepatic resection was performed in all: resection of the gallbladder bed in 15 and extended right hepatic lobectomy in 2. The 17 patients were divided into two groups: 12 with invasion of the extrahepatic bile duct and 5 without such invasion. ***RESULTS:*** Thirteen patients had Stage IVB disease. Overall, 5 patients (29%), of whom 4 had Stage IVB disease with positive peripancreatic nodes, survived 5 years after surgery. Five patients (42%) in the group without bile duct invasion survived 5 years with median survival of 57mo, compared with no 5-year survivors with median survival of 15 mo in the group with bile duct invasion (P<0.01). A 5-year survival of 45% in those undergoing a potentially curative (R0) resection (median survival, 57 mo) was significantly higher (P<0.01) than 0 % in those undergoing a palliative resection (median survival, 11 mo). ***CONCLUSIONS:*** PD provides long-term palliation for selected patients with advanced GBC with peripancreatic nodal disease. The absence of bile duct invasion and R0 resection are prerequisites for long-term survival. The results justify the use of PD in selected patients with advanced GBC.

### F260

### GALLBLADDER EMPTYING PRESSURE IN PATIENTS WITH ACALCULOUS GALLBLADDER PAIN AND IN PATIENTS WITH NORMAL GALLBLADDERS.

Richard S Stubbs and Michael WC Booth. The Wakefield Clinic for Gastrointestinal Diseases, Wakefield Hospital, Wellington, New Zealand.

**Introduction:** Chronic acalculous gallbladder pain (CAGBP) is a controversial entity. This study was undertaken to compare the GB pressures observed during emptying of normal gallbladders with that of patients believed to be suffering from CAGBP. **Methods:** GB pressures were measured directly in anaesthetised patients immediately prior to GB removal in 15 patients undergoing lap chole for CAGBP and in 10 patients who were having open removal of a normal GB at the time of major hepatic surgery for metastatic cancer (controls). Baseline pressures were recorded prior to bolus IV injection of CCK-OP (sincalide) 2.5 µg given over 1 minute. Pressures were recorded at 30 sec intervals commencing at the completion of the CCK injection (0 time) and continuing for a total of 10 minutes. The maximum pressure rise over baseline was calculated for each patient ($\Delta P_1$) as was the change in pressure from baseline at the 10 minute period ($\Delta P_2$). CAGBP patients were divided into responders (those whose problem was resolved by cholecystectomy, n = 11) and non-responders (whose problem was unchanged by cholecystectomy, n = 4). $\Delta P_1$ of responders was compared with controls and with non-responders using a two tailed Wilcoxan Signed Rank test. $\Delta P_2$ values were similarly compared. **Results:** Mean ± SE (mmHg) $\Delta P_1$ and $\Delta P_2$ were as follows:- controls, 4.50 ± 0.51 and 0.15 ± 0.68; responders, 9.27 ± 1.43 and 4.36 ± 1.23; non-responders, 5.50 ± 0.65 and 2.75 ± 0.48. The difference between mean $\Delta P_1$ in responders and controls was significant (p = 0.015). Similarly the difference between mean $\Delta P_2$ in responders and controls was significant (p = 0.044). The differences between these measurements in the non-responders and responders or controls was not significant. **Conclusion:** To our knowledge this is the first data to be collected of this kind and the findings lend support to the existence of CAGBP as an entity and provide support for the hypothesis that the pain is due to increased resistance to GB emptying.

F261

**PROSPECTIVE COMPARISON OF ENDOSCOPIC SPHINCTEROTOMY (ES) WITH GALLBLADDER 'in situ', OPEN SURGERY AND ES plus LAP. CHOLECYSTECTOMY FOR TREATMENT OF BILE DUCT STONES IN HIGH RISK PATIENTS.** EM Targarona, RM Perez-Ayuso, JM Bordas, E Ros, C Balagué, J Martínez, I Pros, J Terés, M Trías. Serv.of Surgery, Gastroenterology and Endoscopy. Hosp. Clinic. Univ.of Barcelona

Lap cholecystectomy (LC) has modified the approach to bile duct stones (BDS). During last decade, ES with gallbladder 'in situ'(EE-GiS) has been used as alternative to surgery in the high risk patient, but symptoms up to 30% during f-up has been reported and it has never been compared with open surgery. ES+LC is the most accepted therapy for BDS in the laparoscopic era. **AIM:** To compare the efficacy of open surgery with EE-GiS or ES+LC for treatment of BDS in the high risk patient. **MATERIAL AND METHODS:** From set-91 to set-94, 100 patients suspected to harbour duct stones were randomly allocated to open surgery (**Group I**, n: 48) or EE-GiS (**Group II**, n: 50). From set-94 to nov 95, 54 consecutive patients were treated with ES-LC (**Group III**) Criteria for suspicion of BDS were gallstones+jaundice, cholangitis or pancreatitis+dilated bile duct. High risk factors were: age > 70 or severe disease (cardiac, pulmonary, cirrhosis or limited mobilization). **RESULTS:**

| | Open surgery | EE-GiS | ES+LC | p |
|---|---|---|---|---|
| **Age** (y) | 80 ± 7 | 79 ± 9 | 79±7 | ns |
| **Technical success** | 94 % | 90% | 98 % | ns |
| **Morbidity** | 23 % | 16% | 10 % | ns |
| **Mortality** | 4 % | 6 % | 2 % | ns |
| **Postop stay** (d) | 11 ± 8 | 5 ± 4 | 4 ± 4 | .001 |
| **FOLLOW UP** (months) | 18 ± 10 | 15 ± 11 | 7 ± 4 | |
| **Late biliary morbidity** | 6.5 % | 20 % | 4 % | .002 |

Conversion rate was 11%. **CONCLUSION:** ES+LC is a safe and effective alternative to open surgery or EE-GiS for treatment of bile duct stones in the elderly or high risk patient.

F262

**PROGNOSTIC VALUE OF NUCLEAR DNA CONTENT AND CELL PROLIFERATION (Ki-67) IN RESECTABLE DISTAL BILE DUCT CARCINOMA**

A.Umezawa*, T.M.van Gulik, A.Bosma**, K.Koyama*, G.J.A.Offerhaus**, H.Obertop, D.J.Gouma.
Dept.of Surgery, Akita University Hospital, Akita, Japan (*), Depts.of Surgery and Pathology(**), Academic Medical Center, University of Amsterdam, Amsterdam, The Netherlands.

In 33 patients (age 31-73y, mean 60y) who had undergone subtotal pancreatoduodenectomy for distal bile duct carcinoma (DBDC), tumor DNA content and the cell cycle-associated antigen Ki-67 were studied in relation with survival time. *Methods:* Of each pancreato-duodenectomy specimen, 1-5 samples were selected from the cancer-containing tissue blocks. As controls, non-tumor areas of the distal bile duct were analyzed in 12 patients. For assessment of nuclear DNA content, a nuclear suspension was prepared from 50μ sections of each sample and stained with propidium iodide. DNA content was measured with a flowcytometry FACScan and the data of 20.000 nuclei of each sample were analyzed. Tumor ploidy was classified as diploid or aneuploid on the basis of the resulting DNA histograms. Ki-67 was detected by immunohistochemical staining of 4μ tumor sections with a new antibody: MIB-1. MIB-1 expression was recorded as the ratio of positive nuclei (%) in 1000 tumor cells (MIB-1 index). *Results:* 19 pats. had a radical resection, 14 resections were microscopically non-radical. 22 pats. were found to have diploid tumors and 11 pats. had aneuploid tumors. Median survival of patients with diploid and aneuploid tumors was 13mths and 10mths, respectively. No correlation of DNA ploidy with survival time was found, neither after stratification for radicality of the resection. Of the 12 non-tumor areas, 10 cases were diploid and 2 cases aneuploid. Mean MIB-1 index in tumor cases was 17.6%, whereas in benign areas mean MIB-1 index was significantly lower, i.e. 3.6% (p=0.0007). Univariate survival analysis of the whole group of patients showed that MIB-1 index of >20% was associated with decreased survival time, i.e. <12 months (log rank test, p=0.005) (risk ratio 2.86). *Conclusions:* 1) Assessment of DNA content has no prognostic value in distal bile duct carcinoma 2) Mean MIB-1 index (reflecting Ki-67 expression) greater than 20%, is associated with poor survival ( <12 months) in resectable distal bile duct carcinoma.

F263

**GROWTH HORMONE TREATMENT REDUCES BACTERIAL TRANSLOCATION IN OBSTRUCTIVE JAUNDICE**

**C Vagianos, S Koureleas, A Arvaniti, CD Scopa, T Alexandridis, M Stavropoulos**

**Departments of Surgery and Internal Medicine and Laboratories of Microbiology and Pathology, University of Patras, Patras, Greece**

Septic complications in the presence of obstructive jaundice may be related to increased bacterial translocation (BT) from the gut. This may be due to the lack of bile salts and their trophic effect on intestinal mucosa. We investigated the effect of Growth Hormone (GH) in maintaining intestinal mucosal integrity and reducing BT in jaundiced rats.
***Methods:*** Male Wistar rats were used divided randomly into four Groups: I (n=21), controls; II (n=21), sham operated; III (n=22), ligation and division of the common bile duct (CBDL) and IV (n=15) CBDL and treatment with Growth Hormone (GH). Laparotomies were performed on day 0, under sterile conditions. GH (250 μg/kg BW) was given s.c. once daily until sacrifice. On day 10 all animals were sacrificed. Blood bilirubin was determined, mesenteric lymph nodes (MLN) and liver specimens were cultured aerobically, as well as bile from the ligated CBD stump. By washing the colon with 2 ml of saline, 0.5 aliquots were obtained and the total aerobic flora was determined. Samples from the terminal ileum were removed for histologic examination and DNA meaurements.
***Results:*** Bilirubin was significantly increased in Groups III and IV (p<0.01). 77% of the animals in Group III had positive MLN and 36% positive liver cultures (p<0.05 vs Groups I and II). GH treatment decreased positive cultures to 35% and 0 respectively and this was statistically significant (p<0.05 vs Group III). All cultured bacteria were enteric in origin. The bile was sterile in all animals subjected to CBDL. Aerobic colonic flora was the same in all Groups (p>0.05). Histology revealed insignificant mucosal atrophy, while intestinal DNA content was significantly decreased in Group III (p<0.05 vs groups I and II). These returned to normal after GH treatment.
***Conclusion:*** Obstructive jaundice promotes BT both to MLN and liver. This may be partly due to desruption of intestinal mucosal integrity, following the lack of the trophic effect of bile salts. GH preserves mucosa, probably by treating atrophy, although immunological factors can not be excluded. This may have interesting clinical applications in patients with obstructive jaundice.

F264

FEASIBILITY OF BILE DUCT STONES EXTRACTION VIA THE CYSTIC DUCT; ANATOMICAL CONSIDERATION.

J. Vracko
Department of Gastroenterological Surgery, University Medical Centre, Ljubljana, Slovenia

Inner diameter of the cystic duct and the size of the largest common bile duct stones have been prospectively measured in a consecutive series of 30 patients undergoing open choledocholithotripsy, out of 250 patients referred to our department for laparoscopic cholecystectomy, to asses whether anatomy of the cystic duct ascertains bile duct stone extraction via the cystic duct. Average inner diameter of the cystic ducts, measured postoperatively on intraoperative cholangiograms, was 5.233 ± 2.373 mm, range 2 -10 mm, and was significantly wider as compared with the average size of largest common bile duct stones which measured 3.666 ± 2.475 mm, range 0.5 - 10 mm (p<0.05). In 14 (47%) patients bile duct stones were smaller and in 9 (30%) patients they were of equal size as compared with the concomitant diameter of the cystic duct, indicating feasibility of transcystic bile duct stones extraction in 3/4 of patients with common bile duct stones. In 7 (23%) patients bile duct stones were larger as compared with concomitant cystic duct caliber; in five patients they were 1mm and in two patients they were 2 and 4 mm larger. Therefor, anatomy of the cystic duct ascertains bile duct stone extraction via the cystic duct in 3/4 of bile duct stone patients. In 1/4, cystic duct dilatation and/or laser lithotripsy should be used. These features strongly support the concept of laparoscopic common bile duct stone extractions via the cystic duct and confirm that the transcystic access to bile duct stones is a logical way of bile duct clearance.

### F265

GUT BARRIER FAILURE IN OBSTRUCTIVE JAUNDICE

F. Welsh, CW. Ramsden, K. MacLennan, M. Sheridan, GR. Barclay, PJ. Guillou and JV. Reynolds.
Academic Surgical Unit, St. James's University Hospital, Leeds, UK.

Endotoxaemia and bacterial translocation may underlie the high incidence of septic complications in the jaundiced patient. The degree of intestinal permeability in obstructive jaundice has not been quantified. Altered mucosal immunity may also contribute. This study aimed to examine the effect of obstructive jaundice on intestinal permeability and immunological cellular infiltration. Thirty three patients were studied, 18 jaundiced (median plasma bilirubin 303μmolL$^{-1}$) and 15 non-icteric matched controls. Gut permeability was measured by the lactulose-mannitol absorbance test. Endoscopic biopsies of the second part of the duodenum were stained and morphological and immunohistochemical features graded from +1 to +4 by and independent blinded pathologist. The results (median and interquartile range) are shown below:

| | Control | Jaundiced |
|---|---|---|
| **Lactulose-mannitol ratio:** | 0.03 (0.01-0.06) | 0.24 (0.07-0.52)* |
| **Intestinal mucosal immunohistochemistry score:** | | |
| CD68 (macrophage marker) | 2 | 4* |
| CD3 (pan- T-cell marker) | 2 | 2 |
| CD30 (late activated T-cells) | 2 | 3* |
| HLA-DR (activated T-cells/ macrophages) | 1 | 3* |
| HLA-DR (epithelial cells) | 1 | 3* |

*p<0.05, Mann-Whitney U test.
There was no difference in morphology between groups.

The results show a significant increase in intestinal permeability, in association with local intestinal immunological activation in jaundiced patients. These results show intestinal barrier function to be impaired in the jaundiced patient. This may underlie the high risk of sepsis in this group.

### F266

BENIGN AND MALIGNANT GALLBLADDER LESIONS: SURGICAL RESULTS OF LAPAROSCOPIC TREATMENT IN THE PROSPECTIVE SALTS-STUDY ON LAPAROSCOPIC CHOLECYSTECTOMY

K. Z'graggen, HU. Baer, S. Birrer, F. Holzinger, L. Krähenbühl, C. Klaiber*
Department of Visceral and Transplantation Surgery, Inselspital University of Bern, Bern and Department of Surgery, Spital Aarberg*, Switzerland

Since 1992 the Swiss Association of Laparoscopic and Thoracoscopic Surgery (SALTS) performed a prospective multiinstitutional study on laparoscopic cholecystectomy (LC) including approximatly 50% of all cholecystectomies done in Switzerland. 10724 patients entered the study, and 0.97% (104 patients) were operated for benign (66 pat.) or malignant (38 pat.) gallbladder tumors.
*Results:* In gallbladder carcinoma LC was successfully performed in 60.5% (23/38), Primary conversion rate to an open procedure was 21.1% (8 cases), 7 cases(18.4%) had to be converted for intraoperative complications in 13.3% and for inflammatory changes or unclear anatomy in 10%. LC in benign gallbladder tumors was successfull in 100%. Intraoperative complication rate was 30% in gallbladder carcinoma and 16.6% in benign gallbladder lesions. Morbidity was 18.4% in malignant and 3% in benign lesions. Reoperation rate in gallbladder cancer was 15.8% (10.5% for liver resection, 5.3% for complications), in benign gallbladder lesions 0%. 30-day mortality was 0 in both groups.
*Conclusions:* LC is the treatment of choice for suspected benign gallbladder lesions with a 0% conversion rate, a 0% reoperation rate and low morbidity. Laparoscopic treatment for malignant or suspected malignant gallbladder lesions leads to a high primary and secondary conversion rate, a high morbidity and to reoperation in 15.8%. We therefore recommend an open approach in all preoperatively suspected or known gallbladder cancer and a primary or secondary conversion after laparoscopic diagnosis of malignant gallbladder lesions. Wether more extended resectional procedures are indicated remains open for discussion

### F267

EXPERIMENTAL AND CLINICAL BASIS FOR THE TARGETED DELIVERY OF ANTIBIOTICS IN BILIARY SURGERY

Zh. Sh. Zhumadilov
Semipalatinsk Medical Institute, Kazakstan

The possibility of the use and effectiveness of targeted delivery of antibiotics by autological erythrocyte ghosts has been studied experimentally on 20 noninbred dogs and applied clinically in 200 patients with acute complicated cholecystitis and high risk of urgent operation, in 21 patients with biliary abscesses of the liver, in 45 patients with acute cholangitis. The control group of 65 patients received traditional therapy. We studied the effect of the new method on the immune,clinical and biochemical status of patients. We infused intravenously ghosts containing single dose of antibiotic twice daily. It was found that the new method made it possible to provide a high concentration of antibiotic for prolonged periods in the liver and biliary ducts, in comparison with traditional methods. No septic complications of disease or urgent operation was noted in patients with acute cholecystitis. Our method resulted in a threefold reduction in the duration of treatment of patients with biliary abscesses of the liver. The amelioration of immunological and clinical states was more marked in patients receiving targeted delivery of antibiotic. On the basis of this analysis, we conclude that the new method is effective, economical, and will increase the quality of biliary surgery.

